# Review of Eyeless *Pseudosinella* Schäffer (Collembola, Entomobryidae, and Lepidocyrtinae) from Brazilian Caves

**DOI:** 10.3390/insects11030194

**Published:** 2020-03-19

**Authors:** Nikolas G. Cipola, João Victor L. C. Oliveira, Bruno C. Bellini, Aila S. Ferreira, Estevam C. A. Lima, Roniere A. Brito, Luis C. Stievano, Paolla G. C. Souza, Douglas Zeppelini

**Affiliations:** 1 Laboratório de Sistemática e Ecologia de Invertebrados do Solo, Instituto Nacional de Pesquisas da Amazônia—INPA, COBIO/Entomologia, Campus II, Petrópolis, Manaus 69067-375, Brazil; 2 Programa de Pós-Graduação em Ciências Biológicas—Zoologia, Universidade Federal da Paraíba, João Pessoa 58051-900, Brazil; jv_lemos@hotmail.com.br (J.V.L.C.O.); roniere_ra@hotmail.com (R.A.B.); zeppelini@daad-alumni.de (D.Z.); 3 Laboratório de Sistemática de Collembola e Conservação—Coleção de Referência de Fauna de Solo—CCBSA—Universidade Estadual da Paraíba campus V, João Pessoa 58070-450, Brazil; aillasoares@gmail.com (A.S.F.); estevam.araujo@gmail.com (E.C.A.L.); luis@joaopessoa.net (L.C.S.); 4 Programa de Pós-Graduação em Sistemática e Evolução, UFRN, Natal 59072-970, Brazil; entobellini@gmail.com (B.C.B.); paollasouzac@gmail.com (P.G.C.S.); 5 Laboratório de Collembola, Departamento de Botânica e Zoologia, Centro de Biociências, Universidade Federal do Rio Grande do Norte—UFRN, BR 101, Lagoa Nova, Campus Universitário, Natal 59072-970, Brazil; 6 Laboratório de Apterygotologia, Departamento de Entomologia, Museu Nacional, Universidade Federal do Rio de Janeiro, Rio de Janeiro 20940-040, Brazil

**Keywords:** caves conservation, idiochaetotaxy, new species, species group, taxonomy, troglofauna, troglomorphism

## Abstract

Herein, eyeless *Pseudosinella* species from Brazilian caves are reviewed, including the description of 23 new species, new records plus additional notes on the descriptions of *P. ambigua* Zeppelini, Brito, and Lima and of *P. guanhaensis* Zeppelini, Brito, and Lima. We also provide an identification key to 27 eyeless species recorded from Brazil. To organize the 26 Brazilian eyeless taxa analyzed in this work, we organize them in apparently artificial groups: 11 species have one larger tooth on the unguiculus outer lamella (*petterseni* group); one presents unguiculus outer lamella smooth or serrated (never with a larger tooth), with 9 held prelabral chaetae undivided and the last 6 held prelabral chaetae bifurcated. The Brazilian species of eyeless *Pseudosinella* herein described present a remarkably conservate dorsal chaetotaxy; therefore, the main diagnostic characters are related to other features like prelabral, labral, and ventral head chaetotaxy and empodial complex morphology. In addition, our study suggests that Brazilian caves possibly shelter a great diversity of *Pseudosinella* taxa, several of them potentially cave dependent.

## 1. Introduction

*Pseudosinella* Schäffer, 1897 is the largest genus of Collembola (Entomobryidae), with 327 nominal species, representing half of the Lepidocyrtinae species [1,2] and almost 4 % of the world’s 9000 known Collembola species [3]. Out of this, a total of 53 species were described from the Neotropical Region and only 19 of them were from South America [4,5,6,7,8].

In Brazil, eight species of *Pseudosinella* were recorded before [8], of which four are doubtful records since these species are from North America (except *P. biunguiculata* Ellis, 1967 from Guatemala) with old descriptions lacking many diagnostic characters: *P. alba* (Packard, 1873); *P. octopunctata* Börner, 1901; and *P. dubia* Christiansen, 1960 [7,9,10,11]. On the other hand, the species originally described from Brazil are *P. brevicornis* Handschin, 1924 from Santa Catarina State; *P. triocellata* Nunes and Bellini, 2018 from Piauí State; *P. ambigua* Zeppelini, Brito, and Lima, 2018; and *P. guanhaensis* Zeppelini, Brito, and Lima, 2018 from Minas Gerais State, the latter two from caves [12,13,14].

Most described Lepidocyrtinae species from Brazil are epigeic; therefore, this small number of *Pseudosinella* species described/recorded suggests that this taxon is neglected in caves environments [8,10,13,15,16]. This may be true since *Pseudosinella* has wide distribution, holding several troglomorphic taxa (140 from all 600 known troglobite Collembola species—about 23 % of them), inhabiting aphotic environments such as caves, grottos, and deep soil layers [17,18].

Cave *Pseudosinella* have morphological modifications/adaptations to such environments, like elongated appendages, modified claws, and reduction of pigmentation and eyes [17,18]. The eye reduction is the main feature to arbitrarily separate *Pseudosinella* from *Lepidocyrtus* Bourlet, 1839 taxa, combined to few other arguable characters [5,7,19,20]. Nevertheless, neither morphology nor molecular data support the monophyly of *Pseudosinella* [21,22,23].

Based on dorsal macrochaetotaxy and unguiculus morphology (with or without an outer tooth), some species groups were proposed to *Pseudosinella*, although many characters have either been ignored or are unknown to several species [20,24,25,26,27]. In addition, it is necessary to reveal characters with phylogenetic signal to establish (or refute) the supposed groups, since most troglomorphic traits are adaptive convergences shared in different groups of cavernicolous Collembola and may have arisen independently among *Pseudosinella* branches as well [17].

The cave environments have relevance for both evolutionary explanations and biodiversity in small and large scales [18]. For this reason, there is a great need to survey and describe the subterranean fauna of springtails from Brazil. Such data is pivotal for Brazilian environmental conservation policies [14]. For instance, there are at least 15 Brazilian Collembola species included in the Red List due to some level of threat of extinction, of which 14 inhabit caves [28]. These caves are in process of mineral exploration, which can cause alteration or destruction of natural habitats [14].

Herein, we review and describe eyeless *Pseudosinella* species from Brazilian caves. We present the description of 23 new species, additional notes on *P. ambigua* and *P. guanhaensis*, an identification key for the Brazilian species, and a discussion concerning possible species groups.

## 2. Materials and Methods

The studied specimens were preserved in slides deposited at the Reference Collection of Soil Fauna, Paraíba State University (CRFS-UEPB). Part of the slides were reassembled in water and Nesbitt’s solution and then remounted on glass slides in Hoyer’s medium [29]. Specimens in ethanol gel were photographed using a stereomicroscope (M165C) attached to a DFC420 digital camera with a dome [30]. Photographs were digitally corrected using Leica Application Suite V3.4.1. For scanning electron microscopy (SEM), specimens were transferred to absolute ethanol and critical-point dried after sputter-coating with gold using the equipment BAL-TEC CPD 030 and BAL-TEC SPD 050, respectively. The images were made using a scanning electron microscope LEO VP 435. The type material of the new species is deposited at the CRFS-UEPB and at the Invertebrate Collection of National Institute for Amazon Research (INPA).

**Figure 1:**
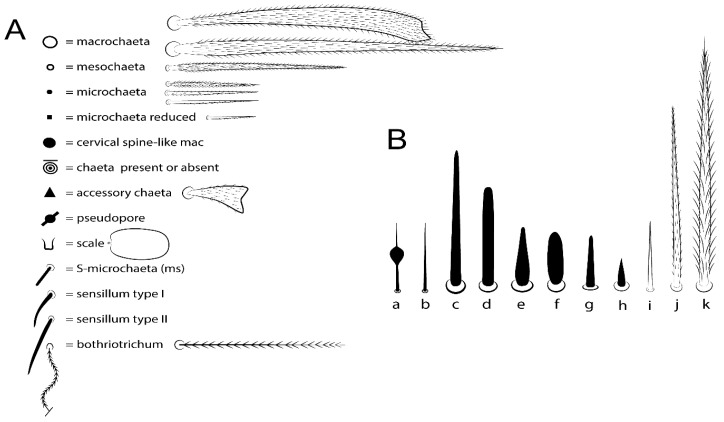
Symbols and chaetae drawings used for chaetotaxy descriptions of *Pseudosinella* species. (**A**) dorsal chaetotaxy; (**B**) Ant I–IV segments.

The terminology used in descriptions follows mainly labial papillae, maxillary palp, and basolateral and basomedian labial [31], using Gisin’s system to a1–5 chaetae labels [32]; chaetotaxy of the labral [33], clypeal [34], postlabial [35,36], and subcoxae outer side [37]; morphology of unguis and unguiculus lamellae [38], genital plate of male and female [39,40]; dorsal chaetotaxy of the head and body [20,41,42]; and specialized chaetae (S-chaetae) [2]. Symbols used to depict the chaetotaxy are presented in Figure 1. All chaetotaxy are given of the left side of the body only.

Abbreviations: Abd-abdominal segment(s); Ant-antennal segment(s); ae-antero-externa lamella; ai-antero-interna lamella; a.t.-unguis apical tooth; b.a.-basal anterior tooth of unguis; b.p.-basal posterior tooth of unguis; b.c.-basal chaeta(e); l.p.-lateral process of labial papilla E; lpc-labial proximal chaeta(e); mac-macrochaeta(e); mes-mesochaeta(e); mic-microchaeta(e); ms-specialized microchaeta(e); m.t.-unguis median tooth; pi-postero-internal lamella; pe-postero-external lamella; psp-pseudopore(s); sens-specialised ordinary chaeta(e); Th―thoracic segment(s); t.a.-terminal appendage of the maxillary palp.

## 3. Results

### 3.1. Definition of Morphological Characters

#### 3.1.1. Characters Shared by *Pseudosinella* Species Described Here

**Figure 2:**
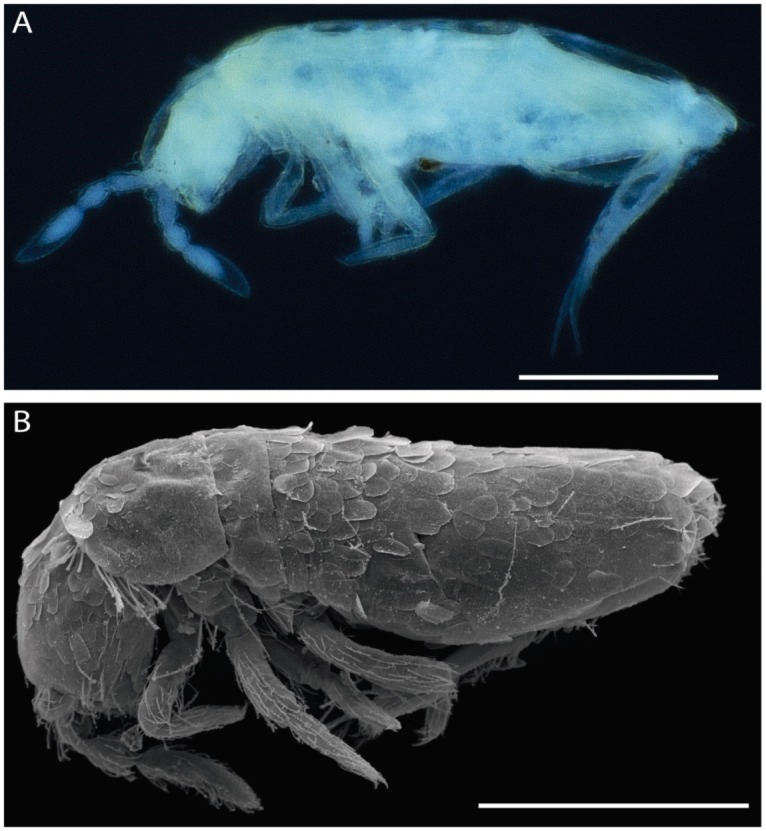
*Pseudosinella* spp.: habitus in lateral view. (**A**) specimen fixed in ethanol; (**B**), metalized specimen. Scale bars: 0.2 mm.

**Figure 3:**
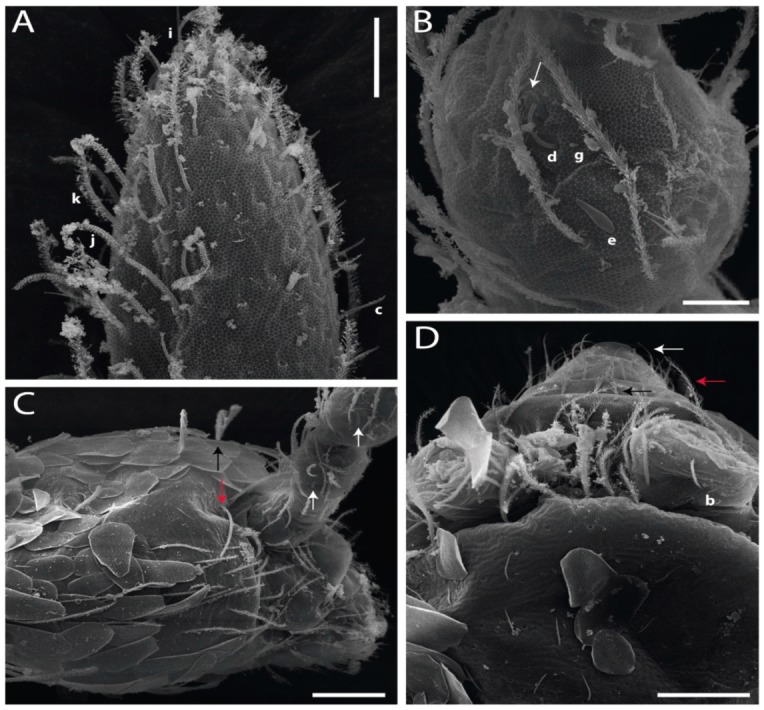
*Pseudosinella* spp.: SEM of head parts. (**A**) Ant IV distal part (dorsal view); (**c**,**i**–**k**) are chaetae types; (**B**) Ant III (lateral view); arrow indicates apical organ, (**d**,**e**,**g**) are sens types; (**C**) head and Ant I–III (lateral view); white arrows show sens e on Ant II and III, black arrow indicates mac apically foot-shaped, and red arrow is mac apically acuminate; (**D**) chaetotaxy of clypeus (including prelabral area), labrum, and head anteriorly (dorsal view); b type sens on Ant I: white arrow indicates labral chaetae, black arrow is prelabral chaetae, and red arrow indicates l2 clypeal chaeta. Scale bars: 0.01 mm (**A**), 0.005 mm (**B**), and 0.02 mm (**C**–**D**).

- Habitus typical of Lepidocyrtinae; specimens pale, without pigments (Figure 2).
- Head. Antennae shorter than body length (Figure 2); Ant IV not annulated, without apical bulb, smooth chaetae distally, two types of ciliate chaetae (weakly and normal), and s-blunt sens of different sizes (Figure 1A, Figure 3A, and Figure 12A). Ant I dorsally with 3 smooth sens (type b) at the base. Eyeless (Figure 3C). Head Pa6 bothriotrichum present; head posterior region with one transversal row of cervical spine-like mac ciliate and apically pointed (Figure 3C and Figure 6C). Labral and prelabral formula with 4 (a1–2), 5 (m0–2), and 5 (p0–2)/4 chaetae (Figure 3D and Figure 8B). Labral papillae typically absent (Figure 19B). Labial palp with five main papillae (A–E) plus one hypostomal papilla (H) with 0, 5, 0, 3–4, 4, and 2 guard appendages, respectively; labial papilla E with l.p.; and labium with 5 lpc (Figure 12E). Maxillary palp with smooth terminal appendage (t.a.) and basal chaeta (b.c.); sublobal plate internally with 3 smooth appendages and distally with 1 minimum smooth appendage (Figure 8D).
- Legs. Trochanteral organ chaetae discretely serrate (seen only in SEM images) (Figure 5A). Tibiotarsus distally with 1 tenent hair on outer side and 1 smooth chaetae on inner side of tibiotarsus III (Figure 5C). Pretarsus with one small anterior and posterior chaetae (Figure 7B,C). Unguis outer side with one pair of lateral teeth and one unpaired proximal dorsal tooth; unguiculus with 4 lamellae (ai, ae, pi, and pe) (Figures 5B,C and 7B,C).
- Tenaculum with 4 teeth on each ramus; corpus with 1 basal ciliate chaeta apically acuminate.
- Genital plate. Male plate multisetaceous, with 8 + 8 circumgenital and 2 + 2 eugenital smooth chaetae, all of similar length (Figure 51H). Female with two pairs (superior and inferior) of small smooth chaetae, without other modifications (Figure 33G).
- Furcula. Manubrium ventrally with 2 inner ciliate chaetae, with the outer chaetae larger (Figure 11B). Dens dorsally crenulate and without spines and proximal tubercle (Figure 5D). Mucro bidentate with 1 basal smooth spine (Figure 11D).

#### 3.1.2. Antennal Chaetae

Antennal segments with about 11 types of chaetae (see Figure 1B); a–h as sens and i–k as chaetae: type a short and thin, apically acuminate and with median region swollen, present subapically on Ant IV; type b short and thin, apically acuminate, present on Ant I and IV; type c finger-shaped gently pointed in apex, present densely on Ant I–IV; type d finger-shaped, present on Ant III–IV; type e conical, present on Ant II–IV; type f ball, present on Ant III; type g guard sens, present on Ant III; type h ms-like sens present on Ant IV; type i smooth present on Ant I–IV; type j weakly ciliate, present on Ant I–IV; type k heavily ciliate, present on Ant I–IV.

#### 3.1.3. Tergal Chaetae

**Figure 4:**
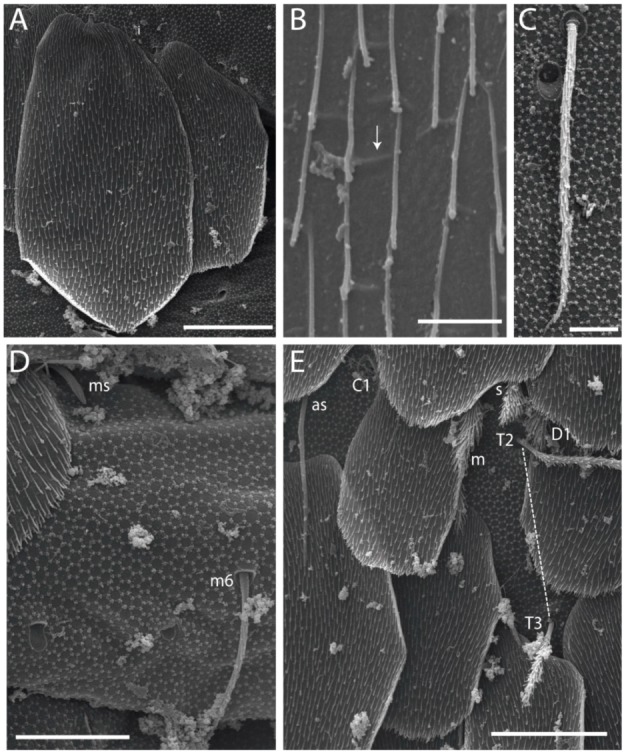
*Pseudosinella* spp.: SEM of thorax and abdomen. (**A**) Tergal scales; (**B**) scale part, with the arrow indicating interciliary connection; (**C**) ciliate mic on Th II; (**D**) Abd I lateral area (right side), where ms is sens and m6 is mic; (**E**) Abd IV area (right side), as elongated sens, C1 mac, T2 bothriotrichum, T3 ciliate mic, and D1, with m and s fan-shape accessory chaetae. Scale bars: 0.01 mm (**A**,**E**), 0.001 mm (**B**), 0.002 mm (**C**), and 0.005 mm (**D**).

**Figure 5:**
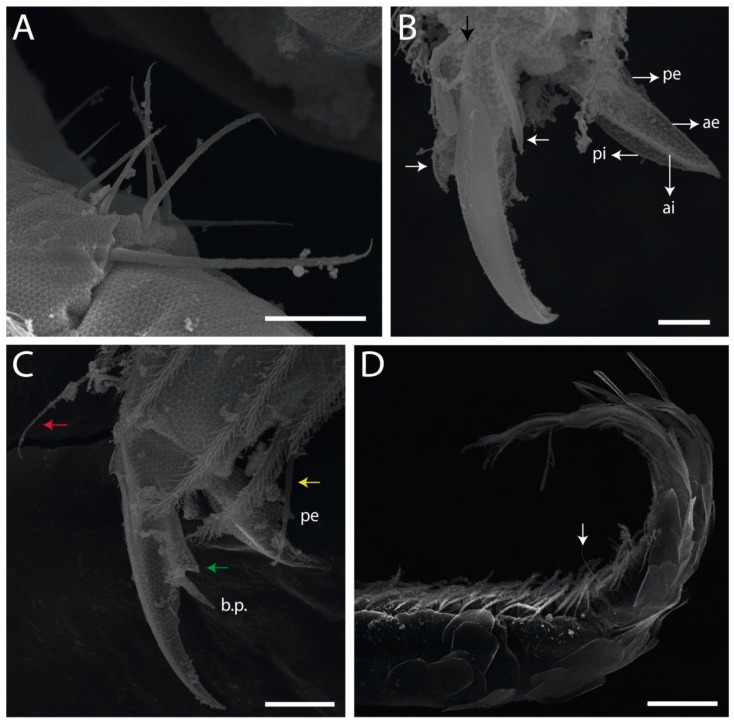
*Pseudosinella* spp.: SEM of trunk appendages. (**A**) trochanteral organ (inner side); (**B**) empodial complex II (anterior side); white arrows on unguis outer side indicate paired hook-shaped teeth; black arrow indicates unpaired median tooth; and ae, ai, pi, and pe are unguiculus lamellae; (**C**) distal tibiotarsus and empodial complex III (anterior side); red arrow indicates tenent hair on tibiotarsus outer side, yellow indicates smooth chaeta on tibiotarsus inner side; green indicates paired hook-shaped teeth; b.p. is posterior basal tooth; and pe is unguiculus lamella; (**D**) furcula (lateral view): arrow indicates smooth chaetae on proximal dens. Scale bars: 0.005 mm (**A**,**C**), 0.002 mm (**B**), and 0.02 mm (**D**).

- Bothriotricha (Figure 1A and Figure 4E). Thin and densely ciliate, short on head (Pa6), elongated on Abd II–III (a5) and Abd IV (T2), others subequal. Abd II–IV bothriotrichal formulas 2 (a5 and m2), 3 (a5, m2, and m5), and 2 (T2 and T4), respectively (Figure 9B,C).
- Bothriotricha accessory chaeta (Figure 1A, Figure 4E, and Figure 9B,C). Fan-shaped and heavily ciliate present on Abd II–IV (mi, ml, lm, ll, li, im, em, a, s, m, pe, and pi).
- Mac (Figure 1A, Figure 3C, and Figure 7A). Finely ciliate and apically acuminate or foot-shaped, present on dorsal head and Th II to Abd V.
- Mes (Figure 1A). Heavily ciliate and apically acuminate, present on head antennal series and Th II to Abd V.
- Mic (Figure 1A, Figure 3D, Figure 4C–E). Unilaterally or fully, weakly or heavily ciliate and apically acuminate of similar lengths present on different regions of the body, on the head, and AMP (anterior, median, posterior) series of Th II to Abd V (Figures 8E and 9).
- Ordinary ms (Figure 1A and Figure 4D), smooth, and apically conical. Ordinary sens (Figure 1A and Figure 4E); smooth and apically rounded; short (type I) and elongated (type II); and present on Th II–III (al), Abd II–III (as), Abd IV (as, ps) and Abd V (as, acc.p4, acc.p5). Th II–Abd V with ms and sens formulas 1, 0/1, 0, 0, 0, 0 and 1, 1/0, 1, 1, +, 3, respectively (Figure 9A–C).
- Scales (Figure 4A,B). Heavily ciliate, with cilia short, uniform, and rounded at the apex and with weak interciliary connections. Scales oval or elongated and apically rounded or truncate (rarely irregular), present on both head sides, Th II to Abd VI, and furcula ventrally (Figure 2B, Figure 3C, Figure 4D,E, and Figure 5D).

#### 3.1.4. Tibiotarsal Modified Chaetae

Tibiotarsus I–III with 7 types of modified mac present on proximal half (as in Figure 10E–G); tibiotarsus I with 1 mac on inner side, tibiotarsus II–III with 1 mac on inner, anterior and outer side (Figure 10E–G):

- Type I (Figure 63E): Finely ciliate and finger-shaped, with rounded apex.
- Type II (Figure 10G): Finely ciliate and finger-shaped, with pointed apex abruptly.
- Type III (Figure 51E): Finely ciliate and finger-shaped, abruptly pointed in the apex and with 1 small smooth filament.
- Type IV (Figures 39E, 54E, and 57E): Finely ciliate and lance-shaped, unilaterally acuminate in the apex.
- Type V (Figure 42E): Heavily ciliate with weakly pointed apex.
- Type VI (Figure 45E): Finely ciliate and lance-shaped, pointed at the apex.
- Type VII (Figure 60E): Heavily ciliate and finger-shaped, abruptly dilated in the apex [43] (p. 47, Figure 169).

### 3.2. Species Group with Outer Tooth on Unguiculus “pe” Lamellae

#### 3.2.1. *Pseudosinella guanhaensis* Zeppelini, Brito, and Lima, 2018

**Figure 6:**
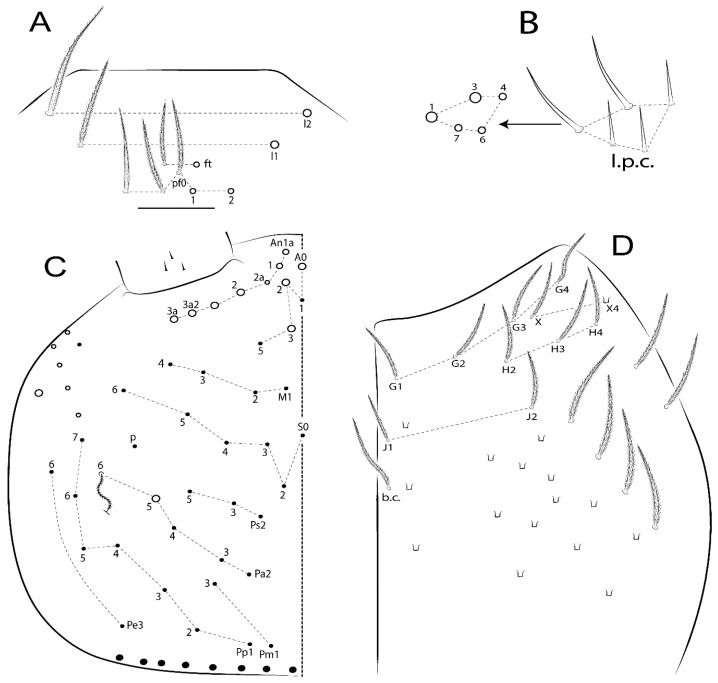
*Pseudosinella guanhaensis*: head. (**A**) Chaetotaxy of the clypeus; (**B**) labial proximal chaetae, arrow indicates proximal chaetae nomenclature; (**C**) head dorsal chaetotaxy (left side); and (**D**) postlabial chaetotaxy (right side).

**Figure 7:**
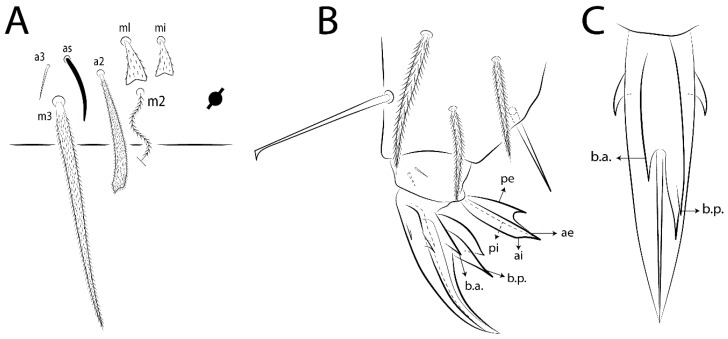
*Pseudosinella guanhaensis*. (**A**) Abd II central chaetotaxy; (**B**) distal tibiotarsus and empodial complex III (anterior view); and (**C**) unguis (inner view).

Figure 6 and Figure 7, Table 1 and Table 2

*Pseudosinella guanhaensis* Zeppelini, Brito, and Lima, 2018, p. 73, Figures 26–39, Brazil, Minas Gerais, Dores de Guanhães (orig. descr.) [14].

*Examined type material*. Holotype and paratype deposited in CRFS-UEPB (10507, 10508).

*Description complement and corrections*. Ant III smaller than Ant II length. Clypeal formula with 4 (l1–2), 2 (ft), and 5 (pf0–2) ciliate chaetae; l1 and pf2 larger acuminate; l1–2 larger; and others subequal (Figure 6A). Prelabral chaetae ciliate and not bifurcate; labral chaetae smooth, a1–2 thicker, others subequal. Labial papilla D with 3 appendages; papilla E with l.p. conical, curved, and not reaching the base of the apical appendage. Head dorsal chaetotaxy (Figure 6C) with 7 “An” (An1a–3a), 5 “A” (A0–3 and A5), 4 “M” (M1–4), 6 “S” (S0, S2–6), 3 “Ps” (Ps2–3, Ps5), 4 “Pa” (Pa2–5), 2 “Pm” (Pm1 and Pm3), 7 “Pp” (Pp1–7), and 2 “Pe” (Pe3 and 6) chaetae; An1a–1, An2–3a, A0, A2–3, and Pa5 as mac; interocular p mic present; and head posterior region with 8 cervical like-spine mac. Basomedian and basolateral labial fields with a1–5 smooth; M1, E, and L1–2 unilaterally ciliate; r reduced; and M2 absent. Labial proximal chaetae smooth, 2 subequal (lpc1 and 3) and 3 gently smaller (lpc4 and 6–7) (Figure 6B). Ventral chaetotaxy with about 17 ciliate chaetae (lateral spine absent), postlabial formula 4 (G1–4), 1 (X), 3 (H2–4), and 2 (J1–2) ciliate chaetae, X4 as scale, b.c. present (Figure 6D). Abd II with 2 central mac (a2 and m3), with a2 short and m3 elongated, typically; as sens is larger, mi and ml are fan-shaped (Figure 7A). Th II–Abd IV formula with 00|020 + 21 + 1 mac. Subcoxa I with 2 chaetae and 2 psp; subcoxa II with “a” row of 7 chaetae, “p” row with 5 chaetae, and 2 psp; subcoxa III with one row of 6 chaetae and 2 posterior psp. Tibiotarsus I–III formula with 1, 2, 2 finger-shaped mac (basal outer mac absent) type I (as Figure 63E). Unguis outer side with paired teeth developed and hook-shaped; inner side with slender lamella and 2 unequal basal teeth; with the b.p. tooth larger; and with 1 smaller split tooth posteriorly, the b.a. tooth smaller, and m.t. and a.t. absent. Unguiculus with all smooth and acuminate lamellae (ai, ae, pi, and pe), except ai gently excavated distally and pe with outer tooth (Figure 7B,C). Collophore anterior side with 6 ciliate chaetae apically acuminated, 1 median larger, others subequal; posterior side distally with 1 smooth chaeta, 1 ciliate chaeta, and 1 subdistal reduced spine; lateral flap with 6 smooth chaetae (2 larger). Manubrium with 2 rows of 4 smooth chaetae each, plus 1 proximal smooth chaeta on dens; ventrally with 2 subapical and about 7 distal scales; manubrial plate with 4 ciliate chaetae (2 inner mac) and 2 psp.

*Remarks.* In the original description, *P. guanhaensis* was not compared with *P. biunguiculata* Ellis, 1967; *P. federicoi* Simón-Benito and Palacios-Vargas, 2008; and *P. violenta* (Folsom, 1924) [27,43,44,45]. These species are similar by head Pa5 mac present and M2 absent, Th II–III devoid of mac, Abd II a2 mac present, and Abd IV with 2 central mac (Table 1 and Table 2). However, *P. guanhaensis* clearly differs from these last two species in head A2 and A3 mac present (absent in both), basomedian and basolateral labial fields with M1, E, and L1–2 ciliate (smooth in *P. federicoi*) plus M2 chaeta absent (present in both). *Pseudosinella guanhaensis* and *P. biunguiculata* also differ by Abd II m3e mac absent (present in both) and Abd IV C1 mac present (absent in both). Thus, *P. guanhaensis* is even more similar to *P. biunguiculata* by Ant III with conical sens (type e), basomedian labial field devoid of M2 chaeta, Abd II with 2 central mac (a2, m3), Abd IV B6 mac absent, manubrium dorsally with 2 rows of 4 smooth chaetae, and dens basis with 1 smooth chaeta. However, *P. guanhaensis* differs from this species by Ant III with 3 conical sens next to sense organ (1 in *P. biunguiculata*), Ant II with 2 lateroventral sens apically capitate (normal in *P. biunguiculata*), and tenent hair capitate (acuminate in *P. biunguiculata*) [43,44]. Another difference observed in *P. biunguiculata* from specimens from “Fernando de Noronha” is lpc1 and lpc7 chaetae being subequal and lpc3-4 and lpc6 being gently smaller, while in *P. guanhaensis*, lpc1 and lpc3 are subequal and lpc4 and lpc6–7 are gently smaller. From our extensive sampling, it is suggested here that *P. guanhaensis* is a species restricted to caves, while *P. biunguiculata* has wide Neotropical distribution, present in Guatemala, Puerto Rico, and Brazil [8].

#### 3.2.2. Pseudosinella biunguiculata Ellis, 1967

Table 1 and Table 2

*Pseudosinella biunguiculata* Ellis, 1967:103, Figure 6 and Figure 7, Guatemala (orig. descr.) [44].

*Examined material.* Three males, 2 females, and 1 juvenile in slides (15150–53, 15156, 15158/CRFS-UEPB): Brazil, Fernando de Noronha Island, “Cacimba do Padre”, 03°51′02″ S, 32°26′18″ W, forest, 25.vii.2012, pitfall-trap, ECA Lima and AS Ferreira coll. One male and 2 females in slides (INPA): same date as above. Eighteen specimens in alcohol (INPA): same date as above, except Sancho Beach, 03°51′18″ S, 32°26′35″ W, 31.vii.2012.

*Geographical records.* Guatemala, Puerto Rico, and Brazil in Espirito Santo state and Fernando de Noronha archipelago (new records).

*Remarks.* Some characteristics were omitted in the descriptions of *P. biunguiculata*, and it was observed that praelabral, labral, postlabial, and collophore chaetotaxy of the Fernando de Noronha specimens are as in *P. guanhaensis*.

#### 3.2.3. *Pseudosinella acantholabrata* sp. nov. Cipola

**Figure 8:**
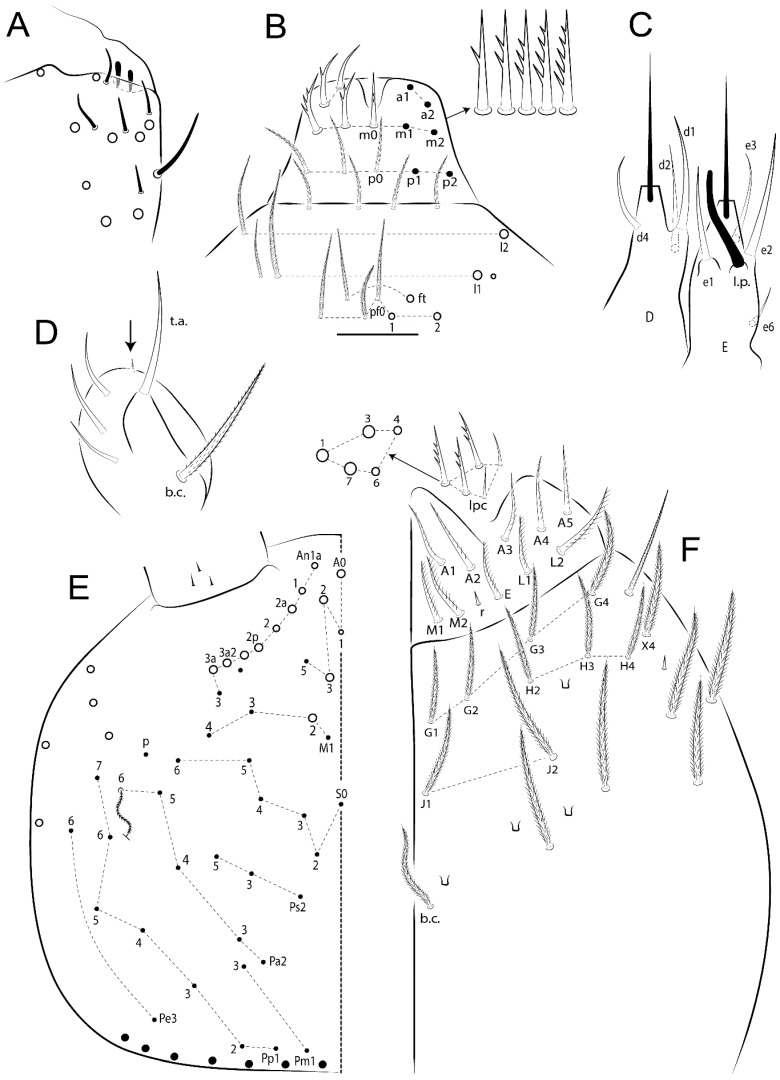
*Pseudosinella acantholabrata* sp. nov.: head. (**A**) Ant III apical organ (lateral view); (**B**) chaetotaxy of clypeus, prelabrum, and labrum; (**C**) labial papillae; (**D**,**E**) (right side); (**D**) maxillary palp and sublobal plate (right side), arrow indicates minute smooth appendage; (**E**) head dorsal chaetotaxy (left side); (**F**) labial proximal chaetae, basomedian, and basolateral labial fields and complete postlabial chaetotaxy (right side), where the arrow indicates proximal chaetae nomenclature.

**Figure 9:**
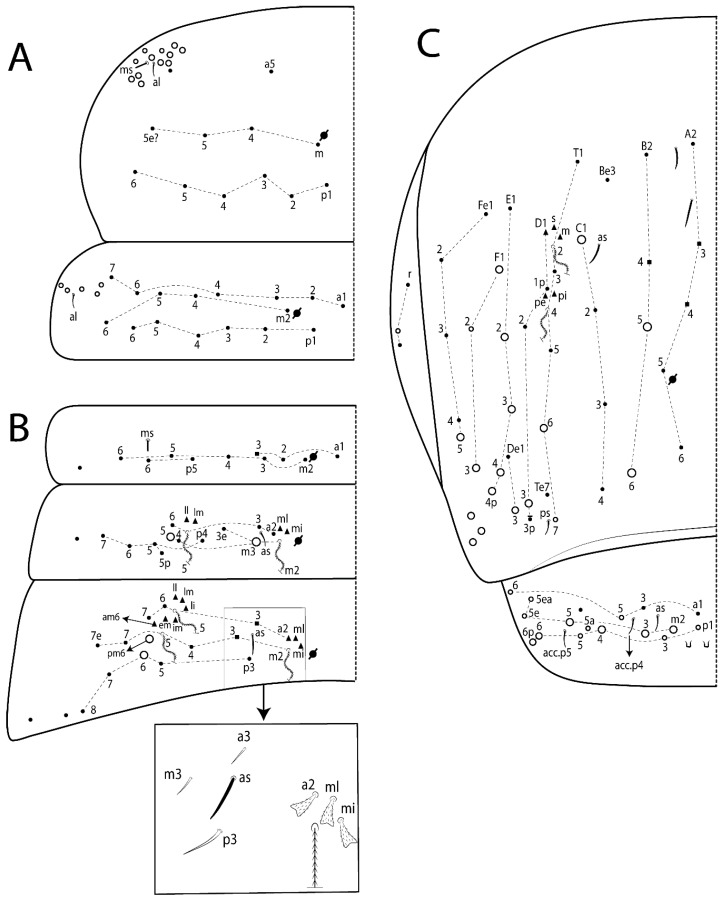
*Pseudosinella acantholabrata* sp. nov.: dorsal chaetotaxy. (**A**) Th II–III; (**B**) Abd I–III, arrow indicates part of Abd III central chaetotaxy; and (**C**) Abd IV–V.

**Figure 10:**
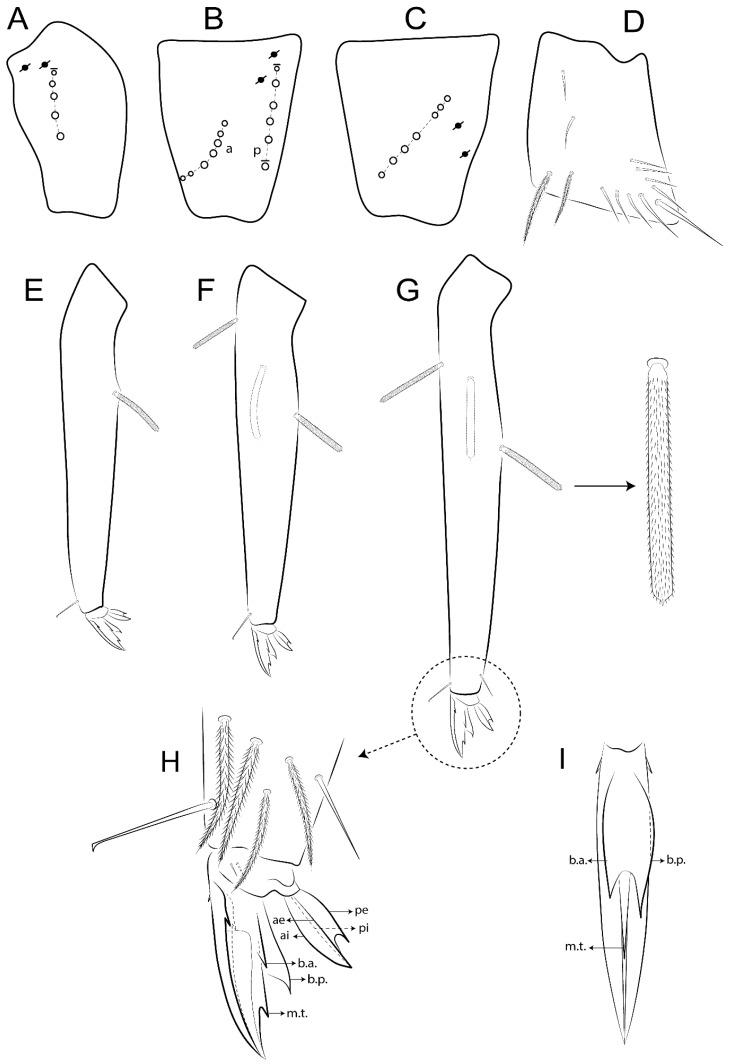
*Pseudosinella acantholabrata* sp. nov.: legs chaetotaxy. (**A**–**C**) subcoxa I–III, respectively; (**D**) trochanteral organ; (**E**–**G**) tibiotarsus I–III, respectively (anterior view), arrow indicates modified chaetae; (**H**) distal tibiotarsus and empodial complex III (anterior view); and (**I**) unguis (inner view).

**Figure 11:**
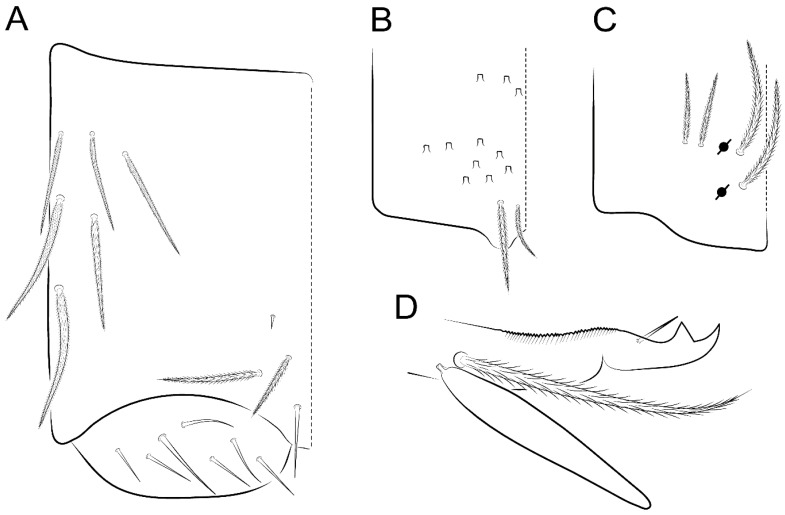
*Pseudosinella acantholabrata* sp. nov. (**A**) collophore (lateral view); (**B**) distal part of manubrium (ventral view); (**C**) manubrial plate (dorsal view); and (**D**) distal dens and mucro (outer view).

Figure 8, Figure 9, Figure 10 and Figure 11, Table 1 and Table 3

*Type material.* Holotype female in slide (10661/CRFS-UEPB): Brazil, Minas Gerais State, Conceição do Mato Dentro municipality, “São Sebastião do Bom Sucesso” burg, Serra da Serpentina, next to road MG-010, 18°56′46.1″ S, 43°24′25.6″ W, 790 m, 31.v-12.vii.2016, Carste et al. coll. Paratypes in slides (9727–28, 9735–36, 10500–01, 10642, 10661/CRFS-UEPB): 1 male, 4 females, and 3 juveniles, same data as holotype. Paratypes in slides (9334, 10598/CRFS-UEPB): 2 female, same data as holotype, except 20.vii-06.viii.2015 and 22.xi-15.xii.2016, respectively. Paratypes in slides (13463, 13491/CRFS-UEPB): 1 male and 1 female, same data as holotype, except 30.iv–05.v.2018. Paratypes in slides (11291–92/CRFS-UEPB donated to INPA/076): 2 females, same data as holotype, except 29.ii–04.iii.2016.

*Typological observation.* Two juveniles designated here as paratypes of *P. acantholabrata* sp. nov. were published as additional material of *P. ambigua* [14].

*Description.* Total length (head + trunk) of holotype 0.96 mm.

Head. Ratio antennae:trunk = 1:2.51 in holotype; Ant III subequal or gently smaller than Ant II length; Ant segments ratio as 1:1.54:1.61:3.05 in holotype. Ant IV dorsally with 1 sens a and numerous sens (b–c) and chaetae (i–k), other type of sens absent on Ant II–III. Ant III with 2 apical sens clubs surrounded by at least 1 sens c and 4 g (Figure 8A). Clypeal formula with 6 (l1, 1e–2), 2 (ft), 5 (pf0–2) ciliate chaetae, all apically acuminate, except pf2 normal, l1–2 larger (Figure 8B). Prelabral chaetae ciliate and not bifurcate. Labral a1–2 and m0–2 chaetae thicker, a1 and a2 laterally with 1–2 median filaments (a1 sometimes without filament), m0 with 2–3 median filaments, and m1–2 with 1–5 median filaments, p0–2 chaetae ciliate (Figure 8B). Labial papilla D with 3 appendages; papilla E with l.p. finger-shaped, sinuous, and surpassing the base of apical appendage (Figure 8C). Maxillary palp with b.c. weakly ciliate and 1.38 longer than the t.a. (Figure 8D). Head dorsal chaetotaxy (Figure 8E) with 9 “An” (An1a–3), 5 “A” (A0–3 and A5), 4 “M” (M1–4), 6 “S” (S0 and S2–6), 3 “Ps” (Ps2–3 and Ps5), 4 “Pa” (Pa2–5), 2 “Pm” (Pm1 and Pm3), 7 “Pp” (Pp1–7), and 2 “Pe” (Pe3 and 6) chaetae; An1a–3a, A0, A2–3, and M2 as mac; interocular p mic present; head posterior region with 7 cervical like-spine mac. Basomedian and basolateral labial fields with A1–5 unilaterally and weakly ciliate; M1–2, E, and L1–2 unilaterally ciliate; and r reduced. Labial proximal chaetae (lpc1, lpc3–4, and lpc6–7) with 2–3 filaments and subequal, except lpc4 and lpc6 smooth and smaller (Figure 8F). Ventral chaetotaxy with about 17 ciliate chaetae, 1 lateral spine reduced, and 1 chaeta weakly ciliate; postlabial formula 4 (G1–4), 1 (X4), 3 (H2–4), and 2 (J1–2) chaetae; G4 and X4 plus 3 lateral chaetae thicker; and b.c. present (Figure 8F).

Thorax dorsal chaetotaxy (Figure 9A). Th II a, m, and p series with 1 (a5), 4 (m–5e?) and 6 (p1–6) mic, respectively. Th III a, m, and p series with 6 (a1–4 and a6–7), 4 (m2 and m4–6), and 6 (p1–6) mic, respectively. Ratio Th II:III = 2.22:1 in holotype.

Abdomen dorsal chaetotaxy (Figure 9B,C). Abd I a, m, and p series with 3 (a1–3), 5 (m2–6), and 2 (p5–6) mic, respectively, and a3 as reduced mic. Abd II a, m, and p series with 3 (a2–3 and a6), 4 (m3–3e and m4–5), and 5 (p4–7) chaetae, respectively, and m3 and m5 as mac; a5 and m2 bothriotricha with 2 (lm and ll) and 2 (ml and mi) fan-shaped chaetae, respectively. Abd III a, m, and p series with 4 (a2–3 and a6–7), 6 (m3–4, am6, pm6, and m7–7e), and 5 (p3 and p5–8) chaetae, respectively, and pm6 and p6 as mac; a3 and m3 as reduced mic; and as sens elongated, ms absent; m2 bothriotricha associated with 3 (a2, ml, and mi) and a5 and m5 bothriotricha associated with 6 (am6, li, lm, ll, im, and em) fan-shaped chaetae. Abd IV A–Fe series with 5 (A2–6), 4 (B2 and B4–6), 1 (Be3), 4 (C1–4), 5 (T1, T3, and T5–7), 1 (Te7), 4–5 (D1–3p), 2 (De1 and De3), 5 (E1–4p), 3 (F1–3), and 5 (Fe1–5) chaetae, respectively, with 3 central (B5–6 and C1) and 13 lateral mac (T6, D3, De3, E2–4p, F1, F3, Fe5, plus 3 unnamed); T2 and T4 bothriotricha with 3 (D1, s, and m) and 2 (pe and pi) fan-shaped chaetae, respectively; 4 sens present, ps type I, as and 2 anteriorly type II, and r series laterally with 3 chaetae. Abd V a, m, and p series with 0, 3 (m2–3 and m5), and 3 (p4 and 6–6p) mac, respectively. Ratio Abd III:IV = 1:3.71 in holotype. Th II–Abd IV formula with 00|010 + 21 + 2 mac.

Legs. Subcoxa I with 4–5 chaetae and 2 psp; subcoxa II with “a” row of 7 chaetae, “p” row with 4–6 chaetae, and 2 psp; subcoxa III with one row of 7 chaetae and 2 posterior psp (Figure 10A–C). Trochanteral organ with about 11 spine-like chaetae (Figure 10D). Tibiotarsus I–III formula with 1, 3, and 3 mac type II (Figure 10E–G). Unguis outer side with paired teeth straight, not developed, and on the proximal one fourth; inner side with wide lamella and 3 teeth, basal pair unequal, b.p. tooth larger but not reaching the m.t. apex, m.t. on the distal one thirds and subequal to b.a. tooth length, with a.t. absent. Unguiculus with all lamellae smooth and acuminate (ai, ae, pi, and pe), except pe with outer tooth (Figure 10H,I); ratio unguis:unguiculus in holotype = 1:0.50. Tibiotarsal smooth chaeta about 0.83 smaller than unguiculus, tenent hair capitate, and about 0.49 smaller than unguis outer edge.

Collophore (Figure 11A). Anterior side with 6 ciliate apically acuminate chaetae, 3 proximal thin and subequal in length, and 3 subdistal larger; posterior side distally with 1 smooth chaeta, 2 ciliate chaetae (outer chaetae larger), and 1 subdistal reduced spine; lateral flap with 7 smooth chaetae, with 1 larger.

Furcula. Only with ciliate chaetae and scales. Manubrium ventrally with 2 subapical and about 8 distal scales; manubrial plate with 4 ciliate chaetae (2 inner mac) and 2 psp (Figure 11B,C). Mucro basal tooth gently larger than distal tooth, basal spine reaches the apex of basal tooth (Figure 11D).

*Etymology.* Refers to labral spines of new species (from Greek: acantho-spine).

*Remarks. Pseudosinella acantholabrata* sp. nov. resembles *P. serpentinensis* sp. nov. by prelabral, labral, and collophore chaetotaxy; head M2 mac present; Th II–Abd IV with 0, 0, 0, 1, 0, and 3 central mac; unguis with 3 inner tooth unequal, being b.p. undivided (Table 1 and Table 3). However, *P. acantholabrata* sp. nov. differs from this species by labial papilla E with l.p. finger-shaped and sinuous (conical and straight in *P. serpentinensis* sp. nov.), maxillary palp with b.c. completely and weakly ciliate (unilaterally ciliate in *P. serpentinensis* sp. nov.), postlabial X chaeta absent (present in *P. serpentinensis* sp. nov.), and labral a2 and m0 chaetae with filaments and m1–2 chaetae with up to 5 filaments, while in *P. serpentinensis* sp. nov. a2 and m0 are smooth and m1–2 smooth or with maximum of 1 filament. They also differ by tenent hair capitate (acuminate in *P. serpentinensis* sp. nov.) and unguiculus ai lamellae acuminate (gently excavate in *P. serpentinensis* sp. nov.). Superficially, *P. acantholabrata* sp. nov. also resembles *P. rolfsi* Mills, 1932 by Th II–Abd III with 0, 0, 0, 1, and 0 central mac and tenent hair capitate (Table 1, Table 2 and Table 3), but the new species differs by head A2 and A3 mac present (absent in *P. rolfsi*), Abd II with m3 mac but m3e absent (opposite in *P. rolfsi*), and Abd IV with 3 central mac (1–2 in *P. rolfsi*) [7,20,49].

#### 3.2.4. *Pseudosinella brumadinhoensis* sp. nov. Cipola

**Figure 12:**
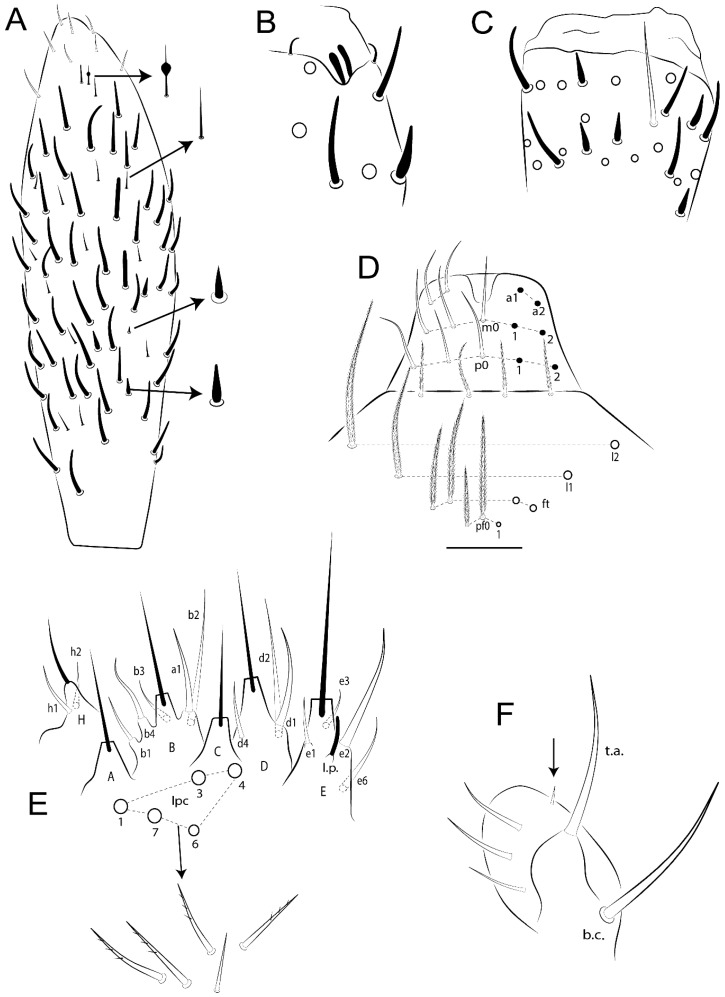
*Pseudosinella brumadinhoensis* sp. nov.: head. (**A**) Ant IV dorsal chaetotaxy, arrows indicate different types of sens; (**B**) Ant III apical organ (lateral view); (**C**) Ant III ventrodistal chaetotaxy; (**D**) chaetotaxy of clypeus, prelabrum, and labrum; (**E**) Labial papillae A–E and proximal chaetae; (**F**) maxillary palp and sublobal plate (right side), arrow indicates minute smooth appendage.

**Figure 13:**
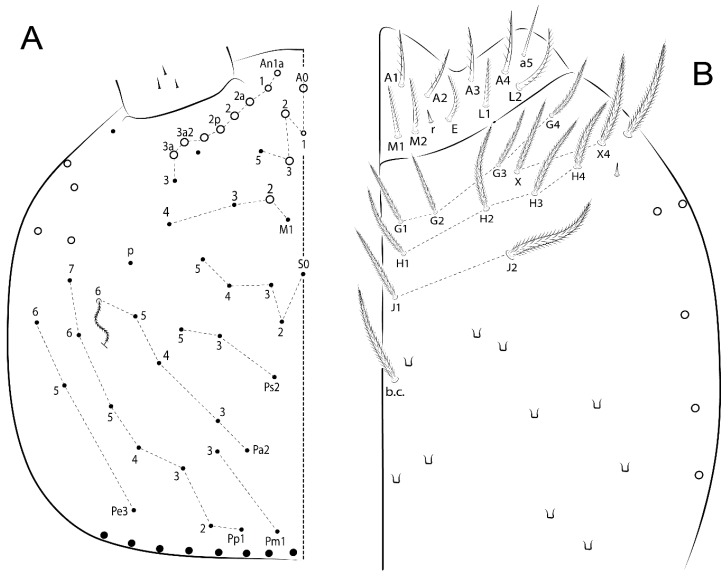
*Pseudosinella brumadinhoensis* sp. nov.: head. (**A**) dorsal chaetotaxy (left side); (**B**) basomedian and basolateral labial fields and complete postlabial chaetotaxy (right side).

**Figure 14:**
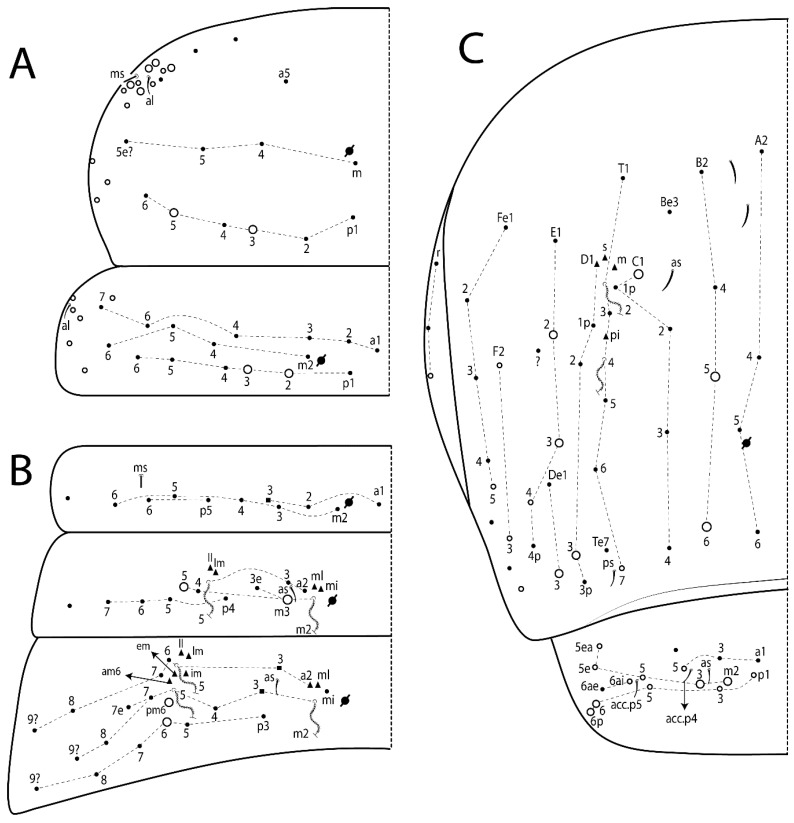
*Pseudosinella brumadinhoensis* sp. nov.: dorsal chaetotaxy. (**A**) Th II–III; (**B**) Abd I–III; (**C**) Abd IV–V.

**Figure 15:**
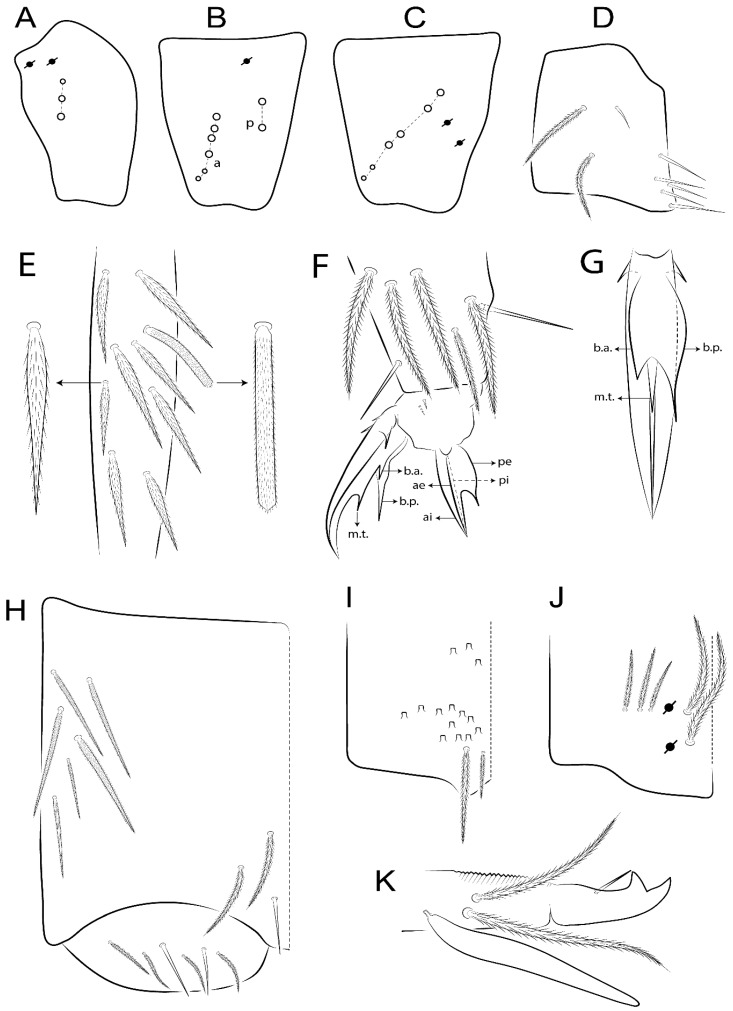
*Pseudosinella brumadinhoensis* sp. nov.: trunk appendages. (**A**–**C**) subcoxa I–III, respectively; (**D**) trochanteral organ; (**E**) tibiotarsus II median part (inner view), arrow indicates scale-like chaeta and modified chaetae, respectively; (**F**) distal tibiotarsus and empodial complex III (anterior view); (**G**) unguis (inner view); (**H**) collophore (lateral view); (**I**) distal part of manubrium (ventral view); (**J**) manubrial plate (dorsal view); (**K**) distal dens and mucro (outer view).

Figure 12, Figure 13, Figure 14 and Figure 15, Table 1 and Table 3

*Type material.* Holotype female in slide (12569/CRFS-UEPB): Brazil, Minas Gerais State, Brumadinho municipality, “Serra da Moeda”, in Vallourec company, “Mina Pau Branco”, 20°09′49.8″ S, 43°58′30.7″ W, 1.560 m, 16.iv.2018, Carste et al. coll. Paratypes in slides (12701, 12759–60, 12767/CRFS-UEPB): 2 females and 2 juveniles, same data as holotype, except 15.vi–17.viii.2018. Paratype in slide (13922/CRFS-UEPB): 1 juvenile, same data as holotype, except 14.xii.2018. Paratypes in slide (12707, 12709/CRFS-UEPB donated to INPA/077): 2 females, same data as holotype, except 15.vi.2018.

*Description.* Total length (head + trunk) of holotype 1.11 mm.

Head. Ratio antennae:trunk = 1:2.26 in holotype; Ant III subequal to Ant II length; Ant segment ratio as I:II:III:IV = 1:2.00:1.93:4.27 in holotype. Ant IV dorsally with 1 sens a and h, 2 d, 3 e, and numerous sens (b–c) and chaetae (i–k) (Figure 12A). Ant III with 2 apical sens clubs surrounded by at least 2 sens c and g and 1 e; ventrally with at least 4 c and 5 e (Figure 12B,C). Ant II dorsally without sens apparently, ventrally with at least 2 c and 1 b together. Clypeal formula with 4 (l1–2e), 4 (ft), and 3 (pf0–1) ciliate chaetae; l1–2 larger and apically acuminate; pf1 smaller; and others subequal (Figure 12D). Prelabral chaetae ciliate and not bifurcate. Labral chaetae smooth and subequal, without modifications (Figure 12D). Labial papilla D with 3 appendages; papilla E with l.p. finger-shaped, curved, and not reaching the base of apical appendage (Figure 12E). Maxillary palp with b.c. smooth and 0.93 smaller than the t.a. (Figure 12F). Head dorsal chaetotaxy (Figure 13A) with 9 “An” (An1a–3), 5 “A” (A0–3 and A5), 4 “M” (M1–4), 5 “S” (S0 and S2–5), 3 “Ps” (Ps2–3 and Ps5), 4 “Pa” (Pa2–5), 2 “Pm” (Pm1 and Pm3), 7 “Pp” (Pp1–7), and 3 “Pe” (Pe3 and 5–6) chaetae; An1a–3a, A0, A2–3, and M2 as mac; interocular p mic present; head posterior region with 8 cervical like-spine mac. Basomedian and basolateral labial fields with a5 smooth gently smaller; a1–4 unilaterally and weakly ciliate; M1–2, E, and L1–2 unilaterally ciliate; and r reduced. Labial proximal chaetae (lpc1, 3, 4, and 7) with 3 filaments and subequal, except lpc6 smooth and smaller (Figure 12E). Ventral chaetotaxy with about 19 ciliate chaetae and 1 reduced lateral spine; postlabial formula 4 (G1–4), 2 (X, X4), 4 (H1–4), and 2 (J1–2) chaetae; X4, H4, and J2 plus 6 lateral chaetae thicker; and b.c. present (Figure 13B).

Thorax dorsal chaetotaxy (Figure 14A). Th II a, m, and p series with 1 (a5), 4 (m–5e?), and 4 (p1–2, p4, and p6) mic, respectively, and with p3 and p5 as mac. Th III a, m, and p series with 6 (a1–4 and a6–7), 4 (m2 and m4–6), and 4 (p1 and p4–6) mic, respectively, and with p2 and p3 as mac. Ratio Th II:III = 1.78:1 in holotype.

Abdomen dorsal chaetotaxy (Figure 14B,C). Abd I a, m, and p series with 3 (a1–3), 5 (m2–6), and 2 (p5–6) mic, respectively, and with a3 as reduced mic. Abd II a, m, and p series with 2 (a2–3), 4 (m3–3e and m4–5), and 4 (p4–7) chaetae, respectively, and with m3 and m5 as mac; a5 and m2 bothriotricha with 2 (lm and ll) and 2 (ml and mi) fan-shaped chaetae, respectively. Abd III a, m, and p series with 6 (a2–3 and a6–9?), 8 (m3–4, am6, pm6, m7–7e, and m8–9?), and 6 (p3 and p5–9?) chaetae, respectively, and with pm6 and p6 as mac, a3 and m3 as reduced mic, and as sens not elongated, ms absent; m2 bothriotricha associated with 2 (a2 and ml) and a5 and m5 bothriotricha with 5 (am6, lm, ll, im, and em) fan-shaped chaetae. Abd IV A–Fe series with 4 (A2 and A4–6), 4 (B2 and B4–6), 1 (Be3), 5 (C1–4), 5 (T1, T3, and T5–7), 1 (Te7), 5 (D1–3p), 2 (De1 and De3), 5 (E1–4p), 2 (F2–3), and 5 (Fe1–5) chaetae, respectively, with 3 central (B5–6 and C1) and 4 lateral mac (D3, De3, and E2–3); T2 and T4 bothriotricha with 3 (D1, s, and m) and 1 (pi) fan-shaped chaetae, respectively; 4 sens present, ps type I, as and 2 anteriorly type II, r series laterally with 3 chaetae. Abd V a, m, and p series with 0, 2 (m2–3) and 2 (p6–6p) mac, respectively. Ratio Abd III:IV = 1:3.28 in holotype. Th II–Abd IV formula with 22|010 + 21 + 2 mac.

Legs. Subcoxa I with 3 chaetae and 2 psp; subcoxa II with “a” row of 6 chaetae, “p” row with 2 chaetae and 1 psp; subcoxa III with one row of 6 chaetae and 2 posterior psp (Figure 15A–C). Trochanteral organ with about 6 spine-like chaetae (Figure 15D). Tibiotarsus I–III formula with 1, 3, and 3 mac type II; tibiotarsus I–III internally with some scale-like chaetae (Figure 15E). Unguis outer side with paired teeth developed in hook-shaped and on proximal one fourth; inner side with slender lamella and 3 teeth, basal pair unequal, b.p. tooth surpassing the m.t. apex, m.t. on a little more than the unguis distal half and gently larger to b.a. tooth length, with a.t. absent. Unguiculus with all lamellae smooth and acuminate (ai, ae, pi, and pe), except pe with outer tooth (Figure 15F–G); ratio unguis:unguiculus in holotype = 1:0.55. Tibiotarsal smooth chaeta about 0.84 smaller than unguiculus; tenent hair acuminate and about 0.38 smaller than unguis outer edge.

Collophore (Figure 15H). Anterior side with 6 ciliate chaetae apically acuminate, 1 larger and 1 smaller, others subequal in length; posterior side distally with 1 smooth chaeta and 2 subequal ciliate chaetae, no spines; lateral flap with 2 smooth and 4 ciliate chaetae, all subequal in length.

Furcula. Only with ciliate chaetae and scales. Manubrium ventrally with 3 subapical and about 11 distal scales; manubrial plate with 5 ciliate chaetae (2 inner mac) and 2 psp (Figure 15I–J). Mucro teeth subequal in size, basal spine reaches the apex of basal tooth (Figure 15K).

*Etymology.* The species was named after its type locality, “Brumadinho” municipality, where a mining disaster killed 252 people (there still 18 missing) on 25 January 2019.

*Remarks. Pseudosinella brumadinhoensis* sp. nov. resembles *P. petterseni* Börner, 1901 and *P. labruspinata* sp. nov. by Th II–Abd I with 2, 2, and 0 central mac, Abd III–IV with 0 and 3 central mac, and unguis basal teeth unequal (see Table 1, Table 2 and Table 3). However, both new species differ from *P. petterseni* by basomedian and basolateral labial field with A1–4 unilaterally ciliate (smooth in *P. petterseni*), and tenent hair acuminate (capitate in *P. petterseni*). In dorsal chaetotaxy the new species differs in head A3 mac present and S2 and Pa5 absent (opposite in *P. petterseni*) and Abd II devoid of a2 mac (present in *P. petterseni*) [26,46,47,48]. *Pseudosinella brumadinhoensis* sp. nov. differs from *P. labruspinata* sp. nov. by unmodified chaetae as labral a1–2 and m0–2, a1 and b4 appendages of labial papilla B, and b.c. of the maxillary palp, while *P. labruspinata* sp. nov. has filaments in all these chaetae. Still on head, *P. brumadinhoensis* sp. nov. differs by M2 mac absent (present in *P. labruspinata* sp. nov.). They also differ by unguis b.p. tooth undivided (with 1 split tooth in *P. labruspinata* sp. nov.), unguiculus ai lamella acuminate (truncate in *P. labruspinata* sp. nov.), collophore posteriorly devoid of spine and lateral flap with ciliate and smooth chaetae, while *P. labruspinata* sp. nov. presents 1 spine posteriorly and chaetae with elongated cilia on lateral flap.

#### 3.2.5. *Pseudosinella keni* sp. nov. Cipola

**Figure 16:**
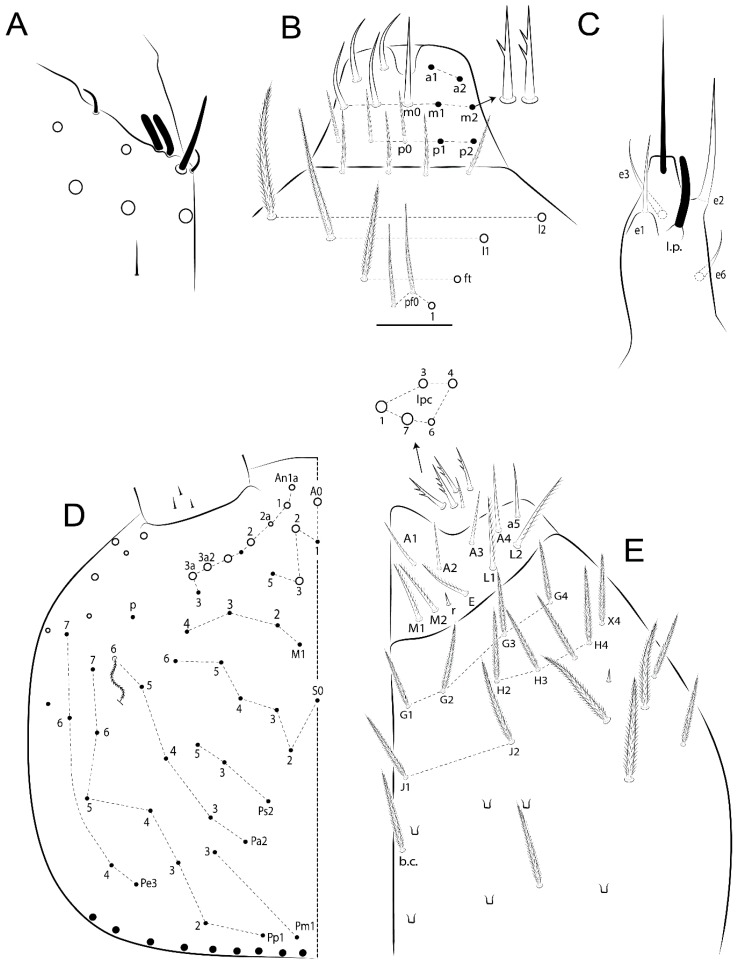
*Pseudosinella keni* sp. nov.: head. (**A**) Ant III apical organ (lateral view); (**B**) chaetotaxy of clypeus, prelabrum and labrum, arrow indicate m1–m2 chaetae with 1 or 2 median filaments; (**C**) labial papilla E (right side); (**D**) head dorsal chaetotaxy (left side); (**E**) labial proximal chaetae, basomedian and basolateral labial fields and complete postlabial chaetotaxy (right side), arrow indicate proximal chaetae nomenclature.

**Figure 17:**
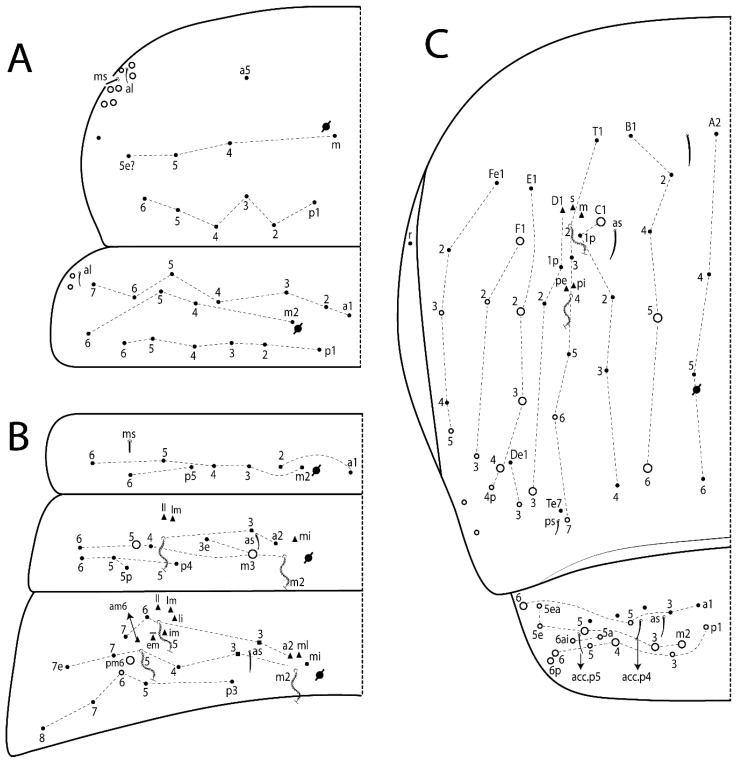
*Pseudosinella keni* sp. nov.: dorsal chaetotaxy. (**A**) Th II–III; (**B**) Abd I–III, arrow indicates part of Abd III central chaetotaxy; (**C**) Abd IV–V.

**Figure 18:**
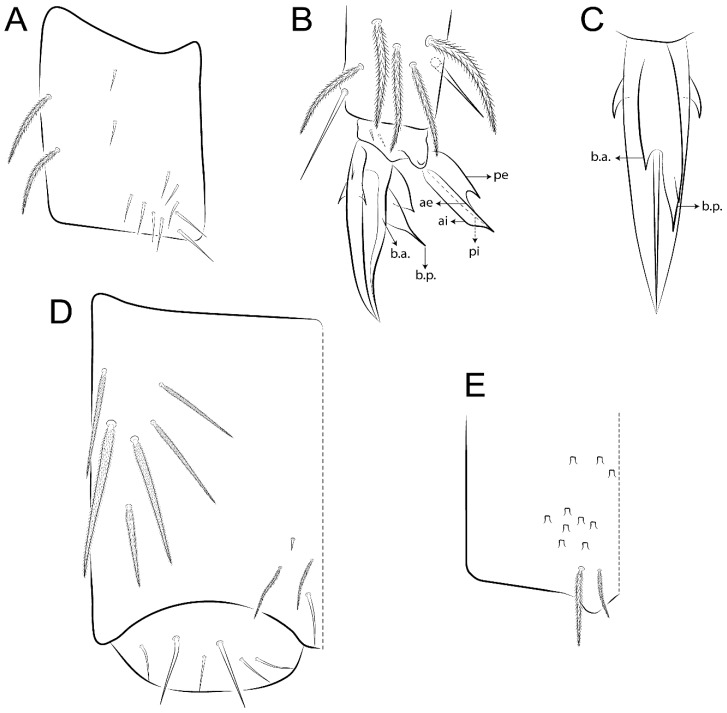
*Pseudosinella keni* sp. nov.: trunk appendages. (**A**) trochanteral organ; (**B**) distal tibiotarsus and empodial complex III (anterior view); (**C**) unguis (inner view); (**D**) collophore (lateral view); (**E**) distal part of manubrium (ventral view).

Figure 16, Figure 17 and Figure 18, Table 1 and Table 3

*Type material.* Holotype female in slide (6094/CRFS-UEPB): Brazil, Minas Gerais State, Rio Acima municipality, Serra do Gandarela National Park, near rural road in mountain top, “SG-51” cavity, 20°05′49.5″ S, 43°40′43.3″ W, 1.269 m, 10.ii–20.iii.2014, Carste et al. coll. Paratype in slide (6095/CRFS-UEPB): 1 female, same data as holotype. Paratypes in slides (10459-60/CRFS-UEPB donated to INPA/078): 1 male and 1 female, same data as holotype, except 14.vii–18.ix.2016. Paratype in slide (6075/CRFS-UEPB): 1 juvenile, same data as holotype, except “SG-37” cavity, 20°06′15.6″ S, 43°40′00.8″ W, 1.282 m. Paratypes in slides (6104, 6162, 6299/CRFS-UEPB): 1 male and 2 females, same data as holotype, except “GAND 8B” cavity, 20°06′19.5″ S, 43°40′23.3″ W, 1.578 m.

*Description.* Total length (head + trunk) of holotype 0.90 mm.

**Figure 19:**
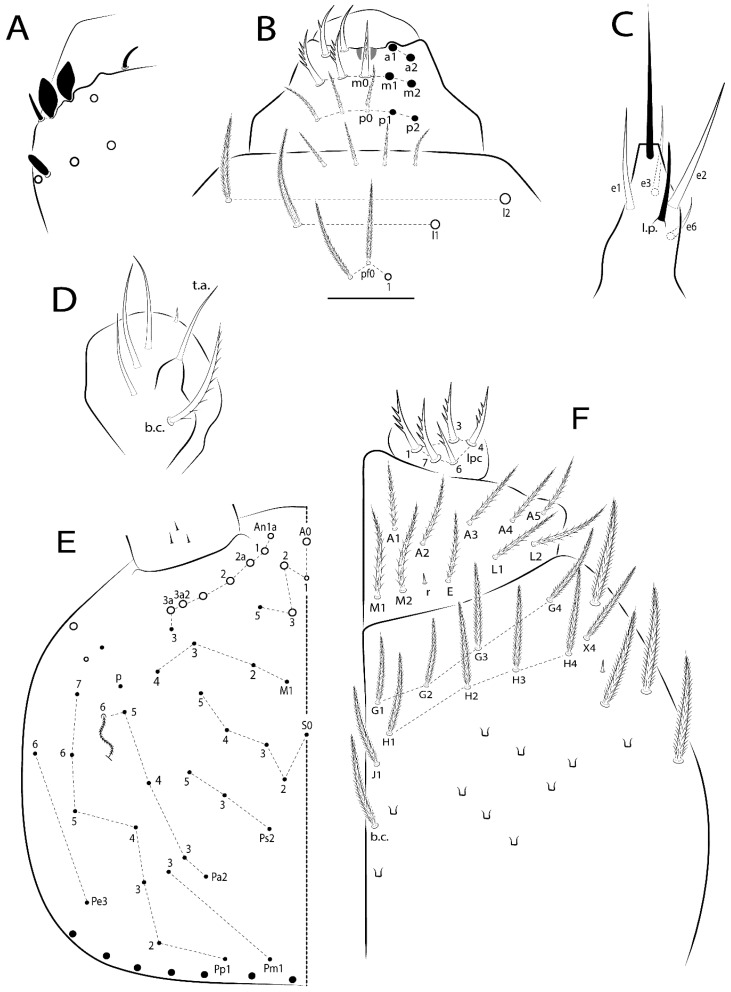
*Pseudosinella labiociliata* sp. nov.: head. (**A**) Ant III apical organ (lateral view); (**B**) chaetotaxy of clypeus, prelabrum, and labrum; (**C**) labial papilla E (right side); (**D**) maxillary palp and sublobal plate (right side); (**E**) head dorsal chaetotaxy (left side); (**F**) labial proximal chaetae, basomedian and basolateral labial fields, and complete postlabial chaetotaxy (right side).

Head. Ratio antennae:trunk = 1:2.63 in holotype; Ant III subequal or gently smaller than Ant II length; Ant segment ratio as I:II:III:IV = 1:2.03:2.00:3.59 in holotype. Ant IV dorsally with 1 sens a and h, 1 e, and numerous sens (b–c) and chaetae (i–k). Ant III with 2 apical sens rods surrounded by at least 1 sens c and 2 g, and 1 subdistal b (Figure 16A); ventrally with at least 2 c and e, and 1 b. Ant II dorsally without sens apparently, ventrally with at least 3 c and 2 b. Clypeal formula with 4 (l1–2), 2 (ft), and 3 (pf0–1) ciliate chaetae, l1–2 larger, l1 and pf0–1 apically acuminate (Figure 16B). Prelabral chaetae ciliate and not bifurcate. Labral a1–2 and m0–2 chaetae thicker, a1–2 and m0 smooth, m1–2 smooth or with 1–2 median filaments, p0–2 chaetae ciliate (Figure 16B). Labial papilla D with 3 appendages; papilla E with l.p. finger-shaped, curved, and reaches the base of apical appendage (Figure 16C). Maxillary palp with b.c. unilaterally and heavily ciliate, 1.20 longer than the t.a. (as Figure 19D). Head dorsal chaetotaxy (Figure 16D) with 9 “An” (An1a–3), 5 “A” (A0–3 and A5), 4 “M” (M1–4), 6 “S” (S0 and S2–6), 3 “Ps” (Ps2–3 and Ps5), 4 “Pa” (Pa2–5), 2 “Pm” (Pm1 and Pm3), 7 “Pp” (Pp1–7), and 4 “Pe” (Pe3–4 and Pe6–6) chaetae; An1a–1, An2–3a, A0, and A2–3; interocular p mic present; head posterior region with 9 cervical like-spine mac. Basomedian and basolateral labial fields with a5 smooth and smaller, A1–4 weakly and unilaterally ciliate; M1–2, E, and L1–2 unilaterally ciliate; and r reduced. Labial proximal chaetae with 2–3 filaments (lpc1, 3, 4, and 7), 2 anterior gently smaller (lpc3–4), and 1 (lpc6) smooth and smaller (Figure 16E). Ventral chaetotaxy with about 18 ciliate chaetae and 1 reduced lateral spine, postlabial formula 4 (G1–4), 1 (X4), 3 (H2–4), and 2 (J1–2) ciliate chaetae, 3 lateral chaetae thicker, and b.c. present (Figure 16E).

Thorax dorsal chaetotaxy (Figure 17A). Th II a, m, and p series with 1 (a5), 5 (m–5e? plus 1 unnamed), and 6 (p1–6) mic, respectively. Th III a, m, and p series with 7 (a1–7), 4 (m2 and m4–6), and 6 (p1–6) mic, respectively. Ratio Th II:III = 1.41:1 in holotype.

Abdomen dorsal chaetotaxy (Figure 17B,C). Abd I a, m, and p series with 2 (a1–2), 5 (m2–6), and 2 (p5–6) mic, respectively, a3 and a6 absent. Abd II a, m and p series with 2 (a2–3), 5 (m3–3e and m4–6), and 4 (p4–6 and p5p) chaetae, respectively, m3 and m5 as mac; a5 and m2 bothriotricha with 2 (lm and ll) and 1 (mi) fan-shaped chaetae (ml absent), respectively. Abd III a, m and p series with 4 (a2–3 and a6–7), 6 (m3–4, am6, pm6, and m7–7e), and 5 (p3 and p5–8) chaetae, respectively, pm6 as mac, p6 as mes, a3 and m3 as reduced mic, as sens elongated, ms absent; m2 bothriotricha associated with 2 (a2 and ml) and a5 and m5 bothriotricha with 5–6 (am6, li, lm, ll, im, and em) fan-shaped chaetae. Abd IV A–Fe series with 4 (A2 and A4–6), 5 (B1–2 and B4–6), 5 (C1–4), 5 (T1, T3, and T5–7), 1 (Te7), 4 (D1–3), 2 (De1 and De3), 5 (E1–4p), 3 (F1–3), and 5 (Fe1–5) chaetae, respectively, with 3 central (B5–6 and C1) and 5 lateral mac (D3, E2–4, and F1); T2 and T4 bothriotricha with 3 (D1, s, and m) and 2 (pe and pi) fan-shaped chaetae, respectively; 3 sens present, ps type I, as and 1 of uncertain homology type II, r series laterally with 1 mic. Abd V a, m, and p series with 1 (a6), 3 (m2–3 and m5), and 3 (p4 and 6–6p) mac, respectively. Ratio Abd III:IV = 1:3.35 in holotype. Th II–Abd IV formula with 00|010 + 11 + 2 mac.

Legs. Subcoxa I with 3 chaetae and 2 psp; subcoxa II with “a” row of 7 chaetae, “p” row with 3 chaetae and 2 psp; subcoxa III with one row of 8 chaetae and 2 posterior psp. Trochanteral organ with about 11 spine-like chaetae (Figure 18A). Tibiotarsus I–III formula with 1, 3, and 3 mac type II (as Figure 10E–G). Unguis outer side with paired teeth developed in hook-shaped and on proximal one third; inner side with slender lamella distally and 2 unequal basal teeth, b.p. tooth larger and with 1 smaller split tooth posteriorly, b.a. tooth smaller; m.t. and a.t. absent. Unguiculus with all lamellae smooth and acuminate (ai, ae, pi, and pe), except ai gently excavate distally and pe with outer tooth (Figure 18B,C); ratio unguis:unguiculus in holotype = 1:0.61. Tibiotarsal smooth chaeta about 0.59 smaller than unguiculus; tenent hair acuminate and about 0.46 smaller than unguis outer edge.

Collophore (Figure 18D). Anterior side with 6 ciliate chaetae apically acuminate, 3 proximal subequal in length, 2 median larger, and 1 distal smaller; posterior side distally with 1 smooth chaeta, 1–2 ciliate chaetae, and 1 subdistal reduced spine; lateral flap with 6 smooth chaetae, 2 larger.

Furcula. Only with ciliate chaetae and scales. Manubrium ventrally with 3 subapical and about 7 distal scales (Figure 18E); manubrial plate with 4 ciliate chaetae (2 inner mac) and 2 psp (as Figure 11C). Mucro teeth subequal in size, basal spine reaches the apex of basal tooth (as Figure 15K).

*Etymology.* “keni” (in apposition) refers to nickname (Ken) of Collembola researcher Dr. Kenneth Christiansen (Grinnell College) who passed away on November 26, 2017.

*Remarks. Pseudosinella keni* sp. nov. resembles *P. labiociliata* sp. nov. by prelabral and labral chaetotaxy, head M2 mac absent, Th II–Abd IV with 0, 0, 0, 1, 0, and 3 central mac, unguis with 2 inner teeth (m.t. absent) unequal, of which b.p. holds 1 splitted tooth (see Table 1 and Table 3). However, *P. keni* sp. nov. differs from this species by Ant III with two sens rods (swollen lance-shaped sens in *P. labiociliata* sp. nov.), clypeal ft chaetae present (absent in *P. labiociliata* sp. nov.), basomedian and basolateral labial fields with 4 (A1–4) chaetae unilateraly ciliate (A1–5 completely ciliate in *P.* *labiociliata* sp. nov.), lpc6 chaetae smooth (with 2 filaments in *P. labiociliata* sp. nov.), labial papilla E with l.p. finger-shaped (pointed in *P. labiociliata* sp. nov.), and labral m0 chaeta smooth and m2 smooth or with up to 2 median filaments, while *P. labiociliata* sp. nov. has m0 with filaments and m2 with up to 5 filaments. They also differ in collophore posteriorly with 2 ciliate chaetae (absent in *P. labiociliata* sp. nov.) and lateral flap only with smooth chaetae (ciliate and smooth in *P. labiociliata* sp. nov.).

The type locaties of *P. keni* sp. nov. and *P. unimacrochaetosa* sp. nov. are 3 km away in Serra do Gandarela National Park. However, these species can be differentiated by *P. keni* sp. nov. the following characters; labral a1–2 chaetae smooth (with filaments in *P. unimacrochaetosa* sp. nov.), basomedian and basolateral labial fields with all chaetae (except A5) unilaterally ciliate (completely ciliate in *P. unimacrochaetosa* sp. nov.), Th II devoid mac (p5 mac in *P. unimacrochaetosa* sp. nov.), and unguis with 2 inner teeth (4 in *P. unimacrochaetosa* sp. nov.)

#### 3.2.6. *Pseudosinella labiociliata* sp. nov. Cipola

**Figure 20:**
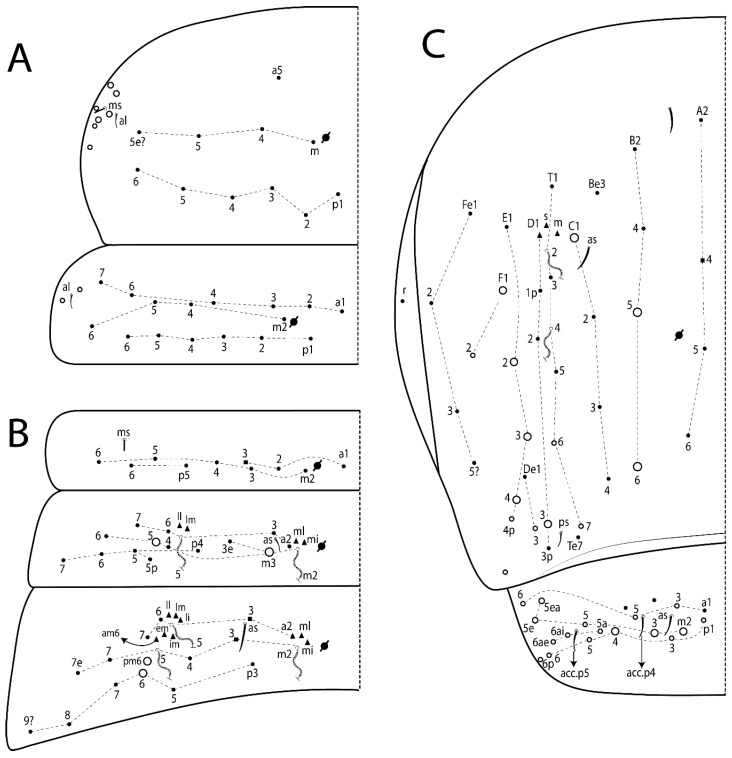
*Pseudosinella labiociliata* sp. nov.: dorsal chaetotaxy. (**A**) Th II–III; (**B**) Abd I–III; (**C**) Abd IV–V.

**Figure 21:**
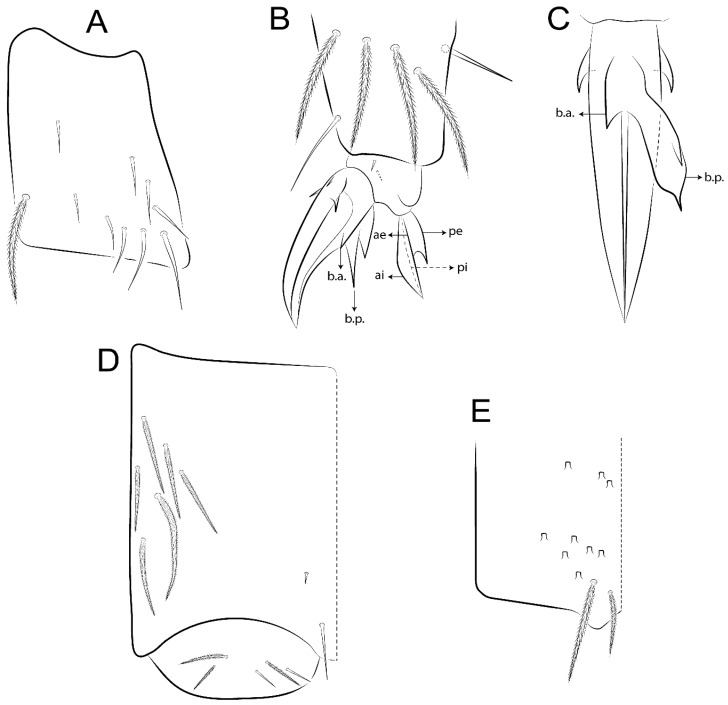
*Pseudosinella labiociliata* sp. nov.: trunk appendages. (**A**) trochanteral organ; (**B**) distal tibiotarsus and empodial complex III (anterior view); (**C**) unguis (inner view); (**D**) collophore (lateral view); (**E**) distal part of manubrium (ventral view).

Figure 19, Figure 20 and Figure 21, Table 1 and Table 3

*Type material.* Holotype female in slide (15196/CRFS-UEPB): Brazil, Minas Gerais State, Caeté municipality, Serra da Piedade, “AVG 71” cavity, 19°49′21.3″ S, 43°41′52.1″ W, 1428 m, 09.vii.2019, entomology aspirator, ECA Lima coll. Paratype in slide (6240/CRFS-UEPB): 1 female, same data as holotype, except 09.iv.2014, Bioespeleo business coll. Paratype in slide (15197/CRFS-UEPB): 1 female, same data as holotype, except “AVG 66” cavity, 19°49′26.6″ S, 43°41′32.3″ W, 1450 m, 11.vii.2019. Paratype in slide (15198/CRFS-UEPB donated to INPA/079): 1 juvenile, idem, except 08.iv.2014

*Description.* Total length (head + trunk) of holotype 1.13 mm.

Head. Ratio antennae:trunk = 1:3.24 in holotype; Ant III subequal to Ant II length; Ant segment ratio as I:II:III:IV = 1:1.83:1.81:3.44 in holotype. Ant IV dorsally with 1 sens a, 2 d and e, and numerous sens (b–c) and chaetae (i–k), sens h absent. Ant III with 2 apical sens swollen lance-shaped surrounded by 2 sens g and 1 f (Figure 19A); ventrally with 2 sens e and 1 b and c. Ant II dorsally with 1 sens c; ventrally with 2 apical sens f and at least 2 sens c. Clypeal formula with 4 (l1–2), 0 (ft), and 3 (pf0–1) ciliate chaetae; l1–2 larger; l1 acuminate; and others subequal (Figure 19B). Prelabral chaetae ciliate and not bifurcate. Labral a1–2 and m0–2 chaetae thicker, a1 smooth, a2 smooth or with 1 median filament, m0–1 with 1–4 median filaments, m2 with 1–5 filaments, p0–2 chaetae ciliate (Figure 19B). Labial papilla D with 3 appendages; papilla E with l.p. weakly pointed, curved and reaches the base of apical appendage (Figure 19C). Maxillary palp with b.c. unilaterally ciliate and 1.55 longer than the t.a. (Figure 19D). Head dorsal chaetotaxy (Figure 19E) with 8 “An” (An1a–3), 5 “A” (A0–3 and A5), 4 “M” (M1–4), 5 “S” (S0 and S2–5), 3 “Ps” (Ps2–3 and Ps5), 4 “Pa” (Pa2–5), 2 “Pm” (Pm1 and Pm3), 7 “Pp” (Pp1–7), and 2 “Pe” (Pe3 and 6) chaetae; An1a–3a, A0, and A2–3 as mac; interocular p mic present; head posterior region with 7 cervical like-spine mac. Basomedian and basolateral labial fields with A1–5, M1–2, E, and L1–2 ciliate and r reduced. Labial proximal chaetae with 3 filaments (lpc1, 3, 4, and 7) and subequal, except lpc6 smaller and with 2 filaments (Figure 19F). Ventral chaetotaxy with about 15 ciliate chaetae and 1 reduced lateral spine, postlabial formula 4 (G1–4), 1 (X4), 4 (H1–4), and 1 (J1) ciliate chaetae, 4 lateral chaetae thicker and b.c. present (Figure 19F).

Thorax dorsal chaetotaxy (Figure 20A). Th II a, m, and p series with 1 (a5), 4 (m–5e?), and 6 (p1–6) mic, respectively. Th III a, m, and p series with 6 (a1–4 and a6–7), 4 (m2 and m4–6), and 6 (p1–6) mic, respectively. Ratio Th II:III = 1.86:1 in holotype.

Abdomen dorsal chaetotaxy (Figure 20B,C). Abd I a, m, and p series with 3 (a1–3), 5 (m2–6), and 2 (p5–6) mic, respectively, a3 as reduced mic. Abd II a, m, and p series with 4 (a2–3 and a6–7), 5 (m3–3e and m4–6), and 5 (p4–7 and p5p) chaetae, respectively, m3 and m5 as mac; a5 and m2 bothriotricha with 2 (lm and ll) and 2 (ml and mi) fan-shaped chaetae, respectively. Abd III a, m, and p series with 4 (a2–3 and a6–7), 6 (m3–4, am6, pm6, and m7–7e), and 6 (p3 and p5–9?) chaetae, respectively, pm6 and p6 as mac, a3 and m3 as reduced mic, as sens elongated, ms absent; m2 bothriotricha associated with 3 (a2, mi, and mi) and a5 and m5 bothriotricha with 6 (am6, li, lm, ll, im, and em) fan-shaped chaetae. Abd IV A–Fe series with 4 (A2 and A4–6), 4 (B2 and B4–6), 1 (Be3), 4 (C1–4), 5 (T1, T3, and T5–7), 1 (Te7), 5 (D1–3p), 2 (De1 and De3), 5 (E1–4p), 2 (F1–2), and 4 (Fe1–3 and Fe5?) chaetae, respectively, with 3 central (B5–6 and C1) and 5 lateral mac (D3, E2–4, and F1); T2 bothriotricha with 3 (D1, s, and m) fan-shaped chaetae; 3 sens present, ps type I, as and 1 of uncertain homology type II, r series laterally with 1 mic. Abd V a, m, and p series with 0, 2 (m2–3), and 1 (p4) mac, respectively. Ratio Abd III:IV = 1:3.40 in holotype. Th II–Abd IV formula with 00|010 + 21 + 2 mac.

Legs. Subcoxa I with 3 chaetae and 2 psp; subcoxa II with “a” row of 7 chaetae, “p” row with 4–6 chaetae and 2 psp; subcoxa III with one row of 6 chaetae and 2 posterior psp. Trochanteral organ with about 9 spine-like chaetae (Figure 21A). Tibiotarsus I–III formula with 1, 3, and 3 mac type II (as Figure 10E–G). Unguis outer side with paired teeth developed in hook-shaped and on proximal one fourth; inner side with slender lamella distally and 2 unequal basal teeth, b.p. tooth larger and with 1 smaller split tooth posteriorly, b.a. tooth smaller; m.t. and a.t. absent. Unguiculus with all lamellae smooth and acuminate (ai, ae, pi, and pe), except ai gently truncate and pe with outer tooth (Figure 21B,C); ratio unguis:unguiculus in holotype = 1:0.48. Tibiotarsal smooth chaeta about 0.98 smaller than unguiculus; tenent hair acuminate and about 0.47 smaller than unguis outer edge.

Collophore (Figure 21D). Anterior side with 6 ciliate apically acuminate chaetae, 1 median larger, others subequal in length; posterior side distally with 1 smooth chaeta and 1 subdistal reduced spine; lateral flap with 3 ciliate (2 larger) and 2 smooth chaetae.

Furcula. Only with ciliate chaetae and scales. Manubrium ventrally with 3 subapical and about 6 distal scales (Figure 21E); manubrial plate with 4 ciliate chaetae (2 inner mac) and 2 psp (as Figure 11B). Mucro basal tooth gently larger than distal tooth, basal spine reaches the apex of basal tooth (as Figure 11D).

*Etymology.* Refers to presence of abundant ciliate chaetae on mouthparts of the new species (Figure 19F).

*Remarks. Pseudosinella labiociliata* sp. nov. resembles *P. keni* sp. nov., but *P. labiociliata* sp. nov. differ from this species by Ant III with two swollen lance-shaped sens, clypeal ft chaetae absent, basomedian and basolateral labial fields with 5 (A1–5) chaetae ciliate, lpc6 with 2 filaments, labial papilla E with l.p. pointed, and labral m0 chaeta with 1–4 filaments and m2 chaeta with up to 5 filaments (Table 3). *Pseudosinella labiociliata* sp. nov. also differ in collophore posteriorly devoid of ciliate chaetae and lateral flap with ciliate and smooth chaetae. See also the comparison these species in remarks of *P. keni* sp. nov. and Table 1 and Table 3.

#### 3.2.7. *Pseudosinella labruspinata* sp. nov. Cipola

**Figure 22:**
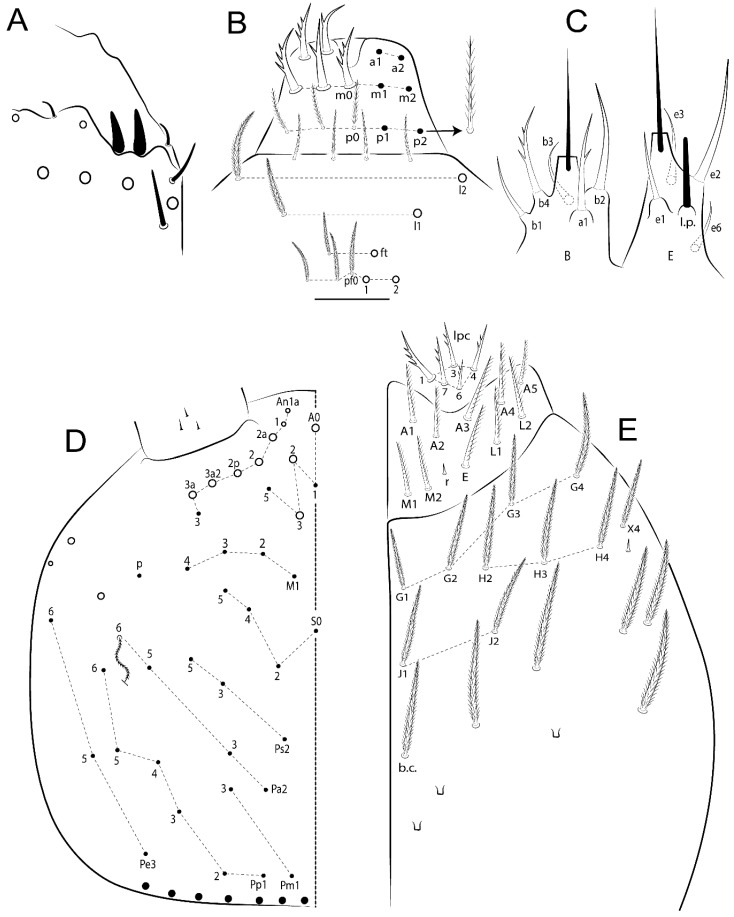
*Pseudosinella labruspinata* sp. nov.: head. (**A**) Ant III apical organ (lateral view); (**B**) chaetotaxy of clypeus, prelabrum, and labrum; (**C**) labial papillae B and E (right side); (**D**) head dorsal chaetotaxy (left side); (**E**) labial proximal chaetae, basomedian and basolateral labial fields, and complete postlabial chaetotaxy (right side).

**Figure 23:**
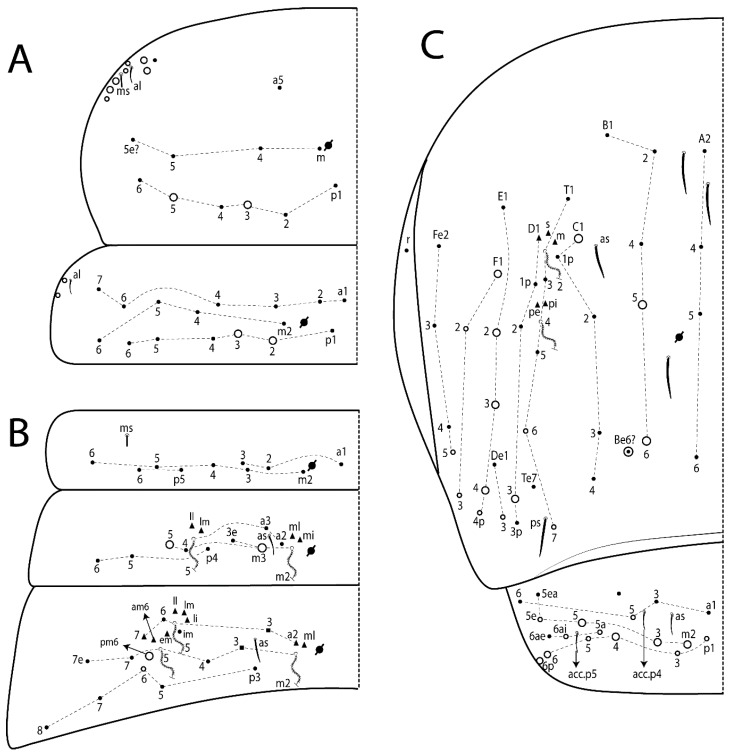
*Pseudosinella labruspinata* sp. nov.: dorsal chaetotaxy. (**A**) Th II–III; (**B**) Abd I–III; (**C**) Abd IV–V.

**Figure 24:**
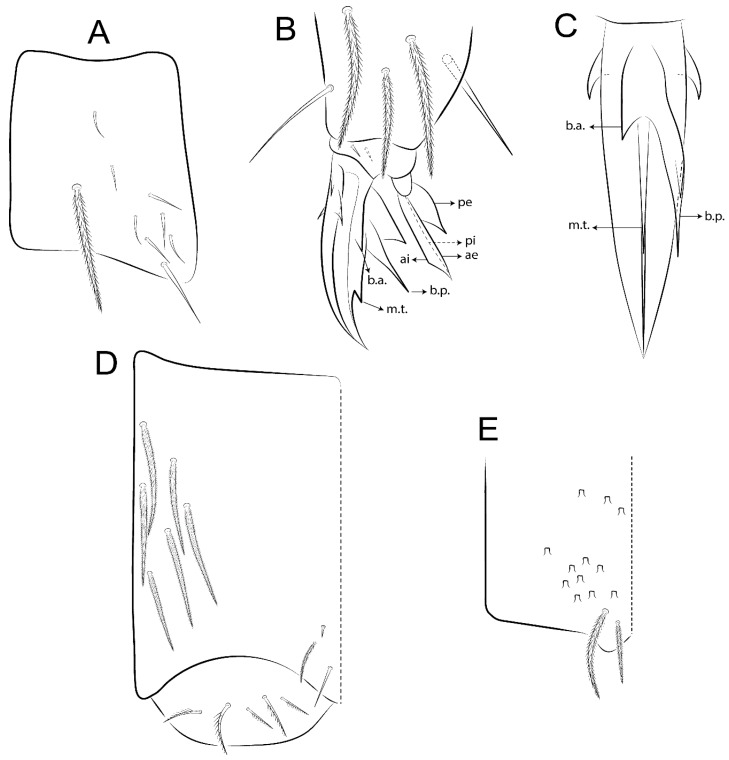
*Pseudosinella labruspinata* sp. nov.: trunk appendages. (**A**) trochanteral organ; (**B**) distal tibiotarsus and empodial complex III (anterior view); (**C**) unguis (inner view); (**D**) collophore (lateral view); (**E**) distal part of manubrium (ventral view).

Figure 22, Figure 23 and Figure 24, Table 1 and Table 3

*Type material.* Holotype female in slide (9757/CRFS-UEPB): Brazil, Minas Gerais State, Itabirito municipality, next to “Mina Várzea Do Lopes Gerdau” in road MG-040, “AS 251” cavity, 20°17′47.6″ S, 43°56′35.5″ W, 1178 m, 01-03.iii.2016, Carste et al. coll. Paratypes in slides (9758-60, 9791, 9866-69/CRFS-UEPB): 1 male, 4 females, and 3 juveniles, same data as holotype. Paratypes in slides (9870, 9921/CRFS-UEPB donated to INPA/080): 2 juveniles, same data as holotype, except “AS 278” and “AS 305” cavities, 05-07.iv.2016 and 05-06.v.2016.

*Description.* Total length (head + trunk) of holotype 0.71 mm.

Head. Ratio antennae trunk = 1:2.64 in holotype; Ant III subequal to Ant II length; Ant segment ratio as I:II:III:IV = 1:1.55:1.45:2.93 in holotype. Ant IV dorsally with 1 sens a and 1 h, 3 d and e, and numerous sens (b–c) and chaetae (i–k). Ant III with 2 apical sens conical surrounded by at least 2 sens c and 2 g (Figure 22A); ventrally with at least 2 c and 1 e. Ant II dorsally with 1 sens h, ventrally with at least 2 c and 1 e. Clypeal formula with 4 (l1–2), 2 (ft), and 5 (pf0–1) ciliate chaetae, l1–2 larger, others smaller (Figure 22B). Prelabral chaetae ciliate and not bifurcate. Labral a1–2 and m0–2 chaetae thicker, a1 with median filament, a2 with 2–3 filaments, m0 with 2–3 filaments, m1 with 1–4 filaments, m2 with 2–4 filaments, p0–2 with elongated cilia (Figure 22B). Labial papilla B with a1 appendage with 1 median filament and b4 appendage with 3 filaments; labial papilla D with 3 appendages; papilla E with l.p. finger-shaped, straight and almost reaches the base of apical appendage (Figure 22C). Maxillary palp with b.c. unilaterally and heavily ciliate, and 1.17 longer than the t.a. (as Figure 19D). Head dorsal chaetotaxy (Figure 22D) with 8 “An” (An1a–3), 5 “A” (A0–3 and A5), 4 “M” (M1–4), 4 “S” (S0, S2, and S4–5), 3 “Ps” (Ps2–3 and Ps5), 3 “Pa” (Pa2–4), 2 “Pm” (Pm1 and Pm3), 6 “Pp” (Pp1–6), and 3 “Pe” (Pe3, Pe5, and Pe7) chaetae; An2a–3a, A0, and A2–3 as mac; interocular p mic present; head posterior region with 7 cervical like-spine mac. Basomedian and basolateral labial fields with A1–5, M1–2, E, and L1–2 unilaterally ciliate and r reduced. Labial proximal chaetae with 2 filaments (lpc1, 3, 4, 6, and 7), except lpc1 thicker and with 3 filaments, lpc6 smaller, others subequal (Figure 22E). Ventral chaetotaxy with about 16 ciliate chaetae and 1 reduced lateral spine, postlabial formula 4 (G1–4), 1 (X4), 3 (H2–4), and 2 (J1–2) ciliate chaetae, 3 lateral chaetae thicker and b.c. present (Figure 22E).

Thorax dorsal chaetotaxy (Figure 23A). Th II a, m, and p series with 1 (a5), 4 (m–5e?), and 4 (p1–2, p4, and p6) mic, respectively, p3 and p5 as mac. Th III a, m, and p series with 6 (a1–4 and a6–7), 4 (m2 and m4–6), and 4 (p1 and p4–6) mic, respectively, p2 and p3 as mac. Ratio Th II:III = 1.72:1 in holotype.

Abdomen dorsal chaetotaxy (Figure 23B,C). Abd I a, m, and p series with 3 (a1–3), 5 (m2–6), and 2 (p5–6) mic, respectively. Abd II a, m, and p series with 2 (a2–3), 4 (m3–3e and m4–5), and 3 (p4–6) chaetae, respectively, m3 and m5 as mac; a5 and m2 bothriotricha with 2 (lm and ll) and 2 (mi and ml) fan-shaped chaetae, respectively. Abd III a, m, and p series with 4 (a2–3 and a6–7), 6 (m3–4, am6, pm6, and m7–7e), and 5 (p3 and p5–8) chaetae, respectively, pm6 as mac, p6 as mes, a3 and m3 as reduced mic, as sens elongated, ms absent; m2 bothriotricha associated with 2 (a2 and ml) and a5 and m5 bothriotricha with 6 (a7, am6, li, lm, ll, and em) fan-shaped chaetae. Abd IV A–Fe series with 4 (A2 and A4–6), 5 (B1–2 and B4–6), 1 (Be6?), 5 (C1–4), 5 (T1, T3, and T5–7), 1 (Te7), 5 (D1–3p), 2 (De1 and De3), 5 (E1–4p), 3 (F1–3), and 4 (Fe2–5) chaetae, respectively, with 3–4 central (B5–6, C1, and Be6? rarely) and 5 lateral mac (D3, E2–4, and F1); T2 and T4 bothriotricha with 3 (D1, s, and m) and 2 (pe and pi) fan-shaped chaetae, respectively; 5 sens type II present, ps, as and 3 of uncertain homology, r series laterally with 1 mic. Abd V a, m, and p series with 0, 3 (m2–3 and m5), and 3 (p4 and 6–6p) mac, respectively. Ratio Abd III:IV = 1:4.28 in holotype. Th II–Abd IV formula with 22|010 + 11 + 2(3) mac.

Legs. Subcoxa I with 3 chaetae and 2 psp; subcoxa II with “a” row of 6 chaetae, “p” row with 2–3 chaetae and 2 psp; subcoxa III with one row of 6 chaetae and 2 posterior psp. Trochanteral organ with about 8 spine-like chaetae (Figure 24A). Tibiotarsus I–III formula with 1, 3, and 3 mac type II (as Figure 10E–G). Unguis outer side with paired teeth developed in hook-shaped and on proximal one third; inner side with wide lamella and 3 teeth, basal pair unequal, b.p. tooth reaches the m.t. apex and with 1 smaller split tooth posteriorly, m.t. on distal one third and subequal to b.a. tooth length, a.t. absent. Unguiculus with all lamellae smooth and acuminate (ai, ae, pi, and pe), except ai truncate distally and pe with outer tooth (Figure 24B,C); ratio unguis:unguiculus in holotype = 1:0.43. Tibiotarsal smooth chaeta about 1.06 longer than unguiculus; tenent hair acuminate and about 0.51 smaller than unguis outer edge.

Collophore (Figure 24D). Anterior side with 6 ciliate apically acuminate chaetae, 1 distal gently smaller, others subequal in length; posterior side distally with 1 smooth chaetae, 1 chaeta weakly ciliate, and 1 subdistal reduced spine; lateral flap with 5 chaetae (2 larger) with elongated cilia.

Furcula. Only with ciliate chaetae and scales. Manubrium ventrally with 3 subapical and about 9 distal scales (Figure 24E); manubrial plate with 4 ciliate chaetae (2 inner mac) and 2 psp (as Figure 11C). Mucro teeth subequal in size, basal spine reaches the apex of basal tooth (as Figure 15K)

*Etymology.* Refers to presence of filaments similar to spines on anterior and median labral chaetae of the new species (Figure 22B).

*Remarks.* The presence of filaments in labral chaetae and head M2 mac absent make *P. labruspinata* sp. nov. similar to *P. keni* sp. nov. and *P. labiociliata* sp. nov., but *P. labruspinata* sp. nov. easily differs from these species by the modifications in a1 and b4 appendages of labial papilla B and Th II–III with 2 (p3 and p5) and 2 (p2–3) mac, respectively. Due to this last feature, *P. labruspinata* sp. nov. is more similar to *P. brumadinhoensis* sp. nov. More comparisons are presented in the remarks of this late species and Table 1 and Table 3.

#### 3.2.8. *Pseudosinella paraensis* sp. nov. Cipola

**Figure 25:**
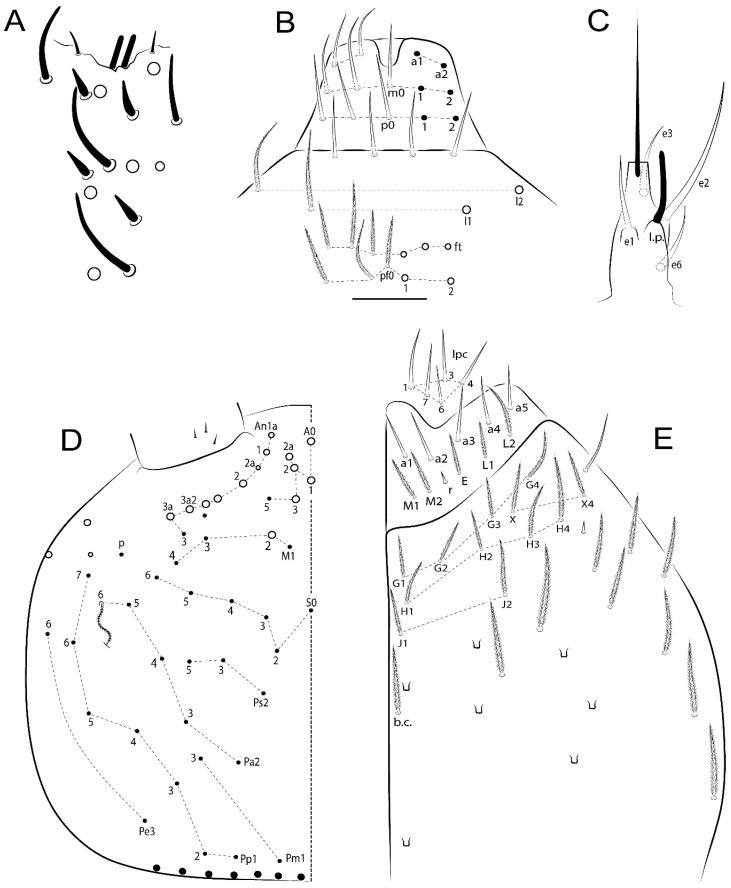
*Pseudosinella paraensis* sp. nov.: head. (**A**) Ant III apical organ (lateral view); (**B**) chaetotaxy of clypeus, prelabrum, and labrum; (**C**) labial papilla E (right side); (**D**) head dorsal chaetotaxy (left side); (**E**) labial proximal chaetae, basomedian and basolateral labial fields, and complete postlabial chaetotaxy (right side).

**Figure 26:**
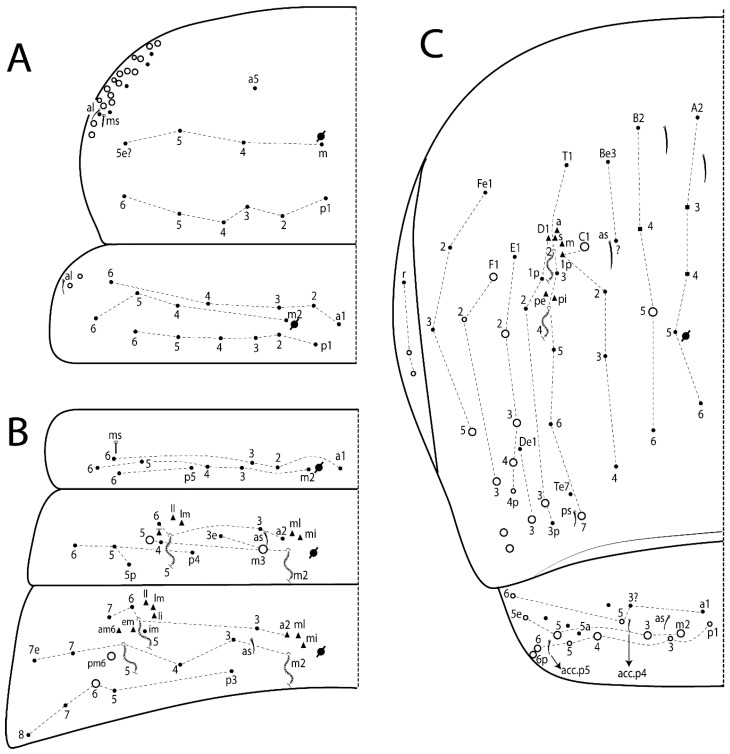
*Pseudosinella paraensis* sp. nov.: dorsal chaetotaxy. (**A**) Th II–III; (**B**) Abd I–III; (**C**) Abd IV–V.

**Figure 27:**
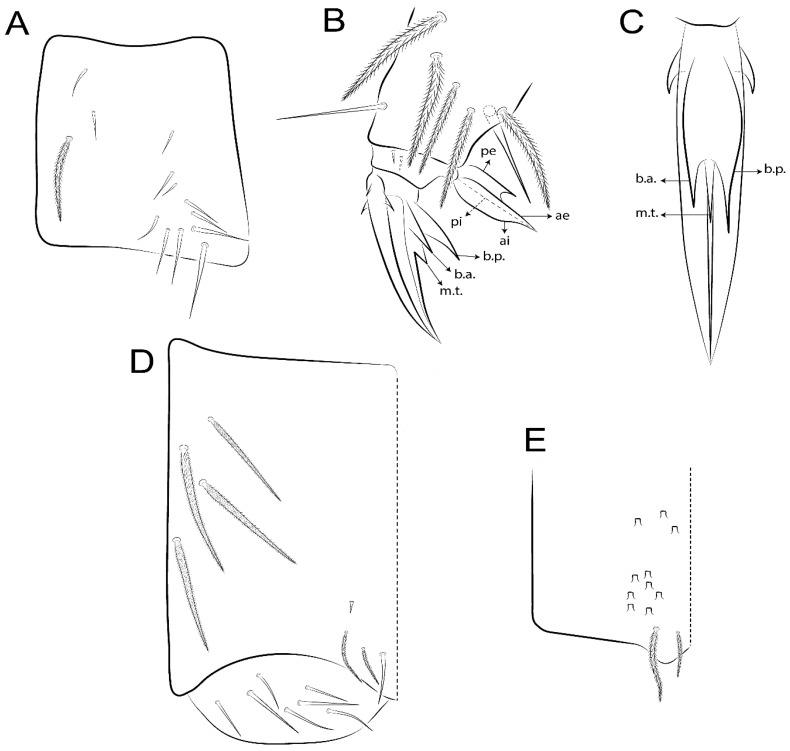
*Pseudosinella paraensis* sp. nov.: trunk appendages. (**A**) trochanteral organ; (**B**) distal tibiotarsus and empodial complex III (anterior view); (**C**) unguis (inner view); (**D**) collophore (lateral view); (**E**) distal part of manubrium (ventral view).

Figure 25, Figure 26 and Figure 27, Table 1 and Table 3

*Type material.* Holotype female in slide (2919/CRFS-UEPB): Brazil, Pará State, Canaã dos Carajás municipality, Carajás National Forest, 06°03′37.1″ S, 50°10′12.6″ W, 707 m., 7-12.x.2008, Andrade coll. Paratypes in slides (2970, 2973/CRFS-UEPB): 2 females, same data as holotype, except 28.ix–03.x.2007. Paratype in slide (2920/CRFS-UEPB donated to INPA/081): 1 female, same data as holotype.

*Description.* Total length (head + trunk) of holotype 1.26 mm.

Head. Ratio antennae:trunk = 1:2.09 in holotype; Ant III gently smaller than Ant II length; Ant segment ratio as I:II:III:IV = 1:2.24:2.24:3.85 in holotype. Ant IV dorsally with at least 2 sens d and 2 e, and numerous sens (b–c) and chaetae (i–k), sens a and h apparently absent. Ant III with 2 slim apical sens finger-shaped surrounded by at least 4 sens c and e, and 2 g (Figure 25A); ventrally with at least 4 b and c, and 3 e. Ant II dorsally with 1 apical sens c; ventrally with at least 6 sens c, 7 b, and 2 e. Clypeal formula with 4 (l1–2), 6 (ft), 5 (pf0–2) ciliate chaetae, l1–2 and pf1 acuminate, l1–2 larger, others subequal (Figure 25B). Prelabral chaetae smooth and not bifurcate. Labral chaetae smooth, no modifications (Figure 25B). Labial papilla D with 3 appendages; papilla E with l.p. finger-shaped, curved, and exceed the base of apical appendage (Figure 25C). Maxillary palp with b.c. smooth and 1.68 longer than the t.a. Head dorsal chaetotaxy (Figure 25D) with 9 “An” (An1a–3), 6 “A” (A0, A2a–3, and A5), 4 “M” (M1–4), 6 “S” (S0 and S2–6), 3 “Ps” (Ps2–3 and Ps5), 4 “Pa” (Pa2–5), 2 “Pm” (Pm1 and Pm3), 7 “Pp” (Pp1–7), and 2 “Pe” (Pe3 and 6) chaetae; An1a–3a, A0–3, and M2 as mac; interocular p mic present; head posterior region with 7 cervical like-spine mac. Basomedian and basolateral labial fields with a1–5 smooth; M1–2, E, and L1–2 clearly multiciliate; and r reduced. Labial proximal chaetae smooth and subequal (lpc1, 3, 4, and 6–7), except lpc6 gently smaller (Figure 25E). Ventral chaetotaxy with about 22 ciliate chaetae, 1 reduced lateral spine and 1 chaeta weakly ciliate, postlabial formula 4 (G1–4), 2 (X and X4), 4 (H1–4), and 2 (J1–2) ciliate chaetae, b.c. present (Figure 25E).

Thorax dorsal chaetotaxy (Figure 26A). Th II a, m, and p series with 1 (a5), 4 (m–5e?), and 6 (p1–6) mic, respectively. Th III a, m, and p series with 5 (a1–4 and a6), 4 (m2 and m4–6), and 6 (p1–6) mic, respectively. Ratio Th II:III = 2.31:1 in holotype.

Abdomen dorsal chaetotaxy (Figure 26B,C). Abd I a, m, and p series with 4 (a1–3 and a6), 5 (m2–6), and 2 (p5–6) mic, respectively. Abd II a, m, and p series with 3 (a2–3 and a6), 4 (m3–3e and m4–5), and 4 (p4–6 and p5p) chaetae, respectively, m3 and m5 as mac; a5 and m2 bothriotricha with 2–3 (lm, ll, plus 1 unnamed) and 2 (ml and mi) fan-shaped chaetae, respectively. Abd III a, m, and p series with 4 (a2–3 and a6–7), 6 (m3–4, am6, pm6, and m7–7e), and 5 (p3 and p5–8) chaetae, respectively, pm6 and p6 as mac, as sens not elongated, ms absent; m2 bothriotricha associated with 3 (a2, mi, and mi) and a5 and m5 bothriotricha with 5 (am6, li, lm, ll, and em) fan-shaped chaetae. Abd IV A–Fe series with 5 (A2–6), 4 (B2 and B4–6), 2 (Be3 and 1 unnamed), 5 (C1–4), 5 (T1, T3, and T5–7), 1 (Te7), 5 (D1–3p), 2 (De1 and De3), 5 (E1–4p), 3 (F1–3), and 4 (Fe1–3 and Fe5) chaetae, respectively, with 2 central (B5 and C1) and 11 lateral mac (T7, D3, De3, E2–4, F1, F3, Fe5, plus 2 unnamed); T2 and T4 bothriotricha with 5 (D1, a, s, m, plus 1 unnamed) and 2 (pe and pi) fan-shaped chaetae, respectively; 4 sens present, ps type I, as and 2 of uncertain homology type II, r series laterally with 3 chaetae. Abd V a, m, and p series with 0, 3 (m2–3 and m5), and 3 (p4 and 6–6p) mac, respectively. Ratio Abd III:IV = 1:4.63 in holotype. Th II–Abd IV formula with 00|010 + 21 + 1 mac.

Legs. Subcoxa I with 3 chaetae and 2 psp; subcoxa II with “a” row of 6–7 chaetae, “p” row with 6 chaetae and 2 psp; subcoxa III with one row of 7 chaetae and 2 posterior psp. Trochanteral organ with about 12 spine-like chaetae (Figure 27A). Tibiotarsus I–III formula with 0, 0, and 1 (outer) mac type VI (as Figure 45E). Unguis outer side with paired teeth developed in hook-shaped on proximal one fifth; inner side with slender lamella distally and 3 teeth, basal pair unequal, b.p. tooth surpass the m.t. apex, m.t. smaller than b.a. tooth and on unguis half, near to basal teeth, a.t. absent. Unguiculus with all lamellae smooth and acuminate (ai, ae, pi, and pe), except ai gently excavate distally and pe with outer tooth (Figure 27B,C); ratio unguis:unguiculus in holotype = 1:0.45. Tibiotarsal smooth chaeta about 0.93 smaller than unguiculus; tenent hair acuminate and about 0.57 smaller than unguis outer edge.

Collophore (Figure 27D). Anterior side with 4 ciliate chaetae apically acuminate, 1 proximal slim, others subequal; posterior side distally with 1 smooth chaeta, 2 ciliate chaetae, and 1 subdistal reduced spine; lateral flap with 6 smooth chaetae, 2 larger.

Furcula. Only with ciliate chaetae and scales. Manubrium ventrally with 3 subapical and about 7 distal scales (Figure 27E); manubrial plate with 4 ciliate chaetae (2 inner mac) and 2 psp (as Figure 11C). Mucro basal tooth gently larger than distal tooth, basal spine reaches the apex of basal tooth (as Figure 11D).

*Etymology.* Refers to type locality of the new species, “Pará” State.

*Remarks. Pseudosinella paraensis* sp. nov. resembles *P. sera* Christiansen and Bellinger, 1980 by Th II–Abd IV with 0, 0, 0, 1, 0, and 2 central mac, and unguis with 3 inner tooth, with b.p. larger than b.a (see Table 1, Table 2 and Table 3). However, *P. paraensis* sp. nov. differs from this species by head A2a and M2 mac (absent in *P. sera*), Abd IV with C1 mac but without B6 mac (the opposite in *P. sera*), unguis m.t. near to basal teeth (distant in *P. sera*), unguiculus ai lamella gently excavate distally (acuminate in *P. sera*), tenent hair acuminate (capitate in *P. sera*), and manubrial plate with 4 chaetae (6–7 in *P. sera*) [5,7,20]. The unguis morphology of *P. paraensis* sp. nov. is similar to *P. goughi* Gisin and Gama, 1972, but the new species differs by unguis m.t. present and unguiculus pe lamellae with outer tooth, both features absent in *P. goughi* [50].

#### 3.2.9. *Pseudosinella serpentinensis* sp. nov. Cipola

**Figure 28:**
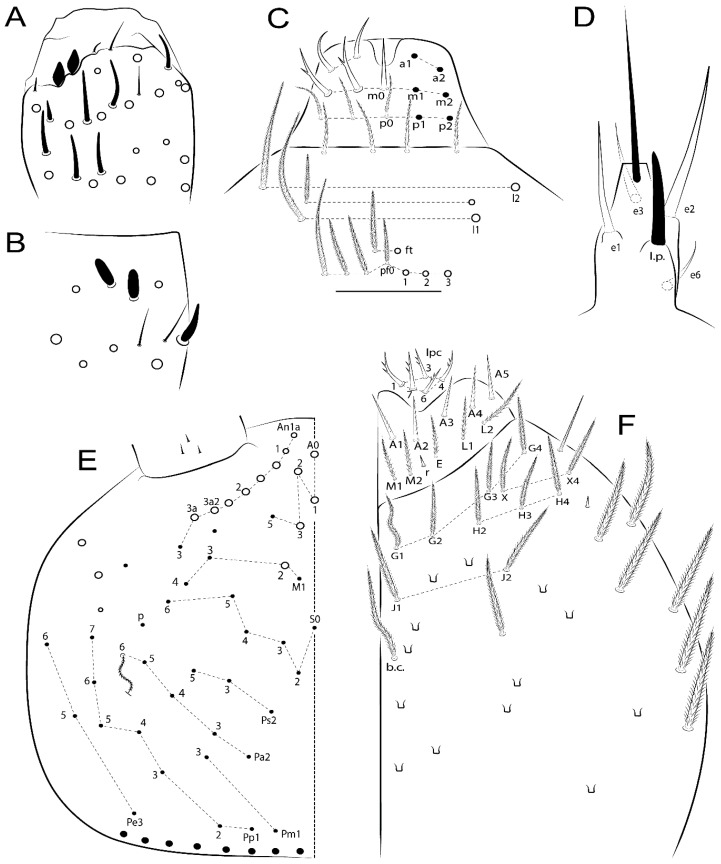
*Pseudosinella serpentinensis* sp. nov.: head. (**A**) Ant III apical organ (lateral view); (**B**) Ant II distal part (dorsal view); (**C**) chaetotaxy of clypeus, prelabrum, and labrum; (**D**) labial papilla E (right side); (**E**) head dorsal chaetotaxy (left side); (**F**) labial proximal chaetae, basomedian and basolateral labial fields, and complete postlabial chaetotaxy (right side).

**Figure 29:**
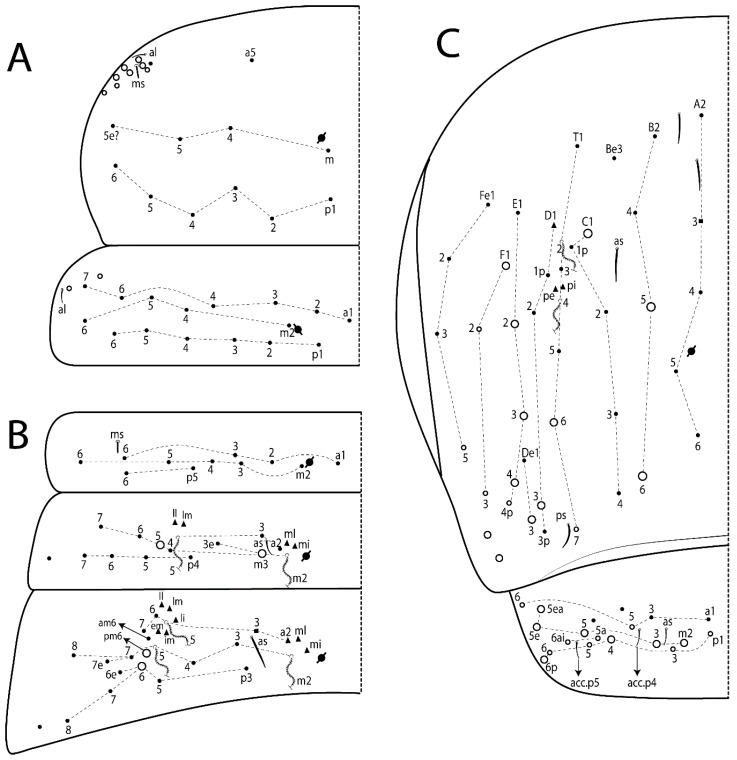
*Pseudosinella serpentinensis* sp. nov.: dorsal chaetotaxy. (**A**) Th II–III; (**B**) Abd I–III; (**C**) Abd IV–V.

**Figure 30:**
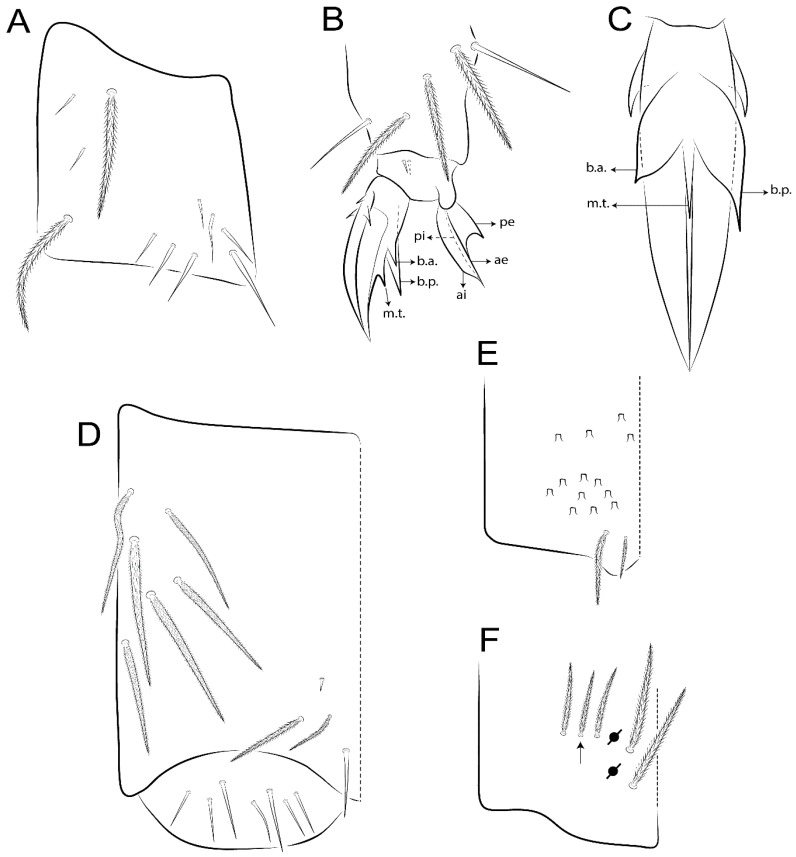
*Pseudosinella serpentinensis* sp. nov.: trunk appendages. (**A**) trochanteral organ; (**B**) distal tibiotarsus and empodial complex III (anterior view); (**C**) unguis (inner view); (**D**) collophore (lateral view); (**E**) distal part of manubrium (ventral view); (**F**) manubrial plate (dorsal view).

Figure 28, Figure 29 and Figure 30, Table 1 and Table 3

*Type material.* Holotype female in slide (11190/CRFS-UEPB): Brazil, Minas Gerais State, Morro do Pilar municipality, South of “Serra da Serpentina”, city west region, 19°13′15.4″ S, 43°23′23.3″ W, 810 m, 06–10.v.2017, Carste et al. coll. Paratypes in slides (11191–92/CRFS-UEPB): 1 male and 1 female, same data as holotype. Paratypes in slides (10994, 11194/CRFS-UEPB donated to INPA/082): 1 male and 1 female, same data as holotype. Paratypes in slides (11102, 11105, 11143-45/CRFS-UEPB): 1 male, 3 females and 1 juvenile, same data as holotype, except 10-15.i.2017. Paratype in slide (11101/CRFS-UEPB donated to INPA/082): 1 female, same data as holotype, except 10-15.i.2017. Paratype in slide (11146/CRFS-UEPB): 1 juvenile, same data as holotype, except 16-26.i.2017. Paratypes in slides (11613-14/CRFS-UEPB): 2 females, same data as holotype, except 12-14.vi.2017. Paratypes in slides (4790, 4798/CRFS-UEPB): 2 females, same data as holotype, except 19–20.xi.2013, LGS Soares et al. and FO Borges coll, respectively. Paratype in slide (11302/CRFS-UEPB): 1 female, same data as holotype, except 08-10.vi.2015.

*Description.* Total length (head + trunk) of holotype 1.11 mm.

Head. Ratio antennae:trunk = 1:2.49 in holotype; Ant III subequal or gently smaller than Ant II length; Ant segment ratio as I:II:III:IV = 1:1.29:1.14:2.09 in holotype. Ant IV dorsally with 1 sens a and 1 h, 2 d and 2 e, and numerous sens (b–c) and chaetae (i–k). Ant III with 2 apical sens swollen lance-shape surrounded by at least 6 sens c, 2 b and g, and 1 e (Figure 28A); ventrally with at least 1 c and 3 e. Ant II dorsally with 2 apical sens f, 2 b and 1 e, all together (Figure 28B); ventrally with at least 3 sens c and b, and 1 e. Clypeal formula with 6 (l1, 1e–2), 2 (ft), and 7 (pf0–3) ciliate chaetae, l1–2 and pf3 larger and acuminate, pf0 gently smaller, others subequal (Figure 28C). Prelabral chaetae ciliate and not bifurcate. Labral a1–2 and m0–2 chaetae thicker, a1–2 and m0 smooth, m1–2 smooth or with 1 median filament, p0–2 chaetae ciliate (Figure 28C). Labial papilla D with 3 appendages; papilla E with l.p. conical, straight and gently exceed the base of apical appendage (Figure 28D). Maxillary palp with b.c. unilaterally and heavily ciliate, 1.27 longer than the t.a. (as Figure 19D). Head dorsal chaetotaxy (Figure 28E) with 9 “An” (An1a–3), 5 “A” (A0–3 and A5), 4 “M” (M1–4), 6 “S” (S0 and S2–6), 3 “Ps” (Ps2–3 and Ps5), 4 “Pa” (Pa2–5), 2 “Pm” (Pm1 and Pm3), 7 “Pp” (Pp1–7), and 3 “Pe” (Pe3 and Pe6–7) chaetae; An1a–3a, A0–3, and M2 as mac; interocular p mic present; head posterior region with 8 cervical like-spine mac. Basomedian and basolateral labial fields with A1–5 unilaterally and weakly ciliate; M1–2, E, and L1–2 ciliate; and r reduced. Labial proximal chaetae with 2 filaments (lpc1, 3, 4, 6, and 7), lpc6 gently smaller, others subequal (Figure 28F). Ventral chaetotaxy with about 19 ciliate chaetae, 1 reduced lateral spine and 1 chaeta weakly ciliate, postlabial formula 4 (G1–4), 2 (X and X4), 3 (H2–4), and 2 (J1–2) ciliate chaetae, 6 lateral chaetae thicker and b.c. present (Figure 28F).

Thorax dorsal chaetotaxy (Figure 29A). Th II a, m, and p series with 1 (a5), 4 (m–5e?), and 6 (p1–6) mic, respectively. Th III a, m, and p series with 6 (a1–4 and a6–7), 4 (m2 and m4–6), and 6 (p1–6) mic, respectively. Ratio Th II:III = 1.96:1 in holotype.

Abdomen dorsal chaetotaxy (Figure 29B,C). Abd I a, m, and p series with 4 (a1–3 and a6), 5 (m2–6), and 2 (p5–6) mic, respectively. Abd II a, m, and p series with 2 (a2–3), 6 (m3–3e and m4–7), and 4 (p4–7 and p5p absent) chaetae, respectively, m3 and m5 as mac; a5 and m2 bothriotricha with 2 (lm and ll) and 2 (mi and ml) fan-shaped chaetae, respectively. Abd III a, m, and p series with 4 (a2–3 and a6–7), 7 (m3–4, am6, pm6, m7–7e, and m8), and 6 (p3, p5–8, and p6e?) chaetae, respectively, pm6 and p6 as mac, a3 as reduced mic, as sens elongated, ms absent; m2 bothriotricha associated with 3 (a2, mi, and mi) and a5 and m5 bothriotricha with 5 (li, lm, ll, im, and em) fan-shaped chaetae. Abd IV A–Fe series with 5 (A2–6), 4 (B2 and B4–6), 1 (Be3), 5 (C1–4), 5 (T1, T3, and T5–7), 5 (D1–3p), 2 (De1 and De3), 5 (E1–4p), 3 (F1–3), and 4 (Fe1–3 and Fe5) chaetae, respectively, with 3 central (B5–6 and C1) and 9 lateral mac (T6, D3, De3, E2–4, F1, and 2 unnamed); T2 and T4 bothriotricha with 1 (D1) and 2 (pe and pi) fan-shaped chaetae, respectively; 4 sens type II present, ps, as and 2 of uncertain homology, r series unclear to see. Abd V a, m, and p series with 0, 5 (m2–3 and m5–5ea), and 2 (p4 and p6p) mac, respectively. Ratio Abd III:IV = 1:2.85 in holotype. Th II–Abd IV formula with 00|010 + 21 + 2 mac.

Legs. Subcoxa I with 4–5 chaetae and 2 psp; subcoxa II with “a” row of 6–7 chaetae, “p” row with 4–5 chaetae and 2 psp; subcoxa III with one row of 7–9 chaetae and 2 posterior psp. Trochanteral organ with about 9 spine-like chaetae (Figure 30A). Tibiotarsus I–III formula with 1, 3, and 3 mac type II (as Figure 10E–G). Unguis outer side with paired teeth developed in hook-shaped on proximal one fourth; inner side with wide lamella and 3 teeth, basal pair unequal, b.p. tooth surpass the m.t. apex, m.t. on a little more than the unguis distal half and subequal to b.a. tooth length, a.t. absent. Unguiculus with all lamellae smooth and acuminate (ai, ae, pi, and pe), except ai gently excavate distally and pe with outer tooth (Figure 30B,C); ratio unguis:unguiculus in holotype = 1:0.42. Tibiotarsal smooth chaeta about 0.81 smaller than unguiculus; tenent hair acuminate and about 0.37 smaller than unguis outer edge.

Collophore (Figure 30D). Anterior side with 6 ciliate chaetae apically acuminate, 3 proximal subequal in length, 2 median larger and 1 distal gently smaller; posterior side distally with 1 smooth chaeta, 2 ciliate chaetae (outer chaetae larger), and 1 subdistal reduced spine; lateral flap with 7 smooth chaetae, 2 larger.

Furcula. Only with ciliate chaetae and scales. Manubrium ventrally with 4 subapical and about 9 distal scales; manubrial plate with 4–5 ciliate chaetae (2 inner mac) and 2 psp (Figure 30E,F). Mucro teeth subequal in size, basal spine reaches the apex of basal tooth (as Figure 15K).

*Etymology.* Refers to “Serpentina” in the name of the type locality of the new species.

*Remarks. Pseudosinella serpentinensis* sp. nov. resembles *P. acantholabrata* sp. nov., but *P. serpentinensis* sp. nov. differs from this species by labial papilla E with l.p. conical and straight, maxillary palp with b.c. unilaterally ciliate, postlabial X chaeta present, labral a2 and m0 chaetae smooth and m1–2 chaetae smooth or with 1 filament. They also differ by tenent hair acuminate and unguiculus with ai lamellae gently excavate in *P. serpentinensis* sp. nov. See also the comparison these species in remarks of *P. acantholabrata* sp. nov. and Table 1 and Table 3.

#### 3.2.10. *Pseudosinella taurina* sp. nov. Cipola

**Figure 31:**
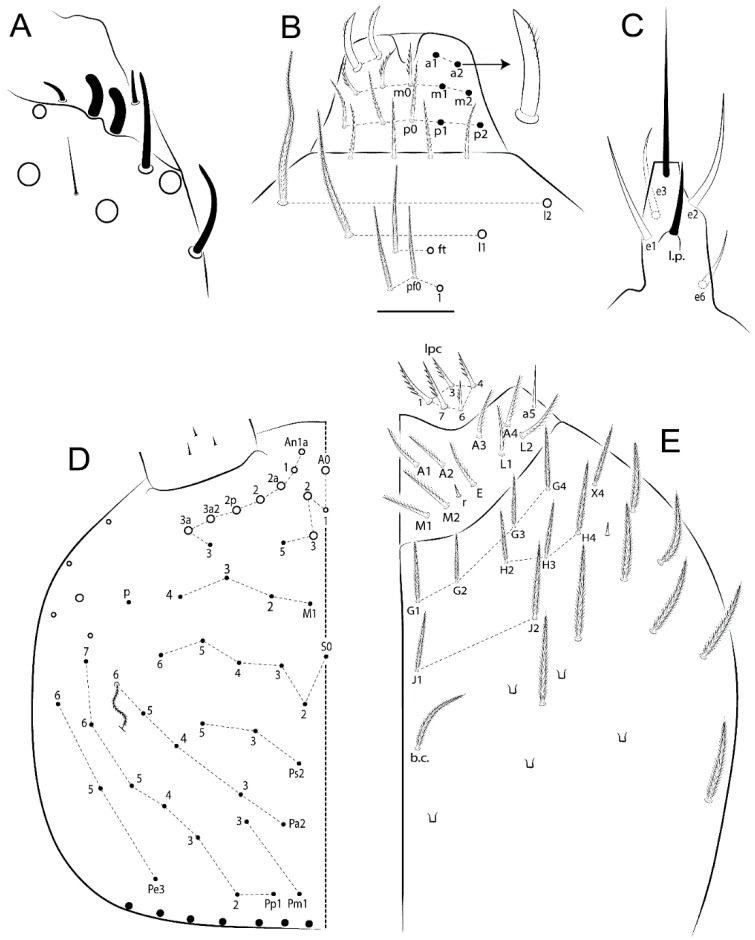
*Pseudosinella taurina* sp. nov.: head. (**A**) Ant III apical organ (lateral view); (**B**) chaetotaxy of clypeus, prelabrum, and labrum; (**C**) labial papilla E (right side); (**D**) head dorsal chaetotaxy (left side); (**E**) labial proximal chaetae, basomedian and basolateral labial fields, and complete postlabial chaetotaxy (right side).

**Figure 32:**
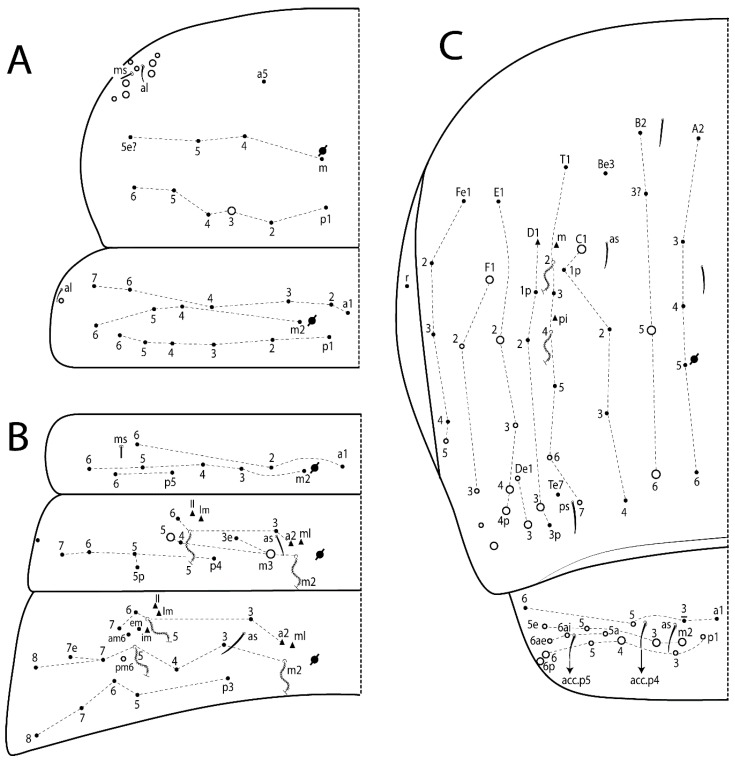
*Pseudosinella taurina* sp. nov.: dorsal chaetotaxy. (**A**) Th II–III; (**B**) Abd I–III; (**C**) Abd IV–V.

**Figure 33:**
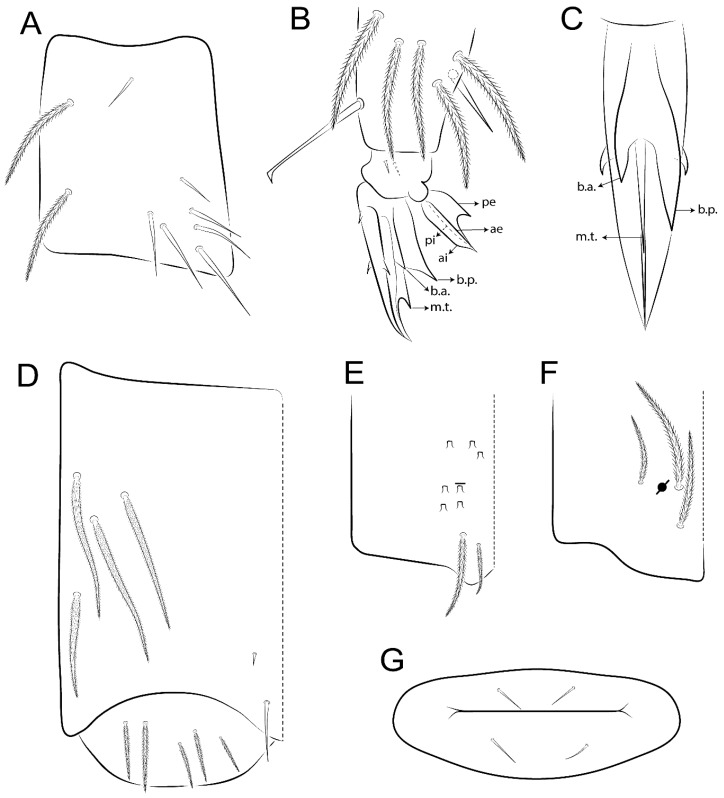
*Pseudosinella taurina* sp. nov.: trunk appendages. (**A**) trochanteral organ; (**B**) distal tibiotarsus and empodial complex III (anterior view); (**C**) unguis (inner view); (**D**) collophore (lateral view); (**E**) distal part of manubrium (ventral view); (**F**) manubrial plate (dorsal view); (**G**) female genital plate (ventral view).

Figure 31, Figure 32 and Figure 33, Table 1 and Table 3

*Type material.* Holotype female in slide (8254/CRFS-UEPB): Brazil, Pará State, Curionópolis municipality, near mining of “Serra Pelada”, 10km to city north, 05°59′57.8″ S, 49°36′57.1″ W, 447 m, 01-19.iii.2016, Spelayon coll. Paratypes in slides (7880, 7998, 8251-52, 8160/CRFS-UEPB): 4 females and 1 juvenile, same data as holotype. Paratypes in slides (8382–83/CRFS-UEPB donated to INPA/083): 2 females, same data as holotype. Paratype in slide (8491/CRFS-UEPB): 1 juvenile, same data as holotype, except 02-15.iii.2016.

*Description.* Total length (head + trunk) of holotype 0.78 mm.

Head. Ratio antennae:trunk = 1:2.33 in holotype; Ant III gently smaller than Ant II length; Ant segment ratio as I:II:III:IV = 1:1.77:1.46:3.09 in holotype. Ant IV dorsally with 1 sens a and at least 1 e, and numerous sens (b–c) and chaetae (i–k), sens d and h apparently absent. Ant III with 2 apical sens finger-shaped surrounded by at least 3 sens c, 2 g, and 1 b (Figure 31A); ventrally with at least 2 sens c and 1 b. Ant II dorsally with at least 2 sens c; ventrally with at least 3 sens c, 2 e, and 1 b. Clypeal formula with 4 (l1–2), 2 (ft), and 3 (pf0–1) ciliate chaetae, all acuminate, l1–2 larger, others subequal (Figure 31B). Prelabral chaetae ciliate and not bifurcate. Labral a1–2 chaetae thicker as horn, both smooth or with 3–5 small distal filaments (similar to ciliations), m0–2 not thicker, smooth or with 2–5 median filament, p0–2 chaetae ciliate (Figure 31B). Labial papilla D with 3 appendages; papilla E with l.p. acuminate, straight and reaches the base of apical appendage (Figure 31C). Maxillary palp with b.c. weakly ciliate and 1.71 longer than the t.a. (as Figure 8D) Head dorsal chaetotaxy (Figure 31D) with 8 “An” (An1a–3), 5 “A” (A0–3 and A5), 4 “M” (M1–4), 6 “S” (S0 and S2–6), 3 “Ps” (Ps2–3 and Ps5), 4 “Pa” (Pa2–5), 2 “Pm” (Pm1 and Pm3), 7 “Pp” (Pp1–7), and 3 “Pe” (Pe3 and Pe5–6) chaetae; An1a–3a, A0, and A2–3 as mac; interocular p mic present; head posterior region with 7 cervical like-spine mac. Basomedian and basolateral labial fields with a5 smooth and smaller; A1–4, M1–2, E, and L1–2 unilaterally ciliate; and r reduced. Labial proximal chaetae with 3–5 filaments (lpc1, 3, 4, and 6–7), 1 smaller and 3 filaments (lpc6), 2 gently smaller (lpc1 and lpc7), others subequal (Figure 31E). Ventral chaetotaxy with about 19 ciliate chaetae and 1 reduced lateral spine, postlabial formula 4 (G1–4), 1 (X4), 3 (H2–4), and 2 (J1–2) ciliate chaetae, about 6 lateral chaetae thicker, b.c. present (Figure 31E).

Thorax dorsal chaetotaxy (Figure 32A). Th II a, m, and p series with 1 (a5), 4 (m–5e?), and 5 (p1–2 and p4–6) mic, respectively, p3 as mac. Th III a, m, and p series with 6 (a1–4 and a6–7), 4 (m2 and m4–6), and 6 (p1–6) mic, respectively. Ratio Th II:III = 1.61:1 in holotype.

Abdomen dorsal chaetotaxy (Figure 32B,C). Abd I a, m, and p series with 3 (a1–2 and a6), 5 (m2–6), and 2 (p5–6) mic, respectively, a3 absent. Abd II a, m, and p series with 3 (a2–3 and a6), 4 (m3–3e and m4–5), and 5 (p4–7 and p5p) chaetae, respectively, m3 and m5 as mac; a5 and m2 bothriotricha with 2 (lm and ll) and 2 (a2 and ml) fan-shaped chaetae (mi absent), respectively. Abd III a, m, and p series with 4 (a2–3 and a6–7), 7 (m3–4, am6, pm6, m7–7e, and m8), and 5 (p3 and p5–8) chaetae, respectively, pm6 as mes, as sens elongated, ms absent; m2 bothriotricha associated with 2 (a2 and ml) and a5 bothriotricha with 3 (lm, ll, and im) fan-shaped chaetae, others as mic. Abd IV A–Fe series with 5 (A2–6), 4 (B2–3? and B5–6), 1 (Be3), 5 (C1–4), 5 (T1, T3, and T5–7), 1 (Te7), 5 (D1–3p), 2 (De1 and De3), 5 (E1–4p), 3 (F1–3), and 5 (Fe1–5) chaetae, respectively, with 3 central (B5–6 and C1) and 6 lateral mac (D3, De3, E2, E4–4p, F1, plus 1 unnamed); T2 and T4 bothriotricha with 2 (D1 and m) and 1 (pi) fan-shaped chaetae, respectively; 4 sens type II present, ps, as and 2 of uncertain homology, r series laterally with 1 mic. Abd V a, m, and p series with 0, 2 (m2–3), and 3 (p4 and 6–6p) mac, respectively. Ratio Abd III:IV = 1:3.21 in holotype. Th II–Abd IV formula with 10|010 + 01 + 2 mac.

Legs. Subcoxa I with 2 chaetae and 2 psp; subcoxa II with “a” row of 3 chaetae, “p” row with 7 chaetae and 2 psp; subcoxa III with one row of 7 chaetae and 2 posterior psp. Trochanteral organ with about 7 spine-like chaetae (Figure 33A). Tibiotarsus I–III formula with 1, 3, and 3 mac type II (as Figure 10E–G). Unguis outer side with paired teeth developed in hook-shaped on unguis half; inner side with slender lamella and 3 teeth, basal pair unequal, b.p. tooth larger, but not reaching the m.t. apex, m.t. on distal one thirds and subequal to b.a. tooth length, a.t. absent. Unguiculus with all lamellae smooth and acuminate (ai, ae, pi, and pe), ai gently excavate distally and pe with outer tooth (Figure 33B,C); ratio unguis:unguiculus in holotype = 1:0.42. Tibiotarsal smooth chaeta about 1.05 longer than unguiculus; tenent hair capitate and about 0.78 smaller than unguis outer edge.

Collophore (Figure 33D). Anterior side with 4 ciliate chaetae apically acuminate, 1 distal gently smaller; posterior side distally with 1 smooth chaeta and 1 subdistal reduced spine; lateral flap with 2 ciliate chaetae, 2 larger.

Furcula. Only with ciliate chaetae and scales. Manubrium ventrally with 3 subapical and about 3–4 distal scales; manubrial plate with 3 ciliate chaetae (2 inner mac) and 1 psp (Figure 33E,F). Mucro teeth subequal in size, basal spine reaches the apex of basal tooth (as Figure 15K).

*Etymology.* Refers to labral a1–2 chaetae of the new species which resemble the horns of a bull or an ox (from Latim: taurus) (Figure 31B).

*Remarks. Pseudosinella taurina* sp. nov. resembles most *P. certa* Christiansen and Bellinger, 1980 and *P. unimacrochaetosa* sp. nov. by head “M” and “S” series devoid of mac, Th II–Abd III with 1, 0, 0, 1, and 0 central mac, and unguis basal teeth unequal (see Table 1, Table 2 and Table 3). However, both new species differ from *P. certa* by basomedian and basolateral labial fields with A1–4 unilaterally or completely ciliate in *P. taurina* sp. nov and *P. unimacrochaetosa* sp. nov., respectively (smooth in *P. certa*). They also differ by Abd IV with 3 central mac (2 in *P. certa*), tenent hair capitate (acuminate in *P. certa*), unguis with m.t. (absent in *P. certa*), and mucro teeth subequal and with basal spine reaching the apex of basal tooth, while *P. certa* has basal tooth smaller and basal spine exceding the apex of it [5,7,20]. Both new species also resemble *P. josemarii* Soto-Adames, 2010 by the presence of 1 mac on Th II, but they differ in Pa5 mac absent and Th III devoid of mac, while in *P. josemarii* this chaeta is present (see Table 2) [20]. Finally, *P. taurina* sp. nov. differs from *P. unimacrochaetosa* sp. nov. by labral a1–2 thicker, similar to horns (no such morphology in *P. unimacrochaetosa* sp. nov.), labial papilla E with l.p. acuminate (finger-shaped in *P. unimacrochaetosa* sp. nov.), and collophore anteriorly with 4 chaetae and lateral flap with 5 ciliate chaetae (7 anterior and 6 smooth chaetae on lateral flap in *P. unimacrochaetosa* sp. nov.). They also differ by 3 unguis inner teeth, with b.p. undivided in *P. taurina* sp. nov. while in *P. unimacrochaetosa* sp. nov. there are 4 unguis inner teeth, with b.p. divided.

#### 3.2.11. *Pseudosinella unimacrochaetosa* sp. nov. Cipola

**Figure 34:**
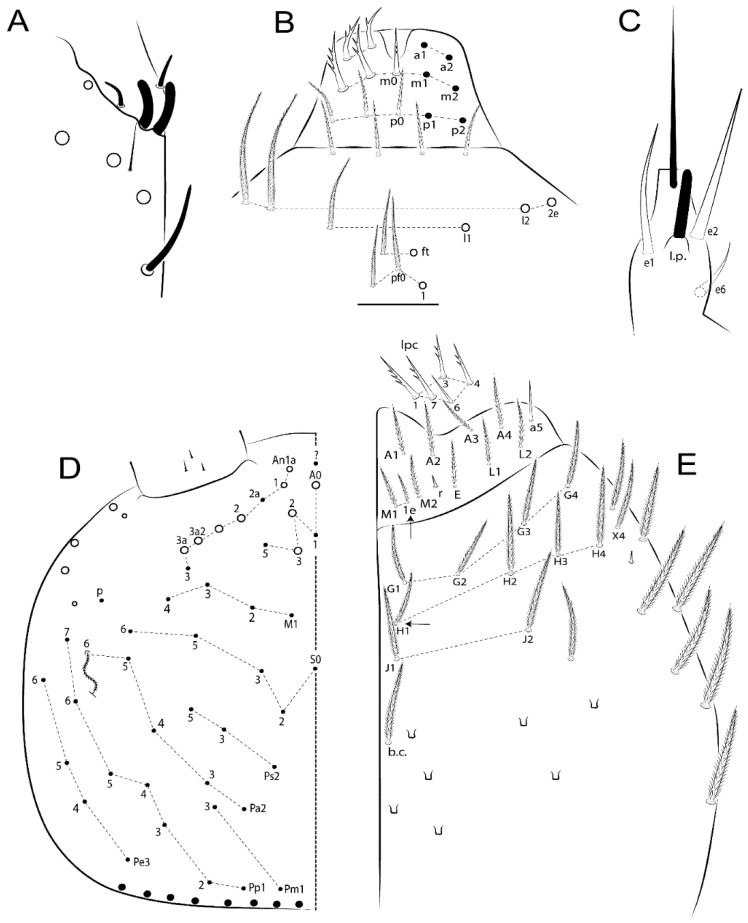
*Pseudosinella unimacrochaetosa* sp. nov.: head. (**A**) Ant III apical organ (lateral view); (**B**) chaetotaxy of clypeus, prelabrum, and labrum; (**C**) labial papilla E (right side); (**D**) head dorsal chaetotaxy (left side); (**E**) labial proximal chaetae, basomedian and basolateral labial fields, and complete postlabial chaetotaxy (right side), arrows indicate chaetae present or absent.

**Figure 35:**
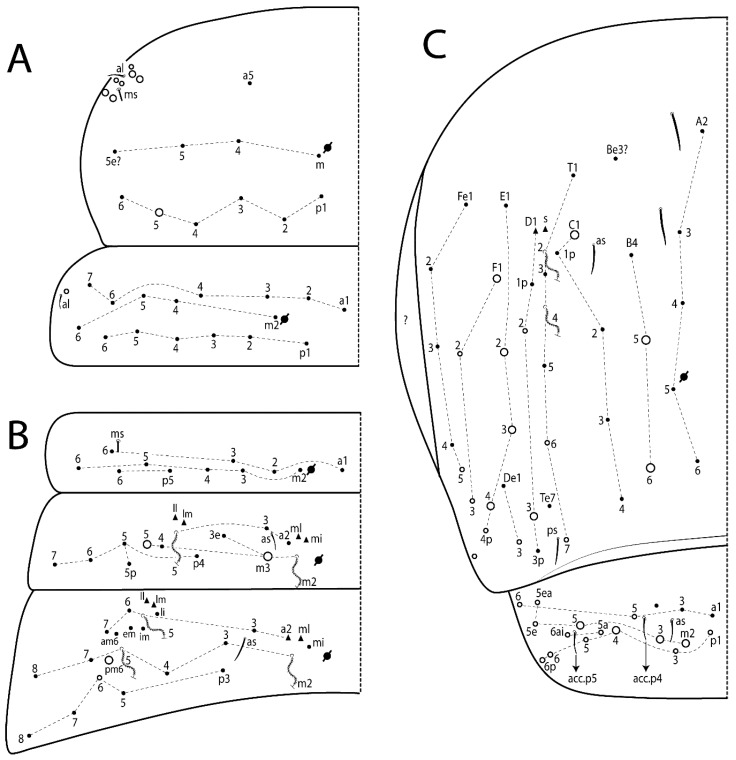
*Pseudosinella unimacrochaetosa* sp. nov.: dorsal chaetotaxy. (**A**) Th II–III; (**B**) Abd I–III; (**C**) Abd IV–V.

**Figure 36:**
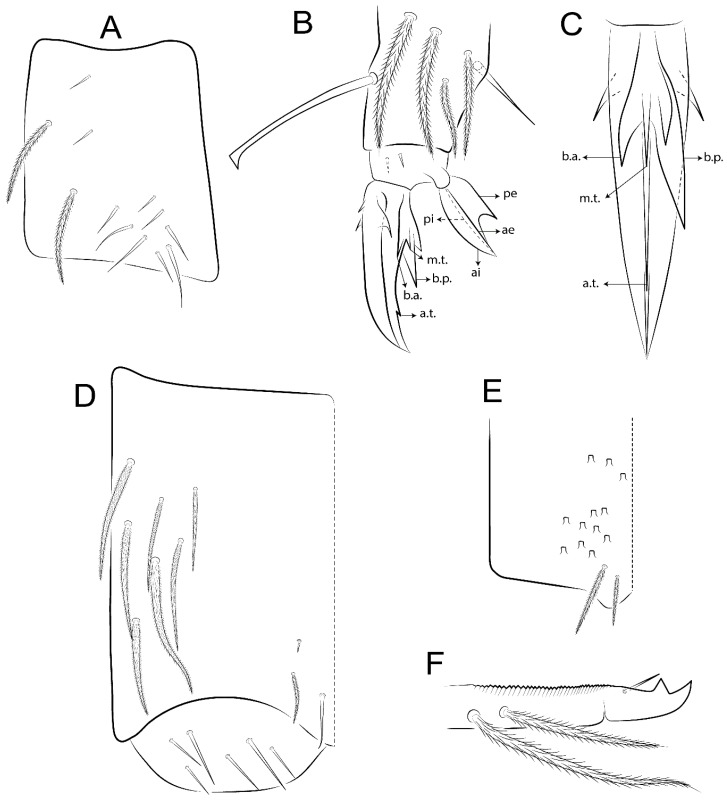
*Pseudosinella unimacrochaetosa* sp. nov.: trunk appendages. (**A**) trochanteral organ; (**B**) distal tibiotarsus and empodial complex III (anterior view); (**C**) unguis (inner view); (**D**) collophore (lateral view); (**E**) distal part of manubrium (ventral view); **F**) distal dens and mucro (outer view).

Figure 34, Figure 35 and Figure 36, Table 1 and Table 3

*Type material.* Holotype male in slide (10093/CRFS-UEPB): Brazil, Minas Gerais State, Rio Acima municipality, Serra do Gandarela National Park, “GAND-114” cavity, 20°04′04.5″ S, 43°40′11.9″ W, 1278 m, 14.vii-18.ix.2016, Carste et al. coll. Paratypes in slides (10094, 10121, 10155/CRFS-UEPB): 1 male and 2 juveniles, same data as holotype. Paratype in slide (9961/CRFS-UEPB): 1 male, same data as holotype, except 10.ii-20.iii.2014. Paratypes in slides (10046, 10048, 10096, 10158, 10221, 10305, 10337, 10542, 10548/CRFS-UEPB): 2 males and 7 juveniles, same data as holotype, except in other nearby cavities. Paratypes in slides (6083–84, 6099, 6100, 6167-68, 6170, 6172, 6301-02, 6305, 6308, 6311, 10215–16/CRFS-UEPB): 5 males, 6 females and 4 juveniles, same data as holotype, except in other nearby cavities and 10.ii-20.iii.2014. Paratypes in slides (10213–14/CRFS-UEPB): 2 juveniles, same data as holotype, except in other nearby cavities and 15-31.iii.2016. Paratypes in slides (6088–91/CRFS-UEPB donated to INPA/084): 2 males and 2 females, same data as holotype, except in other nearby cavities and 10.ii-20.iii.2014.

*Description.* Total length (head + trunk) of holotype 0.87 mm.

Head. Ratio antennae:trunk = 1:2.23 in holotype; Ant III subequal to Ant II length; Ant segment ratio as I:II:III:IV = 1:1.75:1.68:3.18 in holotype. Ant IV dorsally with 1 sens a, 1 e and 1 h, 3 d, and numerous sens (b–c) and chaetae (i–k). Ant III with 2 apical sens finger-shape surrounded by 1 sens b and 1 c, and 2 g (Figure 34A); ventrally with at least 2 sens c, 1 b, and 1 e. Ant II dorsally with 1 sens c; ventrally with at least 3 sens c, 1 b, and 1 e. Clypeal formula with 6 (l1, l2–2e), 2 (ft), and 3 (pf0–1) ciliate chaetae, all acuminate, l1–2e larger, and other subequal (Figure 34B). Prelabral chaetae ciliate and not bifurcate. Labral a1–2 and m0–2 chaetae thicker, a1–2 smooth or with 1 and 1–2 median filaments, respectively, m0–1 with 1–2 median filament, m2 with 2–4 median filaments, p0–2 chaetae ciliate (Figure 34B). Labial papilla D with 3 appendages; papilla E with l.p. finger-shaped, direct and reaches the base of apical appendage, e3 appendage absent or no see (Figure 34C). Maxillary palp with b.c. unilaterally and heavily ciliate, 1.51 longer than the t.a. (as Figure 19D). Head dorsal chaetotaxy (Figure 34D) with 8 “An” (An1a–3), 5 “A” (A0–3 and A5), 4 “M” (M1–4), 5 “S” (S0, S2–3, and S5–6), 3 “Ps” (Ps2–3 and Ps5), 4 “Pa” (Pa2–5), 2 “Pm” (Pm1 and Pm3), 7 “Pp” (Pp1–7), and 4 “Pe” (Pe3–6) chaetae; An1a–1, An2–3a, A0, and A2–3 as mac, S4 absent; interocular p mic present; head posterior region with 8 cervical like-spine mac. Basomedian and basolateral labial fields with a5 smooth and gently smaller; A1–4, M1–1e, M2, E, and L1–2 ciliate; r reduced; M1e gently smaller and rarely present. Labial proximal chaetae with 2–3 median filaments (lpc1, 3, 4, and 6–7), lpc6 smooth and gently smaller, others subequal (Figure 34E). Ventral chaetotaxy with about 19–20 ciliate chaetae and 1 reduced lateral spine, postlabial formula 4 (G1–4), 1 (X4), 3–4 (H1–4), and 2 (J1–2) ciliate chaetae, about 6 lateral chaetae thicker, b.c. present (Figure 34E).

Thorax dorsal chaetotaxy (Figure 35A). Th II a, m, and p series with 1 (a5), 4 (m–5e?), and 5 (p1–4 and p6) mic, respectively, p5 as mac. Th III a, m, and p series with 6 (a1–4 and a6–7), 4 (m2 and m4–6), and 6 (p1–6) mic, respectively. Ratio Th II:III = 1.58:1 in holotype.

Abdomen dorsal chaetotaxy (Figure 35B,C). Abd I a, m, and p series with 4 (a1–3 and a6), 5 (m2–6), and 2 (p5–6) mic, respectively. Abd II a, m, and p series with 2 (a2–3), 4 (m3–3e and m4–5), and 5 (p4–6 and p5p) chaetae, respectively, m3 and m5 as mac; a5 and m2 bothriotricha with 2 (lm and ll) and 2 (ml and mi) fan-shaped chaetae, respectively. Abd III a, m, and p series with 4 (a2–3 and a6–7), 6 (m3–4, am6, pm6, and m7–8), and 5 (p3 and p5–8) chaetae, respectively, pm6 as mac and p6 as mes, as sens elongated, ms absent; m2 bothriotricha associated with 2 (mi and ml) and a5 bothriotricha with 2 (lm and ll) fan-shaped chaetae, others as mic. Abd IV A–Fe series with 5 (A2–6), 3 (B4–6), 1 (Be3?), 5 (C1–4), 5 (T1, T3, and T5–7), 1 (Te7), 5 (D1–3p), 2 (De1 and De3), 5 (E1–4p), 3 (F1–3), and 5 (Fe1–5) chaetae, respectively, with 3 central (B5–6 and C1) and 5 lateral mac (D3, E2–4, and F1); T2 and T4 bothriotricha with 2 (D1 and s) and 0 fan-shaped chaetae, respectively; 4 sens type II present, ps, as, and 2 of uncertain homology, r series unclear to see. Abd V a, m, and p series with 0, 3 (m2–3 and m5), and 1 (p4) mac, respectively. Ratio Abd III:IV = 1:3.62 in holotype. Th II–Abd IV formula with 10|010 + 11 + 2 mac.

Legs. Subcoxa I with 2–3 chaetae and 2 psp; subcoxa II with “a” row of 6–7 chaetae, “p” row with 3 chaetae and 2 psp; subcoxa III with one row of 7 chaetae and 2 posterior psp. Trochanteral organ with about 10 spine-like chaetae (Figure 36A). Tibiotarsus I–III formula with 1, 3, and 3 mac type II (as Figure 10E–G). Unguis outer side with paired teeth developed in hook-shaped on proximal one fourth; inner side with wide lamella and 4 teeth, basal pair unequal, b.p. tooth reaches to half unguis length, and with 1 smaller split tooth posteriorly, m.t. on proximal one third (between basal teeth) and smaller to b.a. tooth length, a.t. minute on distal one quarter. Unguiculus with all lamellae smooth and acuminate (ai, ae, pi, and pe), except pe with outer tooth (Figure 36B,C); ratio unguis:unguiculus in holotype = 1:0.52. Tibiotarsal smooth chaeta about 0.84 smaller than unguiculus; tenent hair capitate and about 0.97 smaller than unguis outer edge.

Collophore (Figure 36D). Anterior side with 7 ciliate chaetae apically acuminate, 4 mac subequal in length (except 1 distal smaller) and 3 proximal thin; posterior side distally with 1 smooth chaeta, 1 lateral ciliate chaetae, and 1 subdistal reduced spine; lateral flap with 6 smooth chaetae, 2 larger.

Furcula. Only with ciliate chaetae and scales. Manubrium ventrally with 3 subapical and about 9 distal scales (Figure 36E); manubrial plate with 4–5 ciliate chaetae (2 inner mac) and 2 psp (as Figure 11C and Figure 15F). Mucro distal tooth gently larger than basal tooth, basal spine reaches the apex of basal tooth (Figure 36F).

*Etymology.* Refers to the presence of a single macrochaeta on Th II of the new species (from Latim: uni-one) (Figure 35A).

*Remarks. Pseudosinella unimacrochaetosa* sp. nov. resembles *P. certa* and *P. taurina* sp. nov. (see Table 1, Table 2 and Table 3). However, *P. unimacrochaetosa* sp. nov. differ from *P. taurina* sp. nov. by labral a1–2 chaetae with filaments (no horns), labial papilla E with l.p. finger-shaped, collophore anteriorly with with 7 chaetae and lateral flap with 7 smooth chaetae, and unguis with 4 inner teeth, b.p. tooth divided. *Pseudosinella unimacrochaetosa* sp. nov. differ from *P. certa* by basomedian and basolateral labial fields with A1–4 ciliate (smooth in *P. certa*), Abd IV with 3 central mac (2 in *P. certa*), tenent hair capitate (acuminate in *P. certa*), unguis with m.t. (absent in *P. certa*), and mucro teeth subequal and with basal spine reaching the apex of basal tooth, while *P. certa* has basal tooth smaller and basal spine exceding the apex of it [5,7,20]. See also the comparison these species in remarks of *P. taurina* sp. nov. and Table 1, Table 2 and Table 3.

### 3.3. Species Group with Prelabral Chaetae Not Bifurcate and Unguiculus “pe” Lamellae Acuminate

#### 3.3.1. *Pseudosinella alfanjeunguiculata* sp. nov. Bellini, Cipola, and Souza

**Figure 37:**
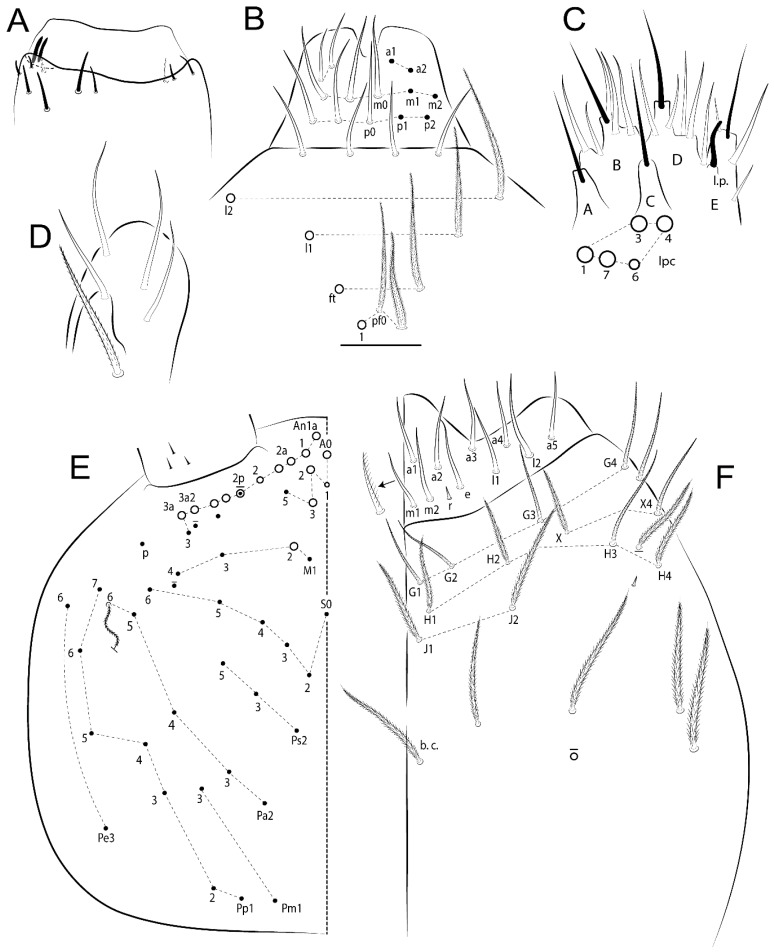
*Pseudosinella alfanjeunguiculata* sp. nov.: head. (**A**) Ant III apical organ (ventro-lateral view); (**B**) chaetotaxy of clypeus, prelabrum and labrum; (**C**) labial papillae (H omitted) plus proximal chaetae; (**D**) maxillary palp and sublobal plate (left side), minute appendage omitted; (**E**) head dorsal chaetotaxy (left side); (**F**) basomedian and basolateral labial fields and complete postlabial chaetotaxy (right side).

**Figure 38:**
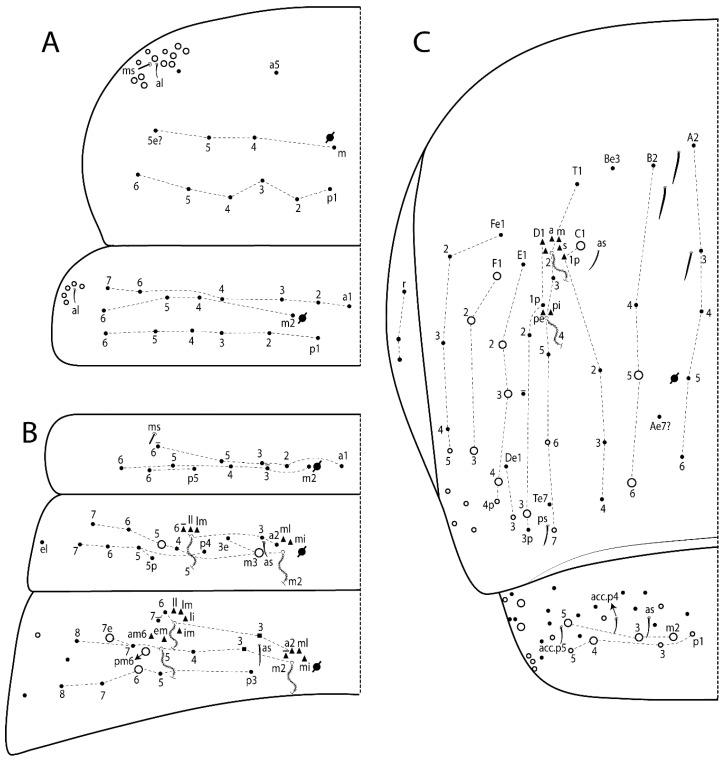
*Pseudosinella alfanjeunguiculata* sp. nov.: dorsal chaetotaxy. (**A**) Th II–III; (**B**) Abd I–III; (**C**) Abd IV–V.

**Figure 39:**
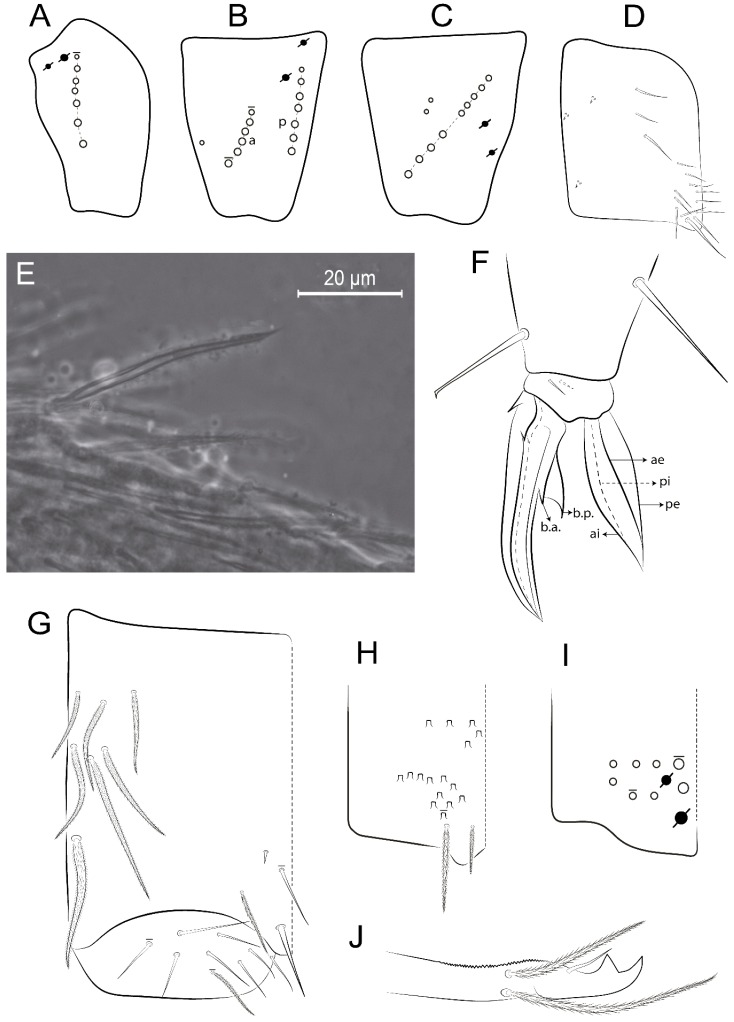
*Pseudosinella alfanjeunguiculata* sp. nov.: trunk appendages. (**A–C**) subcoxa I–III, respectively; (**D**) trochanteral organ; (**E**) modified chaeta of tibiotarsus III; (**F**) distal tibiotarsus and empodial complex III (anterior view); (**G**) collophore (lateral view); (**H**) distal part of manubrium (ventral view); (**I**) manubrial plate (dorsal view); (**J**) distal dens and mucro (inner view).

Figure 37, Figure 38 and Figure 39, Table 1 and Table 5

*Type material.* Holotype female in slide (2546/CRFS-UEPB): Brazil, Minas Gerais State, Morro do Pilar municipality, 7 km to north of city, “MP-01” cavity, 19°09′15.1″ S, 43°24′12.7″ W, 746 m, 28.ii.2012, Bessi et al. coll. Paratypes in slides (2507–11, 2533, 2535–39, 2541–44, 2572/CRFS-UEPB): 1 male, 8 females and 7 juveniles, same data as holotype. Paratypes in slides (2502, 2504–06, 2550/CRFS-UEPB): 2 females and 3 juveniles, same data as holotype, except 03-06.x.2011, Andrade et al. coll. Paratypes in slides (2532, 2534/CRFS-UEPB donated to INPA/085): 1 male and 1 female, same data as holotype. Paratypes in slides (2512, 2549/CRFS-UEPB donated to INPA/085): 2 females, same data as holotype, except 03-06.x.2011, Andrade et al. coll.

*Description.* Total length (head + trunk) of specimens 0.89–1.38 mm (n = 4), holotype 1.11 mm. Head. Ratio antennae:trunk = 1:2.87–3.05 (n = 4), holotype 1:3.1; Ant III smaller than Ant II length; Ant segment ratio as I:II:III:IV = 1:1.45–2.75:1.14–2:3.09–4, holotype 1:1.67:1.29:3,19. Ant IV with numerous common sens dorsally and ventrally (b–c), ventrally with at least 1 subapical f and 2 proximal e sens, plus several ordinary chaetae (i–k); Ant III common sens b–c, apical organ with 2 apical sens clubs surrounded by at least 7 sens c and 3 g (Figure 37A); Ant II common sens b–c, with 1 dorso-distal f, 1 dorso-lateral h, and 2 ventro-subdistal d sens (Figure 5B). Clypeal formula with 4 (l1–2), 2 (ft), and 3 (pf0–1) ciliate chaetae, all apically acuminate, pf1 thicker, l2 longer (Figure 37B). Prelabral chaetae smooth and not bifurcate. Labral chaetae smooth, no modifications (Figure 37B). Labial papilla D with 4 appendages; papilla E with l.p. finger-shaped, sinuous or almost straight and surpassing the base of apical appendage (Figure 37C). Labial proximal chaetae smooth (lpc1, 3–4, and 6–7), lpc6 shorter and slightly thinner than others (Figure 37C). Maxillary palp with b.c. weakly ciliate and 1.4 longer than t.a. (Figure 37D, anterior sublobal minute appendage not represented). Head dorsal chaetotaxy (Figure 37E) with 10–11 “An” (An1a–3), 5 “A” (A0–3, A5, and A2a absent), 4 “M” (M1–4), 6 “S” (S0 and S2–6), 3 “Ps” (Ps2–3 and Ps5), 4 “Pa” (Pa2–5), 2 “Pm” (Pm1 and Pm3), 7 “Pp” (Pp1–7), and 2 “Pe” (Pe3 and 6) chaetae; An1a–3a, A0, A2–3, and M2 as mac (M2 as mic in some juveniles); interocular p mic present; head posterior region with at least 8 cervical spine-like mac (not represented in Figure 37E). Basomedian and basolateral labial fields with a1–5, m1–2, e, and l1–2 smooth; r reduced; M1 also as unilaterally ciliate chaetae in some specimens, always slightly smaller than m2, l2 longer than others (Figure 37F). Ventral chaetotaxy with 12–13 ciliate chaetae, 7 weakly ciliate chaetae anteriorly, and 1 reduced lateral spine; postlabial formula 4 (G1–4), 2 (X and X4), 4 (H1–4), and 2 (J1–2) chaetae, 3–4 latero-posterior chaetae thicker, b.c. present (Figure 37F).

Thorax dorsal chaetotaxy (Figure 38A). Th II a, m, and p series with 1 (a5), 4 (m–5e?), and 6 (p1–6) mic, respectively. Th III a, m, and p series with 6 (a1–4 and a6–7), 4 (m2 and m4–6), and 6 (p1–6) mic, respectively. Ratio Th II:III = 1.34:1 in holotype.

Abdomen dorsal chaetotaxy (Figure 38B,C). Abd I a, m, and p series with 4–5 (a1–3 and a5–6), 5 (m2–6), and 2 (p5–6) mic, respectively, a3 not remarkably smaller than others. Abd II a, m, and p series with 2–3 (a2–3 and a6), 6 (m3–3e and m4–7), and 5 (p4–7) chaetae, respectively, m3 and m5 as mac; a5 and m2 bothriotricha surrounded by 2–3 (a2, lm, and ll) and 2 (ml and mi) fan-shaped chaetae, respectively. Abd III a, m, and p series with 3–4 (a2–3 and a6–7), 7 (m3–4, am6, pm6, and m7–8), and 5 (p3 and p5–8) chaetae, respectively, m7e, pm6, and p6 as mac; a3 and m3 as reduced mic; as sens elongated; and ms absent; m2 bothriotrichum associated with 3–4 (a2, ml, mi, plus another one) and a5 and m5 bothriotricha with 6 (am6, li, lm, ll, im, and em) fan-shaped chaetae. Abd IV A–Fe series with 5 (A2–6), 1 (Ae7?), 4 (B2 and B4–6), 1 (Be3), 5 (C1–4), 5 (T1, T3, and T5–7), 1 (Te7), 5 (D1–3p), 2 (De1 and De3), 5 (E1–4p), 3 (F1–3), and 5 (Fe1–5) chaetae, respectively, with 3 central (B5–6 and C1) and 7 lateral mac (D3, E2–4, and F1–3); T2 and T4 bothriotricha surrounded by 6 (D1, a, s, m, C1p, plus 1 other) and 2 (pe and pi) fan-shaped chaetae, respectively; 5 sens present, ps type I, as plus 3 anterior type II, r series laterally with 3 chaetae. Abd V m and p series with 3 (m2–3 and m5) and 1 (p4) central mac, respectively. Ratio Abd III:IV = 1:5.84 in holotype. Th II–Abd IV formula with 00|010 + 31 + 2 mac.

Legs. Subcoxa I with 6–7 chaetae and 2 psp; subcoxa II with “a” row of 4–6 chaetae plus 1 anterior chaeta, “p” row with 7 chaetae and 2 psp; subcoxa III with one row of 9 plus 2 anterior chaetae and 2 posterior psp (Figure 39A–C). Trochanteral organ with about 15 spine-like chaetae, plus 3 small spines on anterior side (Figure 39D). Tibiotarsus I–III formula with 1, 3, and 3 mac type IV (Figure 39E). Unguis outer side with paired teeth straight and not developed on proximal one fifth; inner side with slender lamella in sickle-shape and 2 basal paired unequal teeth, b.p. tooth larger, m.t. and a.t. absent. Unguiculus in lance-shape, all lamellae smooth and acuminate (ai, ae, pi, and pe), except ai gently excavate or truncate, ae and pi interrupted on distal three fourth (Figure 39F); ratio unguis:unguiculus in holotype = 1.55:1. Tibiotarsal smooth chaeta about 0.93 shorter than unguiculus; tenent hair weakly capitate and about 0.44 smaller than unguis outer edge.

Collophore (Figure 39G). Anterior side with 7 ciliate chaetae apically acuminate, 3 proximal thin, one slightly longer; 3 subdistal larger, one longer; and 1 distal mac; posterior side distally with 1–2 smooth chaetae, 1 ciliate chaeta, and 1 subdistal reduced spine; lateral flap with 7–8 smooth (1 larger) and 0–1 ciliate chaeta.

Furcula. Only with ciliate chaetae and scales. Manubrium ventrally with 5 subapical and about 10–11 distal scales; manubrial plate with 6–8 ciliate chaetae (1–2 inner mac) and 2 psp (Figure 39H,I). Mucro basal tooth gently larger than distal tooth, basal spine surpassing the apex of basal tooth (Figure 39J).

*Etymology.* Refers to sword-shaped unguiculus (from Arabic: al-handjar–alfanje, scimitar).

*Remarks. Pseudosinella alfanjeunguiculata* sp. nov. resembles *P. christianseni* Salmon 1964 from United States, and *P. concii* Gisin, 1950; *P. dallaii* Gisin and Gama, 1970; and *P. insubrica* Gisin and Gama, 1969 from Europe, all belonging *vandeli* species group [7,24,51]. They are similar in eyes absent, Th II–Abd IV with 0, 0, 0, 1, 0, and 3 central mac, unguis slender in sickle-shape with basal teeth near to basis, and sword-shaped unguiculus and with 1–2 lamellae (ae and pi) interrupted, and toothless pe lamella (Table 4 and Table 5). However, *P. alfanjeunguiculata* sp. nov. differs from these species by pigments absent (present in the other taxa except *P. christianseni*), antennae with one third or less of the body length (longer in *P. christianseni*), Ant III without swollen sens (present in these species). The new species also differs in head with 4 (A0, A2–3, and M2) mac (all absent in P. concii and *P. dallaii*, A3 present in *P. insubrica*, M2 absent in *P. christianseni*), and basomedian labial field with spine-like reduced r, while in the other species this chaeta is ciliate (smooth only in *P. christianseni*). They still differ by tenent hair capitate (acuminate in these species), and unguis with basal teeth unequal and m.t. absent (basal teeth equal and m.t. present in these species). Due to empodial complex shape, *P. alfanjeunguiculata* sp. nov. also resembles other species of *vandeli* group, but the new species differs by absence of eyes and mac on Th II–III [24,50,52].

#### 3.3.2. *Pseudosinella aphelabiata* sp. nov. Bellini, Cipola, and Souza

**Figure 40:**
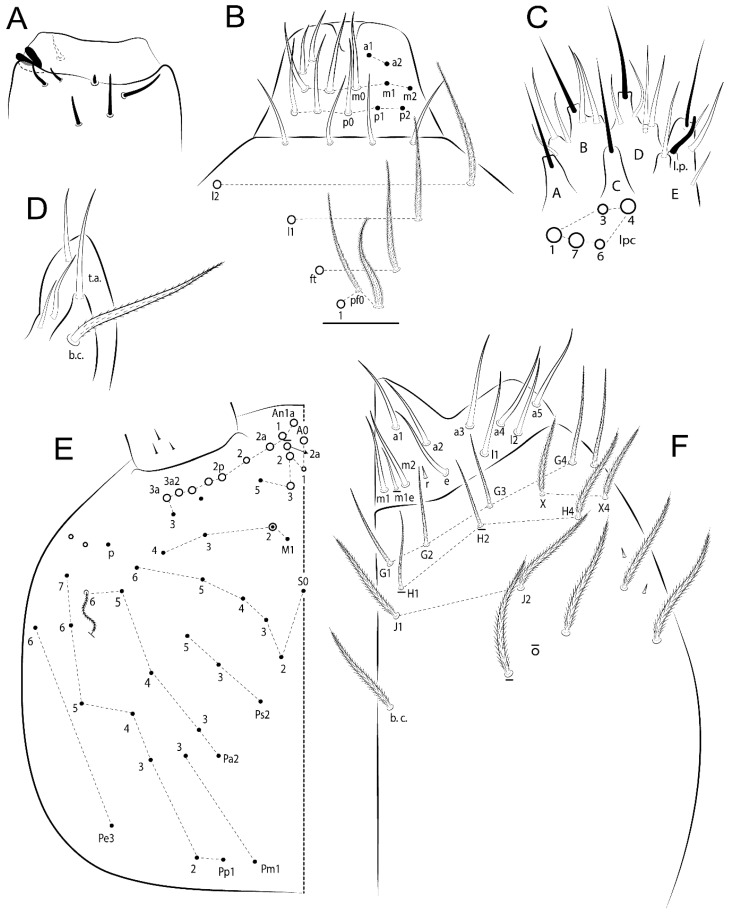
*Pseudosinella aphelabiata* sp. nov.: head. (**A**) Ant III apical organ (ventro-lateral view); (**B**) chaetotaxy of clypeus, prelabrum, and labrum; (**C**) labial papillae (H omitted) plus proximal chaetae; (**D**) maxillary palp and sublobal plate (right side), minute appendage omitted; (**E**) head dorsal chaetotaxy (left side); (**F**) basomedian and basolateral labial fields and complete postlabial chaetotaxy (right side).

**Figure 41:**
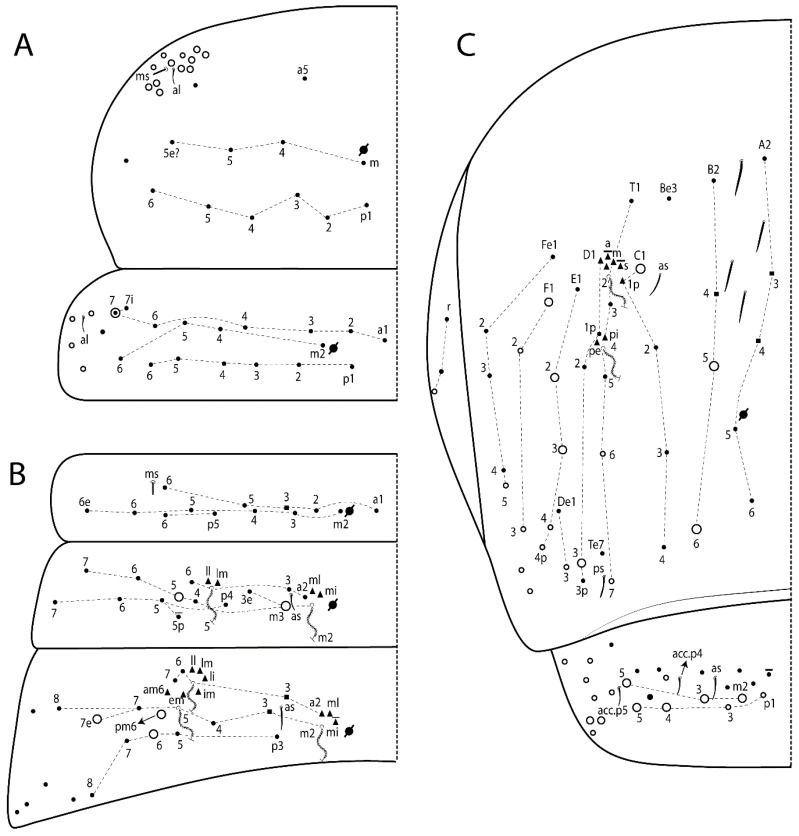
*Pseudosinella aphelabiata* sp. nov.: dorsal chaetotaxy. (**A**) Th II–III; (**B**) Abd I–III; (**C**) Abd IV–V.

**Figure 42:**
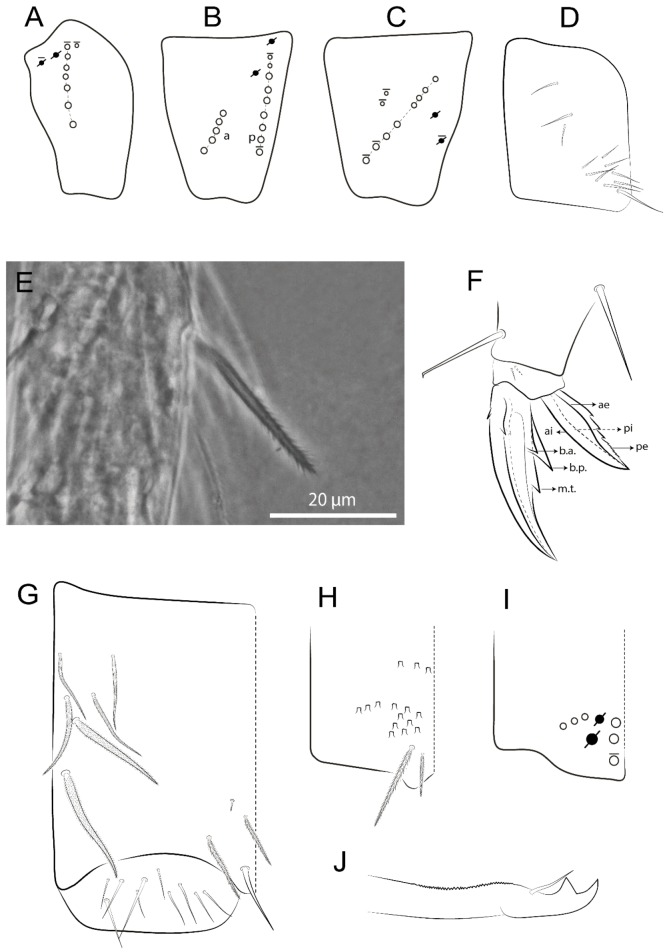
*Pseudosinella aphelabiata* sp. nov.: trunk appendages. (**A**–**C**) subcoxa I–III, respectively; (**D**) trochanteral organ; (**E**) modified chaeta of tibiotarsus III; (**F**) distal tibiotarsus and empodial complex III (anterior view); (**G**) collophore (lateral view); (**H**) distal part of manubrium (ventral view); (**I**) manubrial plate (dorsal view); (**J**) distal dens and mucro (inner view).

Figure 40, Figure 41 and Figure 42, Table 1 and Table 5

*Type material.* Holotype female in slide (12510/CRFS-UEPB): Brazil, Minas Gerais State, São Sebastião do Rio Preto municipality, next to “Estátua de Dominguinhos da Pedra” “MCFC” cavity, 19°20′33.3″ S, 43°18′27.5″ W, 829 m, 26.vi. -06.vii.2018, Carste et al. coll. Paratypes in slides (12496, 12500–02, 12504–09, 12511–14/CRFS-UEPB): 2 males, 12 females, same data as holotype. Paratypes in slides (12515–16/CRFS-UEPB donated to INPA/086): 2 females, same data as holotype.

*Description.* Total length (head + trunk) of specimens 0.81–1.24 mm (n = 10), holotype 0.93 mm.

Head. Ratio antennae:trunk = 1:2.56–3.16 (n = 10), holotype 1:2.8; Ant III smaller than Ant II length; Ant segment ratio as I:II:III:IV = 1:1.45–2.29:0.95–1.75:2.27–4.14, holotype 1:1.87:1:3. Ant IV with numerous common sens dorsally and ventrally (b–c), at least 2 e and 1 h sens ventrally, plus several ordinary chaetae (i–k); Ant III with common sens b–c plus 1 h ventrally, apical organ with 2 apical slightly swollen sens clubs surrounded by at least 6 sens c and 1 h (Figure 40A); Ant II with common sens b–c, with 1 dorso-distal f and 2 dorso-proximal h sens. Clypeal formula with 4 (l1–2), 2 (ft), and 3 (pf0–1) ciliate chaetae, all apically acuminate, pf1 thicker, ft and l1–2 longer (Figure 40B). Prelabral chaetae smooth and not bifurcate. Labral chaetae smooth, no modifications (Figure 40B). Labial papilla D with 4 appendages; papilla E with l.p. finger-shaped, sinuous, and surpassing the base of apical appendage (Figure 40C). Labial proximal chaetae smooth (lpc1, 3–4, and 6–7), lpc6 and lpc3 shorter and slightly thinner than others (Figure 40C). Maxillary palp with b.c. weakly ciliate and 1.25 longer than t.a. (Figure 40D, anterior sublobal minute appendage not represented). Head dorsal chaetotaxy (Figure 40E) with 10 “An” (An1a–3), 5–6 “A” (A0–3, A5, and A2a present or absent), 4 “M” (M1–4), 6 “S” (S0 and S2–6), 3 “Ps” (Ps2–3 and Ps5), 4 “Pa” (Pa2–5), 2 “Pm” (Pm1 and Pm3), 7 “Pp” (Pp1–7), and 2 “Pe” (Pe3 and 6) chaetae; An1a–3a, A0, A2–3, and M2 as mac (M2 rarely as mic); interocular p mic present; head posterior region with at least 7 cervical spine-like mac (not represented in Figure 40E). Basomedian and basolateral labial fields with a1–5, m1–1e, m2, e, and l1–2 smooth; r reduced; and m1 slightly smaller or subequal to m2; only two specimens with m1e, clearly smaller than m1–2 (Figure 40F). Ventral chaetotaxy with 9–11 ciliate chaetae, 5–7 weakly ciliate chaetae anteriorly, and 2 reduced lateral spines; postlabial formula 4 (G1–4), 2 (X and X4), 1–3 (H1–2 and H4), and 2 (J1–2) chaetae, 4–6 latero-posterior chaetae thicker, b.c. present (Figure 40F).

Thorax dorsal chaetotaxy (Figure 41A). Th II a, m, and p series with 1 (a5), 4 (m–5e?), and 6 (p1–6) mic, respectively; one lateral mic (possibly p6e) also present. Th III a, m, and p series with 7 (a1–4 and a6–7i), 4 (m2 and m4–6), and 6 (p1–6) mic, respectively; a7 as mac in some specimens. Ratio Th II:III = 2.06:1 in holotype.

Abdomen dorsal chaetotaxy (Figure 41B,C). Abd I a, m, and p series with 5 (a1–3 and a5–6), 6 (m2–6e), and 2 (p5–6) mic, respectively, a3 as reduced mic. Abd II a, m, and p series with 3 (a2–3 and a6), 6 (m3–3e and m4–7), and 4–5 (p4–7 and p5p present or absent) chaetae, respectively, m3 and m5 as mac; a5 and m2 bothriotricha surrounded by 2 (lm and ll) and 2(mL and mi) fan-shaped chaetae, respectively. Abd III a, m, and p series with 4 (a2–3 and a6–7), 7 (m3–4, am6, pm6, and m7–8), and 5 (p3 and p5–8) chaetae, respectively, pm6, p6 and p7e as mac, a3 and m3 as reduced mic, as sens elongated, ms absent; m2 bothriotrichum associated with 2–3 (a2, ml, and mi) and a5 and m5 bothriotricha with 6 (am6, li, lm, ll, im, and em) fan-shaped chaetae. Abd IV A–Fe series with 5 (A2–6), 4 (B2 and B4–6), 1 (Be3), 5 (C1–4), 5 (T1, T3, and T5–7), 1 (Te7), 5 (D1–3p), 2 (De1 and De3), 5 (E1–4p), 3 (F1–3), and 5 (Fe1–5) chaetae, respectively, with 3 central (B5–6 and C1) and 4 lateral mac (D3, E2–3, and F1); T2 and T4 bothriotricha surrounded by 4–6 (D1, a, s, m, C1p, plus 1 other) and 2 (pe and pi) fan-shaped chaetae, respectively; 6 sens present, ps type I, as plus 4 anterior type II, r series laterally with 3 chaetae. Abd V m and p series with 3 (m2–3 and m5) and 2 (p4–5) central mac, respectively. Ratio Abd III:IV = 1:3.73 in holotype. Th II–Abd IV formula with 00|010 + 31 + 2 mac.

Legs. Subcoxa I with 6–8 chaetae and 1–2 psp; subcoxa II with “a” row of 5 chaetae, “p” row with 7–9 chaetae and 2 psp; subcoxa III with one row of 6–8 plus 0–2 anterior chaetae and 1–2 posterior psp (Figure 42A–C). Trochanteral organ with about 12 spine-like chaetae (Figure 42D). Tibiotarsus I–III formula with 1, 3, and 3 mac type V (Figure 42E). Unguis outer side with paired teeth straight and not developed on proximal one fourth; inner side with wide lamella and 3 teeth, basal pair unequal, b.p. tooth larger, but not reaching the m.t. apex, m.t. on distal half and subequal to b.a., a.t. absent. Unguiculus with all lamellae smooth and acuminate (ai, ae, pi, and pe), except pe serrate (Figure 42F); ratio unguis:unguiculus in holotype = 1.6:1. Tibiotarsal smooth chaeta about 0.87 shorter than unguiculus; tenent hair weakly capitate and about 0.80 smaller than unguis outer edge.

Collophore (Figure 42G). Anterior side with 6 ciliate apically acuminate chaetae, 3 proximal thin and subequal in length and 1 subdistal larger, 1 subdistal and 1 distal mac; posterior side distally with 1 smooth chaeta, 2 ciliate chaetae (outer chaeta larger), and 1 subdistal reduced spine; lateral flap with 10 smooth chaetae, 2 larger.

Furcula. Only with ciliate chaetae and scales. Manubrium ventrally with 3 subapical and about 12 distal scales; manubrial plate with 5–6 ciliate chaetae (2–3 inner mac) and 2 psp (Figure 42H,I). Mucro basal tooth gently larger than distal tooth, basal spine surpassing the apex of basal tooth (Figure 42J).

*Etymology.* Refers to labial smooth chaetae (Figure 42F) on basomedian and basolateral fields (from Greek: aphelo-smooth).

*Remarks. Pseudosinella aphelabiata* sp. nov. resembles *P. nata* Christiansen and Bellinger 1980 and *P. diamantinensis* sp. nov. by basomedian and basolateral labial fields with m2, e, and l1–2 smooth and r reduced; Th II–Abd III with 0, 0, 0, 2, and 0 central mac, unguis with 3 inner teeth, and mucro spine surpassing the apex of basal tooth (Table 1, Table 4, and Table 5). *Pseudosinella aphelabiata* sp. nov. differs from *P. nata* in Abd IV with 3 central mac (2 in *P. nata*), unguis basal teeth unequal (equal in *P. nata*) and m.t. simple (double in *P. nata*), and unguiculus pe lamella serrate (smooth in *P. nata*) [5,7]. However, *P. aphelabiata* sp. nov. is more similar to *P. diamantinensis* sp. nov. by head M2 chaeta as mac or mic, labral chaetae smooth, Abd III with 3 lateral mac, Abd IV 3 central mac, unguiculus pe lamella serrate (see Table 1 and Table 5). The new species differs from *P. aphelabiata* sp. nov. in prelabral chaetae smooth (ciliate *P. diamantinensis* sp. nov.), lpc 3 and lp6 smaller (only lp6 smaller in *P. diamantinensis* sp. nov.), and collophore anteriorly with 6 chaetae, posteriorly with 2 ciliate and 1 smooth chaetae, and lateral flap only with smooth chaetae. In *P. diamantinensis* sp. nov. there are 5–7 anterior chaetae and posteriorly 5 ciliate and 2 smooth chaetae, and ciliate and smooth chaetae on lateral flap. They still differ in tibiotarsus with mac type V (type IV in *P. diamantinensis* sp. nov.), tenent hair capitate (acuminate in *P. diamantinensis* sp. nov.), and unguis b.p. tooth larger than m.t. (subequal in *P. diamantinensis* sp. nov.).

#### 3.3.3. *Pseudosinella cearensis* sp. nov. Oliveira, Brito, and Cipola

**Figure 43:**
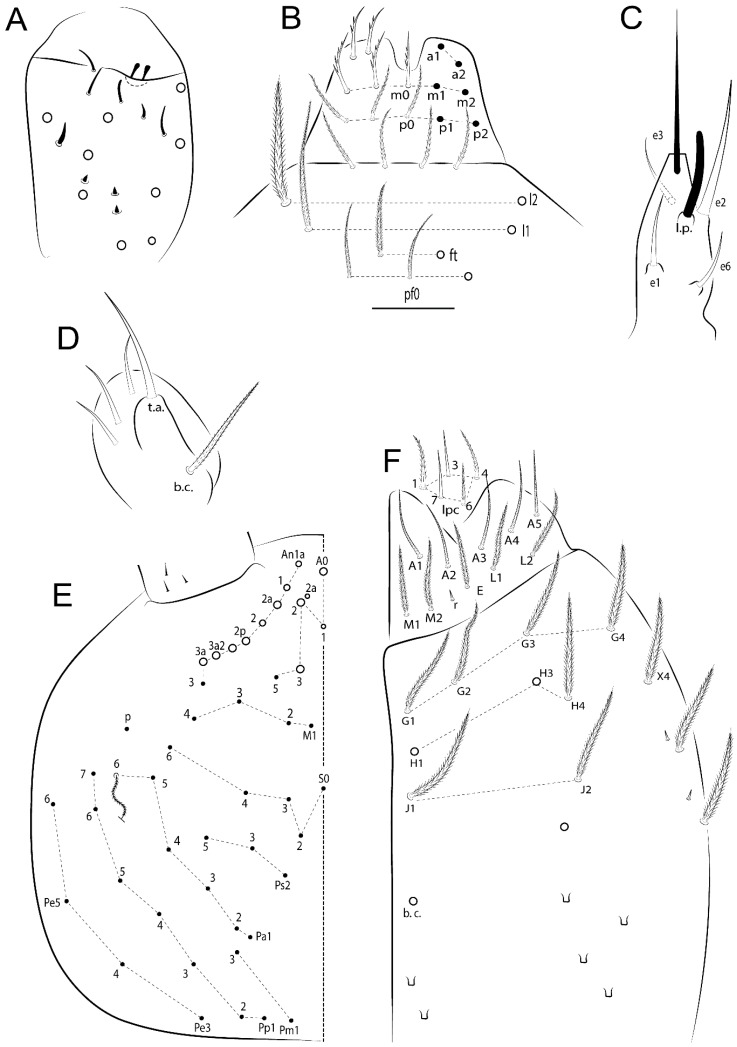
*Pseudosinella cearensis* sp. nov.: head. (**A**) Ant III apical organ (lateral view); (**B**) chaetotaxy of clypeus, prelabrum, and labrum; (**C**) labial papilla E; (**D**) maxillary palp and sublobal plate (right side), minute appendage omitted; (**E**) head dorsal chaetotaxy (left side); (**F**) labial proximal chaetae, basomedian and basolateral labial fields, and complete postlabial chaetotaxy (right side).

**Figure 44:**
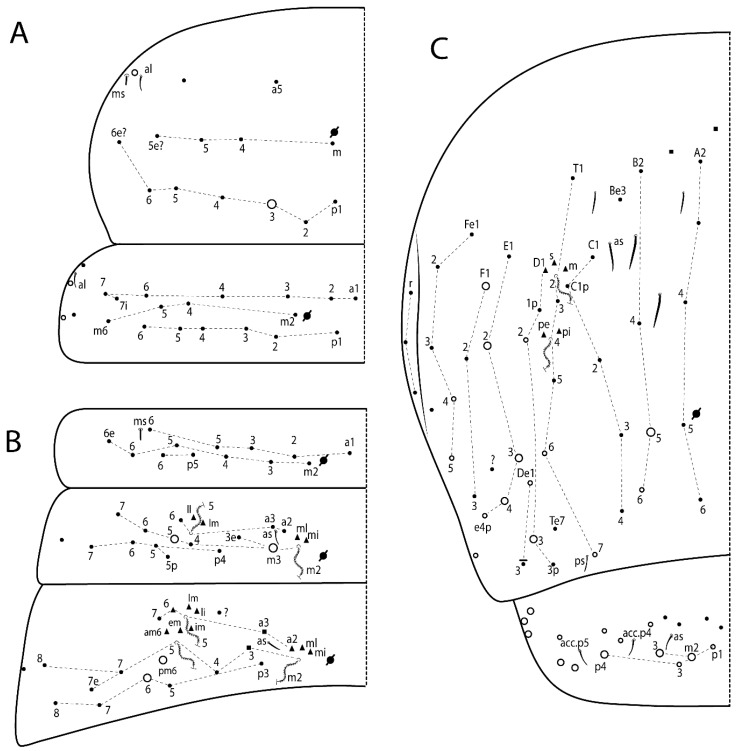
*Pseudosinella cearensis* sp. nov.: dorsal chaetotaxy. (**A**) Th II–III; (**B**) Abd I–III; (**C**) Abd IV–V.

**Figure 45:**
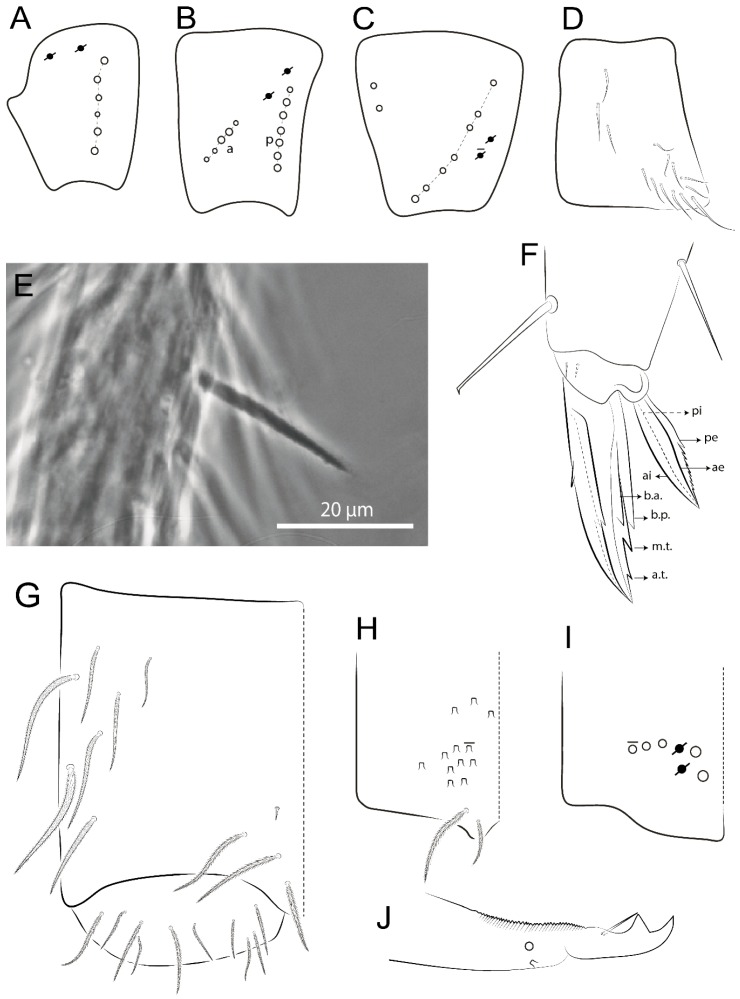
*Pseudosinella cearensis* sp. nov. (**A–C**) subcoxa I–III, respectively; (**D**) trochanteral organ; (**E**) modified chaeta of tibiotarsus; (**F**) distal tibiotarsus and empodial complex III (anterior view); (**G**) collophore (lateral view); (**H**) distal part of manubrium (ventral view); (**I**) manubrial plate (dorsal view); (**J**) distal dens and mucro (outer view).

Figure 43, Figure 44 and Figure 45, Table 1 and Table 5

*Type material.* Holotype female in slide (9362/CRFS-UEPB): Brazil, Ceará State, Santa Quitéria municipality, between the mountain and “Riacho do Mulungu”, 04°33′37.5″ S, 39°45′49.0″ W, 510 m, 15-21.vii.2014, Pellegati and Pedroso coll. Paratypes in slides (9363-64/CRFS-UEPB): 1 females and 1 juvenile, same data as holotype, except 04°33′35.0″ S, 39°45′26.8″ W, 535 m, and 04°33′50.7″ S, 39°46′16.8″ W, 514 m, 03.21.vii.2014. Paratypes in slides (9361/CRFS-UEPB donated to INPA/087): 1 female, as above.

*Description.* Total length (head + trunk) of specimens 0.79–1.16 mm (n = 4), holotype 0.99 mm.

Head. Ratio antennae:trunk = 1:2.69–2.81 (n = 2), holotype 1:2.81; Ant II subequal to Ant III in length; Ant segment ratio as I:II:III:IV = 1:1.59–2.09:1.78–2.2:3.47–4.3, holotype 1:2.09:2.20:4.3. Ant IV dorsally with numerous sens (b–c) and chaetae (i–k). Ant III with 2 apical sens clubs surrounded by at least 1 sens d, 3 c, 2 e, and 3 h (Figure 43A). Ant II with common sens b–c, with 1 dorso-distal c, 2 dorso-lateral c and 2 ventro-subdistal e sens. Clypeal formula with 4 (l1–2), 2 (ft), and 3 (pf0–1) ciliate chaetae, all apically acuminate, l1–2 larger (Figure 43B). Prelabral chaetae weakly ciliated and not bifurcate. Labral a1–2 chaetae with 2 lateral filaments, m0–2 chaetae with 3–4 lateral filaments, p0–2 chaetae weakly ciliated (Figure 43B). Labial papilla D with 3 appendages; papilla E with l.p. finger-shape, curved, and exceed the apical appendage (Figure 43C). Maxillary palp with b.c. smooth and 1.2 smaller than the t.a. (Figure 43D). Dorsal head chaetotaxy (Figure 43E) with 9 “An” (An1a–3), 6 “A” (A0–2a, A3, and A5), 4 “M” (M1–4), 5 “S” (S0, S2–4, and S6), 3 “Ps” (Ps2–3 and Ps5), 5 “Pa” (Pa1–5), 2 “Pm” (Pm1 and Pm3), and 7 “Pp” (Pp1–7) chaetae, 4 “Pe” (Pe3–6); An1a–3a, A0, A2, and A3 as mac; interocular p mic present; head posterior region with 8–10 cervical like-spine mac (not represented in Figure 43E). Basomedian and basolateral labial fields with A1–5 weak ciliate, M1–2, E, and L1–2 multiciliate, r reduced (Figure 43F). Labial proximal chaetae ciliate (lpc1, 4, and 6) and smooth (lpc3 and 7), lpc1 gently thicker and lpc6 smaller, others subequal. Ventral chaetotaxy with about 14 ciliate chaetae and 2 reduced lateral spines, postlabial formula 4 (G1–4), 1 (X4), 3 (H1 and H3–4), and 2 (J1–2) ciliate chaetae, b.c. present (Figure 43F).

Thorax dorsal chaetotaxy (Figure 44A). Th II a, m, and p series with 1 (a5), 4 (m and m4–5e?), and 7 (p2–6e?) chaetae, respectively, p3 as mac. Th III a, m, and p series with 7 (a1–4 and 6–7i), 4 (m2 and m4–6), and 6 (p1–6) mic, respectively. Ratio Th II:III = 1.58–1.79:1 (n = 4), holotype 1.58:1.

Abdomen dorsal chaetotaxy (Figure 44B,C). Abd I a, m, and p series with 5 (a1–3 and 5–6), 6 (m2–6e), and 2 (p5–6) mic, respectively. Abd II a, m, and p series with 3 (a2–3 and a6), 6 (m3–3e and m4–7), and 5 (p4–7) chaetae, respectively, m3 and m5 as mac; a5 and m2 bothriotricha with 2 (ll and lm) and 2 (ml and mi) fan-shaped chaetae, respectively. Abd III a, m, and p series with 4 (a2–3 and a6–7), 7 (m3–4, am6, pm6, and m7–8), and 5 (p3 and p5–8) chaetae, respectively, pm6 and p6 as mac, a3 and m3 as reduced mic, and as sens elongated, ms absent; m2 bothriotricha associated with 3 (a2, ml, and mi) and a5 and m5 bothriotricha with 6 (a6, am6, lm, li, em, and im) fan-shaped chaetae. Abd IV A–Fe series with 5 (A2–6), 4 (B2 and B4–6), 1 (Be3), 5 (C1–4), 5 (T1, T3, and T5–7), 1 (Te7), 5 (D1–3p), 1–2 (De1 and De3), 5 (E1–4p), 3 (F1–3), and 5 (Fe1–5) chaetae, respectively, with 1 central (B5) and 5 lateral mac (D3, E2–4, and F1); T2 and T4 bothriotricha with 3 (D1, s, and m) and 2 (pe and pi) fan-shaped chaetae, respectively; 6 sens present, ps type I, as and 4 of uncertain homology type II, r series laterally with 3 chaetae. Abd V m and p series with 2 (m2–3) and 1 (p4) central mac, respectively. Ratio Abd III:IV = 1:2.74–3.9 (n = 4), holotype 1:2.82. Th II–Abd IV formula with 10|010 + 20 + 1 mac.

Legs. Subcoxa I with “p” row with 6 chaetae and 2 psp; subcoxa II with “a” row with 5 chaetae, “p” row with 7 chaetae and 2 psp; subcoxa III with one row of 7 plus 2 anterior chaetae and 1–2 posterior psp (Figure 45A–C). Trochanteral organ with about 14 spine-like chaetae (Figure 45D). Tibiotarsus I–III formula with 1, 2, and 1 mac type VI (Figure 45E). Unguis outer side with paired teeth straight, not developed and on little more than distal half; inner side with wide lamella and 4 teeth, basal pair (b.a. and b.p.) subequal and not reaching the m.t. apex, m.t. on distal one third and gently larger to basal teeth, a.t. smaller on distal one sixth. Unguiculus with all lamellae smooth and acuminate (ai, ae, pi, and pe), except pe serrate (Figure 45F); ratio unguis:unguiculus in holotype = 1:0.66. Tibiotarsal smooth chaeta about 1.02 longer than unguiculus; tenent hair capitate and about 0.85 smaller than unguis outer edge.

Collophore (Figure 45G). Anterior side with 7 ciliate apically acuminate chaetae, 3 inner larger, others subequal in length; posterior side distally with 3 ciliated chaeta and 1 subdistal reduced spine; lateral flap with 10 ciliate chaetae (3 larger).

Furcula. Only with ciliate chaetae and scales. Manubrium ventrally with 3 subapical and 8–9 distal scales; manubrial plate with 4–5 ciliate chaetae (2 inner mac) and 2 psp (Figure 45H,I). Mucro basal tooth gently larger than distal tooth, basal spine reaching the apex of basal tooth (Figure 45J).

*Etymology.* Refers to type locality of new species, “Ceará” State.

*Remarks. Pseudosinella cearensis* sp. nov. resembles *P. strinatii* Christiansen, 1973 and *P. spurimarianensis* sp. nov. by tenent hair capitate, unguis with outer paired teeth on nearly distal half and with 4 inner teeth (a.t. present) (Table 1, Table 4, and Table 5). In chaetotaxy, *P. cearensis* sp. nov. resembles *P. strinatii* by Th II–Abd III with 1, 0, 0, 1, and 0 central mac, and as *P. spurimarianensis* sp. nov. by Th III–Abd IV with 0, 0, 1, 0, and 3 central mac. Consequently, *P. cearensis* sp. nov. differs these species by Th II with 1 mac (absent in *P. spurimarianensis* sp. nov.) and Abd IV with 3 (B5–6 and C1) mac (C1 absent in *P. strinatii*). On head, they differ by labral a1–2 and m0–2 chaetae with filaments and p0–2 ciliate (smooth in *P. spurimarianensis* sp. nov.), head M2 mac absent (present in both species), basomedian and basolateral labial fields with M1–2, E, and L1–2 multiciliate (smooth in *P. strinatii*, weakly ciliate in *P. spurimarianensis* sp. nov.). They still differ in tibiotarsus I–III formula with 1, 2, and 1 mac type VI (1, 3, and 3 mac type I in *P. spurimarianensis* sp. nov.), unguis basal teeth equal (unequal in *P. spurimarianensis* sp. nov.), and unguiculus pe lamellae serrate (smooth in *P. strinatii*) [51].

#### 3.3.4. *Pseudosinella diamantinensis* sp. nov. Bellini, Cipola, and Souza.

**Figure 46:**
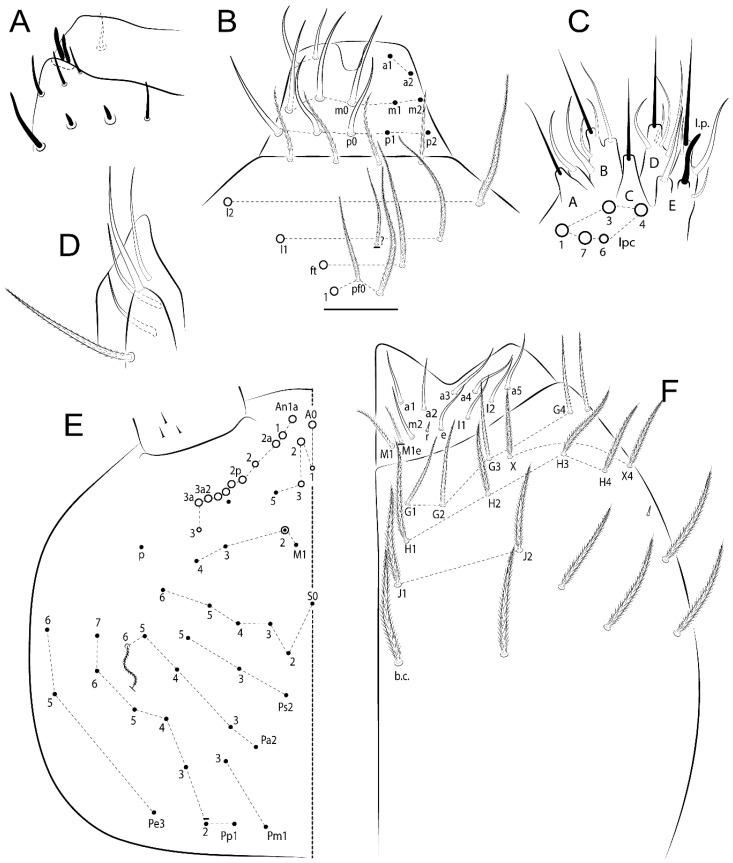
*Pseudosinella diamantinensis* sp. nov.: head. (**A**) Ant III apical organ (ventro-lateral view); (**B**) chaetotaxy of clypeus, prelabrum, and labrum; (**C**) labial papillae (H omitted) plus proximal chaetae; (**D**) maxillary palp and sublobal plate (left side), minute appendage omitted; (**E**) head dorsal chaetotaxy (left side); (**F**) basomedian and basolateral labial fields and complete postlabial chaetotaxy (right side).

**Figure 47:**
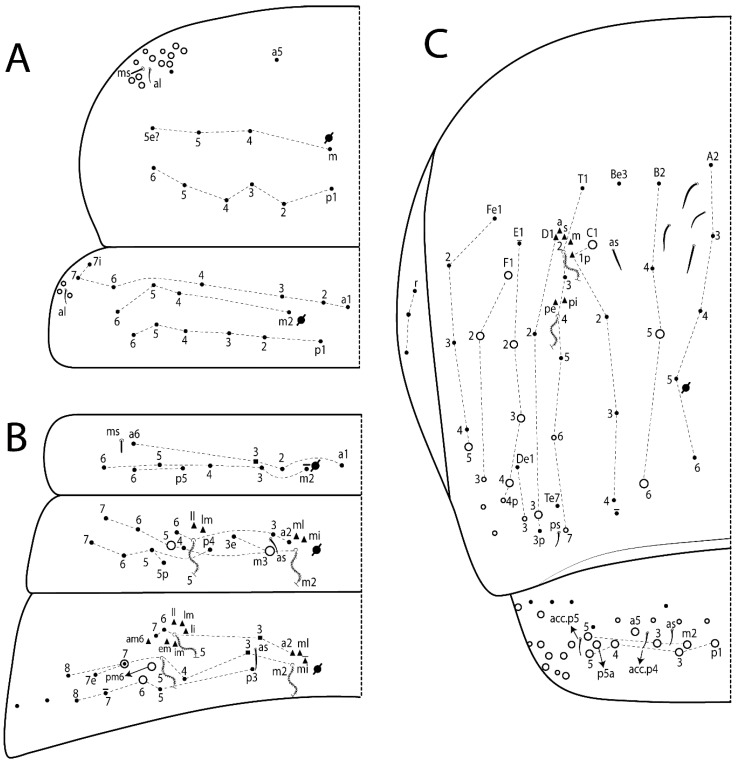
*Pseudosinelladiamantinensis* sp. nov.: dorsal chaetotaxy. (**A**) Th II–III; (**B**) Abd I–III; (**C**) Abd IV–V.

**Figure 48:**
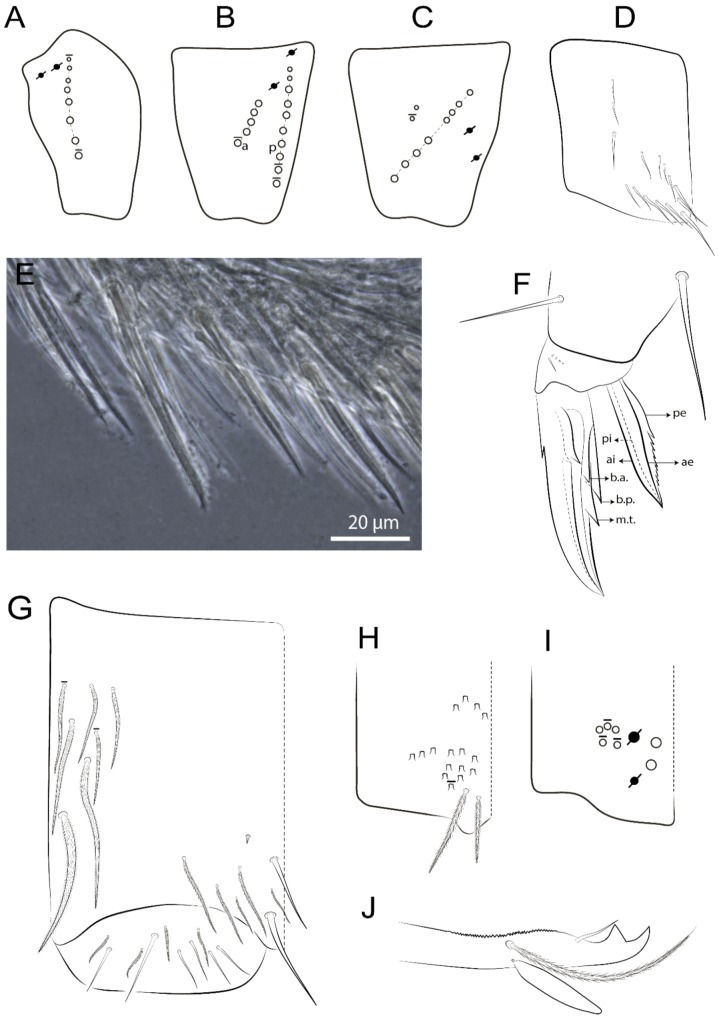
*Pseudosinella diamantinensis* sp. nov.: trunk appendages. (**A–C**) subcoxa I–III, respectively; (**D**) trochanteral organ; (**E**) modified chaeta of tibiotarsus III; (**F**) distal tibiotarsus and empodial complex III (anterior view); (**G**) collophore (lateral view); (**H**) distal part of manubrium (ventral view); (**I**) manubrial plate (dorsal view); (**J**) distal dens and mucro (inner view).

Figure 46, Figure 47 and Figure 48, Table 1 and Table 5

*Type material.* Holotype female in slide (7255/CRFS-UEPB): Brazil, Minas Gerais State, Diamantina municipality, “Extração” district, “Salitre” grotto, 18°16′41.4″ S, 43°32′09.0″ W, 1168 m, 03-10.xii.2015, Carste et al. coll. Paratypes in slides (7257–7260/CRFS-UEPB): 1 male and 3 females, same data as holotype. Paratype in slide (7256/CRFS-UEPB donated to INPA/088): 1 female, same data as holotype. Paratype in slide (9335/CRFS-UEPB donated to INPA/088): 1 female, same data as holotype, except 23-30.vi.2015.

*Description.* Total length (head + trunk) of specimens 0.99–1.61 mm (n = 3), holotype 1.61 mm.

Head. Ratio antennae:trunk = 1:2.25–2.46 (n = 3), holotype 1:2.46; Ant III smaller than Ant II length; Ant segment ratio as I:II:III:IV = 1:1.6–2.32:1.45–2.05:3.45–4.2, holotype 1:2.32:2.05:4. Ant IV with numerous common sens dorsally and ventrally (b–c), dorsally with 1 apical a in cavity, and at least 2 subapical f and 2 proximal e sens ventrally, plus several ordinary chaetae (i–k); Ant III with common sens b–c plus 2 e and 2 h ventrally, apical organ with 2 apical slightly sens clubs surrounded by at least 6 sens c and 2 h (Figure 46A); Ant II common sens b–c, with 1 dorso-distal f and 1 dorso-proximal h sens. Clypeal formula with 4 (l1–2), 2 (ft), and 3 (pf0–1) ciliate chaetae, all apically acuminate, pf1 thicker than pf0, l2 longer, holotype with one extranumerary chaeta on the right side (?) (Figure 46B). Prelabral chaetae weakly ciliate and not bifurcate. Labral chaetae smooth, no modifications (Figure 46B). Labial papilla D with 4 appendages; papilla E with l.p. finger-shaped, sinuous, and surpassing the base of apical appendage (Figure 46C). Labial proximal chaetae smooth (lpc1, 3–4, and 6–7), lpc6 shorter and slightly thinner than others (Figure 46C). Maxillary palp with b.c. weakly ciliate and 1.11 longer than t.a. (Figure 46D, anterior sublobal minute appendage not represented). Head dorsal chaetotaxy (Figure 46E) with 11 “An” (An1a–3), 5 “A” (A0–3 and A5), 4 “M” (M1–4), 6 “S” (S0 and S2–6), 3 “Ps” (Ps2–3 and Ps5), 4 “Pa” (Pa2–5), 2 “Pm” (Pm1 and Pm3), 6–7 “Pp” (Pp1–7), and 3 “Pe” (Pe3 and Pe5–6) chaetae; An1a–3a (An2 slightly smaller), A0, A2–3, and M2 as mac (M2 rarely mic); interocular p mic present; head posterior region with at least 9 cervical spine-like mac (not represented in Figure 46E). Basomedian and basolateral labial fields with a1–5, m2, e, and l1–2 smooth; M1–1e unilaterally ciliate; and r reduced; M1 slightly smaller or subequal to m2; only two specimens with M1e, clearly smaller than M1 (Figure 46F). Ventral chaetotaxy with 14 ciliate chaetae, 5 weakly ciliate chaetae anteriorly and 1 reduced lateral spine; postlabial formula 4 (G1–4), 1 (X4), 4 (H1–4), and 2 (J1–2) chaetae, 1 extra chaeta near G3, 5 latero-posterior chaetae slightly thicker, b.c. present (Figure 46F).

Thorax dorsal chaetotaxy (Figure 47A). Th II a, m, and p series with 1 (a5), 4 (m–5e?), and 6 (p1–6) mic, respectively. Th III a, m, and p series with 7 (a1–4 and a6–7i), 4 (m2 and m4–6), and 6 (p1–6) mic, respectively. Ratio Th II:III = 2.13:1 in holotype.

Abdomen dorsal chaetotaxy (Figure 47B,C). Abd I a, m, and p series with 4 (a1–3 and a6), 5–6 (m2–6), and 2 (p5–6) mic, respectively, a3 as reduced mic. Abd II a, m, and p series with 3 (a2–3 and a6), 6 (m3–3e and m4–7), and 5 (p4–7) chaetae, respectively, m3 and m5 as mac; a5 and m2 bothriotricha surrounded by 2 (lm and ll) and 2 (ml and mi) fan-shaped chaetae, respectively. Abd III a, m, and p series with 4 (a2–3, a6–7), 7 (m3–4, am6, pm6, and m7–8), and 4–5 (p3 and p5–8) chaetae, respectively, pm6 and p6 as mac, m7e mac or mic, a3 and m3 as reduced mic, as sens elongated, ms absent; m2 bothriotrichum associated with 2–3 (a2, ml, and mi) and a5 and m5 bothriotricha with 6 (am6, li, lm, ll, im, and em) fan-shaped chaetae. Abd IV A–Fe series with 5 (A2–6), 4 (B2 and B4–6), 1 (Be3), 5 (C1–4), 5 (T1, T3, and T5–7), 1 (Te7), 4 (D1–3p), 2 (De1 and De3), 4–5 (E1–4p), 3 (F1–3), and 5 (Fe1–5) chaetae, respectively, with 3 central (B5–6 and C1) and 7 lateral mac (D3, E2–4, F1–2, and Fe5); T2 and T4 bothriotricha surrounded by 5 (D1, a, s, m, and C1p) and 2 (pe and pi) fan-shaped chaetae, respectively; 6 sens present, ps type I, as plus 4 anterior type II, r series laterally with 3 mic; Abd. IV lacking clearly reduced mic. Abd V a, m, and p series with 1 (a5), 3 (m2–3 and m5), and 5 (p1, p3–5, plus p5a) central mac, respectively. Ratio Abd III:IV = 1:3.42 in holotype. Th II–Abd IV formula with 00|010 + 2(3)1 + 2 mac.

Legs. Subcoxa I with 6–8 chaetae and 2 psp; subcoxa II with “a” row of 4–5 chaetae, “p” row with 8–10 chaetae and 2 psp; subcoxa III with one row of 8 plus 1–2 anterior chaetae and 2 posterior psp (Figure 48A–C). Trochanteral organ with about 15 spine-like chaetae (Figure 48D). Tibiotarsus I–III formula with 1, 3, and 3 mac type IV (Figure 48E). Unguis outer side with paired teeth straight and not developed on proximal one third; inner side with wide lamella and 3 teeth, basal pair unequal, b.p. tooth larger, but not reaching the m.t. apex, m.t. on little more than distal half and subequal to b.a., a.t. absent. Unguiculus with all lamellae smooth and acuminate (ai, ae, pi, and pe), except ai gently truncate distally and pe serrate (Figure 48F); ratio unguis:unguiculus in holotype = 1.81:1. Tibiotarsal smooth chaeta about 1.14 longer than unguiculus; tenent hair acuminate and about 0.55 smaller than unguis outer edge.

Collophore (Figure 48G). Anterior side with 5–7 ciliate apically acuminate chaetae, 3–5 proximal thinner, the internal one larger; 1 subdistal longer and thicker than the proximal; and 1 distal mac; posterior side with 2 smooth chaetae, distal one larger; 5 ciliate chaetae (outer chaeta larger), and 1 subdistal reduced spine; lateral flap with 6 smooth (1 larger) and 4 ciliate chaetae.

Furcula. Only with ciliate chaetae and scales. Manubrium ventrally with 4 subapical and about 11–12 distal scales; manubrial plate with 4–7 ciliate chaetae (2 inner mac) and 2 psp (Figure 48H,I). Mucro basal tooth gently larger than distal tooth, basal spine surpassing the apex of basal tooth (Figure 48J).

*Etymology.* Refers to type locality of new species, “Diamantina” municipality.

*Remarks. Pseudosinella diamantinensis* sp. nov. resembles *P. nata* [51] and *P. aphelabiata* sp. nov. (Table 1, Table 4, and Table 5). However, *P. diamantinensis* sp. nov. differ from *P. nata* by Abd IV with 3 central mac (2 in *P. nata*), unguis basal teeth unequal (equal in *P. nata*) and m.t. simple (double in *P. nata*), and unguiculus pe lamella serrate (smooth in *P. nata*) [5,7]. *Pseudosinella diamantinensis* sp. nov. differ from *P. aphelabiata* sp. nov. by prelabral chaetae ciliate (smooth in *P. aphelabiata* sp. nov.), collophore anteriorly with up to 7 chaetae, posteriorly with 5 ciliate chaetae, and lateral flap with ciliate and smooth chaetae. They still differs in tibiotarsus with mac type IV (type V in *P. aphelabiata* sp. nov.), tenent hair acuminate (capitate in *P. aphelabiata* sp. nov.), and unguis b.p. tooth subequal to m.t. (larger in *P. diamantinensis* sp. nov.). See also the comparison these species in remarks of *P. aphelabiata* sp. nov. and Table 1, Table 4, and Table 5.

*Pseudosinella diamantinensis* sp. nov. also resembles *P. marianensis* sp. nov., but *P. diamantinensis* sp. nov. differ by lpc6 chaetae smaller (lpc3 and lpc6 smaller in *P. marianensis* sp. nov.), basomedian and basolateral labial fields with m2, e and l1–2 smooth (unilaterally ciliate in *P. marianensis* sp. nov.), postlabial G1–4 chaetae weakly ciliate (multiciliate in *P. marianensis* sp. nov.), abd IV laterally with F2 and Fe5 as mac (mic in *P. marianensis* sp. nov.). They differ also in the posterior collophore with *P. diamantinensis* sp. nov. having 5 ciliate chaetae and 1 spine while *P. marianensis* sp. nov. have 2 ciliate chaetae and 2 spines.

#### 3.3.5. *Pseudosinella marianensis* sp. nov. Bellini, Cipola, and Souza

**Figure 49:**
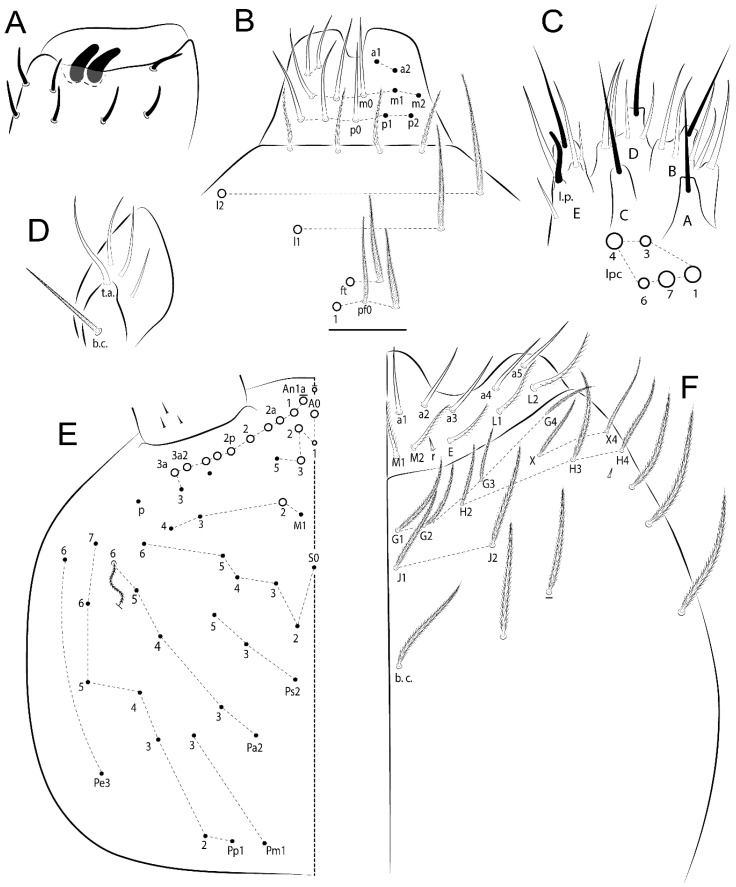
*Pseudosinella marianensis* sp. nov.: head. (**A**) Ant III apical organ (ventro-lateral view); (**B**) chaetotaxy of clypeus, prelabrum, and labrum; (**C**) labial papillae (H omitted) plus proximal chaetae (left side); (**D**) maxillary palp and sublobal plate (left side), minute appendage omitted; (**E**) head dorsal chaetotaxy (left side); (**F**) basomedian and basolateral labial fields and complete postlabial chaetotaxy (right side).

**Figure 50:**
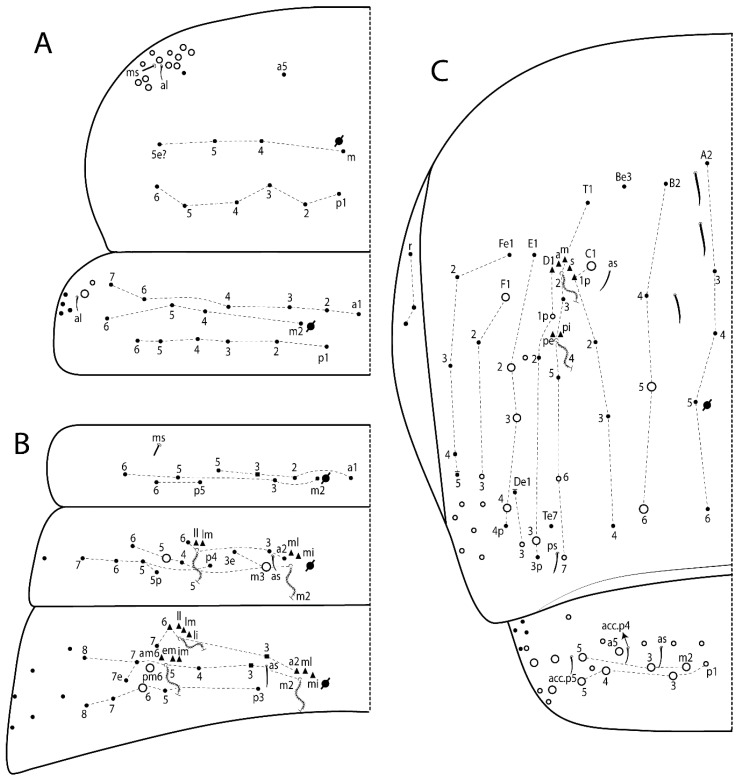
*Pseudosinella marianensis* sp. nov.: dorsal chaetotaxy. (**A**) Th II–III; (**B**) Abd I–III; (**C**) Abd IV–V.

**Figure 51:**
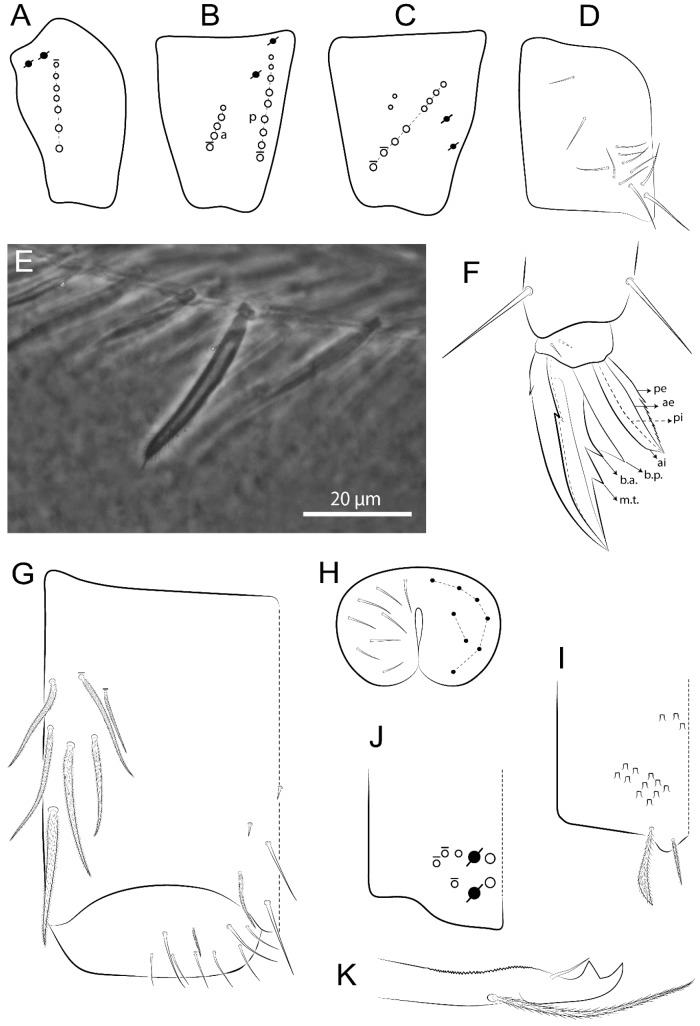
*Pseudosinella marianensis* sp. nov.: trunk appendages. (**A**–**C**) subcoxa I–III, respectively; (**D**) trochanteral organ; (**E**) modified chaeta of tibiotarsus III; (**F**) distal tibiotarsus and empodial complex III (anterior view); (**G**) collophore (lateral view); (**H**) male genital plate (ventral view); (**I**) distal part of manubrium (ventral view); (**J**) manubrial plate (dorsal view); (**K**) distal dens and mucro (inner view).

Figure 49, Figure 50 and Figure 51, Table 1 and Table 5

*Type material.* Holotype female in slide (13550/CRFS-UEPB): Brazil, Minas Gerais State, Mariana municipality, between Camargos and Antônio Pereira districts, 20°17′06.8″ S, 43°25′20.7″ W, 790 m, 13.ii.2019, Bioespeleo et al. coll. Paratypes in slides (13556–57/CRFS-UEPB): 1 male and 1 female, same data as holotype, except 14.ii.2019. Paratype in slide (13562/CRFS-UEPB): 1 male, same data as holotype, except 27.ix.2018. Paratypes in slides (13961–63/CRFS-UEPB): 1 female and 2 juveniles, same data as holotype, except 11-12.iii.2019, Bioespeleo et al. coll. Paratypes in slide (1372/CRFS-UEPB): 1 female and 1 juvenile, same data as holotype, except 24.ix.2011. Paratypes in slides (1663, 1725/CRFS-UEPB): 2 juveniles, same data as holotype, except 06–16.vi.2011. Paratypes in slides (1079, 1128/CRFS-UEPB): 1 female and 1 juvenile, same data as holotype, except 27-29.vi.2012, Borges and Souza coll. Paratype in slides (2214/CRFS-UEPB): 1 male, same data as holotype, except 17.xii.2012. Paratypes in slides (2222-23, 2226, 2260/CRFS-UEPB): 4 juveniles, same data as holotype, except 18.x.2018. Paratypes in slides (13624–626/CRFS-UEPB): 1 male and 2 females, same data as holotype, except near to “Piracicaba” river, in Santa Rita Durão district, 20°09′53.3″ S, 43°23′40.4″ W, 876 m, 22–24.i.2018, Carste et al. coll. Paratype in slide (13129/CRFS-UEPB donated to INPA/089): 1 female, data as above, except 30.i.2018, Bioespeleo et al. coll. Paratype in slide (13964/CRFS-UEPB donated to INPA/089): 1 juvenile, same data as holotype, except between the mining in road MG-129, 20°13′01.9″ S, 43°29′34.9″ W; 20°13′01.9″ S, 43°29′34.9″ W, 1071 m, 12.iii.2019.

*Description.* Total length (head + trunk) of specimens 0.87–1.09 mm (n = 4), holotype 1.09 mm.

Head. Ratio antennae:trunk = 1:2.23–2.8 (n = 4), holotype 1:2.8; Ant III smaller than Ant II length; Ant segment ratio as I: II: III: IV = 1: 1.65–1.75: 0.9–1.41: 2.5–3.12, holotype 1: 1.75: 0.9:2,5. Ant IV with numerous common sens dorsally and ventrally (b–c), ventrally with at least 2 proximal g sens, plus several ordinary chaetae (i–k); Ant III with common sens b–c, apical organ with 2 basally swollen apical sens clubs surrounded by at least 7 sens c (Figure 49A); Ant II with common sens b–c, with 1 dorso-distal f. Clypeal formula with 4 (l1–2), 2 (ft), 3 (pf0–1) ciliate chaetae, all apically acuminate, pf1 slightly thicker and shorter than pf0, l2 longer (Figure 49B). Prelabral chaetae ciliate and not bifurcate. Labral chaetae smooth, no modifications (Figure 49B). Labial papilla D with 4 appendages; papilla E with l.p. finger-shaped, sinuous, and surpassing the base of apical appendage (Figure 49C). Labial proximal chaetae smooth (lpc1, 3–4, and 6–7), lpc3 and lpc6 shorter and slightly thinner than others (Figure 49C). Maxillary palp with b.c. weakly ciliate and 1.13 longer than t.a. (Figure 49D, anterior sublobal minute appendage not represented). Head dorsal chaetotaxy (Figure 49E) with 10–11 “An” (An1a–3), 5 “A” (A0–3, A5, and A2a absent), 4 “M” (M1–4), 6 “S” (S0 and S2–6), 3 “Ps” (Ps2–3 and Ps5), 4 “Pa” (Pa2–5), 2 “Pm” (Pm1 and Pm3), 7 “Pp” (Pp1–7), and 2 “Pe” (Pe3 and 6) chaetae; An1a–3a, A0, A2–3, and M2 as mac; interocular p mic present; 1 extra mes anterior to A0 present or absent; head posterior region with at least 8 cervical spine-like mac (not represented in Figure 49E). Basomedian and basolateral labial fields with a1–5 smooth; M1–2, E, and L1–2 unilaterally ciliate; r reduced; M1 subequal to M2; and L2 slightly longer than others (Figure 49F). Ventral chaetotaxy with 16–17 ciliate chaetae, laterally 1 weakly ciliate thinner (X4) and 1 reduced lateral spine; postlabial formula 4 (G1–4), 2 (X and X4), 3 (H2–4), and 2 (J1–2) chaetae, 3 latero-posterior chaetae thicker, b.c. present (Figure 49F).

Thorax dorsal chaetotaxy (Figure 50A). Th II a, m, and p series with 1 (a5), 4 (m–5e?), and 6 (p1–6) mic, respectively. Th III a, m, and p series with 6 (a1–4 and a6–7), 4 (m2 and m4–6), and 6 (p1–6) mic, respectively. Ratio Th II:III = 2.36:1 in holotype.

Abdomen dorsal chaetotaxy (Figure 50B,C). Abd I a, m, and p series with 4 (a1–3 and a5), 4 (m2–3 and m5–6), and 2 (p5–6) mic, respectively, a3 as reduced mic. Abd II a, m, and p series with 3 (a2–3 and a6), 5 (m3–3e and m4–6), and 5 (p4–7) chaetae, respectively, m3 and m5 as mac; a5 and m2 bothriotricha surrounded by 2 (lm and ll) and 2 (ml and mi) fan-shaped chaetae, respectively. Abd III a, m, and p series with 4 (a2–3 and a6–7), 7 (m3–4, am6, pm6, and m7–8), and 5 (p3 and p5–8) chaetae, respectively, pm6 and p6 as mac, a3 and m3 as reduced mic, as sens elongated, ms absent; m2 bothriotrichum associated with 3 (a2, ml, and mi) and a5 and m5 bothriotricha with 7 (am6, a6, li, lm, ll, im, and em) fan-shaped chaetae. Abd IV A–Fe series with 5 (A2–6), 4 (B2 and B4–6), 1 (Be3), 5 (C1–4), 5 (T1, T3, and T5–7), 1 (Te7), 5 (D1–3p), 1–2 (De1 and De3), 5 (E1–4p), 3 (F1–3), and 4–5 (Fe1–5) chaetae, respectively, with 3 central (B5–6 and C1) and 5 lateral mac (D3, E2–4, and F1); T2 and T4 bothriotricha surrounded by 5 (D1, a, s, m, and C1p) and 2 (pe and pi) fan-shaped chaetae, respectively; 1 extra mes near to E2, 5 sens present, ps type I, as plus 3 anterior type II, r series laterally with 3 chaetae. Abd V a, m, and p series with 1 (a5), 3 (m2–3 and m5), and 3 (p3–5) central mac, respectively. Ratio Abd III:IV = 1:3.26 in holotype. Th II–Abd IV formula with 00|010 + 21 + 2 mac.

Legs. Subcoxa I with 6–7 chaetae and 2 psp; subcoxa II with “a” row of 4–5 chaetae, “p” row with 8–9 chaetae and 2 psp; subcoxa III with one row of 6–8 plus 2 anterior chaetae and 2 posterior psp (Figure 51A–C). Trochanteral organ with about 12 spine-like chaetae (Figure 51D). Tibiotarsus I–III formula with 1, 3, and 3 mac type III (Figure 51E). Unguis outer side with paired teeth straight and not developed on proximal one third; inner side with wide lamella and 3 teeth, basal pair unequal, b.p. tooth larger, but not reaching the m.t apex, m.t on distal one third and subequal to b.a., a.t. absent. Unguiculus with all lamellae and smooth and acuminate (ai, ae, pi, and pe), except ai gently truncate distally and pe serrate (Figure 51F); ratio unguis:unguiculus in holotype = 1.57:1. Tibiotarsal smooth chaeta about 0.86 shorter than unguiculus; tenent hair acuminate and about 0.64 smaller than unguis outer edge.

Collophore (Figure 51G). Anterior side with 5–7 ciliate apically acuminate chaetae, 1 subdistal thicker than others except the distal mac; posterior side distally with 1 smooth and 1 ciliate lateral chaeta, 1 subdistal smooth chaeta and 2 reduced spines (1 unpaired); lateral flap with 7–8 smooth (2 longer) and 1 ciliate chaeta.

Furcula. Only with ciliate chaetae and scales. Manubrium ventrally with 3 subapical and about 11 distal scales, plus 1 foot-shape chaetae distally; manubrial plate with 3–6 ciliate chaetae (2 inner mac) and 2 psp (Figure 51I,J). Mucro basal tooth gently larger than distal tooth, basal spine reaching the apex of basal tooth (Figure 51K).

*Etymology.* Refers to type locality of new species, “Mariana” municipality, where a mining disaster killed 18 people on 5 November 2015.

*Remarks. Pseudosinella marianensis* sp. nov. resembles *P. pusilla* sp. nov. in prelabral chaetae ciliate, labral chaetae smooth, basomedian and basolateral labial fields with a1–5 smooth; M1–2, E, and L1–2 unilaterally ciliate; and r reduced; Th II–Abd III with 0, 0, 0, 1, and 0 central mac, tenent hair acuminate, unguis with 3 inner teeth, and unguiculus ai lamella discretely truncate distally (Table 1 and Table 5). However, *P. marianensis* sp. nov. differs from this species in head M2 mac and Abd IV B6 mac present (both absent in *P. pusilla* sp. nov.), tibiotarsus I–III with mac type III (type VII in *P. pusilla* sp. nov.), unguiculus pe lamella serrate (smooth in *P. pusilla* sp. nov.), and collophore posteriorly with 2 spines (1 in *P. pusilla* sp. nov.). *Pseudosinella marianensis* sp. nov. also resembles *P. spurimarianensis* sp. nov. in prelabral, labral, and dorsal heads to Abd IV chaetotaxy, but *P. marianensis* sp. nov. differs from this species in basomedian and basolateral labial fields with M2, E, and L1–2 unilaterally ciliate (completely ciliate in *P. spurimarianensis* sp. nov.), tenent hair acuminate (capitate in *P. spurimarianensis* sp. nov.), unguis outer teeth on proximal one third (on distal half in *P. spurimarianensis* sp. nov.), and unguis a.t. absent (present in *P. spurimarianensis* sp. nov.). In addition, *P. marianensis* sp. nov. have 1 foot-shape chaeta on distal ventral manubrium not seen in any other species (Figure 51I).

#### 3.3.6. *Pseudosinella mitodentunguilata* sp. nov. Bellini, Cipola, and Souza

**Figure 52:**
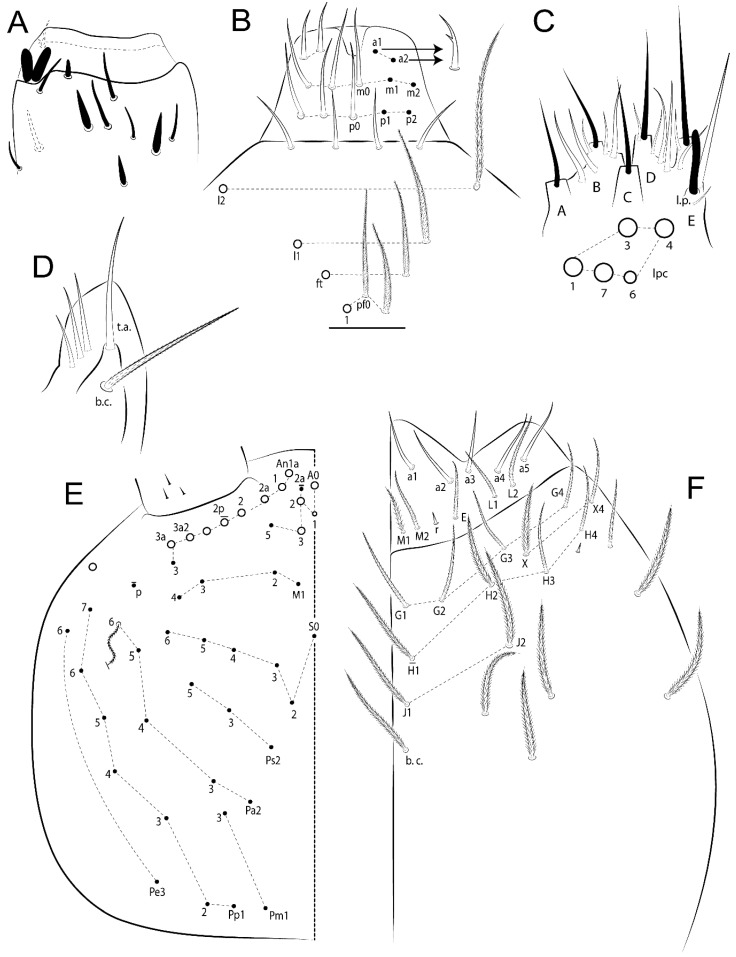
*Pseudosinella mitodentunguilata* sp. nov.: head. (**A**) Ant III apical organ (ventro-lateral view); (**B**) chaetotaxy of clypeus, prelabrum, and labrum; (**C**) labial papillae (H omitted) plus proximal chaetae (right side); (**D**) maxillary palp and sublobal plate (right side), minute appendage omitted; (**E**) head dorsal chaetotaxy (left side); (**F**) basomedian and basolateral labial fields and complete postlabial chaetotaxy (right side).

**Figure 53:**
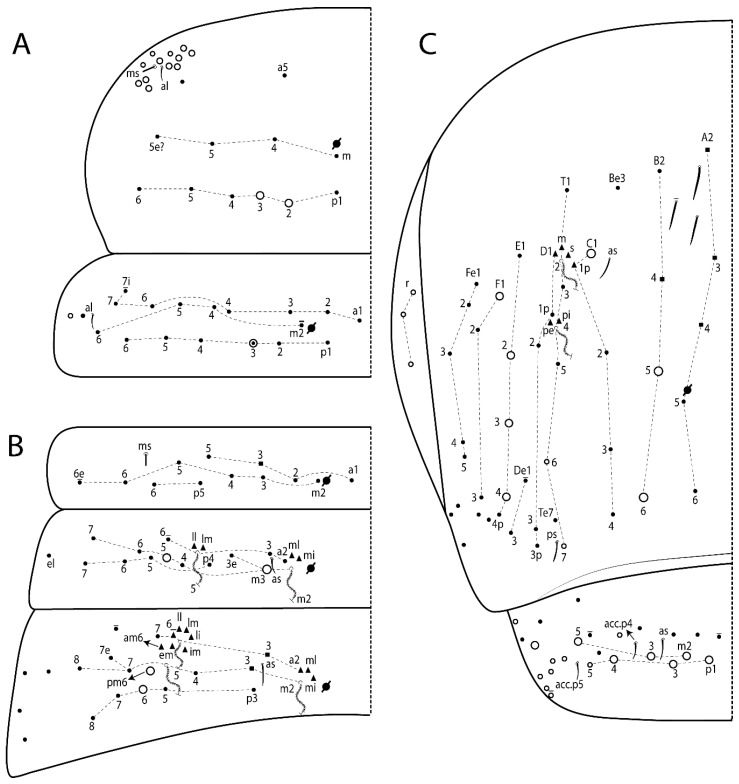
*Pseudosinellamitodentunguilata* sp. nov.: dorsal chaetotaxy. (**A**) Th II–III; (**B**) Abd I–III; (**C**) Abd IV–V.

**Figure 54:**
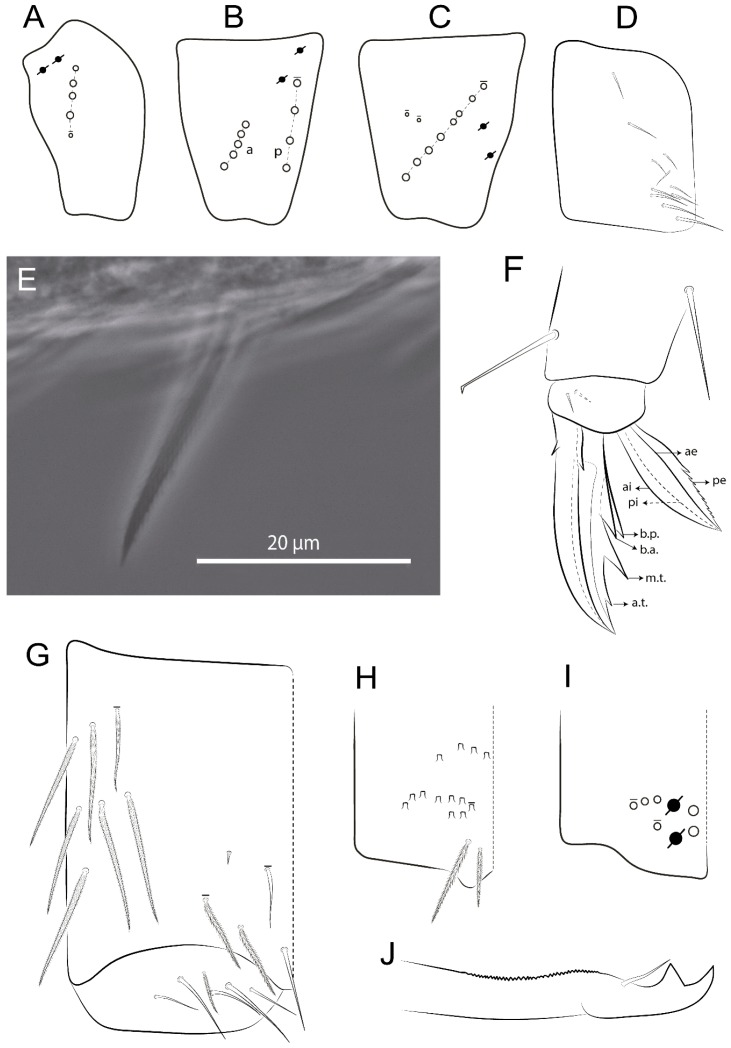
*Pseudosinella mitodentunguilata* sp. nov.: trunk appendages. (**A–C**) subcoxa I–III, respectively; (**D**) trochanteral organ; (**E**) modified chaeta of tibiotarsus III; (**F**) distal tibiotarsus and empodial complex III (anterior view); **(G**) collophore (lateral view); (**H**) distal part of manubrium (ventral view); (**I**) manubrial plate (dorsal view); (**J**) distal dens and mucro (inner view).

Figure 52, Figure 53 and Figure 54, Table 1 and Table 5

*Type material.* Holotype female in slide (6462/CRFS-UEPB): Brazil, Minas Gerais State, Luislândia municipality, “Lapa sem Fim” cave complex, 16°08′54.0″ S, 44°37′38.0″ W, 691 m, 10-27.iv.2015, Carste et al. coll. Paratypes in slides (6450–55, 6457–58, 6460–61, 6463,6472–73/CRFS-UEPB): 3 males, 5 females and 5 juveniles, same data as holotype. Paratypes in slides (6486–87/CRFS-UEPB donated to INPA/090): 2 females, same data as holotype. Paratypes in slides (6228–29, 6232–34/CRFS-UEPB): 2 males and 3 females, same data as holotype, except 07-24.x.2014.

*Description.* Total length (head + trunk) of specimens 0.97–1.23 mm (n = 4), holotype 1.23 mm.

Head. Ratio antennae:trunk = 1:2.07–2.56 (n = 4), holotype 1:2.07; Ant III smaller than Ant II length; Ant segment ratio as I:II: III:IV = 1:2.37–3:1.58–2.64:4.33–5.73, holotype 1:2.37:1.58:5. Ant IV with numerous common sens dorsally and ventrally (b–c), dorsal face with 1 f and 1 e sens apically and 2 e proximally; Ant III with common sens b–c, apical organ with 2 swollen apical sens clubs surrounded by at least 8 sens c and 4 e (Figure 52A), ventral face with 2 more e sens proximally; Ant II with common sens b–c, with 2 ventro-lateral sens e. Clypeal formula with 4 (l1–2), 2 (ft), and 3 (pf0–1) ciliate chaetae; all apically acuminate, pf1 shorter than pf0, and l2 longer (Figure 52B). Prelabral chaetae smooth and not bifurcate. Labral chaetae smooth, a1–2 thicker and sometimes with 1 median filament (Figure 52B). Labial papilla D with 4 appendages; papilla E with l.p. finger-shaped, straight, and surpassing the base of apical appendage (Figure 52C). Labial proximal chaetae smooth (lpc1, 3–4, and 6–7), lpc6 gently smaller and thinner than others (Figure 52C). Maxillary palp with b.c. weakly ciliate and 1.24 longer than t.a. (Figure 52D, anterior sublobal minute appendage not represented). Head dorsal chaetotaxy (Figure 52E) with 8–9 “An” (An1a–3), 5–6 “A” (A0–3, A5, and A2a present or absent), 4 “M” (M1–4), 6 “S” (S0 and S2–6), 3 “Ps” (Ps2–3 and Ps5), 4 “Pa” (Pa2–5), 2 “Pm” (Pm1 and Pm3), 7 “Pp” (Pp1–7), and 2 “Pe” (Pe3 and 6) chaetae; An1a–3a, A0, and A2–3 as mac; interocular p mic present or absent; head posterior region with at least 8 cervical spine-like mac (not represented in Figure 52E). Basomedian and basolateral labial fields with a1–5 smooth; M2, E, and L1–2 weakly ciliate; M1 ciliate; r reduced; M1 smaller than M2; and L2 slightly longer than others (Figure 52F). Ventral chaetotaxy with 8 weakly ciliate anteriorly, 10–11 ciliate chaetae and laterally with 1 reduced spine; postlabial formula 4 (G1–4), 2 (X and X4), 3–4 (H1–4), and 2 (J1–2) chaetae, 6 latero-posterior chaetae thicker (including J2), b.c. present (Figure 52F).

Thorax dorsal chaetotaxy (Figure 53A). Th II a, m, and p series with 1 (a5), 4 (m–5e?), and 6 (p1–6) chaetae, respectively, p2–3 as mac. Th III a, m, and p series with 6–7 (a1–4 and a6–7i), 3–4 (m2 and m4–6), and 6 (p1–6) chaetae, respectively, p3 mostly as mac (as mic asymmetry in one specimen). Ratio Th II:III = 2.14:1 in holotype.

Abdomen dorsal chaetotaxy (Figure 53B,C). Abd I a, m, and p series with 4 (a1–3 and a5), 5–6 (m2–6e), and 2 (p5–6) mic, respectively, a3 as reduced mic. Abd II a, m, and p series with 2–3 (a2–3 and a6), 6 (m3–3e and m4–7), and 4 (p4–7) chaetae, respectively, m3 and m5 as mac; a5 and m2 bothriotricha surrounded by 2 (lm and ll) and 2 (ml and mi) fan-shaped chaetae, respectively. Abd III a, m, and p series with 3–4 (a2–3 and a6–7), 7 (m3–4, am6, pm6, and m7–8), and 5 (p3 and p5–8) chaetae, respectively, pm6 and p6 as mac, a3 and m3 as reduced mic, as sens elongated, ms absent; m2 bothriotrichum associated with 3 (a2, ml, and mi) and a5 and m5 bothriotricha with 6–7 (am6, a6, li, lm, ll, im, and em) fan-shaped chaetae. Abd IV A–Fe series with 5 (A2–6), 4 (B2 and B4–6), 1 (Be3), 5 (C1–4), 5 (T1, T3, and T5–7), 1 (Te7), 5 (D1–3p), 1–2 (De1 and De3), 5 (E1–4p), 3 (F1–3), and 5 (Fe1–5) chaetae, respectively, with 3 central (B5–6 and C1) and 4 lateral mac (E2–4 and F1); T2 and T4 bothriotricha surrounded by 4 (D1, s, m, and C1p) and 2 (pe and pi) fan-shaped chaetae, respectively; A2–4 and B4 as reduced mic; 4–5 sens present, ps type I, as plus 2–3 anterior type II, r series laterally with 3 mes. Abd V m and p series with 3 (m2–3 and m5) and 3 (p1 and p3–4) central mac, respectively. Ratio Abd III:IV = 1:3.07 in holotype. Th II–Abd IV formula with 21(0)|010 + 21 + 2 mac.

Legs. Subcoxa I with 4–5 chaetae and 2 psp; subcoxa II with “a” row of 5 chaetae, “p” row with 3–4 chaetae and 2 psp; subcoxa III with one row of 7–8 plus 0–2 anterior chaetae and 2 posterior psp (Figure 54A–C). Trochanteral organ with about 11 spine-like chaetae (Figure 54D). Tibiotarsus I–III formula with 1, 3, and 3 mac type IV (Figure 54E). Unguis outer side with paired teeth straight and not developed on proximal one fourth; inner side with wide lamella and 3–4 teeth, basal pair (b.a. and b.p.) apparently subequal and not reaching the m.t. apex, m.t. on little more than distal half, narrow and larger basal teeth, a.t. minute usually present on distal one fifth (absent in few specimens). Unguiculus with all lamellae acuminate and smooth (ai, ae, pi, and pe), except pe serrate (Figure 54F); ratio unguis:unguiculus in holotype = 1.67:1. Tibiotarsal smooth chaeta subequal to unguiculus lenght; tenent hair capitate and about 0.6 smaller than unguis outer edge.

Collophore (Figure 54G). Anterior side with 6–7 ciliate apically acuminate chaetae, 0–1 latero-proximal thinner and shorter, and others subequal; posterior side distally with 1–2 smooth and 1–2 ciliate lateral chaetae plus 1 reduced spine; lateral flap with 6 smooth (2 longer) and 1 ciliate chaeta.

Furcula. Only with ciliate chaetae and scales. Manubrium ventrally with 4 subapical and about 8–9 distal scales; manubrial plate with 4–6 ciliate chaetae (2 inner mac) and 2 psp (Figure 54H and I). Mucro basal tooth gently larger than distal tooth, basal spine reaching the apex of basal tooth (Figure 54J).

*Etymology.* Refers to shape of its unguis median tooth (from Greek: mito—thread, filament; from Latin: dent-tooth) (Figure 51F).

*Remarks. Pseudosinella mitodentunguilata* sp. nov. resembles *P. erehwon* Christiansen and Bellinger, 1996 and *P. extra* Christiansen and Bellinger, 1996 by prelabral and labral chaetae smooth, head with A0 and A2–3 mac and M2 mic, Th II–Abd III with 2, 0–1, 0, 1, and 0 central mac, and unguiculus outer lamellae serrate (Table 4 and Table 5). However, the new species differs in head with M1 mic (mac in *P. extra*), labral a1–2 chaetae with 1 median filament (smooth in *P. erehwon* and *P. extra*), basomedian labial field with r reduced and smooth (long and ciliate in *P. erehwon* and *P. extra*), Abd IV with C1 mac (as mic in *P. erehwon* and *P. extra*), tenent hairs capitate (acuminate in *P. extra*), and unguis m.t. larger than basal teeth (smaller in *P. erehwon* and *P. extra*) [6]. This last feature also was reported to *P. cassagnaui* Gisin and Gama, 1970 from France [56] (p. 183, Figure 15), but the new species clearly differs from it by eyes absent (1 in *P. cassagnaui*), Th II with 2 mac (absent in *P. cassagnaui*), tenent hairs capitate (acuminate in *P. cassagnaui*), and unguis a.t. present (absent in *P. cassagnaui*).

#### 3.3.7. *Pseudosinella neriae* sp. nov. Bellini, Cipola, and Souza

**Figure 55:**
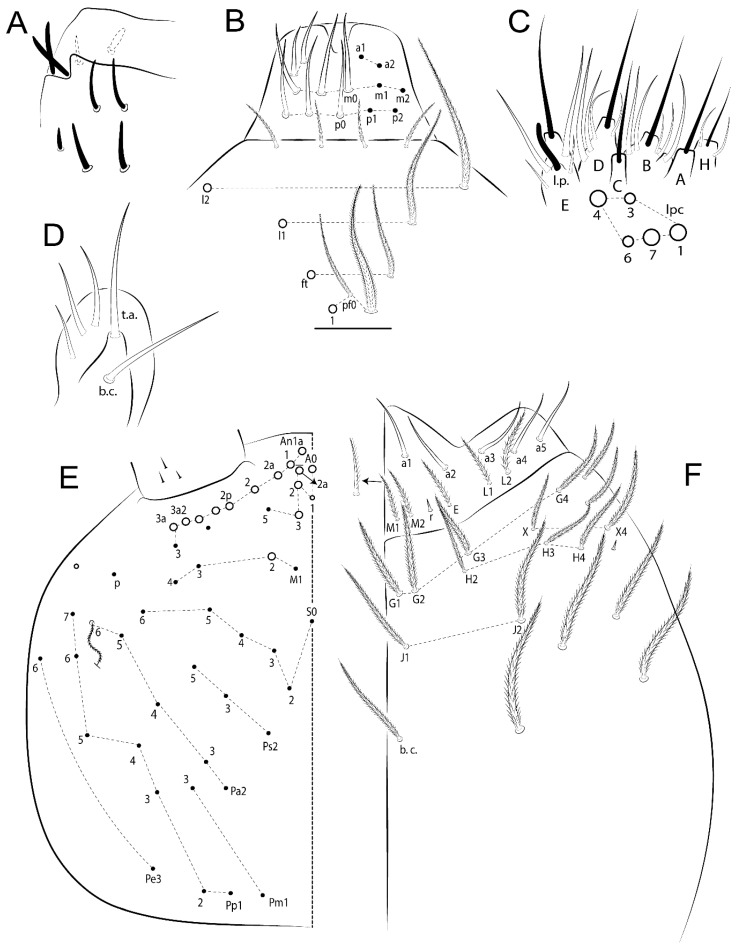
*Pseudosinella neriae* sp. nov.: head. (**A**) Ant III apical organ (ventro-lateral view); (**B**) chaetotaxy of clypeus, prelabrum, and labrum; (**C**) labial papillae plus proximal chaetae (left side); (**D**) maxillary palp and sublobal plate (right side), minute appendage omitted; (**E**) head dorsal chaetotaxy (left side); (**F**) basomedian and basolateral labial fields and complete postlabial chaetotaxy (right side).

**Figure 56:**
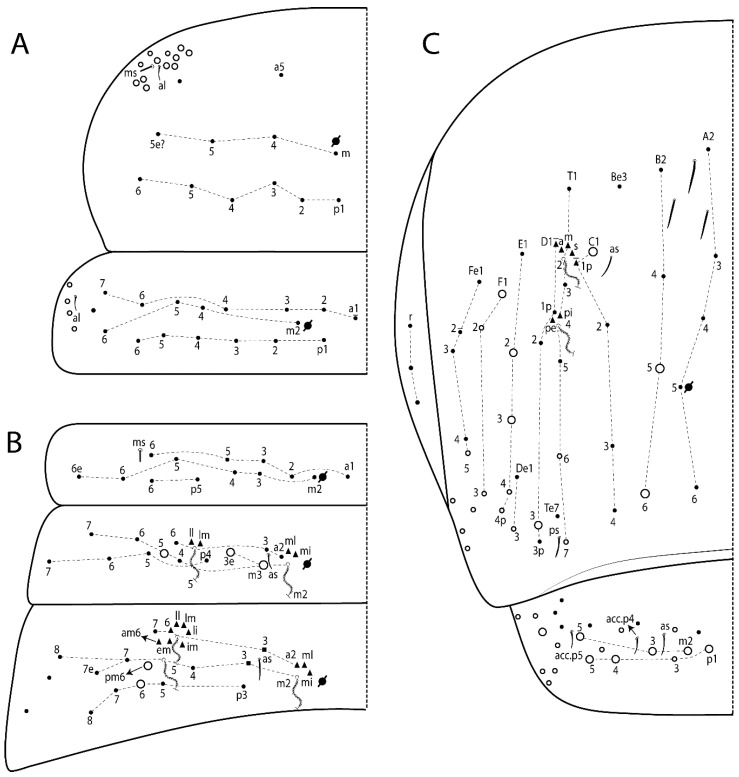
*Pseudosinella neriae* sp. nov.: dorsal chaetotaxy. (**A**) Th II–III; (**B**) Abd I–III; (**C**) Abd IV–V.

**Figure 57:**
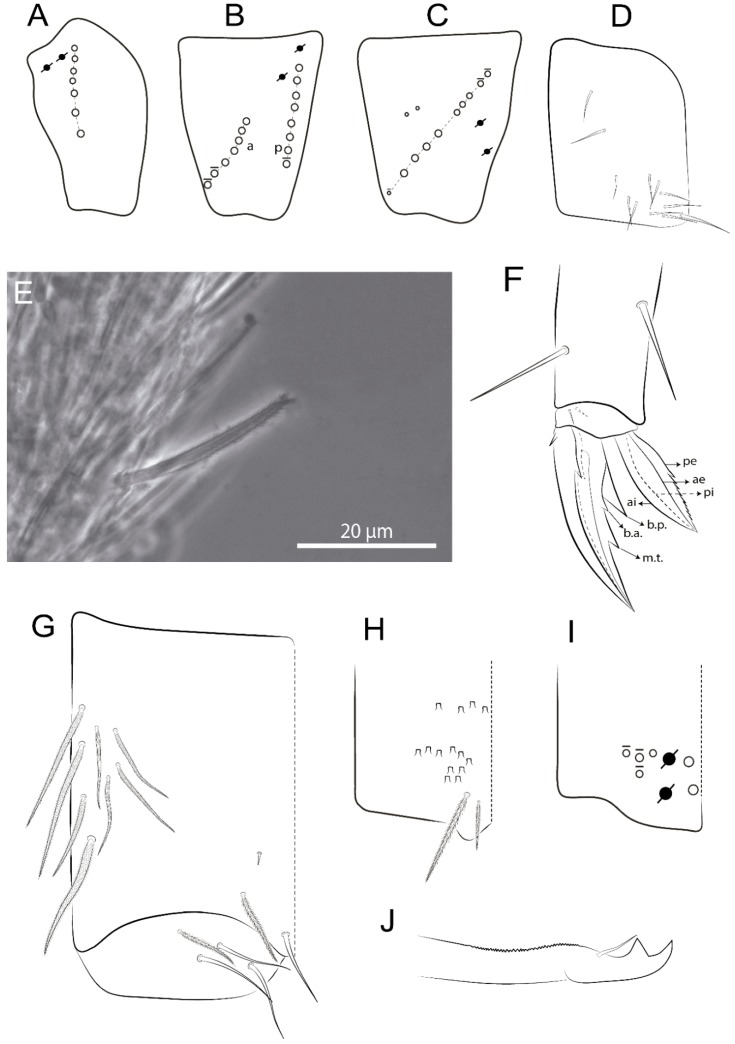
*Pseudosinella neriae* sp. nov.: trunk appendages. (**A–C**) subcoxa I–III, respectively; (**D**) trochanteral organ; (**E**) modified chaeta of tibiotarsus II; (**F**) distal tibiotarsus and empodial complex III (anterior view); (**G**) collophore (lateral view); (**H**) distal part of manubrium (ventral view); (**I**) manubrial plate (dorsal view); (**J**) distal dens and mucro (inner view).

Figure 55, Figure 56 and Figure 57, Table 1 and Table 5

*Type material.* Holotype female in slide (7402/CRFS-UEPB): Brazil, Minas Gerais State, Pedro Leopoldo municipality, Santa Maria neighborhood, next to the mining of Santo Antônio Lake, 19°34′30.2″ S, 44°00′27.1″ W, 806 m, 31.i.2016, L.R. Vieira coll. Paratypes in slides (6529, 6531/CRFS-UEPB): 2 females, same data as holotype, except 04–20.iii.2015, Carste et al. coll. Paratype in slide (6501/CRFS-UEPB donated to INPA/091): 1 female, same data as holotype, except 04–20.iii.2015, Carste et al. coll.

*Description.* Total length (head + trunk) of specimens 1.02–1.22 mm (n = 3), holotype 1.22 mm.

Head. Ratio antennae:trunk = 1:2.41–2.61 (n = 3), holotype 1:2.61; Ant III smaller than Ant II length; Ant segment ratio as I:II:III:IV = 1:1.67–2.67:1.39–2.5:2.89–5.33, holotype 1:2.06:1.78:3.5. Ant IV with numerous common sens dorsally and ventrally (b–c), ventrally with at least 3 proximal e sens, plus several ordinary chaetae (i–k); Ant III with common sens b–c, apical organ with 2 apical sens clubs surrounded by at least 5 sens c, and 1 e and 1 f (Figure 55A); Ant II with common sens b–c, with 2 dorso-distal f, 1 dorso-lateral h and at least 2 ventro-subdistal g sens. Clypeal formula with 4 (l1–2), 2 (ft), and 3 (pf0–1) ciliate chaetae, all apically acuminate, pf1 thicker, and l2 longer (Figure 55B). Prelabral chaetae weakly ciliate and not bifurcate. Labral chaetae smooth, no modifications (Figure 55B). Labial papilla D with 4 appendages; papilla E with l.p. finger-shaped, sinuous, and surpassing the base of apical appendage (Figure 55C). Labial proximal chaetae smooth (lpc1, 3–4, and 6–7), lpc3 and lpc6 shorter and slightly thinner than others (Figure 55C). Maxillary palp with b.c. apparently smooth and 0.93 shorter than t.a. (Figure 55D, anterior sublobal minute appendage not represented). Head dorsal chaetotaxy (Figure 55E) with 10 “An” (An1a–3), 5–6 “A” (A0–3, A5, and A2a present or absent), 4 “M” (M1–4), 6 “S” (S0 and S2–6), 3 “Ps” (Ps2–3 and Ps5), 4 “Pa” (Pa2–5), 2 “Pm” (Pm1 and Pm3), 7 “Pp” (Pp1–7), and 2 “Pe” (Pe3 and 6) chaetae; An1a–3a, A0, A2a–3, and M2 as mac; interocular p mic present; head posterior region with at least 9 cervical spine-like mac (not represented in Figure 55E). Basomedian and basolateral labial fields with a1–5 smooth; M1–2, E, and L1–2 uniformly to unilaterally ciliate; r reduced; M1 subequal to M2; and L2 slightly longer than others (Figure 55F). Ventral chaetotaxy with 18 ciliate chaetae plus 1 reduced lateral spine; postlabial formula 4 (G1–4), 2 (X and X4), 3 (H2–4), and 2 (J1–2) chaetae, H2 thinner than others, 5 latero-posterior chaetae thicker, b.c. present (Figure 55F).

Thorax dorsal chaetotaxy (Figure 56A). Th II a, m, and p series with 1 (a5), 4 (m–5e?), and 6 (p1–6) mic, respectively. Th III a, m, and p series with 6 (a1–4 and a6–7), 4 (m2 and m4–6), and 6 (p1–6) mic, respectively. Ratio Th II:III = 1.87:1 in holotype.

Abdomen dorsal chaetotaxy (Figure 56B,C). Abd I a, m, and p series with 5 (a1–3 and a5–6), 6 (m2–6e), and 2 (p5–6) mic, respectively, a3 not remarkably smaller than others. Abd II a, m, and p series with 2–3 (a2–3 and a6), 6 (m3–3e and m4–7), and 4 (p4–7) chaetae, respectively, m3–3e and m5 as mac; a5 and m2 bothriotricha surrounded by 2 (lm and ll) and 2 (ml and mi) fan-shaped chaetae, respectively. Abd III a, m and p series with 4 (a2–3 and a6–7), 7 (m3–4, am6, pm6, and m7–8), and 5 (p3 and p5–8) chaetae, respectively, pm6 and p6 as mac, a3 and m3 as reduced mic, as sens elongated, ms absent; m2 bothriotrichum associated with 3 (a2, ml, and mi) and a5 and m5 bothriotricha with 6 (am6, li, lm, ll, im, and em) fan-shaped chaetae. Abd IV A–Fe series with 5 (A2–6), 4 (B2 and B4–6), 1 (Be3), 4–5 (C1–4), 5 (T1, T3, and T5–7), 1 (Te7), 4–5 (D1–3p), 2 (De1 and De3), 5 (E1–4p), 3 (F1–3), and 4–5 (Fe1–5) chaetae, respectively, with 3 central (B5–6 and C1) and 4 lateral mac (D3, E2–3, and F1); T2 and T4 bothriotricha surrounded by 3–5 (D1, a, s, m, and C1p) and 2 (pe and pi) fan-shaped chaetae, respectively; 5 sens present, ps type I, as plus 3 anterior type II, r series laterally with 3 chaetae. Abd V m and p series with 3 (m2–3 and m5) and 3 (p1 and p4–5) central mac, respectively. Ratio Abd III:IV = 1:3.28 in holotype. Th II–Abd IV formula with 00|020 + 21 + 2 mac.

Legs. Subcoxa I with 7 chaetae and 2 psp; subcoxa II with “a” row of 5–7 chaetae, “p” row with 7–8 chaetae and 2 psp; subcoxa III with one row of 7–10 plus 2 anterior chaetae and 2 posterior psp (Figure 57A–C). Trochanteral organ with about 12 spine-like chaetae (Figure 57D). Tibiotarsus I–III formula with 1, 3, and 3 mac type IV (Figure 57E). Unguis outer side with paired teeth straight and not developed on proximal one fourth; inner side with wide lamella and 3 teeth, basal pair unequal, b.p. tooth larger, but not reaching the m.t. apex, m.t. on distal one third and larger than b.a., a.t. absent. Unguiculus with all lamellae smooth and acuminate (ai, ae, pi, and pe), except pe serrate (Figure 57F); ratio unguis:unguiculus in holotype = 1.53:1. Tibiotarsal smooth chaeta about 0.88 shorter than unguiculus; tenent hair acuminate and about 0.65 smaller than unguis outer edge.

Collophore (Figure 57G). Anterior side with 8 ciliate apically acuminate chaetae, 3 internal larger than lateral chaetae plus 1 distal mac; posterior side distally with 1 smooth chaeta, 1 ciliate chaeta and 1 subdistal reduced spine; lateral flap with 3 smooth (subequal) and 1 ciliate chaeta.

Furcula. Only with ciliate chaetae and scales. Manubrium ventrally with 4 subapical and about 10 distal scales; manubrial plate with 3–6 ciliate chaetae (2 inner mac) and 2 psp (Figure 57H,I). Mucro basal tooth gently larger than distal tooth, basal spine reaching the apex of basal tooth (Figure 57J).

*Etymology.* The species was named after the nickname of our dear friend, Collembola researcher Dr. Nerivânia Nunes Godeiro.

*Remarks. Pseudosinella neriae* sp. nov. resembles *P. argentea* Folsom, 1902 by prelabral chaetae ciliate and labral smooth, basomedian and basolateral labial fields with a1–5 smooth; M2, E, and L1–2 ciliate; and r reduced, Th II–Abd I devoid of mac, unguis with 3 inner teeth with b.p. larger than the others, and unguiculus pe lamella serrate (Table 4 and Table 5). However, *P. neriae* sp. nov. differs from this species by head M2 mac, Abd II m3e mac, and Abd IV C1 mac present (all absent in *P. argentea*); tenent hair acuminate (capitate in *P. argentea*); tibiotarsus with mac type IV (apparently type VI in *P. argentea*); and collophore posteriorly with 1 spine, 1 smooth, and 1 ciliate chaetae, while in *P. argentea* with 10 posterior chaetae [7,20,53]. The presence of m3e mac on Abd II of *P. neriae* sp. nov. is shared with *P. ambigua* [14] and *P. folsomi* (Mills, 1931) [7,55], but the new species differs from these species by prelabral chaetae smooth and undivided (ciliate and bifurcate in *P. ambigua*), labral p0–2 chaetae smooth (ciliate in *P. ambigua*), head A3 mac present (absent in *P. folsomi*), Abd II a2 mac absent (present in *P. folsomi*), unguis with basal pair unequal and a.t. absent (basal teeth equal and a.t. present in *P. ambigua*), and tenent hair acuminate (capitate in the other two species).

#### 3.3.8. *Pseudosinella pusilla* sp. nov. Oliveira, Brito, and Cipola

**Figure 58:**
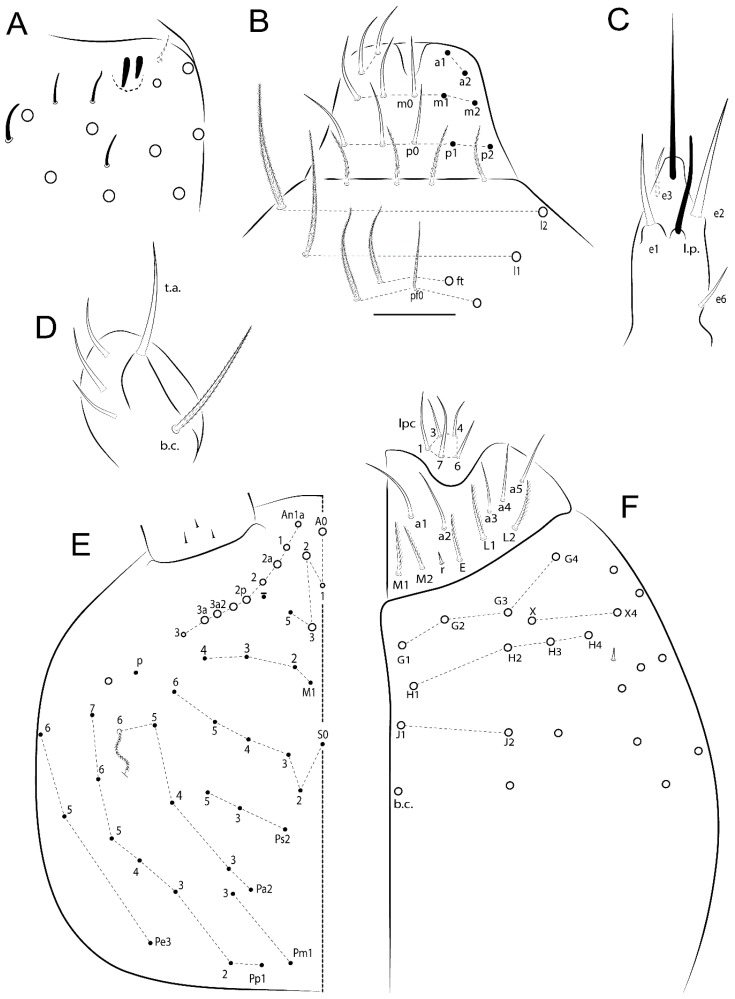
*Pseudosinella pusilla* sp. nov.: head. (**A**) Ant III apical organ (lateral view); (**B**) chaetotaxy of clypeus, prelabrum, and labrum; (**C**) labial papilla E (right side); (**D**) maxillary palp and sublobal plate (left side); (**E**) head dorsal chaetotaxy (left side); (**F**) labial proximal chaetae, basomedian and basolateral labial fields, and complete postlabial chaetotaxy (right side).

**Figure 59:**
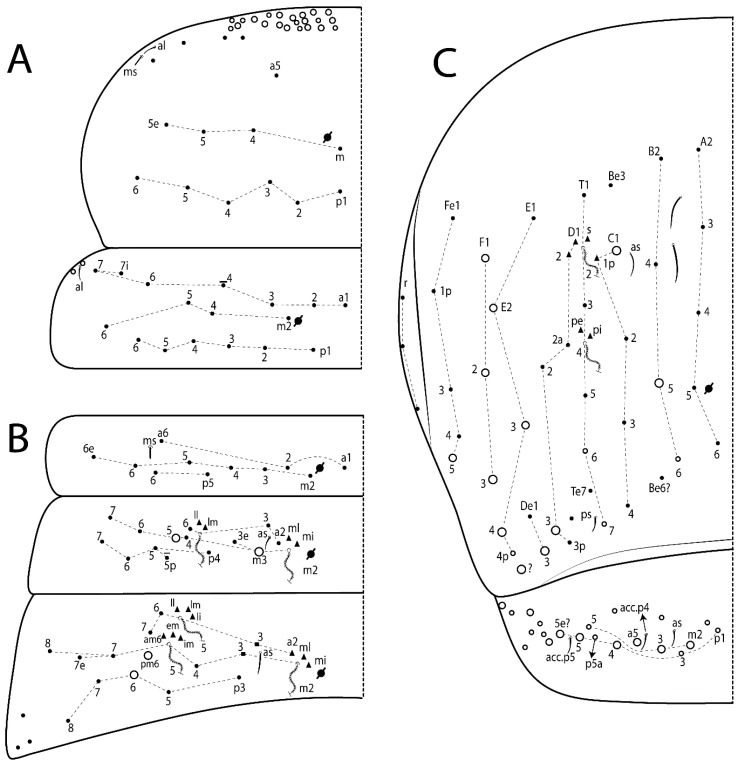
*Pseudosinella pusilla* sp. nov.: dorsal chaetotaxy. (**A**) Th II–III; (**B**) Abd I–III; (**C**) Abd IV–V.

**Figure 60:**
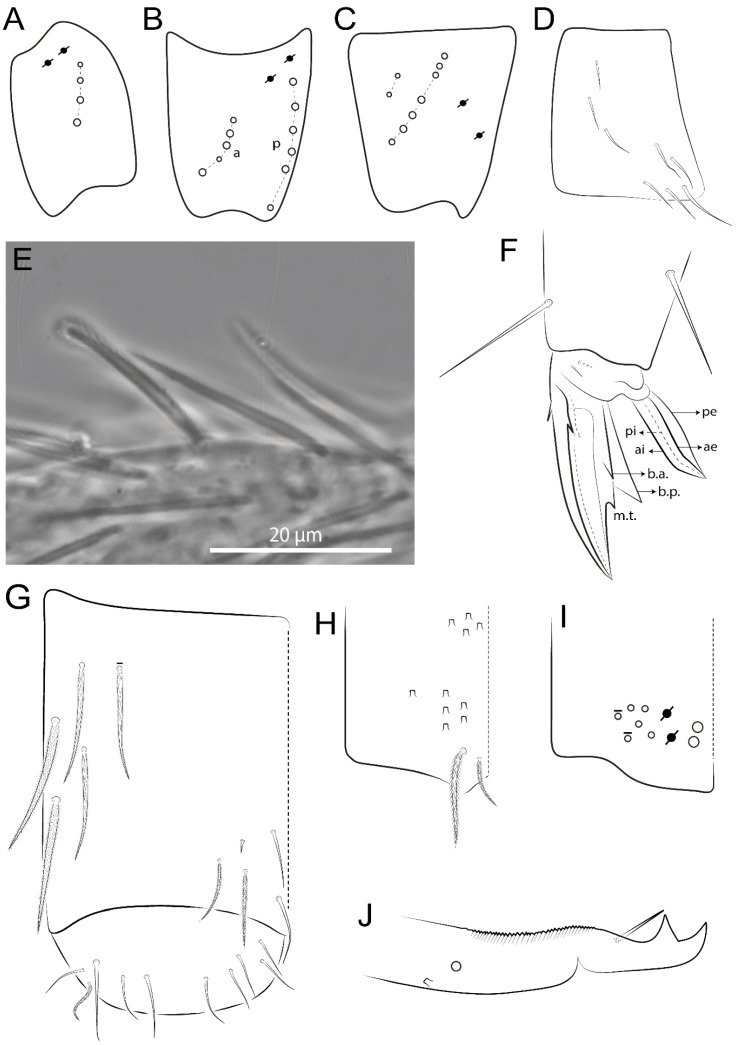
*Pseudosinella pusilla* sp. nov. (**A–C**) subcoxa I–III, respectively; (**D**) trochanteral organ; (**E**) modified chaeta of tibiotarsus; (**F**) distal tibiotarsus and empodial complex III (anterior view); (**G**) collophore (lateral view); (**H**) distal part of manubrium (ventral view); (**I**) manubrial plate (dorsal view); (**J**) distal dens and mucro (outer view).

Figure 58, Figure 59 and Figure 60, Table 1 and Table 5

*Type material.* Holotype female in slide (11497/CRFS-UEPB): Brazil, Pará State, Curionopolis municipality, next to “Serra Leste”, 06°00′31.6″ S, 49°38′06.3″ W, 527 m, 13-29.i.2015, Spelayon et al. coll. Paratypes in slides (8236–37/CRFS-UEPB): 1 male and 1 female, same data as holotype, except 05°59′10.1″ S, 49°37′12.3″ W, 559 m, 02-15.ii.2016. Paratype in slide (6924/CRFS-UEPB donated to INPA/092): 1 female, Parauapebas municipality, near mining area in the Carajás district, 06°01′16.5″ S, 50°16′49.3″ W, 439 m, 24.ii-13.iii.2015. Spelayon et al. coll.

*Description.* Total length (head + trunk) of specimens 0.66–0.89 mm (n = 4), holotype 0.77 mm.

Head. Ratio antennae:trunk = 1:3.13–3.93 (n = 4), holotype 1:3.28; Ant III smaller than Ant II length; Ant segment ratio as I:II:III:IV = 1:1.26–2.31:1.21–1.52:2.88–3.14, holotype 1:1.35:1.52:2.88. Ant IV dorsally with numerous sens (b–c) and chaetae (i–k). Ant III with 2 apical sens clubs surrounded by at least 5 sens c (Figure 58A). Ant II with common sens b–c, 1 c and 1 d dorso-distal, 1 d dorso-lateral, and 4 c and 1 d ventro-subdistal. Clypeal formula with 4 (l1–2), 2 (ft), and 3 (pf0–1) ciliate chaetae, all apically acuminate, l1–2 larger (Figure 58B). Prelabral chaetae ciliate and not bifurcate. Labral chaetae smooth, no modifications (Figure 58B). Labial papilla D with 3 appendages; papilla E with l.p. finger-shape, curved, and exceeds the base of apical appendage (Figure 58C). Maxillary palp with b.c. weakly ciliate and 1.28 longer than the t.a. (Figure 58D). Dorsal head chaetotaxy (Figure 58E) with 9 “An” (An1a–3), 5 “A” (A0–3 and A5), 4 “M” (M1–4), 6 “S” (S0 and S2–6), 3 “Ps” (Ps2–3 and Ps5), 4 “Pa” (Pa2–5), 2 “Pm” (Pm1 and Pm3), 7 “Pp” (Pp1–7), and 3 “Pe” (Pe3 and Pe5–6) chaetae; An1a–3, A0, and A2–A3 as mac; interocular p mic present; head posterior region with 8 cervical like-spine mac (not represented inf Figure 58E). Basomedian and basolateral labial fields with a1–5 smooth; M1–2, E, and L1–2 unilaterally ciliate; r reduced (Figure 58F). Labial proximal chaetae smooth (lpc1, 3–4, and 6–7), lpc3–4 and lpc6 gently smaller (Figure 58F). Ventral chaetotaxy with about 23 ciliate chaetae and 1 reduced lateral spine, postlabial formula 4 (G1–4), 2 (X and X4), 4 (H1–4), and 2 (J1–2) ciliate chaetae, b.c. present (Figure 58F).

Thorax dorsal chaetotaxy (Figure 59A). Th II a, m, and p series with 1 (a5), 4 (m and m4–5e?), and 6 (p1–6) mic, respectively. Th III a, m, and p series with 6–7 (a1–4 and a6–7i), 4 (m2 and m4–6), and 6 (p1–6) mic, respectively. Ratio Th II:III = 1.38–1.77:1 (n = 4), holotype 1.69:1.

Abdomen dorsal chaetotaxy (Figure 59B,C). Abd I a, m, and p series with 3 (a1–2 and a6), 6 (m2–6e), and 2 (p5–6) mic, respectively. Abd II a, m, and p series with 3 (a2–3 and a6), 6 (m3–7), and 4–5 (p4–7) chaetae, respectively, m3 and m5 as mac; a5 and m2 bothriotricha with 2 (ll and lm) and 2 (ml and mi) fan-shaped chaetae, respectively. Abd III a, m, and p series with 4 (a2–3 and a6–7), 7 (m3–4, am6, pm6, and m7–8), and 5 (p3 and p5–8) chaetae, respectively, pm6 and p6 as mac, a3 and m3 as reduced mic, and as sens elongated, ms absent; m2 bothriotrichum associated with 3 (a2, ml, and mi) and a5 and m5 bothriotricha with 6 (ll, lm, li, am6, em, and im) fan-shaped chaetae. Abd IV A–Fe series with 5 (A2–6), 4 (B2 and B4–6), 2 (Be3 and Be6?), 5 (C1–4), 5 (T1, T3, and T5–7), 1 (Te7), 6 (D1–3p), 2 (De1 and De3), 5 (E1–4p), 3 (F1–3), and 5 (Fe1–5) chaetae, respectively, with 2 central (B5 and C1) and 9 lateral mac (D3, De3, E2–E4, F1–3, Fe5, plus 1 unnamed); T2 and T4 bothriotricha with 4 (D1–1p, s, and C1p) and 2 (pe and pi) fan-shaped chaetae, respectively; 4 sens present, ps type I, as and 2 of uncertain homology type II, r series laterally with 3 chaetae. Abd V a, m, and p series with 1 (a5) 2 (m2–3) and 3 (p4–5e?) central mac, respectively. Ratio Abd III:IV = 1:2.99–3.22 (n = 3), holotype 1:3.22. Th II–Abd IV formula with 00|010 + 21 + 1 mac.

Legs. Subcoxa I with “p” row with 4 chaetae and 2 psp; subcoxa II with “a” row with 5 chaetae, “p” row with 6 chaetae and 2 psp; subcoxa III with one row of 7 “p” plus 2 anterior chaetae and 2 psp (Figure 60A–C). Trochanteral organ with about 8 spine-like chaetae (Figure 60D). Tibiotarsus I–III formula with 1, 3, and 3 mac type VII (Figure 60E). Unguis outer side with paired teeth straight and not developed on proximal one fourth; inner side with wide lamella and 3 teeth, basal pair (b.a. and b.p.) unequal, b.p. exceed gently the m.t. apex, m.t. on little more than distal half and subequal to b.a. length, a.t. absent. Unguiculus with all lamella smooth and acuminate (ai, ae, pi, and pe), except pe truncate distally (Figure 60F); ratio unguis:unguiculus in holotype = 1:0.51. Tibiotarsal smooth chaeta about 0.91 smaller than unguiculus; tenent hair acuminate and about 0.7 smaller than unguis outer edge.

Collophore (Figure 60G). Anterior side with 4–5 ciliate apically acuminate chaetae, 2 inner larger, and others subequal in length; posterior side distally with 2 ciliate and 2 smooth chaetae and 1 subdistal reduced spine; lateral flap with 9 smooth (2 larger) and 1 ciliate chaetae.

Furcula. Only with ciliate chaetae and scales. Manubrium ventrally with 4 subapical and 6 distal scales; manubrial plate with 6–8 ciliate chaetae (2 inner mac) and 2 psp (Figure 60 H,I). Mucro basal tooth subequal in size, basal spine reaching the apex of basal tooth (Figure 60J).

*Etymology.* Refers to small size of this new specie (from Latin: pusill-very small).

*Remarks. Pseudosinella pusilla* sp. nov. resembles *P. marianensis* sp. nov., but *P. pusilla* sp. nov. differ from this species by head M2 mac and Abd IV B6 mac absent (both present in *P. marianensis* sp. nov.), tibiotarsus I–III with mac type VII (type III in *P. marianensis* sp. nov.), unguiculus pe lamella smooth (serrate in *P. marianensis* sp. nov.), and collophore posteriorly with 1 spine (2 in *P. marianensis* sp. nov.) (Table 1 and Table 5). See also the comparison these species in remarks of *P. marianensis* sp. nov. and Table 1 and Table 5.

#### 3.3.9. *Pseudosinella spurimarianensis* sp. nov. Bellini, Cipola, and Souza

**Figure 61:**
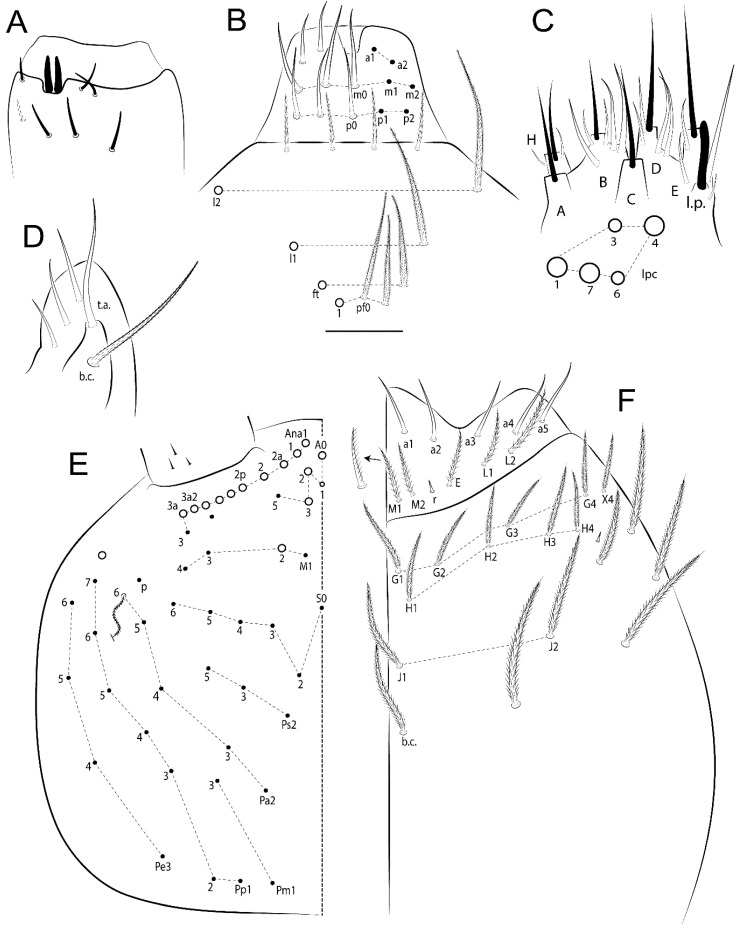
*Pseudosinella spurimarianensis* sp. nov.: head. (**A**) Ant III apical organ (ventro-lateral view); (**B**) chaetotaxy of clypeus, prelabrum, and labrum; (**C**) labial papillae plus proximal chaetae (right side); (**D**) maxillary palp and sublobal plate (right side), minute appendage omitted; (**E**) head dorsal chaetotaxy (left side); (**F**) basomedian and basolateral labial fields and complete postlabial chaetotaxy (right side).

**Figure 62:**
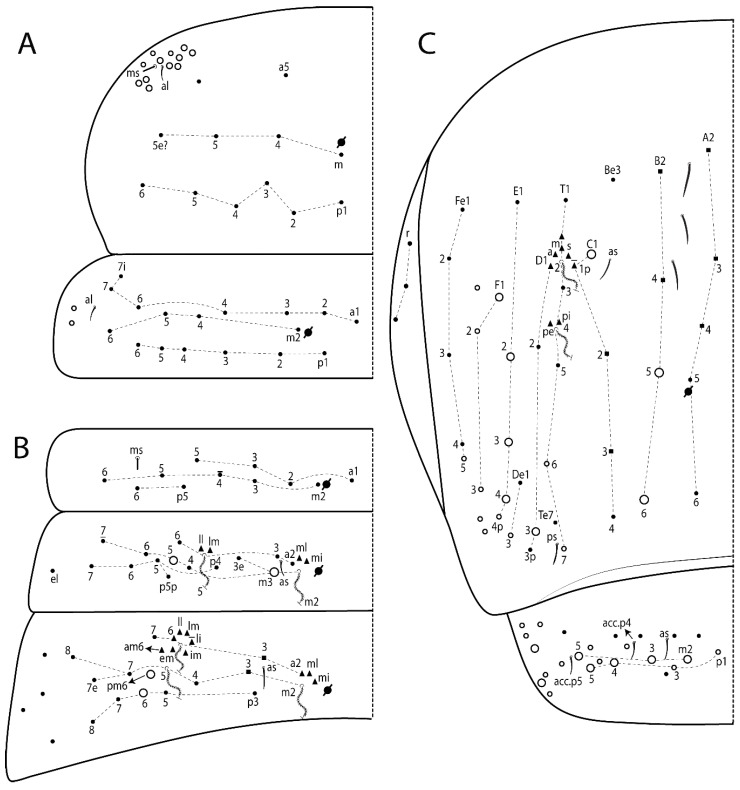
*Pseudosinella spurimarianensis* sp. nov.: dorsal chaetotaxy. (**A**) Th II–III; (**B**) Abd I–III; (**C**) Abd IV–V.

**Figure 63:**
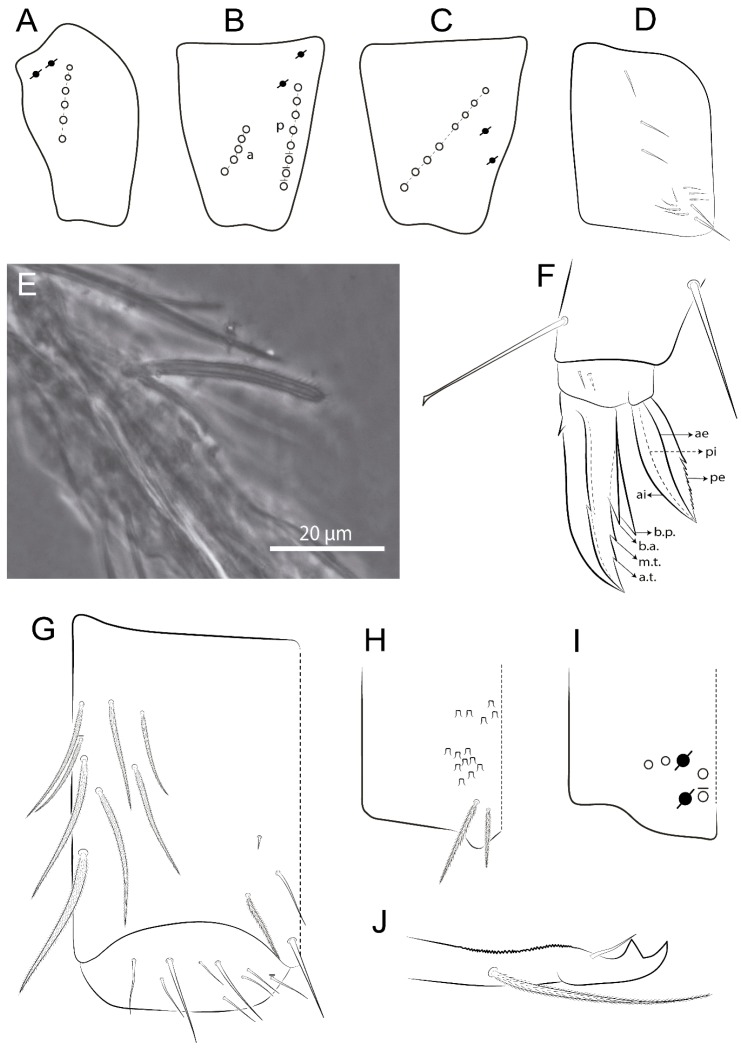
*Pseudosinella spurimarianensis* sp. nov.: trunk appendages. (**A–C**) subcoxa I–III, respectively; (**D**) trochanteral organ; (**E**) modified chaeta of tibiotarsus III; (**F**) distal tibiotarsus and empodial complex III (anterior view); (**G**) collophore (lateral view); (**H**) distal part of manubrium (ventral view); (**I**) manubrial plate (dorsal view); (**J**) distal dens and mucro (inner view).

Figure 61, Figure 62 and Figure 63, Table 1 and Table 5

*Type material.* Holotype female in slide (1950/CRFS-UEPB): Brazil, Minas Gerais State, Mariana municipality, Bento Rodrigues districts, East Region of mining, 20°12′29.5″ S, 43°26′19.9″ W, 966 m., 29.x.2012, Bioespeleo et al. coll. Paratype in slide (1953/CRFS-UEPB): 1 female, same data as holotype. Paratype in slide (2277/CRFS-UEPB donated to INPA/093): 1 female, same data as holotype. Paratypes in slides (2068, 2227/CRFS-UEPB): 1 male and 1 female, same data as holotype, except 17.x.2012. Paratype in slide (1956/CRFS-UEPB): 1 juvenile, same data as holotype, 11.xii.2012. Paratypes in slides (1694–96/CRFS-UEPB): 1 female and 2 juveniles, same data as holotype except 06–16.vi.2011, Bessi et al. coll. Paratypes in slides (13532, 13536/CRFS-UEPB): 1 male and 1 female, same data as holotype, except between Bento Rodrigues and Morro da Água Quente districts, 20°09′56.4″ S, 43°23′15.5″ W, 877m, 11-13.ix.2017, Carste et al. coll. Paratype in slide (13128/CRFS-UEPB): 1 juvenile, as above, except 30.i.2018. Paratype in slide (13250/CRFS-UEPB donated to INPA/093): 1 juvenile, same data as holotype, except mining near Bento Rodrigues district, 20°09′51.0″ S, 43°28′21.5″ W, 952 m, 08.vi.2017.

*Description.* Total length (head + trunk) of specimens 0.79–1.1 mm (n = 4), holotype 1.05 mm.

Head. Ratio antennae:trunk = 1:2.73–4.36 (n = 4), holotype 1:2.95; Ant III smaller than Ant II length; Ant segment ratio as I:II:III:IV = 1:1.65–2.17:1–1.6:2.71–4, holotype 1:1.65:1.35:2.71. Ant IV with numerous common sens dorsally and ventrally (b–c), dorsal face with 1 f more apically and at least 1 e proximally; Ant III with common sens b–c, apical organ with 2 slightly swollen apical sens clubs surrounded by at least 7 sens c (Figure 61A); Ant II with common sens b–c. Clypeal formula with 4 (l1–2), 2 (ft), and 3 (pf0–1) ciliate chaetae, all apically acuminate, pf1 shorter than pf0, l2 longer (Figure 61B). Prelabral chaetae ciliate and not bifurcate. Labral chaetae smooth, no modifications (Figure 61B). Labial papilla D with 4 appendages; papilla E with l.p. finger-shaped, almost straight and surpassing the base of apical appendage (Figure 61C). Labial proximal chaetae smooth (lpc1, 3–4, and 6–7), lpc3 and lpc6 shorter and slightly thinner than others (Figure 61C). Maxillary palp with b.c. weakly ciliate and 1.12 longer than t.a. (Figure 61D, anterior sublobal minute appendage not represented). Head dorsal chaetotaxy (Figure 61E) with 11 “An” (An1a–3), 5 “A” (A0–3, A5, and A2a absent), 4 “M” (M1–4), 6 “S” (S0 and S2–6), 3 “Ps” (Ps2–3 and Ps5), 4 “Pa” (Pa2–5), 2 “Pm” (Pm1 and Pm3), 7 “Pp” (Pp1–7), and 4 “Pe” (Pe3–6) chaetae; An1a–3a, A0, A2–3, and M2 as mac; interocular p mic present; head posterior region with at least 8 cervical spine-like mac (not represented in Figure 61E). Basomedian and basolateral labial fields with a1–5 smooth; M1–2, E, and L1–2 uniformly to unilaterally ciliate; and r reduced, M1 subequal to M2, L2 slightly longer than others (Figure 61F). Ventral chaetotaxy with 17 ciliate chaetae plus 1 reduced lateral spine; postlabial formula 4 (G1–4), 1 (X4), 4 (H1–4), and 2 (J1–2) chaetae, 6 latero-posterior chaetae thicker (including J2), b.c. present (Figure 61F).

Thorax dorsal chaetotaxy (Figure 62A). Th II a, m, and p series with 1 (a5), 4 (m–5e?), and 6 (p1–6) mic, respectively. Th III a, m, and p series with 7 (a1–4 and a6–7i), 4 (m2 and m4–6), and 6 (p1–6) mic, respectively. Ratio Th II:III = 1.96:1 in holotype.

Abdomen dorsal chaetotaxy (Figure 62B,C). Abd I a, m, and p series with 4 (a1–3 and a5), 4–5 (m2–6), and 2 (p5–6) mic, respectively, a3 as normal mic. Abd II a, m, and p series with 3 (a2–3 and a6), 5–6 (m3–3e and m4–7), and 5 (p4–7) chaetae, respectively, m3 and m5 as mac; a5 and m2 bothriotricha surrounded by 2 (lm and ll) and 2 (ml and mi) fan-shaped chaetae, respectively. Abd III a, m, and p series with 4 (a2–3 and a6–7), 7 (m3–4, am6, pm6, and m7–8), and 5 (p3 and p5–8) chaetae, respectively, pm6 and p6 as mac, a3 and m3 as reduced mic, as sens elongated, ms absent; m2 bothriotrichum associated with 3 (a2, ml, and mi) and a5 and m5 bothriotricha with 6–7 (am6, a6, li, lm, ll, im, and em) fan-shaped chaetae. Abd IV A–Fe series with 5 (A2–6), 4 (B2 and B4–6), 1 (Be3), 4–5 (C1–4), 5 (T1, T3, and T5–7), 1 (Te7), 4 (D1–3p), 2 (De1 and De3), 5 (E1–4p), 3 (F1–3), and 5 (Fe1–5) chaetae, respectively, with 3 central (B5–6 and C1) and 5 lateral mac (D3, E2–4, and F1); T2 and T4 bothriotricha surrounded by 5–6 (D1, s, m, a, and C1p plus unnamed) and 2 (pe and pi) fan-shaped chaetae, respectively; A2–4, B2, B4, and C2–3 as reduced mic; 4–5 sens present, ps type I, as plus 3 anterior type II, r series laterally with 3 mic. Abd V m and p series with 3 (m2–3 and m5) and 3 (p4–5) central mac, respectively. Ratio Abd III:IV = 1:4.31 in holotype. Th II–Abd IV formula with 00|010 + 21 + 2 mac.

Legs. Subcoxa I with 6 chaetae and 2 psp; subcoxa II with “a” row of 5 chaetae, “p” row with 5–8 chaetae and 2 psp; subcoxa III with one row of 8 chaetae and 2 posterior psp (Figure 63A–C). Trochanteral organ with about 12 spine-like chaetae (Figure 63D). Tibiotarsus I–III formula with 1, 3, and 3 mac type I (Figure 63E). Unguis outer side with paired teeth straight and not developed on distal half, nearside the inner basal pair; inner side with wide lamella and 4 teeth, basal pair slightly unequal, b.p. larger than b.a. and reaching m.t. apex, m.t. on distal one third and smaller than basal teeth, a.t. minute on distal one fifth. Unguiculus with all lamellae smooth and acuminate (ai, ae, pi, and pe), except pe serrate (Figure 63F); ratio unguis:unguiculus in holotype = 1.46:1. Tibiotarsal smooth chaeta subequal or slightly longer than unguiculus lenght; tenent hair capitate and about 0.78 smaller than unguis outer edge.

Collophore (Figure 63G). Anterior side with 7–8 ciliate apically acuminate chaetae, 3–4 proximal thinner and shorter, and others subequal plus 1 distal thicker mac; posterior side distally with 2 smooth and 1 ciliate lateral chaeta plus 1 reduced spine; lateral flap with 8–9 smooth chaetae (2 larger).

Furcula. Only with ciliate chaetae and scales. Manubrium ventrally with 5 subapical and about 9 distal scales; manubrial plate with 3–4 ciliate chaetae (1–2 inner mac) and 2 psp (Figure 63H,I). Mucro basal tooth gently larger than distal tooth, basal spine surpassing the apex of basal tooth (Figure 63J).

*Etymology.* Refers to similar morphology to *P. marianensis* sp. nov. (from Latin: spuri–false).

*Remarks. Pseudosinella spurimarianensis* sp. nov. resembles *P.* *marianensis* sp. nov., *P. cearensis* sp. nov. and *P. strinatii* [51] (Table 1, Table 4, and Table 5). However, *P. spurimarianensis* sp. nov. differ from these last two species by labral chaetae smooth (a1–2 and m0–2 with filaments and p0–2 ciliate in *P. cearensis* sp. nov.), head M2 mac present (absent in *P. cearensis* sp. nov.) and S3 mac absent (present in *P. strinatii*), and Abd IV with 3 central mac (1 in *P. cearensis* sp. nov. and 2 in *P. strinatii*). *Pseudosinella spurimarianensis* sp. nov. also differ these species in basomedian and basolateral labial fields with M1–2, E, and L1–2 weakly ciliate (smooth in *P. strinatii*, unilaterally ciliate in *P. marianensis* sp. nov., and multiciliate in *P. cearensis* sp. nov.), tibiotarsus I–III formula with 1, 3, and 3 mac type I (1, 2, and 1 mac type VI in *P. cearensis* sp. nov., and type III in *P. marianensis* sp. nov.). *Pseudosinella spurimarianensis* sp. nov. still differs from *P. marianensis* sp. nov. by tenent hair capitate (acuminate in *P. marianensis* sp. nov.), unguis a.t. present (absent in *P. marianensis* sp. nov.), and manubrium ventrally only with distal ciliate chaetae (1 modified in *P. marianensis* sp. nov.). See also the comparison these species in remarks of *P. cearensis* sp. nov. and Table 1, Table 4, and Table 5.

### 3.4. Species Group with Prelabral Chaetae Bifurcate and Unguiculus “pe” Lamellae Acuminate

#### 3.4.1. *Pseudosinella ambigua* Zeppelini, Brito, and Lima, 2018

**Figure 64:**
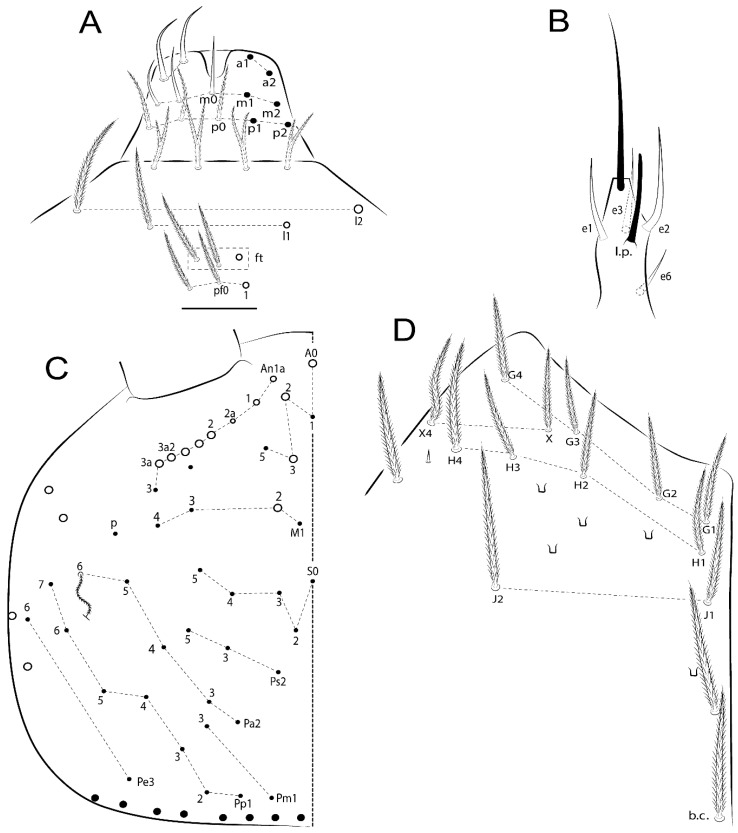
*Pseudosinella ambigua*: head. (**A**) chaetotaxy of clypeus, prelabrum, and labrum; (**B**) labial papilla E (right side); (**C**) head dorsal chaetotaxy (left side); (**D**) postlabial chaetotaxy (left side).

**Figure 65:**
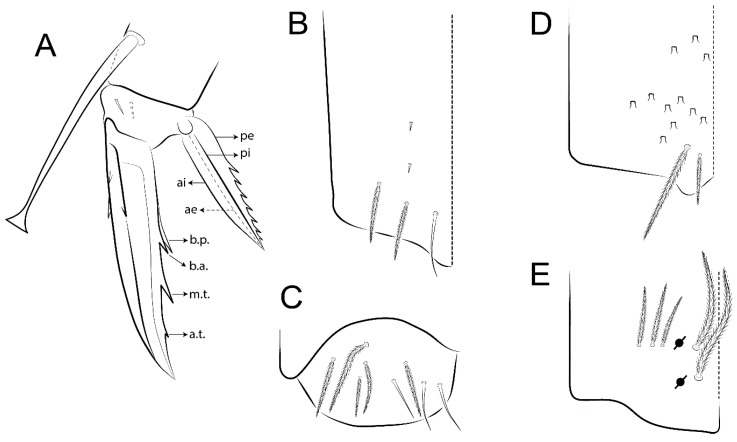
*Pseudosinella ambigua*: appendages. (**A**) distal tibiotarsus and empodial complex III (anterior view); (**B**,**C**) collophore: (**B**) posterior side, (**C**) lateral flap; (**D**,**E**) manubrium: (**D**) ventral side distally, (**E**) manubrial plate (dorsal view).

Figure 64 and Figure 65, Table 1 and Table 6

*Pseudosinella ambigua* Zeppelini, Brito, and Lima, 2018: p. 69, Figure 17, Figure 18, Figure 19, Figure 20, Figure 21, Figure 22, Figure 23, Figure 24 and Figure 25, Brazil, Minas Gerais, Pedro Leopoldo (orig. descr.) [14].

*Examined type material.* Holotype and paratype (10505, 10506/CRFS-UEPB). The five specimens used as additional material of *P. ambigua* (10500–04/CRFS-UEPB) is removed here because they are other species.

*Examined additional material.* 1 male and 1 juvenile in slides (7290, 7037/CRFS-UEPB): Brazil, Minas Gerais State, Pedro Leopoldo municipality, “HOLC-0026, F38” cavities, 19°34′45.7″ S, 44°01′00.0″ W and 19°34′30.2″ S, 44°00′27.1″ W respectively, 04–20.iii.2015 and 07.vi.2016, Carste et al. and R.V. Lorena coll. 2 females, 1 male and 1 juvenile in slides (9252–53, 7273, 4373/CRFS-UEPB): Pains municipality, “ICPA-866, ICPA-867, ICPA-928, ICPA-608” cavities, 20°22′11.4″ S, 45°36′27.1″ W, 20°22′18.8″ S, 45°37′31.8″ W and 20°22′45.9″ S, 45°36′27.7″ W, 24–27.xi.2015, 23–30.iv.2015 and 05.viii–04.x.2013, Carste et al. and Bueno, et al. coll. 2 females in slides (11549, 11555/CRFS-UEPB): Vespasiano municipality, “AM-03” cavity, 19°42′46.8″ S, 43°56′33.2″ W, 29.iii.2017 and 20.vii.2017, Ativo Ambiental et al. coll. 1 female in slide (11366/CRFS-UEPB): São José da Lapa municipality, “AM-31” cavity, 19°42′19.8″ S, 43°56′57.2″ W, 02.iv.2017, Ativo Ambiental et al. coll.

*Description.* Head. Clypeal formula with 4 (l1–2), 3 (f), and 3 (pf0–1) ciliate chaetae, l1 larger, others subequal (Figure 64A). Prelabral chaetae ciliate, inner chaetae bifurcate equally, outer chaetae bifurcated unequally. Labral a1–2 and m0–2 chaetae smooth, p0–2 chaetae ciliate, a1–2 gently thicker, and others subequal. Labial papilla D with 3 appendages; papilla E with l.p. finger-shape, curved, and surpass the base of apical appendage (Figure 64B). Head dorsal chaetotaxy (Figure 64C) with 9 “An” (An1a–3), 5 “A” (A0–3 and A5), 4 “M” (M1–4), 5 “S” (S0 and S2–5), 3 “Ps” (Ps2–3 and Ps5), 4 “Pa” (Pa2–3 and Pa5), 2 “Pm” (Pm1 and Pm3), 7 “Pp” (Pp1–7), and 2 “Pe” (Pe3 and Pe6) chaetae; An1a–1, An2–3a, A0, A2–3, and M2 as mac; interocular p mic present; head posterior region with 8 cervical like-spine mac. Basomedian and basolateral labial fields with A1–5 weakly ciliate; M1–2, E, and L1–2 ciliate; and r reduced. Labial proximal chaetae weakly ciliate (lpc1, 3–4, and 6–7), lpc3–4, and lpc6 gently smaller. Ventral chaetotaxy with about 15 ciliate chaetae and 1 reduced lateral spine; postlabial formula 4 (G1–4), 2 (X and X4), 4 (H1–4), and 2 (J1–2) chaetae, H4 plus 1 lateral chaeta thicker, b.c. present (Figure 64D). Th II–Abd IV formula with 00|020 + 21 + 2 mac. Unguis outer side with paired teeth straight and not developed; inner side with wide lamella and 4 teeth, basal pair equal (b.a. and b.p.), m.t. on distal one third and gently larger than basal teeth, minute a.t. on distal one fifth. Unguiculus with all lamellae acuminate and smooth (ai, ae, pi, and pe), except pe lamella serrate (Figure 65A). Collophore posterior side distally with 1 smooth chaeta, 2 ciliate chaetae, and 2 subdistal reduced spine; lateral flap with 3 smooth and 5 ciliate chaetae, 1 larger (Figure 65B,C). Furcula. Only with ciliate chaetae and scales. Manubrium ventrally with 3 subapical and about 8 distal scales, plus 2 unequal chaetae ciliate; manubrial plate with 5 ciliate chaetae (2 inner mac) and 2 psp (Figure 65D,E)

*Remarks.* In the original description [14], *P. ambigua* was not compared with *P. folsomi* from Texas, USA [7,55]. These species are similar in depigmentations and eyes absent, basomedian labial field with r reduced, Th II–III devoid of mac, Abd II with m3 and m3e mac, Abd IV with 3 central mac (B5–6 and C1), tenent hair capitate, basal basal teeth equal, unguiculus acuminate, and mucro basal tooth gently larger than apical tooth (Table 1, Table 4, and Table 6). However, *P. ambigua* differs from this species by head chaetotaxy with A3 and M2 mac (mic in *P. folsomi*), basomedian and basolateral labial field with M1–2, E, and L1–2 ciliate (smooth in *P. folsomi*), Abd II with a2 as mic (mac in *P. folsomi*), and unguis apical tooth present (absent in *P. folsomi*).

In addition, some morphological characteristics have been misinterpreted or omitted in the original description and for this reason they are included here. It was reported that the labral a1–2 and m0–2 chaetae are serrate and l.p. of labial papilla E curled anteriorly around the papilla base. However, these features were actually observed in specimens of *P. acantholabrata* sp. nov. (see Figure 8B,C) from Conceição do Mato Dentro municipality, which were mistakenly identified as additional material of *P. ambigua*. On head, 5 “An” (An2–3a) and 2 “A” (A2 and A3) chaetae are reported as mac, but illustrated as mic [14] (p. 70, Figure 18). Besides that, at least eigth chaetae were omitted in series “M” (M3–4), “Pp” (Pp2–6), and “Pe” (Pe3), of which are typically in *Pseudosinella* species and Lepidocyrtinae in general [15,16,57,58]. Other generic features unclear in the original description (probably due to the leg position of the holotype) are the size and shape of the inner and outer teeth of unguis as well as the four unguiculus lamellae (Figure 65A). In addition, in collophore posteriorly, there are 2 subdistal spines and 1 smooth chaeta while, in lateral flap, there are 5 ciliate and 3 smooth chaetae, but in the original description, only smooth chaetae were reported in both regions [14] (p. 72, Figure 24). Finally, the manubrial plate holds 2 inner chaetae larger than others and the manubrium ventral chaetotaxy is herein described.

#### 3.4.2. *Pseudosinella chimerambigua* sp. nov. Oliveira, Lima, and Cipola

**Figure 66:**
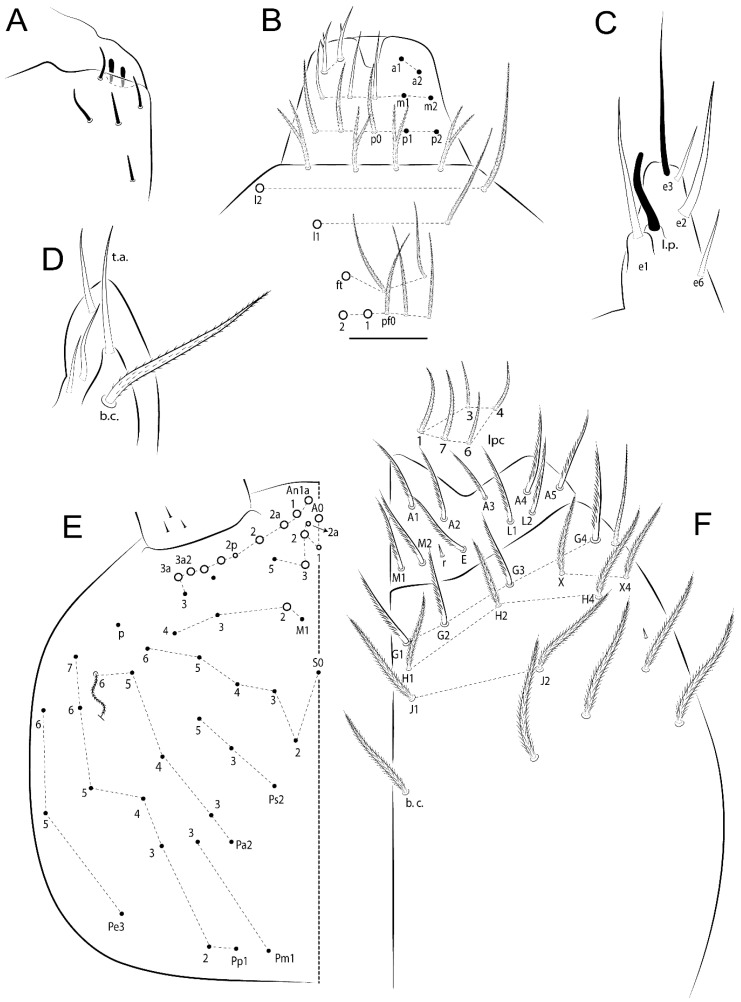
*Pseudosinella chimerambigua* sp. nov.: head. (**A**) Ant III apical organ (lateral view); (**B**) chaetotaxy of clypeus, prelabrum, and labrum; (**C**) labial papilla E (right side); (**D**) maxillary palp and sublobal plate (right side); (**E**) head dorsal chaetotaxy (left side); (**F**) labial proximal chaetae, basomedian and basolateral labial fields, and complete postlabial chaetotaxy (right side).

**Figure 67:**
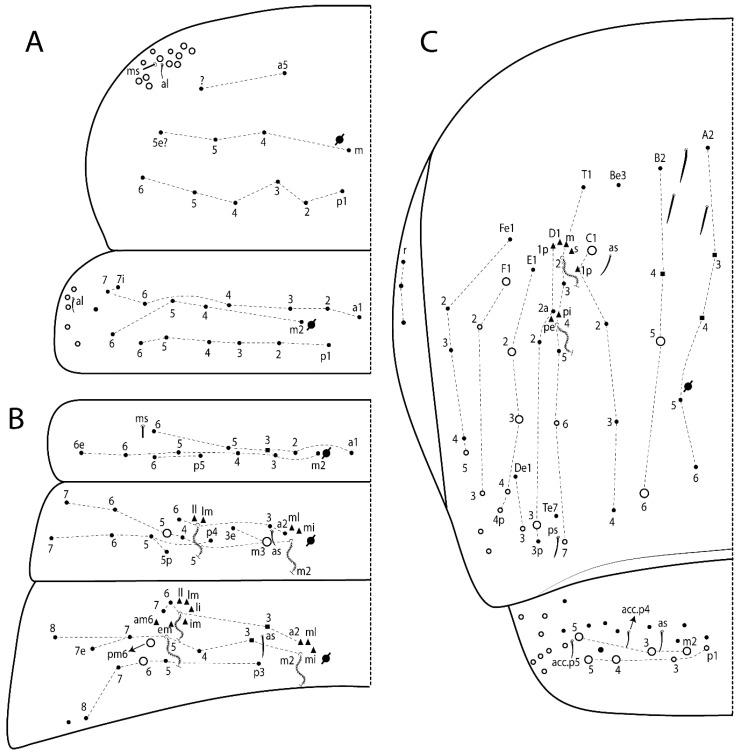
*Pseudosinellachimerambigua* sp. nov.: dorsal chaetotaxy. (**A**) Th II–III; (**B**) Abd I–III; (**C**) Abd IV–V.

**Figure 68:**
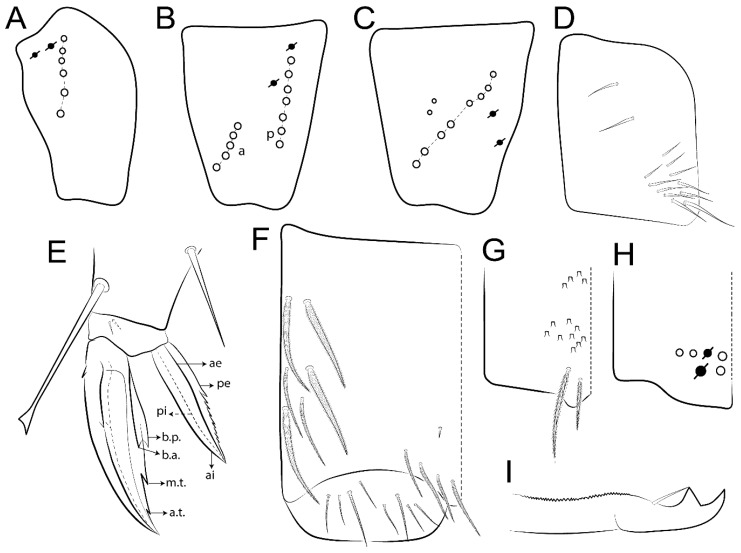
*Pseudosinella chimerambigua* sp. nov. (**A–C**) subcoxa I–III, respectively; (**D**) trochanteral organ; (**E**) distal tibiotarsus and empodial complex III (anterior view); (**F**) collophore (lateral view); (**G**) distal part of manubrium (ventral view); (**H**) manubrial plate (dorsal view); (**I**) distal dens and mucro (outer view).

Figure 66, Figure 67 and Figure 68, Table 1 and Table 6

Type material. Holotype female in slide (6585/CRFS-UEPB): Brazil, Minas Gerais State, Vespasiano municipality, next to “Fazenda Zumbi”, 19°41′57.8″ S, 43°53′59.0″ W, 694 m, 15–25vi.2015, Carste et al. coll. Paratypes in slides (6553–54/CRFS-UEPB): 1 male and 1 female, same data as holotype, except 19°41′52.3″ S, 43°53′44.1″ W, 714 m, 06-16.i.2015 and 05-06.v.2016. Paratypes in slides (6559–60, 6565/CRFS-UEPB): 1 male and 2 females, same data as holotype, except, 19°41′56.8″ S, 43°53′58.6″ W, 702 m, and 19°41′55.3″ S, 43°53′56.5″ W, 709 m, respectively. Paratype in slide (6555/CRFS-UEPB donated to INPA/094): 1 female, same data as holotype, except 19°41′52.3″ S, 43°53′44.1″ W, 714 m, 06-16.i.2015.

*Description.* Total length (head + trunk) of specimens 0.89–1.27 mm (n = 5), holotype 0.89 mm.

Head. Ratio antennae:trunk = 1:2.24–2.82 (n = 4), holotype 1:2.24; Ant II smaller than Ant III length; Ant segment ratio as I:II:III:IV = 1:1.66–2.81:1.58–2.63:3.43–4.81, holotype 1:2.31:2.63:4.45. Ant IV dorsally with numerous sens (b–c) and chaetae (i–k). Ant III with 2 apical sens clubs surrounded by at least 5 sens c (Figure 66A). Ant II with common sens type b–c, 1 c dorso-distal, 2 d dorso-lateral, and 4 b and 1 d ventro-subdistal. Clypeal formula with 4 (l1–2), 3 (ft), and 5 (pf0–2) ciliate and apically acuminate chaetae, l1–2 larger (Figure 66B). Prelabral chaetae weakly ciliate and bifurcate. Labral a1–2 chaetae thicker and with 1–2 lateral filaments, respectively, m0–2 chaetae smooth, p0–2 chaetae weakly ciliate (Figure 66B). Labial papilla D with 3 appendages; papilla E with l.p. finger-shape, sinuous, and exceed the base of apical appendage (Figure 66C). Maxillary palp with b.c. weakly ciliate and 1.09 longer than the t.a. (Figure 66D). Dorsal head chaetotaxy (Figure 66E) with 10 “An” (An1a–3), 6 “A” (A0–3 and A5), 4 “M” (M1–4), 6 “S” (S0 and S2–6), 3 “Ps” (Ps2–3 and Ps5), 4 “Pa” (Pa2–5), 2 “Pm” (Pm1 and Pm3), 7 “Pp” (Pp1–7), and 3 “Pe” (Pe3 and Pe5–6) chaetae; An1a–3a (except An2p), A0, A2–3, and M2 as mac; interocular p mic present; head posterior region with 9 cervical like-spine mac (not represented in Figure 66E). Basomedian and basolateral labial fields with A1–5, M1–2, E, and L1–2 unilaterally and heavily ciliate and r reduced (Figure 66F). Labial proximal chaetae weakly ciliate (lpc1, 3–4, and 6–7), lpc3–4 and lpc6 gently smaller. Ventral chaetotaxy with about 17 chaetae and 1 reduced lateral spine, postlabial formula 4 (G1–4), 2 (X and X4), 3 (H1–2 and H4), and 2 (J1–2) chaetae, G1–4 unilaterally and heavily ciliate, 1 laterally weakly ciliate, another ciliate, b.c. present (Figure 66F).

Thorax dorsal chaetotaxy (Figure 67A). Th II a, m, and p series with 2 (a5 and a?), 4 (m and m4–5e?), and 6 (p1–6) mic, respectively. Th III a, m, and p series with 7 (a1–4 and a6–7i), 4 (m2 and m4–6), and 6 (p1–6) mic, respectively. Ratio Th II:III = 1.63–2.5:1 (n = 5), holotype 1.99:1.

Abdomen dorsal chaetotaxy (Figure 67B,C). Abd I a, m, and p series with 5 (a1–3 and a5–6), 6 (m2–6e), and 2 (p5–6) mic, respectively. Abd II a, m, and p series with 3 (a2–3 and a6), 6 (m3–7), and 5 (p4–7) chaetae, respectively, m3 and m5 as mac; a5 and m2 bothriotricha with 2 (ll and lm) and 2 (ml and mi) fan-shaped chaetae, respectively. Abd III a, m, and p series with 4 (a2–3 and a6–7), 7 (m3–4, am6, pm6, and m7–8), and 5 (p3 and p5–8) chaetae, respectively, pm6 and p6 as mac, a3 and m3 as reduced mic, ms absent; m2 bothriotrichum associated with 3 (a2, ml, and mi) and a5 and m5 bothriotricha with 6 (ll, lm, li, am6, em, and im) fan-shaped chaetae. Abd IV A–Fe series with 5 (A2–6), 4 (B2 and B4–6), 1 (Be3), 5 (C1–4), 5 (T1, T3, and T5–7), 1 (Te7), 6 (D1–3p), 2 (De1 and De3), 5 (E1–4p), 3 (F1–3), and 5 (Fe1–5) chaetae, respectively, with 3 central (B5–6 and C1) and 4 lateral mac (D3, E2–3, and F1); T2 and T4 bothriotricha with 5 (D1–1p, m, s, and C1p) and 2 (pe and pi) fan-shaped chaetae, respectively; 5 sens present, ps type I, as and 3 of uncertain homology type II, and r series laterally with 3 chaetae. Abd V m and p series with 3 (m2–3 and m5) and 2 (p4–5) central mac, respectively. Ratio Abd III:IV = 1:3.12–4.26 (n = 5), holotype 1:3.57. Th II–Abd IV formula with 00|010 + 21 + 2 mac.

Legs. Subcoxa I with “p” row with 6 chaetae and 2 psp; subcoxa II with “a” row with 5 chaetae, “p” row with 7 chaetae and 2 psp; subcoxa III with one row of 8 chaetae plus 2 anterior chaeta and 2 psp (Figure 68A–C). Trochanteral organ with 13 spine-like chaetae (Figure 68D). Tibiotarsus I–III formula with 1, 1, and 2 mac type III (as Figure 51E). Unguis outer side with paired teeth straight on proximal one third; inner side with wide lamella and 4 teeth, basal pair (b.a. and b.p.) equal and not reaching the m.t. apex, m.t. on distal one third and subequal to basal teeth, and a.t. minute on distal one seventh. Unguiculus with all lamella smooth and acuminate (ai, ae, pi, and pe), except pe serrate (Figure 68E); ratio unguis:unguiculus in holotype = 1:0.55. Tibiotarsal smooth chaeta about 0.92 smaller than unguiculus; tenent hair capitate and about 0.73 smaller than unguis outer edge.

Collophore (Figure 68F). Anterior side with 6 ciliate apically acuminate chaetae, 4 larger, others subequal in lenght; posterior side distally with 4 chaetae weakly ciliated and 1 subdistal reduced spine; lateral flap with 8 chaetae weakly ciliate (2 larger).

Furcula. Only with ciliate chaetae and scales. Manubrium ventrally with 4 subapical and 9 distal scales; manubrial plate with 4 ciliate chaetae (2 inner mac) and 2 psp (Figure 68G,H). Mucro basal tooth gently larger than distal tooth, basal spine reaching the apex of basal tooth (Figure 68I).

*Etymology.* Refers to mix of morphological features, resembling both *P. ambigua* and *P. parambigua* sp. nov. (from Greek: chimeraa hybrid monster).

*Remarks. Pseudosinella chimerambigua* sp. nov. resembles *P. ambigua* and *P. parambigua* sp. nov. by labral m0–2 chaetae smooth and p0–2 ciliate, prelabral chaetae ciliate and bifurcate, head with 4 mac (A0, A2–3, and M2), Th II–Abd I devoid of mac, Abd III–IV with 0 and 3 central mac, respectively, tenent hair capitate, and unguis with 4 inner teeth (Table 1 and Table 6). However, *P. chimerambigua* sp. nov. differs from these species by labral a1–2 with filaments (smooth in these species), all chaetae unilaterally and heavily ciliate on basomedian and basolateral labial fields (except r) and presence of postlabial G1–4, while in *P. ambigua* and *P. parambigua* sp. nov. has A1–5 weakly ciliate and M1–2, E, and L1–2 multiciliate, including G1–4 postlabial. They also differ by unguis basal teeth equal (unequal in *P. parambigua* sp. nov.), unguiculus pe lamella serrate (smooth in *P. parambigua* sp. nov.), collophore posteriorly with 4 ciliate chaetae and 1 spine (1 smooth, 2 ciliate, and 2 spines in *P. ambigua*), and lateral flap with chaetae weakly ciliate, while in these species has smooth and ciliate chaetae. On manubrial plate there are 2 outer chaetae in *P. chimerambigua* sp. nov. and 3 outer chaetae in *P. ambigua* and *P. parambigua* sp. nov. Finally, in Abd II only *P. ambigua* has 2 central mac (m3–3e) plus prelabral chaetae bifurcate compared to the other species. More data is presented in the remarks of *P. ambigua* and Table 1 and Table 6.

#### 3.4.3. *Pseudosinella macrolignicephala* sp. nov. Oliveira, Lima, and Cipola

**Figure 69:**
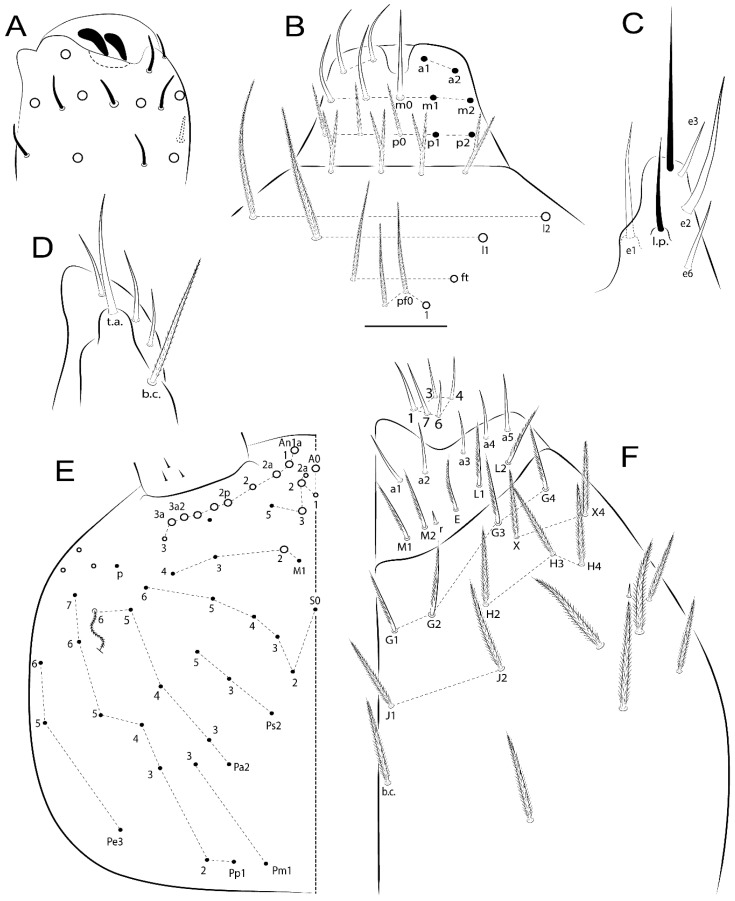
*Pseudosinella macrolignicephala* sp. nov.: head. (**A**) Ant III apical organ (lateral view); (**B**) chaetotaxy of clypeus, prelabrum, and labrum; (**C**) labial papilla E (right side); (**D**) maxillary palp and sublobal plate (right side); (**E**) head dorsal chaetotaxy (left side); (**F**) labial proximal chaetae, basomedian and basolateral labial fields, and complete postlabial chaetotaxy (right side).

**Figure 70:**
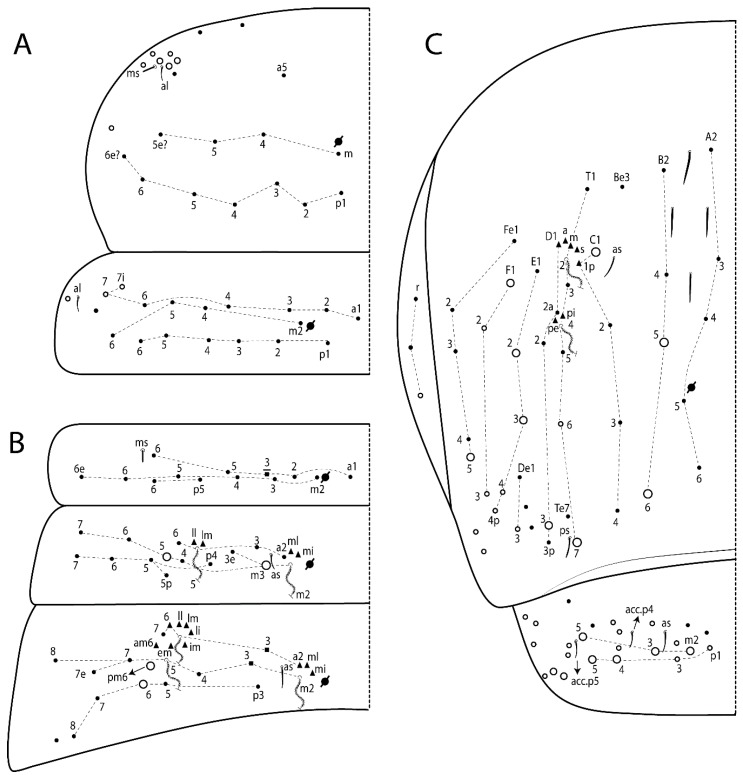
*Pseudosinella macrolignicephala* sp. nov.: dorsal chaetotaxy. (**A**) Th II–III; (**B**) Abd I–III; (**C**) Abd IV–V.

**Figure 71:**
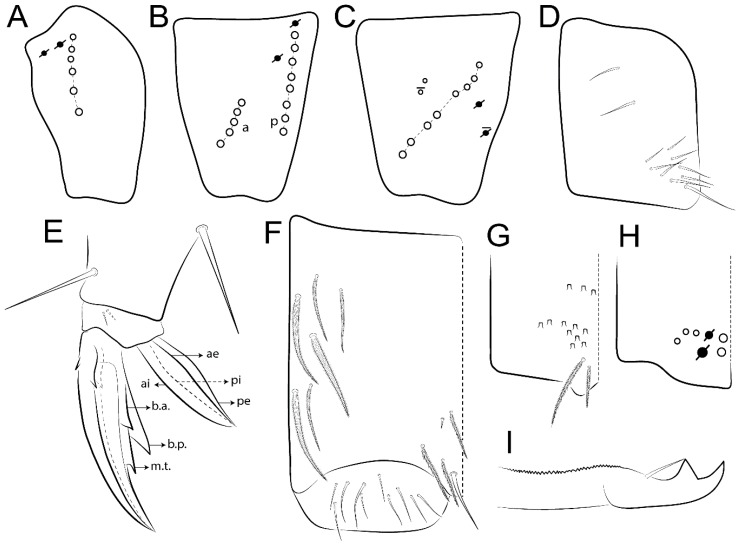
*Pseudosinella macrolignicephala* sp. nov. (**A–C**) subcoxa I–III, respectively; (**D**) trochanteral organ; (**E**) distal tibiotarsus and empodial complex III (anterior view); (**F**) collophore (lateral view); (**G**) distal part of manubrium (ventral view); (**H**) manubrial plate (dorsal view); (**I**) distal dens and mucro (outer view).

Figure 69, Figure 70 and Figure 71, Table 1 and Table 6

*Type material.* Holotype male in slide (13764/CRFS-UEPB): Brazil, Minas Gerais State, Itabirito municipality, next to “Vieira de Cima”, 20°17′50.9″ S, 43°56′34.5″ W, 1473 m, 10-14.xii.2018, Carste et al. coll. Paratype in slide (13765/CRFS-UEPB): 1 male, same data as holotype. Paratypes in slides (5754–55/CRFS-UEPB): 1 female and 1 juvenile, same data as holotype, except 20°17′10.2″ S, 43°56′47.1″ W, 1459 m, 28-30.iv.2014. Paratype in slide (2719/CRFS-UEPB): 1 female, same data as holotype, except 20°17′08.2″ S, 43°56′44.7″ W, 1418 m, 07-09.xi.2011. Paratype in slide (2719, 2786/CRFS-UEPB): 1 female, same data as holotype, except 20°17′07.1″ S, 43°56′43.7″ W, 1400 m, 18–25.iv.2007. Paratypes in slides (1740, 5759, 10722, 10807/CRFS-UEPB donated to INPA/095): 1 male and 3 females, same data as holotype, except 20°17′07.9″ S, 43°56′51.6″ W, 1484 m, 28-30.iv.2014.

*Description.* Total length (head + trunk) of specimens 0.73–1.27 mm (n = 4), holotype 1.13 mm.

Head. Ratio antennae:trunk = 1:2.65–3.17 (n = 2), holotype 1:3.17; Ant II smaller than Ant III length; Ant segment ratio as I:II:III:IV = 1 1.95–2.14:2.18–2.24 4.80–4.91, holotype 1:2.14:2.24:4.80. Ant IV dorsally with numerous sens (b–c) and chaetae (i–k). Ant III with 2 swollen apical sens clubs surrounded by at least 8 sens c (Figure 69A). Ant II with common sens b–c, 2 b, and 1 c dorso-distal; 2 c dorso-lateral; and 4 c ventro-subdistal. Clypeal formula with 4 (l1–2), 2 (ft), and 3 (pf0–1) ciliate chaetae, all apically acuminate, l1–2 larger (Figure 69B). Prelabral chaetae weakly ciliate and bifurcate. Labral chaetae smooth, no modifications, except p0–2 chaetae weakly ciliate (Figure 69B). Labial papilla D with 3 appendages; papilla E with l.p. pointed, straight and exceed the base of apical appendage (Figure 69C). Maxillary palp with b.c. weakly ciliate and 1.09 longer than the t.a. (Figure 69D). Head dorsal chaetotaxy (Figure 69E) with 10 “An” (An1a–3), 6 “A” (A0–3 and A5), 4 “M” (M1–4), 6 “S” (S0 and S2–6), 3 “Ps” (Ps2–3 and Ps5), 4 “Pa” (Pa2–5), 2 “Pm” (Pm1 and Pm3), 7 “Pp” (Pp1–7), and 3 “Pe” (Pe3 and Pe5–6) chaetae; An1a–3a, A0, A2–A3, and M2 as mac; interocular p mic present; head posterior region with 10 cervical like-spine mac (not represented in Figure 69E). Basomedian and basolateral labial fields with a1–5 smooth; M1–2, E, and L1–2 unilaterally and heavily ciliate; and r reduced (Figure 69F). Labial proximal chaetae smooth (lpc1, 3–4, and 6–7), lpc6 smaller. Ventral chaetotaxy with about 18 ciliate chaetae and 1 reduced lateral spine, postlabial formula 4 (G1–4), 2 (X and X4), 3 (H2–4), and 2 (J1–2) ciliate chaetae, G1–4 unilaterally and heavily ciliate, b.c. present (Figure 69F).

Thorax dorsal chaetotaxy (Figure 70A). Th II a, m, and p series with 1 (a5), 4 (m and m4–5e?), and 7 (p1–6?) mic, respectively. Th III a, m, and p series with 7 (a1–4 and a6–7i), 4 (m2 and m4–6), and 6 (p1–6) mic, respectively. Ratio Th II:III = 1.38–2.16:1 (n = 5), holotype 2.11:1.

Abdomen dorsal chaetotaxy (Figure 70B,C). Abd I a, m, and p series with 4–5 (a1–3 and a5–6), 6 (m2–6e), and 2 (p5–6) mic, respectively. Abd II a, m, and p series with 3 (a2–3 and a6), 6 (m3–7), and 5 (p4–7) chaetae, respectively, m3 and m5 as mac; a5 and m2 bothriotricha with 2 (ll and lm) and 2 (ml and mi) fan-shaped chaetae, respectively. Abd III a, m, and p series with 4 (a2–3 and a6–7), 7 (m3–4, am6, pm6, and m7–8), and 5 (p3 and p5–8) chaetae, respectively, pm6 and p6 as mac, a3 and m3 as reduced mic, and as sens elongated, ms absent; m2 bothriotrichum associated with 3 (a2, ml, and mi) and a5 and m5 bothriotricha with 7 (a6, am6, ll, lm, li, em, and im) fan-shaped chaetae. Abd IV A–Fe series with 5 (A2–6), 4 (B2 and B4–6), 1 (Be3), 5 (C1–4), 5 (T1, T3, and T5–7), 1 (Te7), 5 (D1–3p), 2 (De1 and De3), 5 (E1–4p), 3 (F1–3), and 5 (Fe1–5) chaetae, respectively, with 3 central (B5–6 and C1) and 6 lateral mac (T7, D3, E2–3, F1, and Fe5); T2 and T4 bothriotricha with 5 (D1, a, m, s, and C1p) and 2 (pe and pi) fan-shaped chaetae, respectively; 6 sens present, ps type I, as and 4 of uncertain homology type II, r series laterally with 3 chaetae. Abd V m and p series with 3 (m2–3 and m5) and 2 (p4–5) central mac, respectively. Ratio Abd III:IV = 1:3.17–4.65 (n = 5), holotype 1:3.71. Th II–Abd IV formula with 00|010 + 21 + 2 mac.

Legs. Subcoxa I with “p” row with 6 chaetae and 2 psp; subcoxa II with “a” row with 5 chaetae, “p” row with 8 chaetae and 2 psp; subcoxa III with one row of 8 plus 1–2 anterior chaetae and 1–2 psp (Figure 71A–C). Trochanteral organ with 11 spine-like chaetae (Figure 71D). Tibiotarsus I–III formula with 1, 1, and 3 mac type III (as Figure 51E). Unguis outer side with paired teeth straight and on proximal one fourth; inner side with slender lamella distally and 3 teeth, basal pair (b.a. and b.p.) unequal, b.p. larger and almost reaching the m.t. apex, m.t. on distal one third and subequal to b.a., a.t. absent. Unguiculus with all lamella smooth and acuminate (ai, ae, pi, and pe) (Figure 71E); ratio unguis:unguiculus in holotype = 1:0.71. Tibiotarsal smooth chaeta about 0.92 smaller than unguiculus; tenent hair acuminate and about 0.54 smaller than unguis outer edge.

Collophore (Figure 71F). Anterior side with 6 ciliate apically acuminate chaetae, 3 inner larger, others subequal; posterior side distally with 1 smooth chaeta, 3 ciliate chaetae and 1 subdistal reduced spine; lateral flap with 10 smooth chaetae (3 larger).

Furcula. Only with ciliate chaetae and scales. Manubrium ventrally with 3 subapical and 9 distal scales; manubrial plate with 5 ciliate chaetae (2 inner mac) and 2 psp (Figure 71G and H). Mucro teeth subequal in size, basal spine reaching the apex of basal tooth (Figure 71I).

*Etymology.* Refers to macrochaetae pattern in tree shape on head series “A” of the new species (from Latin: ligni-tree, woods; from Greek: cephala-head) (Figure 69E).

*Remarks. Pseudosinella macrolignicephala* sp. nov. resembles *P. flatua* Christiansen and Bellinger, 1996, *P. granda* Christiansen and Bellinger, 1996, and *P. pecki* Christiansen and Bellinger, 1980 in Th II–Abd III with 0, 0, 0, 1, and 0 central mac, tenent hair acuminate, unguis lamellae wide and with 3 inner teeth (a.t. absent), and unguiculus pe lamellae smooth (Table 1, Table 4 and Table 6). However, *P. macrolignicephala* sp. nov. clearly differs from these species by head M2 mac present (absent in the other species), Abd IV with 3 central mac (C1 present), while in the other species only 2 central mac are present (B5–6). They still differ by basal teeth unequal (equal in *P. flatua* and *P. pecki*), mucro spine reaching the apex of basal tooth (surpasses in *P. flatua* and *P. granda*), collophore anteriorly with 6 chaetae and only smooth chaetae on lateral flap, while in *P. flatua* and *P. granda* have 11–13 and 9 anterior chaetae, respectively, and at least 5 ciliate chaetae on lateral flap [5,6,7]. *Pseudosinella macrolignicephala* sp. nov. also resembles other species of Table 6, especially in prelabral chaetae bifurcate, head with 4 mac (A0, A2–3, and M2), and Th II–Abd IV with 0, 0, 0, 1, 0, and 3 central mac. However, *P. macrolignicephala* sp. nov. is different from such species by papilla E with l.p. pointed (finger-shaped in others species), basomedian and basolateral labial fields with a1–5 smooth, tenent hair acuminate and unguis a.t. teeth absent.

#### 3.4.4. *Pseudosinella parambigua* sp. nov. Oliveira, Lima, and Cipola

**Figure 72:**
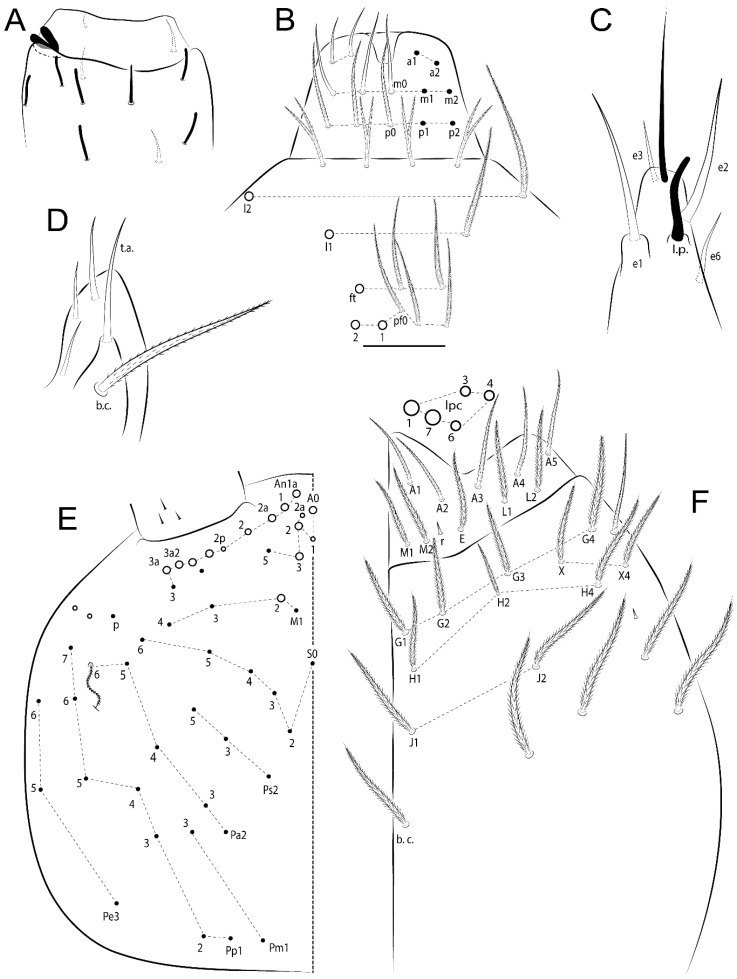
*Pseudosinella parambigua* sp. nov.: head. (**A**) Ant III apical organ (lateral view); (**B**) chaetotaxy of clypeus, prelabrum, and labrum; (**C**) labial papilla E (right side); (**D**) maxillary palp and sublobal plate (left side); (**E**) head dorsal chaetotaxy (left side); (**F**) labial proximal chaetae, basomedian and basolateral labial fields, and complete postlabial chaetotaxy (right side).

**Figure 73:**
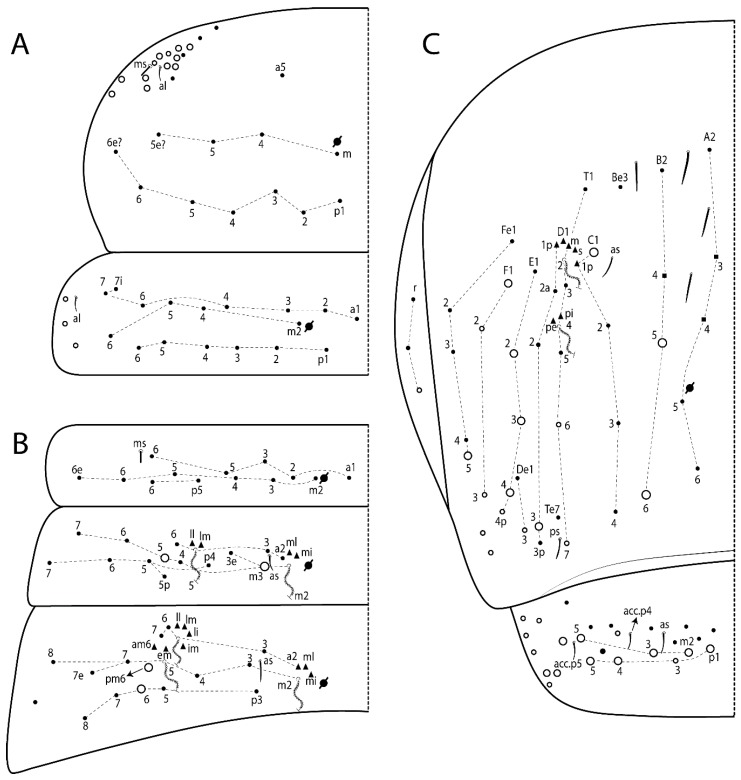
*Pseudosinella parambigua* sp. nov.: dorsal chaetotaxy. (**A**) Th II–III; (**B**) Abd I–III; (**C**) Abd IV–V.

**Figure 74:**
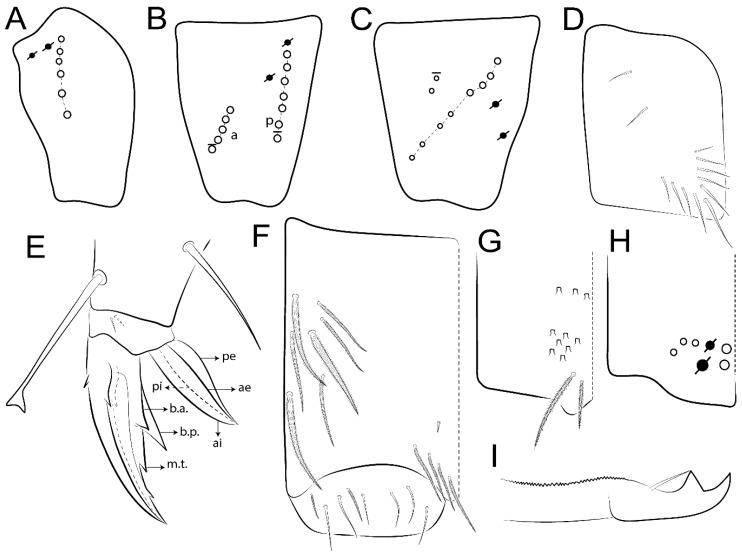
*Pseudosinella parambigua* sp. nov. (**A–C**) subcoxa I–III, respectively; (**D**) trochanteral organ; (**E**) distal tibiotarsus and empodial complex III (anterior view); (**F**) collophore (lateral view); (**G**) distal part of manubrium (ventral view); (**H**) manubrial plate (dorsal view); (**I**) distal dens and mucro (outer view).

Figure 72, Figure 73 and Figure 74, Table 1 and Table 6

*Type material.* Holotype female in slide (5295/CRFS-UEPB): Brazil, Minas Gerais State, Nova Lima municipality, near “Mina D’Água”, 19°56′45.1″ S, 43°52′28.7″ W, 1052 m, 25.v.2014, Bioespeleo et al. coll. Paratypes in slide (5119–20, 5122/CRFS-UEPB): 2 females and 1 juvenile, same data as holotype, except, 13.i.2014. Paratype in slides (5623/CRFS-UEPB): 1 juvenile, same data as holotype, except, 19°56′49.1″ S, 43°52′55.1″ W, 1216 m, 20.v.2014. Paratype in slide (6346/CRFS-UEPB donated to INPA/096): 1 female, same data as holotype, except next to road BR-356, 20°06′34.1″ S, 43°58′31.9″ W, 1341 m, 20.xi.2014.

*Description.* Total length (head + trunk) of specimens 0.84–1.10 mm (n = 3), holotype 0.84 mm.

Head. Ratio antennae:trunk = 1:2.60–2.73 (n = 2), holotype 1:2.60; Ant III smaller than Ant II length; Ant segment ratio as I:II:III:IV = 1:1.64–1.80:1.18–1.39:2.50–3.30, holotype 1:.80:1.18:2.50. Ant IV dorsally with numerous sens (b–c) and chaetae (i–k). Ant III with 2 apical sens clubs surrounded by at least 5 c and 6 d (Figure 72A). Ant II with common sens b–c, with 3 b, 1 e dorso-distal, 1 b dorso-lateral and 4 d, and 2 b ventro-subdistal. Clypeal formula with 4 (l1–2), 3 (ft), and 5 (pf0–2) ciliate chaetae, all apically acuminate, l1–2 larger (Figure 72B). Prelabral chaetae ciliated discreetly and bifurcate. Labral chaetae smooth, no modifications, except p0–2 chaetae discreetly ciliate (Figure 72B). Labial papilla D with 3 appendages; papilla E with l.p. finger-shape, curved, and exceed the apical appendage (Figure 72C). Maxillary palp with b.c. weakly ciliate and 1.17 larger than the t.a. (Figure 72D). Dorsal head chaetotaxy (Figure 72E) with 10 “An” (An1a–3), 6 “A” (A0–3 and A5), 4 “M” (M1–4), 6 “S” (S0 and S2–6), 3 “Ps” (Ps2–3 and Ps5), 4 “Pa” (Pa2–5), 2 “Pm” (Pm1 and Pm3), 7 “Pp” (Pp1–7), and 3 “Pe” (Pe3 and Pe5–6) chaetae; An1a–3a, A0, A2–3, and M2 as mac; interocular p mic present; head posterior region with 10 cervical like-spine mac (not represented in Figure 72E). Basomedian and basolateral labial fields with A1–5 discretely ciliate; M1–2, E, and L1–2 multiciliate; r reduced (Figure 72F). Labial proximal chaetae smooth (lpc1, 3–4, and 6–7), lpc3–4 and lpc6 gently smaller than others. Ventral chaetotaxy with about 17 ciliate chaetae and 1 reduced lateral spine, postlabial formula 4 (G1–4), 1 (X4), 4 (H2–4), and 2 (J1–2) ciliate chaetae, b.c. present (Figure 72F).

Thorax dorsal chaetotaxy (Figure 73A). Th II a, m, and p series with 1 (a5), 4 (m and m4–5e?), and 7 (p1–6e?) mic, respectively. Th III a, m, and p series with 7 (a1–a4 and a6–7i), 4 (m2 and m4–6), and 6 (p1–6) mic, respectively. Ratio Th II:III = 2.00–2.42:1 (n = 3), holotype 2.01:1.

Abdomen dorsal chaetotaxy (Figure 73B,C). Abd I a, m, and p series with 5 (a1–3 and a5–6), 6 (m2–6e), and 2 (p5–6) mic, respectively. Abd II a, m, and p series with 3 (a2–3 and a6), 6 (m3–7), and 5 (p4–7) chaetae, respectively, m3 and m5 as mac; a5 and m2 bothriotricha with 2 (ll, lm) and 2 (ml, mi) fan-shaped chaetae, respectively. Abd III a, m and p series with 4 (a2–3 and a6–7), 7 (m3–4, am6, pm6, and m7–8) and 5 (p3 and p5–8) chaetae, respectively, pm6 and p6 as mac, and as sens elongated, ms absent; m2 bothriotricha with 3 (a2, ml, and mi) and a5 and m5 bothriotrichum with 6 (am6, ll, lm, li, em, and im) fan-shaped chaetae. Abd IV A–Fe series with 5 (A2–6), 4 (B2 and B4–6), 1 (Be3), 5 (C1–4), 5 (T1, T3, and T5–T7), 1 (Te7), 6 (D1–3p), 2 (De1 and De3), 5 (E1–4p), 3 (F1–3), and 5 (Fe1–5) chaetae, respectively, with 3 central (B5–6 and C1), and 6 lateral mac (D3, E2–4, F1, and Fe5); T2 and T4 bothriotricha with 5 (D1–1p, m, s, and C1p) and 2 (pe and pi) fan-shaped chaetae, respectively; 6 sens present, ps type I, as and 4 of uncertain homology type II, r series laterally with 3 chaetae. Abd V m and p series with 2 (m2–3 and m5) and 3 (p1 and p4–5) central mac, respectively. Ratio Abd III:IV = 1:2.61–3.56 (n = 3), holotype 1:3.56. Th II–Abd IV formula with 00|010 + 21 + 2 mac.

Legs. Subcoxa I with “p” row with 6 chaetae and 2 psp; subcoxa II with “a” row with 4–5 chaetae, “p” row with 6–7 chaetae and 2 psp; subcoxa III with one row of 8 chaetae plus 1–2 anterior chaetae and 2 psp (Figure 74A–C). Trochanteral organ with 12 spine-like chaetae (Figure 74D). Tibiotarsus I–III formula with 1, 2, and 3 mac type III (as Figure 51E). Unguis outer side with paired teeth straight on proximal one third; inner side with wide lamella and 4 teeth, basal pair (b.a. and b.p.) unequal, b.p larger but not reaching the m.t. apex, m.t. on distal one third and subequal to b.a., a.t. minute on distal one sixth. Unguiculus with all lamella smooth and acuminate (ai, ae, pi, and pe) (Figure 74E); ratio unguis:unguiculus in holotype = 1:0.61. Tibiotarsal smooth chaeta about 0.84 smaller than unguiculus; tenent hair capitate and about 0.65 smaller than unguis outer edge.

Collophore (Figure 74F). Anterior side with 7 ciliate apically acuminate chaetae, 3 inner larger, others subequal in length; posterior side distally with 4 weakly ciliated chaetae and 1 subdistal reduced spine; lateral flap with 7 smooth chaetae and 2 weak ciliate chaetae (2 larger).

Furcula. Only with ciliate chaetae and scales. Manubrium ventrally with 3 subapical and 7 distal scales; manubrial plate with 5 ciliate chaetae (2 inner mac) and 2 psp (Figure 74G–H). Mucro basal tooth larger than distal tooth, basal spine reaches and sometimes exceeds the apex of basal tooth (Figure 74I).

*Etymology.* Refers to similar morphology compared to *P. ambigua*.

*Remarks. Pseudosinella parambigua* sp. nov. resembles *P. ambigua* and *P. chimerambigua* sp. nov. (Table 1 and Table 6). However, *P. parambigua* sp. nov. differ from these species by labral a1–2 chaetae smooth (with filaments in *P. chimerambigua* sp. nov.); basomedian and basolateral labial fields with A1–5 discretely ciliate; and M1–2, E, and L1–2 plus postlabial G1–4 multiciliate, while in *P. chimerambigua* sp. nov. have all unilaterally ciliate chaetae. On Abd II, m3e mac is absent in *P. parambigua* sp. nov. and present in *P. ambigua* [14]. *Pseudosinella parambigua* sp. nov. also differ from these species by unguis basal teeth unequal (equal in both species), unguiculus pe lamella smooth (serrate in both species), collophore posteriorly devoid of smooth chaetae and with 1 spine (1 smooth and 2 spines in *P. ambigua*), and manubrial plate with 3 outer chaetae (2 in *P. chimerambigua*). See also the comparison these species in remarks of *P. chimerambigua* sp. nov. and Table 1 and Table 6.

#### 3.4.5. *Pseudosinella phyllunguiculata* sp. nov. Oliveira, Lima, and Cipola

**Figure 75:**
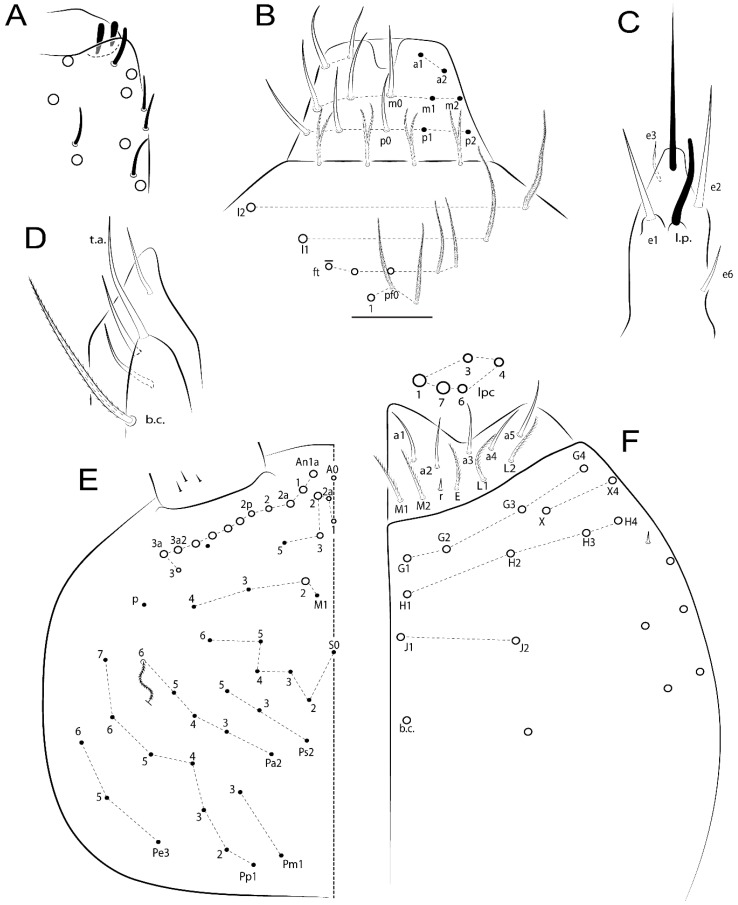
*Pseudosinella phyllunguiculata* sp. nov.: head. (**A**) Ant III apical organ (lateral view); (**B**) chaetotaxy of clypeus, prelabrum, and labrum; (**C**) labial papilla E; (**D**) maxillary palp and sublobal plate (left side); (**E**) head dorsal chaetotaxy (left side); (**F**) labial proximal chaetae; basomedian and basolateral labial fields; and complete postlabial chaetotaxy (right side).

**Figure 76:**
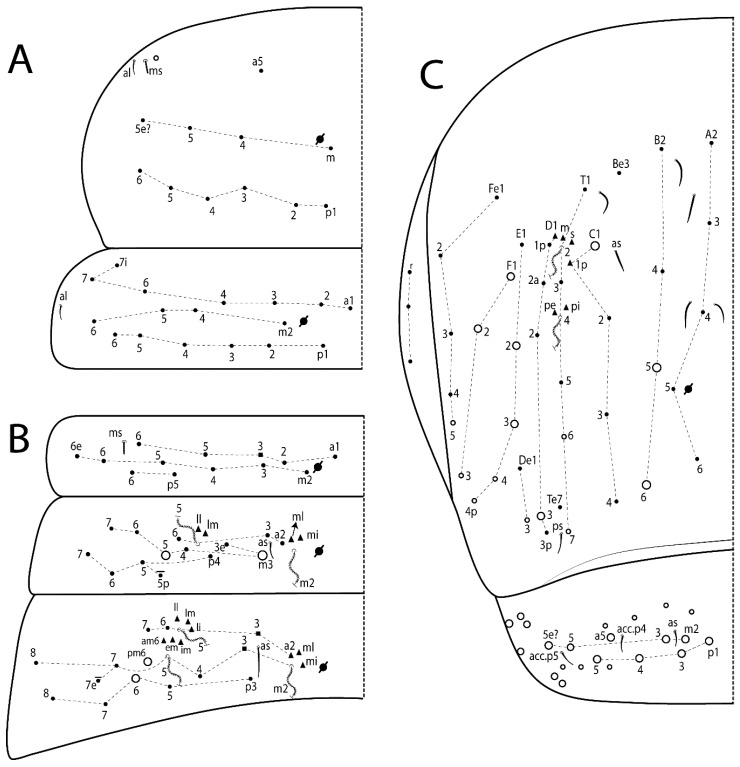
*Pseudosinella phyllunguiculata* sp. nov.: dorsal chaetotaxy. (**A**) Th II–III; (**B**) Abd I–III; (**C**) Abd IV–V.

**Figure 77:**
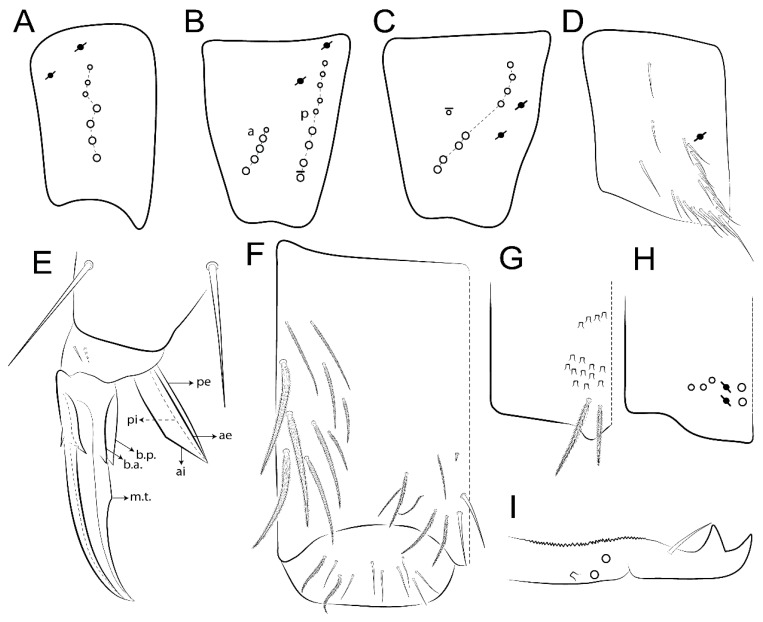
*Pseudosinella phyllunguiculata* sp. nov.: (**A–C**) subcoxa I–III, respectively; (**D**) trochanteral organ; (**E**) distal tibiotarsus and empodial complex III (anterior view); (**F**) collophore (lateral view); (**G**) distal part of manubrium (ventral view); (**H**) manubrial plate (dorsal view); (**I**) distal dens and mucro (outer view).

Figure 75, Figure 76 and Figure 77, Table 1 and Table 6

*Type material.* Holotype female in slide (7352/CRFS-UEPB): Brazil, Minas Gerais State, Barão dos Cocais municipality, “between Salão das Pedras and Cachoeira Água Fria”, 19°55′50.7″ S, 43°31′46.3″ W, 1195 m, 18.ix.2014, Ativo Ambiental et al. coll. Paratypes in slides (7353–54, 1344–45, 1347, 1408–09/CRFS-UEPB): 2 males and 5 females, same data as holotype, except “Nexto to Cachoeira da Lajeado and Cachoeira Água Fria”, 19°55′25.8″ S, 43°29′57.1″ W, 975 m; 19°55′43.6″ S, 43°30′25.8″ W, 968 m; 19°55′40.9″ S, 43°30′28.5″ W, 984 m, and 19°55′45.6”S, 43°30′35.6″ W, 948 m, respectively, 10-21.iii.2009 and 22.vi-03.vii.2009. Paratypes in slides (1349, 1351/CRFS-UEPB donated to INPA/097): 2 females, same data as holotype, except 19°55′45.0″ S, 43°30′45.6″ W, 968 m, 10-21.iii.2009 and 22.vi-03.vii.2009.

*Description.* Total length (head + trunk) of specimens 0.95–1.30 mm (n = 5), holotype 1.30 mm.

Head. Ratio antennae:trunk = 1:2.10–3.16 (n = 5), holotype 1:2.24; Ant III smaller than Ant II length; Ant segment ratio as I:II:III:IV = 1:1.61–2.54:1.44–1.59:3.35–5.22, holotype 1:2.50:1.47:3.35. Ant IV dorsally with numerous sens (b–c) and chaetae (i–k). Ant III with 2 apical sens clubs surrounded by at least 1 sens d and 4 c (Figure 75A). Ant II with common sens b–c, with 1 b and 1 e dorso-distal, 2 b and 1 c dorso-lateral, and 3 b and 1 e ventro-subdistal sens. Clypeal formula with 4 (l1–2), 3–5 (ft), and 3 (pf0–1) ciliate chaetae; all apically acuminate; and l1–2 larger (Figure 75B). Prelabral chaetae weakly ciliate and bifurcate. Labral chaetae smooth, no modifications (Figure 75B). Labial papilla D with 3 appendages; papilla E with l.p. finger-shape, sinuous, and exceeding the base of apical appendage (Figure 75C). Maxillary palp with b.c. weakly ciliate and 1.5 longer than the t.a. (Figure 75D). Dorsal head chaetotaxy (Figure 75E) with 12 “An” (An1a–3), 6 “A” (A0–3 and A5), 4 “M” (M1–4), 6 “S” (S0 and S2–6), 3 “Ps” (Ps2–3 and Ps5), 4 “Pa” (Pa2–5), 2 “Pm” (Pm1 and Pm3), 7 “Pp” (Pp1–7), and 3 “Pe” (Pe3, Pe5, and Pe6) chaetae; An1a–3a, A2–A3, and M2 as mac; interocular p mic present; head posterior region with 7–9 cervical like-spine mac (not represented in Figure 75E). Basomedian and basolateral labial fields with a1–5 smooth chaetae; M1–2, E, and L1–2 unilaterally ciliate; and r reduced (Figure 75F). Labial proximal chaetae smooth (lpc1, 3–4, and 6–7), lpc3–4, and lpc6 smaller. Ventral chaetotaxy with about 19 ciliate chaetae and 1 reduced lateral spine, postlabial formula 4 (G1–4), 2 (X–X4), 4 (H1–4), and 2 (J1–2) ciliate chaetae, b.c. present (Figure 75F).

Thorax dorsal chaetotaxy (Figure 76A). Th II a, m, and p series with 1 (a5), 4 (m and m4–5e?), and 6 (p1–6) mic, respectively. Th III a, m, and p series with 7 (a1–4 and a6–7i), 4 (m2 and m4–6), and 6 (p1–6) mic, respectively. Ratio Th II:III = 1.57–2.92:1 (n = 5), holotype 1.57:1.

Abdomen dorsal chaetotaxy (Figure 76B,C). Abd I a, m, and p series with 5 (a1–3 and a5–6), 6 (m2–6e), and 2 (p5–6) mic, respectively. Abd II a, m, and p series with 3 (a2–3 and a6), 6 (m3–7), and 4–5 (p4–7) chaetae, respectively, m3 and m5 as mac; a5 and m2 bothriotricha with 2 (lm and ll) and 2 (ml and mi) fan-shaped chaetae, respectively. Abd III a, m, and p series with 4 (a2–3 and a6–7), 6–7 (m3–4, am6, pm6, and m7–8) and 5 (p3 and p5–8) chaetae, respectively, pm6 and p6 as mac, a3 and m3 as reduced mic, and as sens elongated, ms absent; m2 bothriotrichum associated with 3 (a2, ml, and mi) and a5 and m5 bothriotricha with 6 (ll, lm, li, am6, em, and im) fan-shaped chaetae. Abd IV A–Fe series with 5 (A2–6), 4 (B2 and B4–6), 1 (Be3), 5 (C1–4), 5 (T1, T3, and T5–7), 1 (Te7), 6 (D1–3p), 2 (De1 and De3), 5 (E1–4p), 3 (F1–3), and 5 (Fe1–5) chaetae, respectively, with 3 central (B5–6 and C1) and 5 lateral mac (D3, E2–3, and F1–2); T2 and T4 bothriotricha with 4 (D1, m, s, and C1p) and 2 (pe and pi) fan-shaped chaetae, respectively; 7 sens present, ps type I, as and 5 of uncertain homology type II, r series laterally with 3 chaetae. Abd V a, m, and p series with 1 (a5), 4 (m2–3 and m5–5e), and 4 (p1 and p3–5) central mac, respectively. Ratio Abd III:IV = 1:3.48–4.48 (n = 5), holotype 1:4.03. Th II–Abd IV formula with 00|010 + 21 + 2 mac.

Legs. Subcoxa I with “p” row with 7 chaetae and 2 psp; subcoxa II with “a” row with 5 chaetae, “p” row with 8–9 chaetae and 2 psp; subcoxa III with one row of 8–9 chaetae, plus 1 anterior chaeta present or absent and 2 psp (Figure 77A–C). Trochanteral organ with about 19 spine-like chaetae and 1 psp (Figure 77D). Tibiotarsus I–III formula with 1, 2, and 3 mac type III (as Figure 51E). Unguis outer side with paired teeth straight and not developed on proximal one third; inner side with slender lamella and 2 small basal teeth (b.a. and b.p.), m.t. vestigial on basal half, a.t. absent. Unguiculus with all lamellae acuminate (ai, ae, pi, and pe), except ai truncate (Figure 77E); ratio unguis:unguiculus in holotype = 1:0.57. Tibiotarsal smooth chaeta about 0.7 smaller than unguiculus; tenent hair acuminate and about 0.58 smaller than unguis outer edge.

Collophore (Figure 77F). Anterior side with 10 ciliate apically acuminate chaetae, 3 inner larger, and others subequal in length; posterior side distally with 2 smooth chaetae, 6 ciliate chaetae, and 1 subdistal reduced spine; lateral flap with 8 smooth and 2 ciliate chaetae (2 larger).

Furcula. Only with ciliate chaetae and scales. Manubrium ventrally with 4 subapical and 11 distal scales; manubrial plate with 5 ciliate chaetae (2 inner mac) and 2 psp (Figure 77G,H). Mucro teeth subequal in size, spine reaching the apex of basal tooth (Figure 77I).

*Etymology.* Refers to tree leaf-shape of the unguiculus in new species (from Greek: phyllum–leaf).

*Remarks. Pseudosinella phyllunguiculata* sp. nov. resembles *P. brevicornis* [12] by eyes and pigments absent and unguiculus ai lamellae truncate, but the new species differs in unguis m.t. and a.t. teeth absent (developed in *P. brevicornis*) and tenent hair smaller than unguis length (larger in *P. brevicornis*).

#### 3.4.6. *Pseudosinella prelabruscervata* sp. nov. Oliveira, Lima, and Cipola

**Figure 78:**
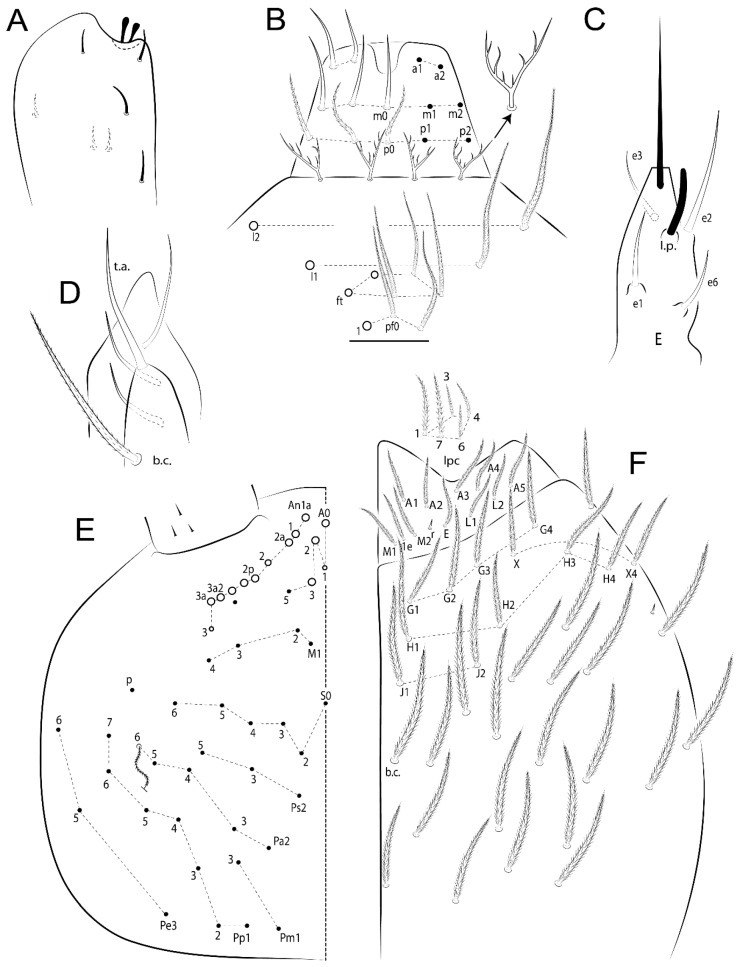
*Pseudosinella prelabruscervata* sp. nov.: head. (**A**) Ant III apical organ (lateral view); (**B**) chaetotaxy of clypeus, prelabrum, and labrum; (**C**) labial papilla E; (**D**) maxillary palp and sublobal plate (left side); (**E**) head dorsal chaetotaxy (left side); (**F**) labial proximal chaetae, basomedian, and basolateral labial fields and complete postlabial chaetotaxy (right side).

**Figure 79:**
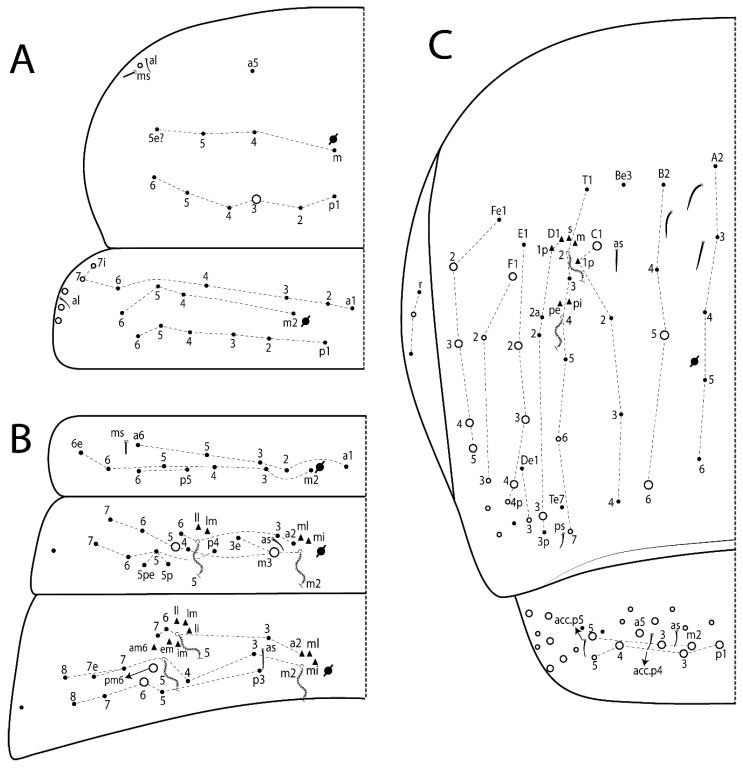
*Pseudosinellaprelabruscervata* sp. nov.: dorsal chaetotaxy. (**A**) Th II–III; (**B**) Abd I–III; (**C**) Abd IV–V.

**Figure 80:**
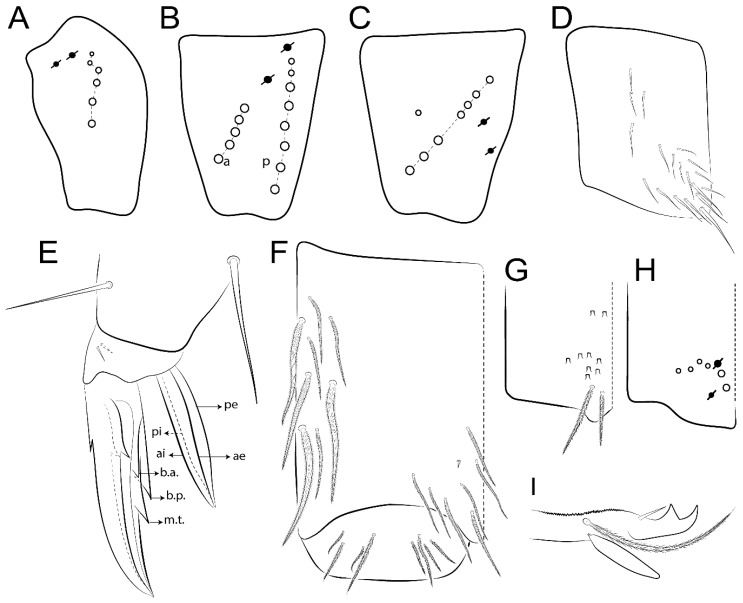
*Pseudosinella prelabruscervata* sp. nov. (**A–C**) subcoxa I–III, respectively; (**D**) trochanteral organ; (**E**) distal tibiotarsus and empodial complex III (anterior view); (**F**) collophore (lateral view); (**G**) distal part of manubrium (ventral view); (**H**) manubrial plate (dorsal view); (**I**) distal dens and mucro (outer view).

Figure 78, Figure 79 and Figure 80, Table 1 and Table 6

*Type material.* Holotype female in slide (10422/CRFS-UEPB): Brazil, Minas Gerais State, Rio Acima municipality, next to “Serra do Gandarela”, 20°09′08.5″ S, 43°39′04.8″ W, 1299 m, 14.vii-18.ix.2016, Carste et al. coll. Paratypes in slides (10405, 10418, 10428/CRFS-UEPB): 3 females, same data as holotype, except 20°09′01.2″ S, 43°39′15.3″ W, 1395 m; 20°08′54.9″ S, 43°39′16.6″ W, 1430 m, and 20°09′01.3″ S, 43°39′15.1″ W, 1394 m, respectively. Paratype in slide (10421/CRFS-UEPB donated to INPA/098): 1 female same data as holotype, except 20°09′08.5″ S, 43°39′04.8″ W, 1299 m.

*Description.* Total length (head + trunk) of specimens 1.24–1.28 mm (n = 4), holotype 1.27 mm.

Head. Ratio antennae:trunk = 1:2.00–3.52 (n = 4), holotype 1:2.42; Ant III smaller than Ant II length; Ant segment ratio as I:II:III:IV = 1:1.59–3.21:1.00–3.00:2.52–5.75, holotype 1:2.66:2.81:5.16. Ant IV dorsally with numerous sens (b–c) and chaetae (i–k). Ant III with 2 apical sens clubs surrounded by at least 7 sens c (Figure 78A). Ant II with common sens b–c, 2 c dorso-distal, 2 c dorso-lateral, and 2 c and 1 e ventro-subdistal sens. Clypeal formula with 4 (l1–2), 5 (ft), and 3 (pf0–1) ciliate chaetae, all apically acuminate, l1–2 larger, and others subequal (Figure 78B). Prelabral chaetae with elongated cilia on branches bifurcate. Labral chaetae smooth, no modifications, except p0–2 chaetae weakly ciliate (Figure 78B). Labial papilla D with 3 appendages; papilla E with l.p. finger-shape, curved, and reaching the base of apical appendage (Figure 78C). Maxillary palp with b.c. weakly ciliate and 1.12 longer than the t.a. (Figure 78D). Dorsal head chaetotaxy (Figure 78E) with 10 “An” (An1a–3), 5 “A” (A0–3 and A5), 4 “M” (M1–4), 6 “S” (S0 and S2–6), 3 “Ps” (Ps2–3 and Ps5), 4 “Pa” (Pa2–5), 2 “Pm” (Pm1 and Pm3), 7 “Pp” (Pp1–7), and 3 “Pe” (Pe3 and Pe5–6) chaetae; An1a–3a, A0, A2, and A3 as mac; interocular p mic present; head posterior region with 7–9 cervical like-spine mac (not represented in Figure 78E). Basomedian and basolateral labial fields with A1–5, M1–1e, M2, E, and L1–2 multiciliate; r reduced; and M1 subequal to M1i in length (Figure 78F). Labial proximal chaetae ciliate (lpc1, 3–4, and 6–7), lpc3, lpc4, and lpc6 smaller. Ventral chaetotaxy with about 33 ciliate chaetae and 1 reduced lateral spine, postlabial formula 4 (G1–4), 2 (X and X4), 4 (H1–4), and 2 (J1–2) ciliate chaetae, b.c. present (Figure 78F).

Thorax dorsal chaetotaxy (Figure 79A). Th II a, m, and p series with 1 (a5), 4 (m and m4–5e?), and 5 (p1–2 and p4–6) mic, p3 as mac. Th III a, m, and p series with 7 (a1–4 and a6–7i), 4 (m2 and m4–6), and 6 (p1–6) chaetae, respectively. Ratio Th II:III = 1.75–2.30:1 (n = 4), holotype 2.11:1.

Abdomen dorsal chaetotaxy (Figure 79B,C). Abd I a, m, and p series with 5 (a1–3 and a5–6), 6 (m2–6e), and 2 (p5–6) mic, respectively. Abd II a, m and p series with 3 (a2–3 and a6), 6 (m3–7), and 6 (p4–7) chaetae, respectively, m3 and m5 as mac; a5 and m2 bothriotricha with 2 (ll and lm) and 2 (ml and mi) fan-shaped chaetae, respectively. Abd III a, m, and p series with 4 (a2–3 and a6–7), 7 (m3–4, am6, pm6, and m7–8) and 5 (p3 and p5–8) chaetae, respectively, pm6 and p6 as mac, a3 and m3 mic normal and as sens elongated, ms absent; m2 bothriotrichum with 3 (a2, ml, and mi) and a5 and m5 bothriotricha with 6 (ll, lm, li, am6, em, and im) fan-shaped chaetae. Abd IV A–Fe series with 5 (A2–6), 4 (B2 and B4–6), 1 (Be3), 5 (C1–4), 5 (T1, T3, and T5–7), 1 (Te7), 6 (D1–3p), 2 (De1 and De3), 5 (E1–4p), 3 (F1–3), and 5 (Fe1–5) chaetae, respectively, with 3 central (B5–6 and C1) and 9 lateral mac (D3, E2–4, F1, and Fe2–5); T2 and T4 bothriotricha with 5 (D1–1p, s, m, and C1p) and 2 (pe and pi) fan-shaped chaetae, respectively; 5 sens present, ps type I, as and 3 of uncertain homology type II, r series laterally with 3 chaetae. Abd V a, m, and p series with 1 (a5), 3 (m2–3 and m5) and 3 (p1 and p3–4) central mac, respectively. Ratio Abd III:IV = 1:2.83–3.35 (n = 4), holotype 1:2.83. Th II–Abd IV formula with 10|010 + 21 + 2 mac.

Legs. Subcoxa I with “p” row with 6 chaetae and 2 psp; subcoxa II with “a” row with 5 chaetae, “p” row with 8 chaetae and 2 psp; subcoxa III with one row of 7 plus 1 anterior chaeta and 2 psp (Figure 80A–C). Trochanteral organ with 19 spine-like chaetae (Figure 80D). Tibiotarsus I–III formula with 1, 1, and 2 mac type III (as Figure 51E). Unguis outer side with paired teeth straight on proximal one third; inner side with wide lamella and 3 teeth, basal pair (b.a. and b.p.) unequal, b.p. larger but not reaching the m.t. apex, m.t. on little more than distal half and subequal to b.p., a.t. absent. Unguiculus with all lamella smooth and acuminate (ai, ae, pi, and pe) (Figure 80E); ratio unguis:unguiculus in holotype = 1:0.57. Tibiotarsal smooth chaeta about 0.71 smaller than unguiculus; tenent hair acuminate and about 0.62 smaller than unguis outer edge.

Collophore (Figure 80F). Anterior side with 8 ciliate chaetae apically acuminate, 4 larger, others subequal in lenght; posterior side distally with 7 weakly ciliated chaetae and 1 subdistal reduced spine; lateral flap with 8 ciliate chaetae (3 larger).

Furcula. Only with ciliate chaetae and scales. Manubrium ventrally with 2 subapical and 7 distal scales; manubrial plate with 6 ciliate chaetae (2 inner mac) and 2 psp (Figure 80G,H). Mucro teeth subequal in size, basal spine not reaching the apex of basal tooth (Figure 80I).

*Etymology.* Refers to ramification of prelabral chaetae resembling a deer’s antlers (Figure 78B).

*Remarks. Pseudosinella prelabruscervata* sp. nov. resembles *P. alba* (Packard, 1873) [7] and *P. cearensis* sp. nov. in head with 3 mac (A0 and A2–3), Th II–Abd III with 1, 0, 0, 1, and 0 central mac, and unguiculus pe lamella toothless (Table 1, Table 5, and Table 6). However, *P. prelabruscervata* sp. nov. differs from these species by eyes absent (2 + 2 in *P. alba*), head S3 mac absent (present in *P. alba*), labral a1–2 and m0–2 chaetae smooth (with filaments in *P. cearensis* sp. nov.), and prelabral chaetae with elongated cilia over the bifurcate branches (no bifurcate and weakly ciliate in both species), of which the characteristic within Entomobryidae so far is exclusive of new species.

### 3.5. Key to Eyeless Pseudosinella Species Recorded from Brazil

1. Unguiculus outer lamella (pe) with robust tooth (Figure 7B and Figure 10H) … 2
  - Unguiculus with all lamellae acuminate (Figure 39F and Figure 45F) … 12
2. Head and Abd II with Pa5 and a2 mac, respectively (Figure 6C and Figure 7A); basomedian labial field devoid of M2 chaeta; and manubrium dorsally and dens basis with smooth chaetae (Figure 4D) … 3
  - Head and Abd II with Pa5 and a2 mic, respectively (Figure 8E and Figure 9B); basomedian labial field with M1 and M2 chaetae (Figure 8F and Figure 13B); and manubrium and dens dorsally only with ciliate chaetae … 4
3. Ant III with 1 conical sens next to sense organ; Ant II with 2 lateroventral sens apically rounded; and tenent hair acuminate (Figure 4C) … *P. biunguiculata*
  - Ant III with 3 conical sens next to sense organ; Ant II with 2 lateroventral sens apically capitate; and tenent hair capitate (Figure 7B) … *P. guanhaensis*
4. Th II with 1–2 posterior mac (p3 and p5) (Figure 14A and Figure 32A) … 5
  - Th II only with posterior mic (Figure 9A) … 8
5. Th II with 2 posterior mac; Th III with 2 posterior mac (p2–3) (Figure 14A); and tenent hair acuminate (Figure 15F) … 6
  - Th II with 1 posterior mac; Th III only with mic (Figure 32A); and tenent hair capitate (Figure 33B) … 7
6. Labral chaetae smooth, subequal, and lacking filaments (Figure 12D); labial papilla B with smooth appendages (Figure 12E); dorsal head with M2 mac (Figure 13A); Abd IV without F1 mac (Figure 14C); and collophore lateral flap chaetae smooth and ciliate (Figure 15H) … *P. brumadinhoensis* sp. nov.
  - Labral a1–2 and m0–2 chaetae thicker and with median filaments, p0–2 chaetae ciliate (Figure 22B); labial papilla B with median filaments in b4 appendage (Figure 22C); dorsal head with M2 mic (Figure 22D); Abd IV with F1 mac (Figure 23C); and collophore lateral flap chaetae with elongated cilia (Figure 24D) … *P. labruspinata* sp. nov.
7. Labral a1–2 chaetae thicker as horn (Figure 31B); Th II with p3 mac and p5 mic (Figure 32A); unguis b.p. tooth undivided, median tooth away from the basal teeth, apical tooth absent (Figure 33B,C); and collophore anteriorly with 4 chaetae (Figure 33D) … *P. taurina* sp. nov.
  - Labral a1–2 and m0–2 chaetae thicker and with median filaments (Figure 43B); Th II with p3 mic and p5 mac (Figure 35A); unguis b.p. tooth with 1 smaller split tooth posteriorly, median tooth between basal teeth, apical tooth minute (Figure 36B,C); and collophore anteriorly with at least 6 chaetae (Figure 36D) … *P. unimacrochaetosa* sp. nov.
8. Prelabral chaetae ciliate; labral a1–2 and m0–2 chaetae thicker and generally with median filaments (at least 1 in m1–2 chaetae) (Figure 19B and Figure 28C); labial proximal chaetae with median filaments, basomedian, and basolateral labial fields with A1–4, M1–2, E, and L1–2 unilaterally and/or weakly ciliate (Figure 8F and Figure 19F); Abd IV with B6 mac (Figure 9C); and collophore anteriorly with 6 chaetae (Figure 11A) …
  - Prelabral chaetae smooth, labral chaetae smooth and without modifications (Figure 25B); labial proximal chaetae smooth, basomedian, and basolateral labial fields with a1–5 smooth and M1–2, E, and L1–2 clearly multiciliate (Figure 25E); Abd IV with B6 mic (Figure 26C); and collophore anteriorly with 4 chaetae (Figure 27D) … *P. paraensis* sp. nov.
9. Dorsal head with M2 mac (Figure 8E) and unguis b.p. tooth undivided, m.t. present (Figure 10H,I) … 10
  - Dorsal head with M2 mic (Figure 16D) and unguis b.p. tooth with 1 smaller split tooth posteriorly, m.t. absent (Figure 18B,C) … 11
10. Ant II distally without f and e sens; labral a2 and m0 chaetae with at least 1 median filament (Figure 8B); labial papilla E with l.p. finger-shape and sinuous (Figure 8C); and tenent hair capitate, unguis b.p. tooth not reaching the m.t. apex, and unguiculus ai lamella acuminate (Figure 10H,I) … *P. acantholabrata* sp. nov.
  - Ant II distally with 2 sens f and 1 e on dorsal side (Figure 28B); labral a2 and m0 chaetae smooth (Figure 28C); labial papilla E with l.p. conical and straight (Figure 28D); and tenent hair acuminate, unguis b.p. tooth surpass the m.t. apex, and unguiculus ai lamella gently excavate distally (Figure 30B) … *P. serpentinensis* sp. nov.
11. Labial papilla E with l.p. finger-shaped (Figure 16C); basomedian and basolateral labial fields with all chaetae unilaterally and weakly ciliate (Figure 16E); unguiculus ai lamella gently excavate distally (Figure 18B); and collophore posteriorly with 2 + 2 ciliate chaetae and lateral flap only with smooth chaetae (Figure 18D) … *P. keni* sp. nov.
  - Labial papilla E with l.p. pointed (Figure 19C), basomedian and basolateral labial fields with all chaetae clearly ciliate (Figure 19F); unguiculus ai lamella gently truncate (Figure 21B); and collophore posteriorly without ciliate chaetae and lateral flap with smooth and ciliate chaetae (Figure 21D) … *P. labiociliata* sp. nov.
12. Tenent hair smaller than unguis length, if subequal, then apically capitate and unguiculus ai lamella acuminate (e.g., Figure 63F), or distally gently excavate (Figure 39F) or truncate (Figure 60F and Figure 77E) … 13
  - Tenent hair acuminate and subequal to unguis length and unguiculus ai lamella (ai) clearly truncate on proximal half … *P. brevicornis* Handschin, 1924
13. Prelabral chaetae normal, undivided (Figure 37B and Figure 43B) … 14
  - Prelabral chaetae bifurcate (Figure 64A and Figure 78B) … 22
14. Th II with posterior mac (Figure 44A and Figure 53A) and labral a1–2 chaetae often with median filaments (Figure 43B and Figure 52B) … 15
  - Th II only with mic (Figure 38A, Figure 41A, and Figure 47A) and labral chaetae ever smooth (Figure 40B) … 16
15. Prelabral and labral p0–2 chaetae weakly ciliate (Figure 43B); basomedian and basolateral labial fields with A1–5 weakly ciliate and M2, E, and L1–2 multiciliate (Figure 43F); Th II with 1 posterior mac (p3); Abd IV only with 1 central mac (B5) (Figure 44C); and unguis m.t. subequal to basal tooth in length (Figure 45F) … *P. cearensis* sp. nov.
  - Prelabral and labral p0–2 chaetae smooth (Figure 52B); basomedian and basolateral labial fields with a1–5 smooth and M2, E, and L1–2 weakly ciliate (Figure 52F); Th II with 2 posterior mac (p2–3); Abd IV only with 3 central mac (B5–6 and C1) (Figure 53C); and unguis m.t. larger than basal teeth (Figure 54F) … *P. mitodentunguilata* sp. nov.
16. Prelabral chaetae smooth … 17
  - Prelabral chaetae ciliate … 18
17. Tibiotarsal modified chaetae type IV (Figure 39E) and unguis slender in sickle-shape and m.t. absent, unguiculus sword-shaped with all lamellae smooth (Figure 39F) … *P. alfanjeunguiculata* sp. nov.
  - Tibiotarsal modified chaetae type V (Figure 42E) and unguis wide and m.t. present, unguiculus lanceolate with pe lamella serrate (Figure 42F) … *P. aphelabiata* sp. nov.
18. Abd II–IV with 1 (m3), 0, and 3 (B5–6 and C1) central mac (Figure 47B,C) … 19
  - Abd II–IV with different formula (Figure 56B,C and Figure 59B,C) … 21
19. Tibiotarsal modified chaetae never type I (Figure 48E and Figure 51E) and tenent hair acuminate and shorter than unguis length, unguis a.t. absent (Figure 48F and Figure 51F) … 20
  - Tibiotarsal modified chaetae type I (Figure 63E) and tenent hair capitate and subequal to unguis length, unguis a.t. present (Figure 63F) … *P. spurimarianensis* sp. nov.
20. Basomedian and basolateral labial fields with m2, e, and l1–2 smooth and postlabial G1–4 chaetae weakly ciliate (Figure 46F); tibiotarsal modified chaetae type IV (Figure 48E); and collophore posteriorly with 5 ciliate chaetae and 1 spine (Figure 48G) … *P. diamantinensis* sp. nov.
  - Basomedian and basolateral labial fields with M2, E, and L1–2 unilaterally ciliate and postlabial G1–4 chaetae clear ciliate (Figure 49F); tibiotarsal modified chaetae type III (Figure 51E); and collophore posteriorly with 2 ciliate chaetae and 2 spines (1 unpaired) (Figure 51G) … *P. marianensis* sp. nov.
21. Head M2 mac present (Figure 55E); Abd II with 2 central mac (m3–3e), and Abd IV with B6 mac (Figure 56B,C); tibiotarsal modified chaetae type IV (Figure 57E); and unguiculus ai lamella acuminate and pe serrate (Figure 57F) … *P. neriae* sp. nov.
  - Head M2 mac absent (Figure 58E); Abd II with 1 central mac (m3), and Abd IV devoid of B6 mac (Figure 59B,C); tibiotarsal modified chaetae type VII (Figure 60E); and unguiculus ai lamella acuminate and pe serrate (Figure 60F) … *P. pusilla* sp. nov.
22. Prelabral chaetae weakly ciliate on all branches (Figure 66B); head M2 mac present (Figure 66E); and Th II only with posterior mic (Figure 67A) … 23
  - Prelabral chaetae with elongated cilia on branches (Figure 78B); head M2 mac absent (Figure 78E); and Th II with 1 posterior mac (p3) (Figure 79A) … *P. prelabruscervata* sp. nov.
23. Labral p0–2 chaetae weakly ciliate (Figure 64A); unguis lamellae wide and with 3–4 unequal inner teeth (b.a., b.p., m.t., and a.t.); unguiculus ai lamella acuminate (Figure 65A and Figure 71E); and collophore anteriorly with less than 8 chaetae (Figure 68F and Figure 74F) … 24
  - Labral p0–2 chaetae smooth (Figure 75B); unguis lamellae slender and with 2 small equal inner teeth (b.a. and b.p.), m.t. vestigial on basal half; unguiculus ai lamella truncate (Figure 77E); and collophore anteriorly with 10 chaetae (Figure 77F) … *P. phyllunguiculata* sp. nov.
24. Unguis a.t. present and tenent hair capitate (Figure 65A) … 25
  - Unguis a.t. absent and tenent hair acuminate (Figure 71E) … *P. macrolignicephala* sp. nov.
25. Labral a1–2 chaetae smooth, without modifications (Figure 69B); basomedian and basolateral labial fields with A1–5 weakly ciliate and M1–2, E, and L1–2 completely ciliate, as well as postlabial G1–4 chaetae (Figure 64D and Figure 72F) … 26
  - Labral a1–2 chaetae with 1–2 lateral filaments, respectively (Figure 66B); basomedian and basolateral labial fields with all chaetae unilaterally ciliate, as well as postlabial G1–4 chaetae (Figure 66F) … *P. chimerambigua* sp. nov.
26. Abd II with 2 central mac (m3–3e) (as Figure 56B); unguis with b.a. and b.p equal; and unguiculus pe lamella serrate (Figure 65A) … *P. ambigua*
  - Abd II with 1 central mac (m3e) (Figure 70B); unguis basal inner teeth unequal (b.a. and b.p.), b.p. larger; and unguiculus pe lamella smooth (Figure 71E) … *P. parambigua* sp. nov.

## 4. Discussion

There are now 27 eyeless *Pseudosinella* species recorded from Brazil, of which 11 are from the group with one larger tooth on unguiculus outer lamella (*petterseni* group), one presents unguiculus outer lamella toothless, 10 hold prelabral chaetae undivided, while the other six hold prelabral chaetae bifurcate.

At this time, it is difficult to state if prelabral and labral chaetae modifications observed in part of the new species could hold phylogenetic signal and delimite Neotropical species group, since such distinct morphology is unknown to most species from North America [6,7,48]. Furthermore, these modifications are not unique to *Pseudosinella*, since bifurcate prelabral chaetae also occur in other genera of Entomobryoidea such as *Amazhomidia*, *Entomobrya*, *Orchesella*, *Plumachaetas*, and *Pseudodicranocentrus* [59,60,61,62,63]. In this same sense, filaments on labral a1 and a2 chaetae as well as ciliation on labral p0–2 chaetae and b.c. of maxillary palp are modifications also seen in *Lepidocyrtus* species [43,64,65,66]. These labral modifications may indicate that Brazilian *Pseudosinella* could have arisen from different branches of *Lepidocyrtus* or that such morphology emerged independently within *Pseudosinella*, and similarities concerning such features with *Lepidocyrtus* taxa are due to parallelism [17,21,22].

The eyes reduction and presence of a large outer tooth on unguiculus pe lamella (*petterseni* group) seen in some *Pseudosinella* species are troglomorphic characteristics quite certainly homoplastic [17], since they are also present in other Entomobryoidea genera such as *Alloscopus*, *Coecobrya*, *Heteromurtrella*, *Sinella*, and Cyphoderinae as a whole [13,67,68,69,70]. Other aggravating factors are that an unguiculus outer tooth is present in species with and without eyes besides interspecific inconsistency in the number of eyes, as observed in at least nine American species [7]. Although there are indications that eyes reduction is homoplastic within Lepidocyrtinae, unguiculus morphology (e.g., presence/absence of external tooth) should be further investigated phylogenetically to reveal if it may hold significance in splitting *Pseudosinella* into natural subgroups.

It is also not clear if the dorsal pattern of macrochaetotaxy is useful to support ingroups of *Pseudosinella* since the same patterns occur in different species regardless of the shape of the unguis and unguiculus [24,32,50,52,56]. Even though such patterns are similar among remarkably different species, Holarctic taxa tend to have a larger number of macrochaetae [7,52,56] while the Holotropical species hold a smaller number of them, as seen in most of the Brazilian new species. Curiously, the dorsal macrochaetae reduction in tropical species was also reported to other Entomobryidae genera like *Seira* and *Lepidocyrtus* [16,36,71] and it may represent some trend in the adaptation to tropical areas, possibly related to high temperature.

## 5. Conclusions

Due to this difficulty in validating species groups using morphology in disjoint regions without a proper phylogenetic study, for now, we cannot assume if they are natural groups or that the diagnostic characters that we are using are homoplastic for troglomorphic springtails [17]. Therefore, a phylogenetic analysis is in need to test whether the genus is really polyphyletic, and if so, how many branches of “*Pseudosinella*” there are and which species groups (inner taxa) are valid. Also, it is important to understand which morphological features can support the lineages of *Pseudosinella*. So far, phylogenetic studies performed with Lepidocyrtinae had few representatives of the genus and were not intended to verify its internal relationships [2,21,22,23].

## Figures and Tables

**Table 1:** Comparison (per side) among 31 species of *Pseudosinella* recorded from Brazil.

	Pigments	Eyes	Prelabral	Head mac Series					Th II–Abd IV mac	Tenent	Unguiculus
Species			Chaetae	An	A	M	S	Pa	Formula	Hair	Outer Tooth
*P. acantholabrata*	–	–	ciliate	8	3	1	0	0	00|010+21+2	capitate	+
*P. alba* [8]	+	2	ciliate	?	3	1	0	0	10|010+?1+2	variable	–
*P. alfanjeunguiculata*	–	–	smooth	10(9)	3	1	0	0	00|010+31+2	capitate	–
*P. ambigua*	–	–	ciliate	8	3	1	0	0	00|020+21+2	capitate	–
*P. aphelabiata*	–	–	smooth	9	4(3)	1(0)	0	0	00|010+31+2	capitate	–
*P. biunguiculata*	–	–	ciliate	7	3	0	0	1	00|020+21+1	acuminate	+
*P. brevicornis* [14]	–	–	?	?	?	?	?	?	?	acuminate	–
*P. brumadinhoensis*	–	–	ciliate	8	3	1	0	0	22|010+21+2	acuminate	+
*P. cearensis*	–	–	ciliate	8	3	0	0	0	10|010+20+1	capitate	–
*P. chimerambigua*	–	–	ciliate	9	3	1	0	0	00|010+21+2	capitate	–
*P. diamantinensis*	–	–	ciliate	10	3	1(0)	0	0	00|010+2(3)1+2	acuminate	–
*P. dubia* [8]	+	5–6	?	?	3	0	0	0	00|010+?0+3	capitate	–
*P. guanhaensis*	–	–	ciliate	7	3	0	0	1	00|020+21+1	capitate	+
*P. keni*	–	–	ciliate	7	3	0	0	0	00|010+11+2	acuminate	+
*P. labiociliata*	–	–	ciliate	7	3	0	0	0	00|010+21+2	acuminate	+
*P. labruspinata*	–	–	ciliate	7	3	0	0	0	22|010+11+2(3)	acuminate	+
*P. macrolignicephala*	–	–	ciliate	10	3	1	0	0	00|010+21+2	acuminate	–
*P. marianensis*	–	–	ciliate	10(9)	3	1	0	0	00|010+21+2	acuminate	–
*P. mitodentunguilata*	–	–	smooth	8(7)	3	0	0	0	21(0)|010+21+2	capitate	–
*P. neriae*	–	–	ciliate	9	4(3)	1	0	0	00|020+21+2	acuminate	–
*P. octopunctata* [8]	+	4–5	?	6	3	1	1	0	10|030+?1+2	capitate	–
*P. paraensis*	–	–	smooth	8	5	1	0	0	00|010+21+1	acuminate	+
*P. parambigua*	–	–	ciliate	9	3	1	0	0	00|010+21+2	capitate	–
*P. phyllunguiculata*	–	–	ciliate	12	3	1	0	0	00|010+21+2	acuminate	–
*P. prelabruscervata*	–	–	branched	10	3	0	0	0	10|010+21+2	acuminate	–
*P. pusilla*	–	–	ciliate	9	3	0	0	0	00|010+21+1	acuminate	–
*P. serpentinensis*	–	–	ciliate	8	4	1	0	0	00|010+21+2	acuminate	+
*P. spurimarianensis*	–	–	ciliate	10	3	1	0	0	00|010+21+2	capitate	–
*P. taurina*	–	–	ciliate	7	3	0	0	0	10|010+01+2	capitate	+
*P. triocellata* [17]	+	3	ciliate	7	3–4	0	1	0	00|010+21+1	acuminate	–
*P. unimacrochaetosa*	–	–	ciliate	6	3	0	0	0	10|010+11+2	capitate	+

Symbols used to represent the morphological characteristics: (+) present; (–) absent; (?) unknown. Dorsal chaetotaxy was interpreted as A0–3 (R0–2), M2 (R3/S), S3 (T), and Pa5 (Po) for head mac series and, for Th II–Abd IV formula, as Th II to Abd II (central), Abd III central (m3) + lateral (m7e, pm6, and p6), and Abd IV anterior (C1 as P_1_) + posterior (B5–6 as M_1–3_) [7,20].

**Table 2:** Comparison (per side) among *Pseudosinella* species described from Americas with large tooth on pe lamella of unguiculus, basomedian labial field with a1–5 smooth, and devoid of pigments, eyes, apical bulb on Ant IV and unguis apical tooth (a.t.).

		*Pseudosinella Species*											
		*biunguiculata*	*certa*	*espana*	*espanita*	*federicoi*	*guanhaensis*	*josemarii*	*parattenuata*	*petterseni*	*rolfsi*	*sera*	*violenta*
Characteristics	References:	[43,44] §	[5,7,20]	[6,7,26]	[6,7]	[27]	[14] §	[20]	[27]	[26,46,47,48]	[7,20,49]	[5,7,20]	[5,7,20,45]
	Locality:	Guatemala	USA	USA	USA	Argentina	Brazil	USA	USA	Introduced: USA and	USA	USA	USA
	Habitat:	ant nest	cave	cave	cave	?	cave	cave	?	Costa Rica	beneath stones	spruce needles	soil
Ant III–II ratio		III < II	III = II *	III = II *	?	III < II	III < II	?	?	?	III = II	III = II *	III < II
Ant III e sens		+	?	?	+	?	+	?	?	?	?	?	?
Prelabral chaetae		C	?	?	C	?	C	C	?	?	?	?	?
Labral chaetae	a1	S and tkc	?	?	S	?	S and tkc	S	?	?	?	?	?
	a2	S and tkc	?	?	S	?	S and tkc	S	?	?	?	?	?
	m0–2	S	?	?	S	?	S	S	?	?	?	?	?
	p0–2	S	?	?	S	?	S	S	?	?	?	?	?
Papilla E l.p.	shape	?	?	?	?	curved	conical/curved	curved	curved	?	?	?	curved
	size	?	?	?	?	=appendix base	<appendix base	</=appendix base	=appendix base	?	?	?	>appendix base
Head chaetotaxy	A2 (R1)	M	M	M	M	m	M	M	M	M	m	M	m
	A3 (R2)	M	M	M	M	m	M	M	m	m	m	M	m
	M1 (R3)	m	m	m	m	m	m	m	M	m	m	m	m
	M2	m	m	m	m	m	m	m	m	m	m	m	m
	S2 (T)	m	m	m	M	m	m	m	M	M	m	m	m
	S3 (S)	m	m	M	M	m	m	m	m	m	m	m	m
	Pa5	M	m	M	m	M	M	M	M	M	m	m	M
	Pa3, Pm3	m	m	M	m	m	m	m	m	m	m	m	m
Basomedian and basolateral labial chaetae	M1	C	C	C	C	S	C un.	C	C	C	C	C	C
	M2	–	C	S	S	S	–	C	C	C	C	C	C
	r	spn	spn	spn	spn +/–	spn	spn	spn	?	S	spn	spn	spn
	E, L1–2	C	C	S	S	S	C un.	C	C	C	C	C	C
Postlabial chaetae	H1	–	?	?	?	+	–	+	+	?	?	?	+
	X	+	?	?	?	?	+	?	?	?	?	?	+
	X4	–	?	?	?	?	–	+	?	?	?	?	?
Th II mac		–	1	2–3	3	–	–	1	4	2	–	–	–
Th III mac		–	–	1	2	–	–	1	–	2	–	–	–
Abd II	a2	M	m	m	m	M	M	m	M	M	m	m	M
	m3	M	M	M	M	M	M	M	M	M	m	M	M
	m3e	m	m	M	m	M	m	m	m	m	M	m	M
Abd III	pm6	M	?	?	M	M	M	M	M	?	?	?	M
	p6	M	?	?	M	m	m	M	m	?	?	?	M
Abd IV inner mac		2	2	3	3	2	2	2	2?	3	1–2	2	2
Trochanteral organ		?	?	?	5–7	?	17	11	?	?	?	?	?
Unguis outer teeth	paired	hook	?	normal	?	?	hook	?	?	normal	normal	normal	hook
Unguis inner tooth	wide	wide	slender	slender	slender	wide	slender distally	wide	wide	wide	wide	wide	slender distally
	ratio	b.p. > b.a.	b.p. > b.a.	b.p. = b.a.	b.p. = b.a. > m.t.	b.p. > b.a. = m.t.	b.p. > b.a.	b.p. > m.t. > b.a.	b.p. > b.a. = m.t.	b.p. > b.a. = m.t.	b.p. > b.a. = m.t.	b.p. = m.t. > b.a.	b.p. > b.a. > m.t.
	b.p.	divided	normal	normal	normal *	normal	divided	normal	normal	normal	normal *	normal	normal *
	median	–	–	–	+ *	+	–	+	+	+	+	+	+
Unguiculus ai lamella		acuminate	acuminate	acuminate	acuminate	acuminate	excavate	acuminate	acuminate	acuminate	truncate	acuminate	acuminate
Tenent hairs	apex	acuminate	acuminate	acuminate	acuminate	acuminate	capitate	acuminate	acuminate	capitate	capitate	capitate	capitate
	ratio	=unguiculus	<unguiculus	<unguiculus	<unguiculus	=unguiculus	>unguiculus	<unguiculus	>unguiculus	>unguiculus	>unguiculus	>unguiculus	>unguiculus
Collophore	anterior	6C	?	?	5–8C	5S *	6C	8C	5S *	?	?	?	8C
	posterior	1S, 1C, 1spn	?	?	4–6C	2S *	1S, 1C, 1spn	1S, 2C	2S *	?	?	?	1S, 3C?
	lateral	6S	?	?	6–8S, 1–2C	?	6S	7	?	?	?	?	?
Manubrium dorsal smooth chaetae		4	?	?	?	?	4	–	?	?	?	?	?
Manubrial plate	chaetae	3	?	?	4–5	3	4	4	?	?	?	6–7	?
	psp	2	?	?	?	2	2	2	?	?	?	?	?
Dens smooth chaeta	proximal	1	?	?	?	?	1	–	?	?	?	?	?
Mucronal spine length		=B apex	>B apex	–	–	?	=B apex	=B apex	?	?	>B apex	=B apex	=B apex
Mucro tooth size		B > A	B < A	B < A	B < A	?	B > A	B < A	?	?	B = A	B = A	B < A

Symbols used to represent the morphological characteristics: (C) ciliated chaeta; (S) smooth chaeta; (M) mac; (m) mic; (bif) bifurcate; (spn) spine; (psp) pseudoporus; (tkc) thicker; (–) absent; (*) dubious characteristic; (§) personal observation; (?) unknown characteristic.

**Table 3:** Comparison (per side) among new species of *Pseudosinella* from Brazil with large tooth on pe lamella of unguiculus, labial field with r chaeta reduced, Abd II with 2 mac (m3, m5), and furcula only with ciliate chaetae, devoid of pigments, eyes and apical bulb on Ant IV.

		*Pseudosinella Species*								
		*acantholabrata*	*brumadinhoensis*	*keni*	*labiociliata*	*labruspinata*	*paraensis*	*serpentinensis*	*taurina*	*unimacrochaetosa*
		sp. nov.	sp. nov.	sp. nov.	sp. nov.	sp. nov.	sp. nov.	sp. nov.	sp. nov.	sp. nov.
Characteristics	Locality:	Minas Gerais	Minas Gerais	Minas Gerais	Minas Gerais	Minas Gerais	Pará	Minas Gerais	Pará	Minas Gerais
Ant IV	a sens	+	+	+	+	+	–	+	+	+
	e sens	–	+	+	+	+	+	+	+	+
Ant III	apical sens	club	club	club	lance	conical	finger	lance	finger	finger
	e sens	–	+	–	–	–	+	+	–	–
	f sens	–	–	–	+	–	–	–	–	–
Ant III–II ratio		III =/< II	III = II	III =/< II	III = II	III = II	III < II	III =/< II	III < II	III = II
Clypeal chaetotaxy	l	3	2	2	2	2	2	3	2	3
	ft	1	2	1	0	2	3	1	1	1
	pf	3	2	2	2	3	3	4	2	2
Prelabral chaetae		C	C	C	C	C	S	C	C	C
Labral chaetae	a1	tck and 0–2f	0f	tck and 0f	tck and 0f	tck and 1f	0f	tck and 0f	horn and 3–5f	tck and 0–1f
	a2	tck and 1–2f	0f	tck and 0f	tck and 0–1f	tck and 2–3f	0f	tck and 0f	horn and 3–5f	tck and 0–2f
	m0	tck and 2–3f	0f	tck and 0f	tck and 1–4f	tck and 2–3f	0f	tck and 0f	0 or 2–5f	tck and 1–2f
	m1	tck and 1–5f	0f	tck and 0–2f	tck and 1–4f	tck and 1–4f	0f	tck and 0–1f	0 or 2–5f	tck and 1–2f
	m2	tck and 1–5f	0f	tck and 0–2f	tck and 1–5f	tck and 2–4f	0f	tck and 0–1f	0 or 2–5f	tck and 2–4f
	p0–2	C	S	C	C	C elongated	S	C	C	C
Labial proximal chaetae	lpc1	2–3f	2–3f	2–3f	3f	3f	0f	2f	3–5f	2–3f
	lpc3, 7	2–3f	2–3f	2–3f	3f	2f	0f	2f	3–5f	2–3f
	lpc4	sm and 0f	2–3f	2–3f	3f	2f	0f	2f	3–5f	2–3f
	lpc6	sm and 0f	sm and 0f	sm and 0f	sm and 2f	sm and 2f	sm and 0f	sm and 2f	sm ans 3f	sm and 0f
Maxillary palp b.c.		WC	S	C un.	C un.	C un.	S	C un.	WC	C un.
Papilla B appendages filaments	a1	–	–	–	–	1	–	–	–	–
	b4	–	–	–	–	3	–	–	–	–
Papilla E l.p.	shape	finger/sinuosus	finger/curved	finger/curved	pointed/curved	finger/straight	finger/curved	conical/straight	acuminate/straight	finger/straight
	size	>appendix base	<appendix base	=appendix base	=appendix base	</=appendix base	>appendix base	>appendix base	=appendix base	=appendix base
Head dorsal chaetotaxy	A2a	–	–	–	–	–	M	–	–	–
	M2	M	M	m	m	m	M	M	m	m
	S4	m	m	m	m	m	m	m	m	–
	S6	m	–	m	–	–	m	m	m	m
Basomedian and basolateral labial chaetae	A1–4	WC un.	WC un.	WC un.	C	C un.	S	WC un.	C un.	C
	A5	WC un.	S	S	C	C un.	S	WC un.	S	S
	M, E, L	C un.	C un.	C un.	C	C un.	MC	C	C un.	C
Postlabial chaetae	H1	–	+	–	+	–	+	–	–	+/–
	X	–	+	–	–	–	+	+	–	–
Th II	p3	m	M	m	m	M	m	m	M	m
	p5	m	M	m	m	M	m	m	m	M
Th III	p2	m	M	m	m	M	m	m	m	m
	p3	m	M	m	m	M	m	m	m	m
Abd I	a3	m	m	–	m	m	m	m	–	m
	a6	–	–	–	–	–	m	m	m	m
Abd II	a6	m	–	–	m	–	m	–	m	–
	p5p	m	–	m	m	–	m	–	m	m
Abd III	pm6	M	M	M	M	M	M	M	mes.	M
	p6	M	M	mes.	M	mes.	M	M	m	mes.
Abd IV	B6 chaeta	M	M	M	M	M	m	M	M	M
	outer mac	13	4	5	5	5	11	9	6	5
	inner sens	3	3	2	2	4	3	3	3	3
Trochanteral organ	chaetae	11	6	11	9	8	12	9	7	10
Tibiotarsal modified chaetae	formula	1, 3, 3	1, 3, 3	1, 3, 3	1, 3, 3	1, 3, 3	0, 0, 1	1, 3, 3	1, 3, 3	1, 3, 3
	shape	type II	type II	type II	type II	type II	type VI	type II	type II	type II
Unguis outer paired teeth	shape	straight	hook	hook	hook	hook	hook	hook	hook	hook
	location	¼	¼	⅓	¼	⅓	⅕	¼	½	¼
Unguis inner teeh	size	wide	slender	slender distally	slender distally	wide	slender distally	wide	slender	wide
	ratio	b.p. > b.a. = m.t.	b.p. > m.t. > b.a.	b.p. > b.a.	b.p. > b.a.	b.p. > b.a. = m.t.	b.p. > b.a. = m.t.	b.p. > b.a. = m.t.	b.p. > b.a. = m.t.	b.p. > b.a. > m.t. > a.t.
	b.p.	normal	normal	divided	divided	divided	normal	normal	normal	divided
	m.t.	⅓	>½	–	–	⅓	½	>½	⅓	⅓
	a.t.	–	–	–	–	–	–	–	–	¼
Unguiculus ai lamella		acuminate	acuminate	excavate	truncate	truncate	excavate	excavate	excavate	acuminate
Tenent hairs	apex	capitate	acuminate	acuminate	acuminate	acuminate	acuminate	acuminate	capitate	capitate
	ratio	>unguiculus	<unguiculus	=unguiculus	=unguiculus	=unguiculus	>unguiculus	<unguiculus	>unguiculus	>unguiculus
Collophore	anterior	6C	6C	6C	6C	6C	4C	6C	4C	7C
	posterior	1S, 2C, 1spn	1S, 2C	1S, 2C, 1spn	1S, 1spn	1S, 1C, 1spn	1S, 2C, 1spn	1S, 2C, 1spn	1S, 1spn	1S, 1C, 1spn
	lateral	7S	2S, 4C	6S	2S, 3C	5C unilat.	7S	7S	5C	6S
Manubrium ventral scales	subapical	3	3	3	3	3	3	4	3	3
	apical	8	11	7	6	9	7	9	3–4	9
Manubrial plate	chaetae	4	5	4	4	4	4	4–5	3	4–5
	psp	2	2	2	2	2	2	2	1	2
Mucro tooth size		B > A	B = A	B = A	B > A	B = A	B > A	B = A	B = A	B < A

Symbols used to represent the morphological characteristics: (C) ciliated chaeta; (S) smooth chaeta; (M) mac; (m) mic; (MC) multiciliate; (WC) weakly ciliate; (spn) spine; (psp) pseudoporus; (f) filaments; (sm) smaller; (un.) unilaterally ciliate; (a.t.) unguis apical tooth; (tkc) thicker; (–) absent.

**Table 4:** Comparison (per side) among *Pseudosinella* species from Americas devoid of eyes and apical bulb on Ant IV and unguiculus with all acuminate lamellae.

			*Pseudosinella Species*										
		argentea *	*christianseni*	*erehwon*	*extra*	*flatua*	*folsomi*	*granda*	*nata*	*orba*	*pecki*	strinatii	*vespera*
	References:	[7,20,53]	[7,54]	[6]	[6]	[6]	[7,55]	[5]	[5,7]	[7,48]	[5,7]	[51]	[6]
	Locality:	USA	USA	USA	USA	USA	USA	USA	USA	USA	USA	Mexico	USA
Characteristics	Habitat:	graves	cave	cave	cave	cave	on boards	cave	cave	cave	cave	cave	cave
Ant./body ratio		Ant < body	Ant > body	Ant < body	Ant < body	Ant < body	Ant < body	Ant < body	Ant < body	Ant < body	Ant < body	?	Ant < bod
Ant III–II ratio		III > II	III = II	III < II	III < II	III = II	III = II	III < II	III = II	III = II	III = II	III > II	III < II
Prelabral chaetae		C	?	S	S	S	?	C	?	?	?	?	S
Labral chaetae	a1	S	?	S	S	S	?	S	?	?	?	?	S
	a2	S	?	S	S	S	?	S	?	?	?	?	S
	m0–2	S	?	S	S	S	?	S	?	?	?	?	S
	p0–2	S	?	S	S	S	?	S	?	?	?	?	S
Head chaetotaxy	A2 (R1)	M	M	M	M	M	M	M	M	M	M	M	M
	A3 (R2)	M	M	M	M	M	m	M	M	M	M	M	M
	M1 (R3)	m	m	m	M	m	m	m	m	M	m	m	m
	M2	m	m	m	m	m	m	m	m	m	m	M	M
	S2 (T)	m	m	m	m	m	m	m	m	M	m	m	m
	S3 (S)	m	m	m	m	m	m	m	m	M	m	M	m
	Pa5	m	m	m	m	m	m	m	m	m	m	m	m
	Pa3, Pm3	m	m	m	m	m	m	m	m	m	m	m	m
Basomedian and basolateral labial chaetae	M1	C	S	C	C	C or S/sm	S	C	S	C	S/sm	S	S
	M1e	–	S	–	–	–	S	–	–	S	–	–	–
	M2	C	S	S	S	S	S	C	S	S	S	S	S
	r	spn	S/sm	C	C	S/sm	spn	spn	spn	spn	C/sm	spn	–
	E, L1–2	C	S	S	S	S	S	C	S	S	S	S	S
Cephalic groove chaetae		4C	6–8S	4C	4C	3S, 1C	4S	4C	?	?	1–2S, 4–5C	3–4S	4C
Th II mac		–	–	2	2	–	–	–	–	2–3	–	1	3
Th III mac		–	–	1	1	–	–	–	–	3(1)	–	–	2
Abd II	a2	m	m	m	m	m	M	m	m	m	m	M	m
	m3	M	M	M	M	M	M	M	M	M	M	M	M
	m3e	m	m	m	m	m	M	m	m	m	m	m	m
Abd IV mac		2	3–4	2	2	2	3	2	2	2–3	2	2	2
Trochanteral organ		?	?	5–6	6–10	9–11	?	5–6	?	?	?	?	6–7
Unguis inner side	lamellae	wide	machete-shape	wide	wide	wide	wide	wide	wide	wide	wide	wide	wide
	ratio	b.p. > b.a. = m.t.	b.p. = b.a. > m.t.	b.p. > b.a. = m.t.	b.p. > b.a. = m.t.	b.p. = b.a. = m.t.	b.p. = b.a. = m.t.	b.p. > b.a. = m.t.	b.p. = b.a. > m.t.	b.p. > b.a. = m.t.	b.p. = b.a. > m.t.	b.p. = b.a. = m.t. > a.t.	b.p. > b.a. < m.t.
	m.t.	>½	>½ (+/–)	⅓	½	>½	⅓	⅓	¼	½	⅓	⅓	<⅓
	a.t.	–	–	–	–	–	–	–	–	–	–	¼	–
Unguiculus lamellae	ai	acuminate	excavate	acuminate	acuminate	acuminate	acuminate	acuminate	acuminate	acuminate	acuminate	acuminate	acuminate
	pe	serrate	smooth	serrate	serrate	smooth	smooth	smooth	smooth	serrate	smooth	smooth	smooth
Tenent hairs	apex	capitate	acuminate	capitate/acuminate	acuminate	acuminate	capitate	acuminate	capitate	acuminate	acuminate	capitate/acuminate	acuminate
	ratio	=unguiculus	<unguiculus	=unguiculus	=unguiculus	=unguiculus	>unguiculus	=unguiculus	>unguiculus	=unguiculus	>unguiculus	>unguiculus	?
Collophore	anterior	9C	?	9–10C	7–8C	11–13C	?	9C	?	?	?	?	7C
	posterior	10?	?	5–6C	5S, 2C	7WC	?	7C	?	?	?	?	3S, 1C
	lateral	4S, 4C	?	4–5S, 4C	3S, 5C	6S, 6C	?	4S, 4–5C	?	?	?	?	7S, 1–2C
Manubrial plate chaetae		8	?	4	2	7–11	?	5–7		?	?	?	4
Mucronal spine length		=B apex	=B apex	>B apex	<B apex	>B apex	=/>B apex	>B apex	>B apex	=B apex	=B apex	=B apex	=B apex
Mucro tooth size		B = A	B < A	B = A	B < A	B < A	B > A	B < A	B < A	B < A	B < A	B = A	B < A

Symbols used to represent the morphological characteristics: (C) ciliated chaeta; (WC) ciliated chaeta; (S) smooth chaeta; (M) mac; (m) mic; (bif) bifurcate; (spn) spine; (sm) smaller; (psp) pseudoporus; (–) absent. (*) Indicates variations in basomedian and basolateral labial fields and tenent hairs are not considered here, since observations were made on specimens from different locations of the United States [7]. We believe it is possibly such populations could belong to different species.

**Table 5:** Comparison (per side) among new species of *Pseudosinella* from Brazil with prelabral chaetae undivided (normal), unguiculus pe lamella toothless, basomedian labial field with r chaeta reduced, Abd II with m3 and m5 mac, Abd II with pm6 and p6 mac, furcula only with ciliate chaetae, devoid of pigments, eyes and apical bulb on Ant IV.

		*Pseudosinella Species*								
		*alfanjeunguiculata*	*aphelabiata*	*cearensis*	*diamantinensis*	*marianensis*	*mitodentunguilata*	*neriae*	*pusilla*	*spurimarianensis*
		sp. nov.	sp. nov.	sp. nov.	sp. nov.	sp. nov.	sp. nov.	sp. nov.	sp. nov.	sp. nov.
Characteristics	Locality:	Minas Gerais	Minas Gerais	Ceará	Minas Gerais	Minas Gerais	Minas Gerais	Minas Gerais	Pará	Minas Gerais
Ant IV	e sens	+	+	–	+	–	+	+	–	+
	f sens	+	–	–	+	–	+	–	–	+
Ant III	apical sens	slender	swollen	slender	slender	swollen	swollen	slender	slender	swollen
	e sens	–	–	+	+	–	+	+	–	–
	f sens	–	–	–	–	–	–	+	–	–
	h sens	–	+	+	+	–	–	–	–	–
Ratio Ant III–II		III < II	III < II	III = II	III < II	III < II	III < II	III < II	III < II	III < II
Prelabral chaetae		S	S	C	C	C	S	C	C	C
Labral chaetae	a1	S	S	2f	S	S	tck and 0–1f	S	S	S
	a2	S	S	2f	S	S	tck and 0–1f	S	S	S
	m0	S	S	3–4f	S	S	S	S	S	S
	m1	S	S	3–4f	S	S	S	S	S	S
	m2	S	S	3–4f	S	S	S	S	S	S
	p0–2	S	S	C	S	S	S	S	S	S
Labial proximal chaetae	lpc3	S	S and sm	S	S	S and sm	S	S and sm	S and sm	S and sm
	lpc4	S	S	WC	S	S	S	S	S and sm	S
	lpc6	S and sm	S and sm	WC and sm	S and sm	S and sm	S and sm	S and sm	S and sm	S and sm
Maxillary palp b.c.		WC	WC	WC	WC	WC	WC	S	WC	WC
Papilla E l.p.	shape	finger/sinuosus	finger/sinuosus	finger/curved	finger/sinuosus	finger/sinuosus	finger/straight	finger/sinuosus	finger/curved	finger/straight
	size	>appendix base	>appendix base	>appendix base	>appendix base	>appendix base	>appendix base	>appendix base	>appendix base	>appendix base
Head dorsal chaetotaxy	M2	M	M or m	m	M or m	M	m	M	m	M
Basomedian and basolateral labial chaetae	A1–5	S	S	WC	S	S	S	S	S	S
	M1	S or WC un.	S	MC	C un.	C un.	C	C	C un.	C or C un.
	M1e	–	S sm +/–	–	C un./sm +/–	–	–	–	–	–
	M2	S	S	MC	S	C un.	WC	C	C un.	C or C un.
	E, L1–2	S	S	MC	S	C un.	WC	C	C un.	C or C un.
Postlabial chaetae	G1–4	WC	WC	C	WC	C	WC	C	C	C
	H1	C	WC +/–	C	C	–	C +/–	–	C	C
	H2	C	WC +/–	–	C	C	C	C	C	C
	H3	WC	–	C	C	C	WC	C	C	C
	X	C	C	–	C	C	C	C	C	–
	X4	WC	C	C	C	C	WC	C	C	C
	spn	1	2	2	1	1	1	1	1	1
Th II	p3	m	m	M	m	m	M	m	m	m
	p5	m	m	m	m	m	M	m	m	m
Th III	p3	m	m	m	m	m	M/m	m	m	m
Abd I	a3	m	m	m	m	m	m	m	–	m
	a6	m +/–	m	m	m	–	–	m	m	–
Abd II	a6	fan +/–	m	m	m	m	m +/–	m	m	m
	m3e	m	m	m	m	m	m	M	m	m
Abd III	m7e	M	M	m	M/m	m	m	m	m	m
Abd IV	B6 mac	+	+	–	+	+	+	+	+	+
	C1 mac	+	+	–	+	+	+	+	–	+
	outer mac	7	4	5	7	5	4	4	10–11	5
	inner sens	4	5	5	5	4	3–4	4	3	4
Trochanteral organ		15	12	14	15	12	11	12	8	12
Tibiotarsal modified chaetae	formula	1, 3, 3	1, 3, 3	1, 2, 1	1, 3, 3	1, 3, 3	1, 3, 3	1, 3, 3	1, 3, 3	1, 3, 3
	shape	type IV	type V	type VI	type IV	type III	type IV	type IV	type VII	type I
Unguis outer teeth	location	⅕	¼	>½	⅓	⅓	¼	¼	¼	½
Unguis inner teeh	size	slender	wide	wide	wide	wide	wide	wide	wide	wide
	ratio	b.p. > b.a.	b.p. > b.a. = m.t.	m.t. > b.p. = b.a. > a.t.		b.p. > b.a. = m.t.	m.t. > b.p. = b.a. < a.t.	b.p. > b.a. = m.t.	b.p. > b.a. = m.t.	b.p. > b.a. > m.t. > a.t
	m.t.	–	½	⅓	>½	⅓	>½	⅓	>½	⅓
	a.t.	–	–	⅙	–	–	⅕ +/–	–	–	⅕
Unguiculus lamella	ai	excavate	acuminate	acuminate	gently truncate	gently truncate	acuminate	acuminate	truncate	acuminate
	pe	smooth	serrated	serrated	serrated	serrated	serrated	serrated	smooth	serrated
Tenent hairs	apex	capitate	capitate	capitate	acuminate	acuminate	capitate	acuminate	acuminate	capitate
	ratio	<unguiculus	<unguiculus	=unguiculus	<unguiculus	=unguiculus	<unguiculus	<unguiculus	>unguiculus	>unguiculus
Collophore	anterior	7C	6C	7C	5–7C	5–7C	6–7C	8C	4–5	7–8C
	posterior	1–2S, 1C, 1spn	1S, 2C, 1spn	3C, 1spn	2S, 5C, 1spn	2S, 1C, 2spn	1–2S, 1–2C, 1spn	1S, 1C, 1spn	2S, 2C, 1spn	2S, 1C, 1spn
	lateral	7–8S, 0–1C	10S	10C	6S, 4C	7–8S, 1C	5S, 1C	3S, 1C	9S, 1C	8–9S
Manubrium ventral scales	subapical	5	3	3	4	3	4	4	4	5
	apical	10–11	12	8–9	11–12	11	8–9	10	6	9
Manubrial plate	chaetae	6–8	5–6	4–5	4–7	3–6	4–6	3–6	6–8	3–4
Mucronal spine length		>B apex	>B apex	=B apex	>B apex	=B apex	=B apex	=B	=B	>B
Mucro tooth size		B > A	B > A	B > A	B > A	B > A	B > A	B > A	B = A	B > A

Symbols used to represent the morphological characteristics: (C) ciliated chaeta; (S) smooth chaeta; (M) mac; (m) mic; (MC) multiciliate; (WC) weakly ciliate; (spn) spine; (psp) pseudoporus; (f) filaments; (sm) smaller; (un.) unilaterally ciliate; (tkc) thicker; (–) absent.

**Table 6:** Comparison (per side) among *Pseudosinella* species from Minas Gerais (Brazil) with prelabral chaetae bifurcate, maxillary palp b.c. weakly ciliate, Th III devoid mac, unguiculus pe lamella of toothless, basomedian labial field with r chaeta reduced, Abd II with m3 and m5 mac, Abd IV with 3 central mac (B5–6 and C1), tibiotarsal modified chaetae type III, furcula only with ciliate chaetae, devoid of pigments, eyes and apical bulb on Ant IV.

		*Pseudosinella Species*					
		*ambigua*	*chimerambigua*	*macrolignicephala*	*parambigua*	*phyllunguiculata*	*prelabruscervata*
Characteristics	References:	[14] §	sp. nov.	sp. nov.	sp. nov.	sp. nov.	sp. nov.
Ant III apical sens		slender	slender	swollen	swollen	slender	slender
Clypeal chaetotaxy	ft	3	3	2	3	3–5	5
	pf	3	5	3	5	3	3
Prelabral chaetae		C	C	C	C	C	elongated cilia
Labral chaetae	a1	S	1f/tck	S	S	S	S
	a2	S	2f/tck	S	S	S	S
	m0–2	S	S	S	S	S	S
	p0–2	WC	WC	WC	WC	S	WC
Labial proximal chaetae	lpc1	WC	WC	S	S	S	C
	lpc3	WC/sm	WC/sm	S	S/sm	S/sm	C/sm
	lpc4	WC/sm	WC/sm	S	S/sm	S/sm	C/sm
	lpc6	WC/sm	WC/sm	S/sm	S/sm	S/sm	C/sm
	lpc7	WC	WC	S	S	S	C
Papilla E l.p.	shape	finger/curved	finger/sinuosus	pointed/straight	finger/sinuosus	finger/sinuosus	finger/curved
	size	>appendix base	>appendix base	>appendix base	>appendix base	>appendix base	=appendix base
Basomedian and basolateral labial chaetae	A1–5	WC	HC un.	S	WC	S	MC
	M1	C	HC un.	HC un.	MC	C un.	MC
	M1e	–	–	–	–	–	MC
	M2	C	HC un.	HC un.	MC	C un.	MC
	E, L1–2	C	HC un.	HC un.	MC	C un.	MC
Postlabial chaetae	G1–4	C	HC un.	HC un.	C	C	C
	H1	C	C	–	C	C	C
	H3	C	–	C	–	C	C
	X	C	C	C	C	C	C
Head chaetotaxy	A2a	–	+	+	+	+	–
	M2	M	M	M	M	M	m
Th II	p3	m	m	m	m	m	M
	p6e?	–	–	m	m	–	–
Abd I	a3	m	m	m +/–	m	m	m
Abd II	m3e	M	m	m	m	m	m
	p5p	m	m	m	m	m +/–	m
	p5pe	–	–	–	–	–	m
Abd IV	outer mac	8	4	6	6	5	9
	inner sens	3	4	5	5	6	4
Trochanteral organ	chaetae	10–11	13	11	12	19	19
	psp	–	–	–	–	1	–
Tibiotarsal chaetae	formula	2, 3, 3	1, 1, 2	1, 1, 3	1, 2, 3	1, 2, 3	1, 1, 2
Unguis outer teeth	location	⅓	⅓	¼	⅓	⅓	⅓
Unguis inner tooth	size	wide	wide	wide	wide	slender	wide
	ratio	m.t. > b.p. = b.a. > a.t.	b.p. = b.a. = m.t. > a.t.	b.p. > b.a. = m.t.	b.p. > b.a. = m.t. > a.t.	b.p. = b.a.	b.p. = m.t. > b.a.
	b.a./b.p.	equal	equal	unequal	unequal	equal	unequal
	m.t.	⅓	⅓	⅓	⅓	–	>½
	a.t.	⅕	⅐	–	⅙	–	–
Unguiculus lamella	ai	acuminate	acuminate	acuminate	acuminate	truncate	acuminate
	pe	serrated	serrated	smooth	smooth	smooth	smooth
Tenent hairs	apex	capitate	capitate	acuminate	capitate	acuminate	acuminate
	ratio	>unguiculus	>unguiculus	<unguiculus	>unguiculus	>unguiculus	<unguiculus
Collophore	anterior	6	6	6	7	10	8
	posterior	1S, 2C, 2spn	4C, 1spn	1S, 3C, 2spn	4C, 1spn	2S, 6C, 1spn	7C, 1spn
	lateral	3S, 5C	8WC	10S	7S, 2C	8S, 2C	8C
Manubrium ventral scales	subapical	3	4	3	3	4	2
	apical	8	9	9	7	11	7
Manubrial plate	chaetae	5	4	5	5	5	6
	psp	2	2	2	2	2	2
Mucro tooth size		B > A	B > A	B = A	B > A	B = A	B = A

Symbols used to represent the morphological characteristics: (C) ciliated chaeta; (S) smooth chaeta; (M) mac; (m) mic; (HC) heavily ciliate; (MC) heavily ciliate; (WC) weakly ciliate; (spn) spine; (psp) pseudoporus; (f) filaments; (sm) smaller; (unilat.) unilaterally ciliate; (tkc) thicker; (§) personal observation; (–) absent.
